# Multidomain ribosomal protein trees and the planctobacterial origin of neomura (eukaryotes, archaebacteria)

**DOI:** 10.1007/s00709-019-01442-7

**Published:** 2020-01-03

**Authors:** Thomas Cavalier-Smith, Ema E-Yung Chao

**Affiliations:** Department of Zoology, University of Oxford, South Parks Road, Oxford, OX1 3PS UK

**Keywords:** Ribosomal protein universal tree, Archaebacteria, Eubacterial phylogeny, Rooting eukaryote trees, Eukaryogenesis

## Abstract

Palaeontologically, eubacteria are > 3× older than neomura (eukaryotes, archaebacteria). Cell biology contrasts ancestral eubacterial murein peptidoglycan walls and derived neomuran N-linked glycoprotein coats/walls. Misinterpreting long stems connecting clade neomura to eubacteria on ribosomal sequence trees (plus misinterpreted protein paralogue trees) obscured this historical pattern. Universal multiprotein ribosomal protein (RP) trees, more accurate than rRNA trees, are taxonomically undersampled. To reduce contradictions with genically richer eukaryote trees and improve eubacterial phylogeny, we constructed site-heterogeneous and maximum-likelihood universal three-domain, two-domain, and single-domain trees for 143 eukaryotes (branching now congruent with 187-protein trees), 60 archaebacteria, and 151 taxonomically representative eubacteria, using 51 and 26 RPs. Site-heterogeneous trees greatly improve eubacterial phylogeny and higher classification, e.g. showing gracilicute monophyly, that many ‘rDNA-phyla’ belong in Proteobacteria, and reveal robust new phyla Synthermota and Aquithermota. Monoderm Posibacteria and Mollicutes (two separate wall losses) are both polyphyletic: multiple outer membrane losses in Endobacteria occurred separately from Actinobacteria; neither phylum is related to Chloroflexi, the most divergent prokaryotes, which originated photosynthesis (new model proposed). RP trees support an eozoan root for eukaryotes and are consistent with archaebacteria being their sisters and rooted between Filarchaeota (=Proteoarchaeota, including ‘Asgardia’) and Euryarchaeota sensu-lato (including ultrasimplified ‘DPANN’ whose long branches often distort trees). Two-domain trees group eukaryotes within Planctobacteria, and archaebacteria with Planctobacteria/Sphingobacteria. Integrated molecular/palaeontological evidence favours negibacterial ancestors for neomura and all life. Unique presence of key pre-neomuran characters favours Planctobacteria only as ancestral to neomura, which apparently arose by coevolutionary repercussions (explained here in detail, including RP replacement) of simultaneous outer membrane and murein loss. Planctobacterial C-1 methanotrophic enzymes are likely ancestral to archaebacterial methanogenesis and β-propeller-α-solenoid proteins to eukaryotic vesicle coats, nuclear-pore-complexes, and intraciliary transport. Planctobacterial chaperone-independent 4/5-protofilament microtubules and MamK actin-ancestors prepared for eukaryote intracellular motility, mitosis, cytokinesis, and phagocytosis. We refute numerous wrong ideas about the universal tree.

**Electronic supplementary material:**

The online version of this article (10.1007/s00709-019-01442-7) contains supplementary material, which is available to authorized users.

## Introduction 1: the eubacteria-neomura dichotomy in cell structure

Use of ribosomal RNA sequences for phylogeny led to recognition of the important distinction between archaebacteria and eubacteria (Fox et al. 1980). It soon became clear that archaebacteria are more closely related to eukaryotes than to eubacteria and that archaebacteria plus eukaryotes constitute a clade characterised ancestrally by surface N-linked glycoproteins. The archaebacteria/eukaryote clade was called neomura, meaning new walls (Cavalier-Smith 1987c), to contrast it with eubacteria that typically have walls of murein peptidoglycan (mycoplasmas that secondarily lost murein the sole exception) instead of N-linked glycoproteins. From the outset, it was controversial whether archaebacteria are ancestral to eukaryotes (Van Valen and Maiorana 1980; Williams et al. 2013) or are their sisters (Cavalier-Smith 1987c, 2002a), still not unambiguously decided (Cavalier-Smith 2014).

**Fig. 1:**
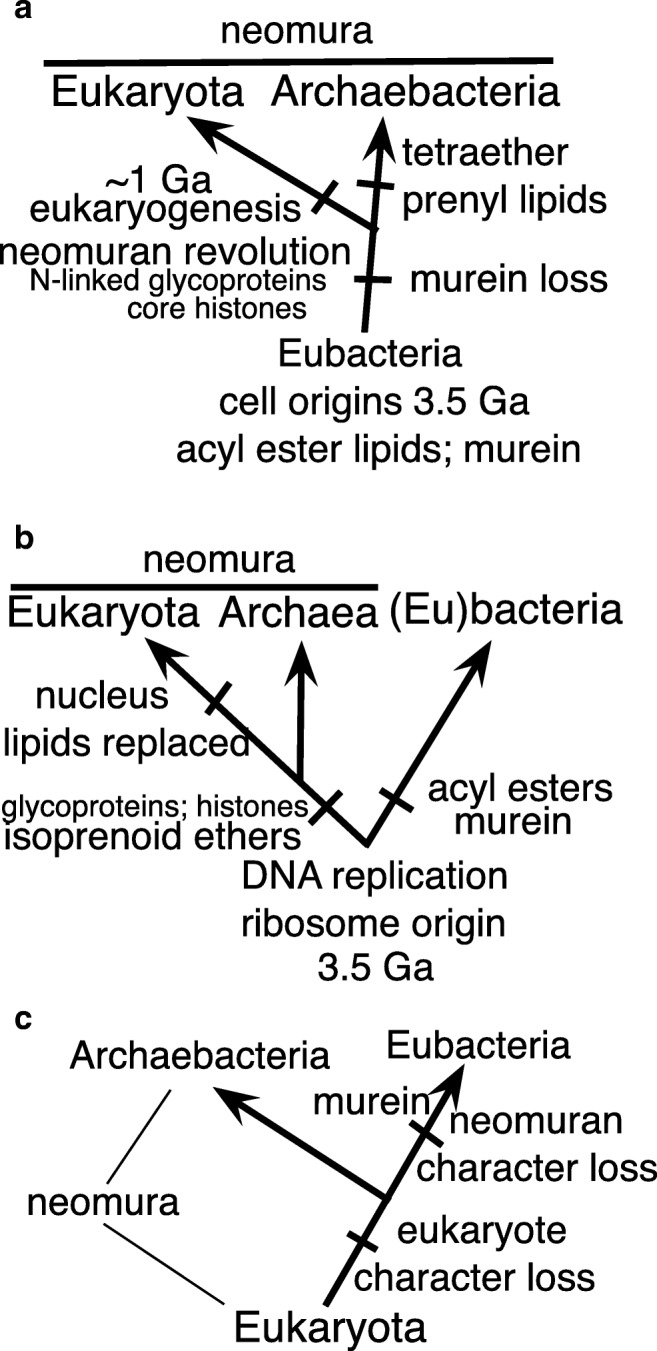
Longstanding contradictory interpretations of the universal rRNA tree. On the ‘eubacteria-first’ view (**a**), eubacteria are the ancestral domain, several times older than neomura which arose by the neomuran revolution (Cavalier-Smith 1987c, 2002a), a radical cell transformation caused by loss of murein peptidoglycan by a eubacterium similarly to the origins of mycoplasmas and L-forms from Bacillia. **a** is strongly supported by the fossil record, which indicates that neomura are 3–4 times younger (originating between 0.8 and 1.45 Ga, depending on controversial identification of fossils in this period as ‘stem eukaryotes’ or ‘unusually complex bacteria’: Cavalier-Smith 2006a). Associated changes in cell biology were explained in detail (Cavalier-Smith 2014) on the assumption that the eubacterial ancestor of neomura was a posibacterium (Lake et al. 2009; Valas and Bourne 2011), whereas new evidence presented here favours the more recent idea that it was a planctobacterium (Reynaud and Devos 2011). It argues that long stems at the base of neomura and eukaryotes on rDNA and RP trees result from episodic hyperacceleration of ribosome evolution caused by origins of cotranslational secretion of glycoproteins and the nucleus respectively (Cavalier-Smith 2002a). The ‘archaea ancient’ view (**b**) assumes that neomura are as old as eubacteria and that neomuran and eubacterial characters evolved divergently immediately after the origin of life, often assuming that their membranes arose independently by simultaneous separate origins of acyl ester lipids in eubacterial ancestors and isoprenoid ethers in ancestral neomura (this ancient ‘lipid divide’ is now refuted by eubacterial prenyl ether lipids, and archaebacterial fatty acids). **b** is based on (1) highly dubious a priori ideas about archaebacteria (Woese and Fox 1977a, b); (2) the false assumption that rDNA nucleotide substitution rates have been largely unchanged since cells began; and (3) uncritical interpretation of the first protein paralogue trees that ignored the likelihood that they also are temporally distorted by episodic hyperacceleration causing long-branch artefacts that misroot the three-domain tree in the stretched neomuran stem (Cavalier-Smith 2002a, 2006c). **b** imagined that eukaryotes replaced isoprenoid ethers by α-proteobacterial acyl esters during mitochondrial enslavement (Martin 1999). Variants of **a** and **b** exist that assume that archaebacteria are ancestral to, not sisters of, eukaryotes (Williams et al. 2013), but also accept neomura as a clade. In contrast, the prokaryotes-late or eukaryotes-first (Mariscal and Doolittle 2015) view (**c**) assumes cells were originally eukaryote-like and prokaryotes arose by radical simplification (‘streamlining’: Forterre 1995) but never explicitly attempted to explain how; Forterre (2013) now prefers **b**. Proponents of **b** and **c** ignore the fossil record that refutes both, and largely ignore cell biology, failing to explain how assumed cell transformations could have occurred (incredible for **c**; highly implausible selectively and mechanistically for **b**—yet **b** may still be the most widespread assumption despite its serious defects; many remain unaware that paralogue pairs more often favour a eubacterial root, like fossils). Only **a** offers a scientifically explicit hypothesis as to the cell structure of LUCA

The cladistic relationship between eubacteria and neomura has been even more controversial, with three contrasting views (Fig. 1): (a) eubacteria are ancestral to neomura, which are therefore younger (Cavalier-Smith 1987b, c, 2002a, 2014; Lake et al. 2009; Valas and Bourne 2011); (b) they are sisters and thus of roughly equal age, with the root of the universal tree lying between them (Gogarten et al. 1989; Iwabe et al. 1989); (c) neomura, specifically eukaryote-like cells, are ancestral to eubacteria, with the universal root lying within the eukaryote stem or crown and prokaryotes having arisen by secondary simplification (so called streamlining) (Forterre 1995); Mariscal and Doolittle (2015) lumped 10 disparate speculations as ‘eukaryote-first’, but all are extremely vague as to the overall *cellular* properties possessed by the last ‘universal’ common ancestor of all life (LUCA), none explicit enough to be worthwhile scientific hypotheses about LUCA, and none truly eukaryote-first (i.e. none positing that LUCA had a nucleus, mitosis, meiosis, syngamy, ER-Golgi differentiated endomembrane system, and cilia or mitochondria, a logical impossibility!) as they mostly refer only to relatively trivial mainly genomic molecular details and ignore most cell biology; calling them ‘eukaryote-first’ is conceptually misleading. Saying ‘eukaryote-first does not mean Eukarya first’ was obscurantist. Unless we can confidently decide between these three roots, we cannot accurately reconstruct the nature of LUCA and determine the direction of evolution at key transitions.

Sequence trees alone did not give a generally accepted answer (Gouy et al. 2015; Philippe and Forterre 1999). Though many mistakenly think paralogue rooting tells us that Fig. 1b is correct, that topology was only true of the first two such papers (Gogarten et al. 1989; Iwabe et al. 1989). A majority of later paralogue trees placed the root within eubacteria (Cavalier-Smith 2006c; Zhaxybayeva et al. 2005) in accord with Fig. 1a. Cavalier-Smith (2002a, 2006c) argued that this eubacterial root is probably correct and that paralogue trees suggesting otherwise are misrooted because of severe long-branch attraction artefacts resulting from transient ultrafast evolution in neomuran stem lineages. Apparently none favour a eukaryote root (Fig. 1c), so most reject this possibility and accept that neomura are a clade, though few know its name. This conflict between different paralogue trees over root 1a and 1b, irrespective of its causes, means that evidence from other sources than sequence trees is indispensible to allow their correct interpretation (Cavalier-Smith 2006c). Sequence evidence from indels puts the root in eubacteria (Lake et al. 2009; Valas and Bourne 2011). So also does evidence from the fossil record that crown eubacteria are 3.5 Ga whereas eukaryotes are only ~ 1 Ga or even less; mapping their rRNA and ribosomal protein trees onto well-dated palaeontological evidence (fossils, biomarkers, and the date of atmospheric oxygenation) strongly argues that the root is within eubacteria, relative dates being incompatible with a root in the interconnecting stem between neomura and eubacteria (Cavalier-Smith 2006c). An ingenious rooting argument is that eubacterial amino acid usage bias makes it likely that the genetic code evolved in eubacteria not neomura (Fournier and Gogarten 2010); this analysis does not tell us whether the root is within the eubacterial crown as Cavalier-Smith (2002a, 2006a, c) argued or in the neomuran stem (which the authors assumed but their analysis could not justify), but it argues against it being within neomura, thus against Fig. 1c and all 10 ideas discussed oversympathetically by Mariscal and Doolittle (2015). Two outgroup-free rooting methods applied to the universal rDNA tree gave contradictory results, the one more sensitive to systematic artefacts placed it in the neomuran stem, whereas the more accurate method put it within eubacteria, implying that archaebacteria evolved from and are younger than eubacteria (Williams et al. 2015).

Recent evidence from sterane and other fossils implies that neither archaebacteria nor eukaryotes became abundant before ~ 0.85 Gy ago (Schinteie and Brocks 2017). A lateral gene transfer from chloroplasts to archaebacteria (Petitjean et al. 2012) as explained later in this paper decisively shows that archaebacteria are at least three times younger than eubacteria, so the root must lie within eubacteria (Cavalier-Smith 2002a). Even 30 years ago, it was clear to those familiar with the microbial fossil record that eukaryotes are several times younger than eubacteria and that sequence trees could only be reconciled with the fossil evidence if archaebacteria also are substantially younger than eubacteria (Cavalier-Smith 1987c). At that time, Woese (1987) considered the possibility that the root of the universal tree may lie within eubacteria, but found that idea ‘intuitively unappealing’ yet provided no evidence against it; though he asserted that eubacterial and archaebacterial rDNA evolved at different rates, he misleadingly called rDNA a chronometer, and never discussed fossil evidence for actual dates, from which alone differential rates can be objectively inferred. Chronometer (an exceptionally accurate clock) was an extremely misleading term for a molecule that actually evolved at vastly different rates in different lineages (Cavalier-Smith 2002a; Cavalier-Smith et al. 1996, 2018) and is often more erratic in its rate evolution than many proteins. Woese (1987 p. 262) wrote ‘Since archaebacterial 16S rRNA is closer in sequence to both its eubacterial and eucaryotic counterparts than these two are to one another, the archaebacterial version of the molecule must be closer to the common ancestral version than is one or both of the other versions’. That was illogical as the seemingly intermediate nature of archaebacteria is compatible with all three Fig. 1 root positions, and most simply explained by (a); his drawing of archaebacteria at the base of his tree (his Fig. 4) and earlier progenote ideas (now disproved) and unwarranted belief in the great antiquity of methanogens (Woese and Fox 1977a, b) and exaggeration of the distinctiveness of archaebacteria apparently prevented him considering contrary evidence and arguments. Many others have been similarly uncritical and still believe that eubacteria are a clade, despite compelling evidence that they are the sole ancestral ‘domain’ of life, as explained in detail previously (Cavalier-Smith 2002a, 2006a, c).

This paper focuses instead on (1) internal phylogeny of eubacteria and archaebacteria, (2) problems in inferring from RP trees which eubacteria were ancestral to neomura, (3) where the archaebacterial and eukaryotic roots lie, and (4) whether eukaryotes are sisters of all archaebacteria or branch within them. Though a firmibacterial ancestry for neomura (Valas and Bourne 2011) was seemingly strengthened by discovery that some Bacilli have both eu- and archaebacterial type lipids (Guldan et al. 2011), our new site-heterogeneous RP trees (more taxon-rich than hitherto) strongly contradict a posibacterial origin (Cavalier-Smith 1987c), being more compatible with the increasingly discussed idea that neomura arose from Planctobacteria (Reynaud and Devos 2011). Furthermore, Sphingobacteria (=FCB group), which we show here are sisters of Planctobacteria, have all the basic archaebacterial lipid-making enzymes, which actually make such lipids when introduced into *Escherichia coli*, and Planctobacteria have some of them (Villanueva et al. 2018; Coleman et al. 2019). We therefore critically reassess steadily growing evidence for a planctobacterial origin of neomura, explain why that idea is greatly superior to all its competitors, and correct many previous misinterpretations of the universal tree and cell evolution.

Though mistaken about the tree’s root and archaebacterial antiquity, Woese was probably the first post-sequencing to suggest that the last eubacterial common ancestor was photosynthetic (Fox et al. 1980). Our improved eubacterial phylogeny enables us jointly with other evidence to confirm this and provide a stronger basis than hitherto for LUCA having been a photosynthetic eubacterium similar to Chloroflexi (Cavalier-Smith 2006a, d); we demonstrate that vertical inheritance coupled with numerous losses best accounts for scattered distribution of photosynthesis across the eubacterial tree (Cavalier-Smith 2002a, 2006a, c) and lateral gene transfer (LGT) was less important than some suggest (e.g. Shih et al. 2017; Ward et al. 2018). We conclude that the murein peptidoglycan wall, eubacterial flagella, and negibacterial outer membrane (OM) with porins were also demonstrably present in LUCA and multiply lost, but OM lipopolysaccharide probably originated only after Chloroflexi and other phyla diverged. We demonstrate also a high frequency of losses for respiration and methylotrophy and that (contrary to widespread assumptions) archaebacteria ancestrally inherited aerobic respiration and prenyl diether lipid synthesis from eubacteria. A general conclusion of our synthesis is that multiple losses, evolutionarily easy by independent gene deletions, and secondary simplification have been much more important in prokaryote evolution than commonly assumed, whereas LGT is too often invoked with insufficient phylogenetic evidence or explicitness when vertical inheritance plus losses are a better explanation.

## Introduction 2: negibacterial root of eubacteria

Most eubacterial phyla have a complex envelope with an OM traversed by hollow cylindrical porin channels (and other β-barrel proteins) connected to the cytoplasmic membrane (CM) via bridges through the murein wall. Such bacteria are called negibacteria as most have thin walls and so stain Gram-negatively (Cavalier-Smith 1987b, c, 2006a, c), though a few (e.g. *Deinococcus*) with thicker murein stain Gram-positively. Two groups with thick murein walls stain Gram-positively (Actinobacteria, the high GC Gram +ves; and Clostridiia/Bacilli, the low GC Gram +ves) and were once formally grouped together as division (=phylum) Firmacutes (Gibbons and Murray 1978) (later Firmicutes: Murray 1984) to contrast them with division Mollicutes (mycoplasmas, with neither walls nor OM). Closer grouping of mycoplasmas to Clostridiia/Bacilli than to Actinobacteria or negibacteria made it clear that the absence of the negibacterial OM in mycoplasmas was evolutionarily more fundamental than the absence of murein and likely that mycoplasmas arose degeneratively from Clostridiia/Bacilli by wall loss analogously to the well-known wall-less L-forms. Therefore, all three were grouped as subkingdom Posibacteria (Cavalier-Smith 1987b, c), which was a clade on the first rDNA trees (Fox et al. 1980), and Endobacteria was introduced as a subphylum name for Clostridia/Bacilli plus mycoplasmas on the assumption that their last common ancestor had thick walls and endospores (Cavalier-Smith 1998b). The distinction between negibacteria and posibacteria appeared to be the most evolutionarily important ultrastructural dichotomy within eubacteria, which highlighted a fundamental question about cell membrane evolution. Did posibacteria arise from negibacteria by OM loss (Blobel 1980; Cavalier-Smith 1987c)? Or were posibacteria with just one membrane older and negibacteria evolved from them by OM addition as many have assumed, e.g. Gupta (1998b) when proposing the terms monoderm or diderm for cells with one or two bounding membranes.

Gupta’s argument that eubacteria were ancestrally monoderm stemmed from two incorrect beliefs: (a) the universal tree is rooted between monoderm archaebacteria and the eubacterium *Thermotoga* and (b) *Thermotoga* is monoderm also. Cavalier-Smith for a while accepted *Thermotoga* as monoderm, so wrongly put it in Posibacteria (Cavalier-Smith 1998b, 2002a), but later excluded it after realising its ‘toga’ is an unusual negibacterial OM with OmpA porin homologues that secondarily lost lipopolysaccharide (LPS) (Cavalier-Smith 2006c), which recent analyses support (Antunes et al. 2016; Eveleigh et al. 2013). A key to understanding posibacterial evolution was the discovery of endospore-forming bacteria that stained Gram-negatively, but confusion over whether they had an OM (as does *Selenomonas*) or not (e.g. *Heliobacterium* with an S-layer, not OM) persisted for some years, hampering classification and making the significance of their frequent grouping with Bacilli/Clostridiia on trees ambiguous. It is now clear that two distinct clades of Gram-negative endospore-forming bacteria have genuine negibacterial OMs (Halanaerobiales and ‘Negativicutes’) but are phylogenetically interspersed with several Gram-negative endospore-forming lineages that lack an OM and so are classically posibacterial or monoderm (e.g. Heliobacteriales); sequence trees group both negibacterial clades more closely with the original posibacterial Endobacteria than they do with Actinobacteria (Campbell et al. 2015; Marchandin et al. 2010). ‘Negativicutes’, a now invalid name corresponding with the Selenobacteria originally excluded from Posibacteria because of their OM (Cavalier-Smith 1992b), and Halanaerobiales both have LPS, whose synthesis is vertically inherited in eubacteria; thus, the OM was lost more than once by negibacterial endospore formers to generate posibacterial monoderm phenotypes (Antunes et al. 2016; Poppleton et al. 2017). Our new RP trees confirm this polyphyly of low-GC Gram-positives and also strongly show that Actinobacteria lost the OM independently of Endobacteria. We conclude that ancestral eubacteria were negibacteria with two membranes, and monoderm posibacteria evolved from them by several OM losses, not one loss as first suggested (Cavalier-Smith 1987b, c). The possibility that Actinobacteria were the ancestral state for eubacteria is excluded as indel analysis put the root outside them (Servin et al. 2008).

As posibacteria are not a clade, we abandon phylum Posibacteria and henceforth treat Actinobacteria (ancestrally monoderm, mycobacteria secondarily diderm) and Endobacteria (ancestrally diderm, polyphyletically mostly secondarily monoderm) as separate phyla, but retain subkingdom Posibacteria to embrace both. ‘Endobacteria’ here refers to the clade comprising all descendants of the endospore-forming last common ancestor of Halanaerobiales, Heliobacteriales, ‘Negativicutes’, Clostridiia/Bacilli and mycoplasmas irrespective of whether or not they retain ancestral OM, murein, and endospores. Our new RP trees strongly confirm the monophyly of thus redefined Endobacteria and also show for the first time that mycoplasmas are polyphyletic and arose from Bacilli by two separate murein losses. Currently, nomenclature and classification of clade Endobacteria is confused. Bergey’s Manual and most recent papers (e.g. Ruggiero et al. 2015) do not accept it as a clade but incorrectly treat it as two phyla: Tenericutes with the single class Mollicutes, which are polyphyletic, and ‘Firmicutes’ which our trees robustly show are paraphyletic. Though some papers use this phylogenetically unsound classification, e.g. Segata et al. (2013), others contradictorily extended Firmicutes to include Mollicutes/Tenericutes when labelling clades on eubacterial trees (Battistuzzi et al. 2004; Ciccarelli et al. 2006; Hug et al. 2016). Though the latter makes sense cladistically, that two contradictory meanings of Firmicutes are now in use is confusing, especially as neither corresponds to its original sense or is descriptively meaningful. As Endobacteria refers to the endospore innovation that ancestrally distinguished the clade from all other eubacteria, it is distinctive and semantically appropriate. Adopting Firmicutes (which originally referred to thick skin, i.e. thick murein walls without an OM) for this group that includes thin-murein negibacterial basal members and derived murein-free members, but excludes the descriptively and originally firmicute Actinobacteria, would be descriptively meaningless and conceptually confusing; so as before we avoid the ambiguous term Firmicutes, and recommend that others likewise abandon it.

**Fig. 2:**
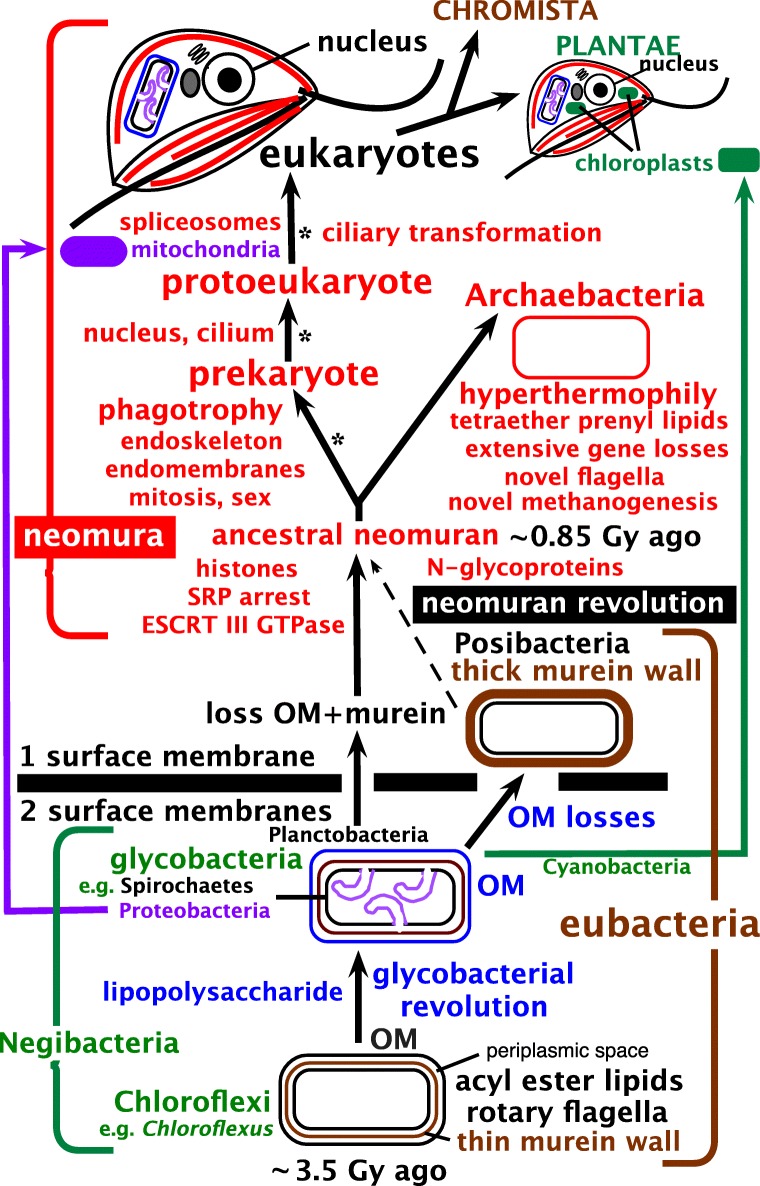
The major kinds of cell and likely evolutionary relationships. Cell envelope and chromosome chemistry divides life into ancestral eubacteria, with murein peptidoglycan walls and DNA negatively supercoiled by DNA gyrase without histones, and derived neomura (probably over three times younger), with N-glycoproteins cotranslationally secreted by more complex SRPs and DNA passively negatively supercoiled by histones (some archaebacteria may retain eubacterial DNA gyrase and reverse gyrase and some lost histones). Eubacteria exhibit three grades of organisation: Chloroflexi (=Chlorobacteria), unusual negibacteria with an outer membrane (OM) of phospholipids but no lipopolysaccharide (LPS); glycobacteria, the majority of negibacteria (11 phyla), whose OM has an outer leaflet of LPS: and monoderm posibacteria whose ancestors lost the OM and comprise a majority of phyla Actinobacteria and Endobacteria. We argue that neomura arose after simultaneous loss of murein and OM by a planctobacterial glycobacterium with primitive microtubules; numerous recent discoveries make the older idea based on OM loss parsimony (Cavalier-Smith 1987c) that they arose from a posibacterium by losing murein only (dashed line) no longer tenable. Eukaryotes kept eubacterial acyl ester lipids but archaebacteria became hyperthermophiles by largely replacing them by stabler prenyl ether lipids (whose biosynthetic enzymes and diether variants probably arose much earlier in glycobacteria). Archaebacteria retained prokaryote cell structure and DNA segregation machinery but not microtubules, whereas eukaryotes evolved phagotrophy that caused evolution of an endomembrane system with coated vesicle budding and targeted vesicle fusion leading to origin of the nucleus, microtubule-based mitosis and consequential radical genetic changes, and enabled intracellular symbiogenesis by enslaving glycobacteria: a chromatophore-bearing α-proteobacterium as mitochondria to make kingdom Protozoa; and later a thylakoid-bearing cyanobacterium as chloroplasts to make kingdom Plantae. Kingdoms Eubacteria and Archaebacteria have non-homologous rotary extracellular flagella; but eukaryotes all descend from an ancestral biciliate protozoan with two immensely more complex microtubule-based intracellular bending cilia that undergo structural transformation once every cell cycle, the younger one losing its juvenile morphology in the second cell cycle. We do not portray the most complex membrane topology of all, found in kingdom Chromista, where chloroplasts, a red algal plasma membrane, and sometimes a relict nucleus, are present inside host ER lumen, having arisen soon after chloroplasts when a biciliate phagotroph enslaved an engulfed red algal symbiont (see Cavalier-Smith 2018). Eukaryogenesis is postulated to have involved three logically distinct stages (asterisks); mitochondria must have preceded spliceosomes and followed the prekaryote phase but might have become symbionts simultaneously with nucleus and cilium origins

Transition analysis excluded the root of the universal tree from neomura and Posibacteria, concluding that its most likely position is between Chloroflexi and all other organisms (Cavalier-Smith 2006c); that paper regarded Chloroflexi as negibacteria, i.e. as having an acyl ester phospholipid bilayer OM evolutionarily distinct from the secondarily derived mycobacterial OM. Unlike almost all other negibacteria, Chloroflexi lack LPS, so Sutcliffe (2011) argued that the outer layer is an S-layer not a membrane. Nobody doubts that LPS is absent in Chloroflexi, but that is not evidence for the absence of an OM of phospholipids, as Sutcliffe incorrectly assumed it to be; Keppen et al. (2018) prematurely assumed that the absence of LPS makes the chloroflexan *Oscillochloris* monoderm, when ultrastructurally it appears to be plausibly diderm with a visible OM. New micrographs of *Pelolinea submarina* convincingly show its outermost layer to be an OM (Imachi et al. 2014 Fig. S1C) with the same trilaminar structure as the CM. Moreover, *Flexilinea* (Sun et al. 2016) and *Thermoflexus* (Dodsworth et al. 2014) outer layers more closely resemble OMs than S-layers; *Nitrolancea* with a thicker envelope appears to have an OM just outside a thin peptidoglycan layer, plus an external thicker capsule that could be related to an S-layer. In the photosynthetic *Chlorobaculum tepidum* cryoelectron tomography, without chemical fixation, sectioning or staining that might distort structure, shows an OM indistinguishable in appearance from the CM (Kudryashev et al. 2014). These better resolved micrographs show that reassigning Chloroflexi to posibacteria (Cavalier-Smith 2014) based on Sutcliffe’s misinterpretation was incorrect. Numerous Chloroflexi porin-homologues are annotated in GenBank (including *Chlorobaculum*) making it likely that most Chloroflexi have a porin-traversed OM of simpler chemistry than most negibacteria. Though Chloroflexi lack the four core LPS biosynthetic genes (Antunes et al. 2016) many others annotated as involved in LPS synthesis are present in GenBank and might be involved in making historical precursors of some LPS components which must have existed before full scale LPS synthesis could have evolved in all its complexity. Therefore, the case for Chloroflexi being the earliest diverging negibacteria prior to LPS origin remains as strong as ever. Figure 2 indicates likely relationships amongst the major kinds of cell that our study aims to test and provide a more robust taxon-richer phylogeny for prokaryotes, especially the extremely diverse and likely ancestral eubacteria. In contrast to the apparent loss of LPS in the thermophilic negibacteria Thermotogales and Caldisericia, and its loss in some spirochaetes, some Hadobacteria, and a few parasitic proteobacteria (none of which has lost the OM, proving several times independently that OMs without LPS exist) (Sutcliffe 2010), LPS absence in Chloroflexi is likely the ancestral state for eubacteria (Cavalier-Smith 2006c).

All three negibacterial groups without LPS have OM porins, so homologous OM porins not LPS are the distinguishing feature of negibacteria, which had a single origin, but gave rise to monoderm posibacteria (by several OM losses) and as we show here independently to neomura. Thus, negibacteria had a monophyletic origin, whereas diderm prokaryotes are polyphyletic having arisen three times: negibacteria soon after the origin of life, the mycobacterial OM with a mycolic acid long after the origin of Actinobacteria, and the wall-less crenarchaeote *Ignicoccus* with an outer cell membrane (OCM) of diether lipids (Jahn et al. 2004; Rachel et al. 2002), which unlike negibacterial OMs is energised and evolved long after archaebacteria. We discuss the independent origins of these non-homologous diderm membranes.

Relationships amongst the above eubacterial groups and internal phylogeny of Negibacteria were not unambiguously answered by rRNA trees as they lacked basal resolution within the dense eubacterial bush with numerous near simultaneously diverging phyla (Woese 1987). rDNA trees were very useful for revealing the major gulf between eubacteria and archaebacteria, leading to the concept of three separate ‘domains’ for them and eukaryotes, and also for establishing preliminary phylogenetic clusters that often came to be called ‘phyla’. At present, there are roughly 30 deep-branching rDNA-defined eubacterial clusters, amongst which most relationships were unclear before our study. Though 29 were provisionally accepted as ‘phyla’ in a recent comprehensive classification of life (Ruggiero et al. 2015), it was noted that this number is highly inflated compared with eukaryote phyla because the widely used rule of thumb rDNA clustering criterion for phylum rank often does not indicate great morphological disparity in body plan amongst clusters as do eukaryote phyla, but in essence just reflects the weak resolution of rDNA trees for deepest branching patterns (as noted earlier in relation to deep-branching clades known only from environmental DNA sequencing (Cavalier-Smith 2002a)).

Multiprotein ribosomal protein (RP) trees now offer markedly higher resolution for prokaryote phylogeny than rDNA (Lasek-Nesselquist and Gogarten 2013; Raymann et al. 2015) giving a chance to resolve some of these issues, especially if evolutionarily more realistic and accurate, site-heterogeneous algorithms are used instead of site-homogeneous ones largely used for rRNA trees, which are more prone to long-branch attraction (LBA) artefacts (Lartillot et al. 2007). Previous site-heterogeneous analyses were taxonomically too undersampled for eubacteria to answer these questions: the broadest (Raymann et al. 2015), with 67 eubacteria, included only 13 of the 29 ‘phyla’; Lasek-Nesselquist and Gogarten (2013), with 42 eubacteria, included only 18. The 151 eubacteria included here represent all 29, plus other more recently recognised lineages, which we now conclude are better reduced to 14 robust phyla by merging clearly related similar groups—a twofold simplification of eubacterial diversity.

## Introduction 3: outstanding key problems in archaebacterial cell evolution

Archaebacteria have many fewer deep divergences than eubacteria or eukaryotes and are divisible into just two (probably sister) clades: Euryarchaeota (most methanogens and halophiles) and Filarchaeota, best ranked as phyla (Ruggiero et al. 2015) or subphyla (Cavalier-Smith 2014). Filarchaeota originally comprised classes ‘Crenarchaeota’, ‘Thaumarchaea’ (including ‘*Cenarchaeum*’ and ‘*Caldiarchaeum*’), and ‘Korarchaea’ (Cavalier-Smith 2014) and should now also include the more recently discovered Asgard archaebacteria (Zaremba-Niedzwiedzka et al. 2017) as a fourth class (here informally called Asgardia) as they all share the group-defining eukaryote-like ESCRT III proteins and actin, absent in most Euryarchaeota. RP trees suggested that the archaebacterial root may lie within euryarchaeotes (Lasek-Nesselquist and Gogarten 2013; Petitjean et al. 2015; Raymann et al. 2015) but trees for a set of 38 longer, more conserved proteins place the root instead between Euryarchaeota and Filarchaea (Petitjean et al. 2015), the most likely position on cell evolutionary grounds (Cavalier-Smith 2014). However, these trees did not include any Asgardia and also excluded a group of lineages of simplified and genomically reduced ultrasmall archaebacteria with extra-long branches on trees that sometimes form a clade distinct from both euryarchaeotes and filarchaeotes, called DPANN (i.e. ‘Diapherotrites’, acidophilic ‘*Parvarchaeum*’ and ‘*Micrarchaeum*’, ‘Aenigmarchaeota’, ‘Nanoarchaeota’, and halophilic ‘Nanohaloarchaea’ (Rinke et al. 2013)).

‘Nanohaloarchaea’ are strongly sisters of Halobacteriales on the rDNA tree in the absence of other long-branch DPANNs (Narasingarao et al. 2012) so were put in phylum Euryarchaeota by Ruggiero et al. (2015). A concatenation of 38 conserved genes with 32 RPs strongly confirmed that and showed that ‘Nanoarchaeum’ and ‘Parvarchaeota’ did not group with ‘Nanohaloarchaea’, but both were separately within euryarchaeotes (Petitjean et al. 2015). That strongly indicates that a DPANN grouping is in part an LBA artefact and that ‘Nanoarchaeum’ is not the earliest branching archaebacterium as sometimes claimed. When ‘Nanohaloarchaea’ and the other longest branching DPANNs were removed, the remaining DPANN strongly grouped as one clade within euryarchaeotes as the second deepest branch (distinct from Halobacteriales) in a 45-protein analysis including some RPs (Williams et al. 2017). Though their trees strongly argued against DPANN being a clade distinct from Euryarchaeota, Williams et al. (2017) presented evidence from a questionable analysis of gene losses and gains by LGT (which could have been confounded by convergent massive gene loss by DPANN lineages) that the archaebacterial root lies between DPANN and all other archaebacteria, contradicting their earlier outgroup-independent rooting between Filarchaeota and Euryarchaeota/DPANN (Williams et al. 2015). To clarify these controversies, we included representatives of all major DPANN lineages in our 60-taxon archaebacterial RP analyses (selectively favouring those with shortest branches to reduce LBA) as well as lokiarchaeotes to represent Asgardia. None of our trees placed the root within non-DPANN euryarchaeotes as did Raymann et al. (2015) or within Filarchaeota, but both the position of DPANNs which appeared as one or more often two clades and of the root were sensitive to taxon sampling and method, the root often seeming within or beside DPANNs; we think this is a long-branch artefact and favour a root between Euryarchaeota/DPANN and filarchaeotes as in rDNA trees of Williams et al. (2015) and the 70-protein trees of Petitjean et al. (2015).

## Introduction 4: long inter-domain stems magnify problems of rooting RP subtrees

It is well known that establishing the root position of a tree by outgroup rooting can be much more difficult than determining the group’s internal branch topology. Rooting is especially difficult when outgroup branches are very long and differ greatly in sequence from ingroups. For universal rRNA trees, the stem at the base of crown eukaryotes is much longer than the entire crown depth and the stem at the base of neomura is much longer than the depth of either the archaebacterial or eukaryote crown radiations. These two hugely stretched stems arise because of temporary, episodic hyperacceleration of nucleotide substitution rates just before archaebacteria and eukaryotes diversified (Cavalier-Smith 2002a). Their immense length made it very easy to divide organisms cleanly into three domains but make determining the position of the root of eukaryotes, archaebacteria, and neomura extremely difficult, both because the original information relating to the root position has been multiply overlain by repeated substitutions and because of long-branch artefacts. Therefore, it proved impossible to determine reliably the position of any of these three root positions using site-homogeneous 16s/18S rDNA trees (Cavalier-Smith 2002a). Even with combined large and small subunit rDNA sequences and improved site-heterogeneous methods, the apparent positions of the eukaryote and archaebacterial roots on three-domain trees are so contradictory amongst methods and taxon samples (e.g. Foster et al. 2009; Williams et al. 2012) that none to date is credible. All are contradicted for both the eukaryotic and archaebacterial roots by RP trees (Lasek-Nesselquist and Gogarten 2013; Petitjean et al. 2015; Raymann et al. 2015). RP trees also have extremely stretched eukaryote and neomuran stems (Lasek-Nesselquist and Gogarten 2013), which Petitjean et al. (2015) rightly attribute to temporarily hugely accelerated amino acid substitution—they noted that neomuran stem acceleration was greater than for the 38 more conserved proteins proving that RPs cannot be a uniform ‘molecular chronometer’. These long stems show that all components of the ribosome underwent coevolutionary ultrarapid evolution during the origin of the cell nucleus and of the novel neomuran RPs, probably for reasons previously partially explained (Cavalier-Smith 2002a) which include coevolution with the novel features of the ribosome-associated neomuran signal-recognition particle (SRP) which underwent more radical changes during the origin of neomura (the neomuran revolution: Cavalier-Smith 2014) than at any other time since the first cells evolved.

This episodic ribosomal evolution during the neomuran revolution and eukaryogenesis grossly exaggerates the duration of eukaryote and neomuran stem evolution relative to crown evolution if one were to erroneously apply a single molecular clock to any universal ribosomal tree; another example of a highly inflated stem at the base of a clade on multigene trees concerns Foraminifera, whose fossil record is so extensive that one can prove that the stem is in fact grossly inflated compared with the crown as Cavalier-Smith et al. (2018) explained in detail. Because the fossil record is so much less good for archaebacteria and stem eukaryotes, the inflation of their ribosomal tree stems had to be inferred by more indirect correlation between trees and fossil evidence and so is not yet appreciated by all. However, though rDNA and RPs clearly coevolved, their relative tempo was not the same during these two evolutionary episodes: for rDNA, the eukaryote stem is much longer than the neomuran stem, whereas for RPs, the reverse is true. Thus, RPs were relatively more affected than rRNA during the neomuran revolution, presumably because that involved the greatest change in RP composition in the history of life.

The neomuran stem on the (incorrectly rooted) RP tree of Lasek-Nesselquist and Gogarten (2013) represents an average of 5.4 amino acid substitutions per site. Most RP sites must have been overwritten many times since archaebacteria diverged from eubacteria, so it is not credible that enough sites could have persisted unchanged in neomura since that epoch to allow consistent determination by RP trees where within the roughly 30 deep branching eubacterial clades neomura actually arose. That probably explains why the apparent eubacterial origin point for neomura is completely different in all three previous site-heterogeneous RP analyses (Lasek-Nesselquist and Gogarten 2013; Petitjean et al. 2015; Raymann et al. 2015) and also different from earlier rDNA analyses. Here, we run separate one-domain, two-domain, and three-domain RP trees in order to disentangle the logically distinct problems of the internal phylogeny of each domain (for which we show RP trees provide highly credible solutions) from those of rooting each domain and placing it accurately relative to ancestral domains, for which the highly stretched internal stems make RPs very bad phylogenetic markers. We conclude that widespread underappreciation of this problem has led to an exaggerated trust in the overall conclusions possible from three-domain universal ribosomal molecular trees, which we show suffer from more distortion than do two-domain trees.

## Introduction 5: Need for more accurate, critically interpreted taxon-rich RP trees

A taxonomically rich maximum likelihood (ML) three-domain tree for 16 RPs from 3,083 taxa using 2596 amino acids heralded as ‘a new view of the tree of life’ (Hug et al. 2016) illustrates the serious pitfalls of massive automated site-homogeneous trees if we examine its branching order within eukaryotes, whose phylogeny is much better established than for prokaryotes by multiple lines of evidence. Though many younger clades are reasonable, problems are greatest amongst the deepest branches. Nine examples: (1) the apusomonad protozoan *Thecomonas trahens* appears with 100% support as sister to the apicomplexan *Toxoplasma gondii* within the alveolate Chromista—completely different kingdom (they are actually as distantly related as humans and grass)—with three lower strongly supported nodes that are all false. (2) The apusomonad *Manchomonas bermudensis* groups with another apicomplexan *Theileria annulata* with 98% to form a false clade that appears wrongly as sister to glaucophytes (kingdom Plantae) that is ‘sister’ to another multiply false clade comprising Rhodophyta (Plantae) into which are intruded three unrelated lineages from kingdom Protozoa. (3) Alveolates are not a clade, not only for these reasons but also because ciliates are completely misplaced within a cluster of Amoebozoa that belong to a different kingdom. (4) Opisthokonts that are easily robustly found to be monophyletic on all good multigene trees and on many single-gene trees are not a clade, as *Nuclearia* groups in a false deep clade with a metamonad and an amoebozoan (none of these three group with their true relatives)—we ignore the fact that one ‘arthropod’ groups within flowering plants which must be a mix up! (5) Rhizaria do not group with alveolates plus heterokonts as they do on every good multiptrotein tree. (6) Haptophytes which on any good single-gene or multiprotein tree form a robust clade appear polyphyletic. (7) Amoebozoa wrongly appear polyphyletic as do other well-established clades. (8) The parasite *Giardia* is shown as the deepest branching eukaryote and is nowhere near it real metamonad relative *Trimastix* and two nodes away from its true sister *Trichomonas* (both should be much higher in the tree). (9) The second deepest branch is the cryptomonad nucleomorph which is an enslaved red algal nucleus that should have grouped with rhodophytes. In fact, the branching order of all nine deepest branching ‘clades’ within the eukaryote domain are meaningless and false; many are false clades. These profound errors probably mainly reflect LBA, which likewise long ago wrongly put *Giardia*, *Trichomonas*, and other long branches like Microsporidia at the base of eukaryotes on site-homogeneous three-domain trees, thereby grossly misleading our understanding of eukaryote early evolution (Cavalier-Smith 2002a). This 16-protein tree is even more profoundly misleading than was rDNA and beautifully exemplifies the criticism made by Gouy et al. (2015) that studies of relationships amongst the three domains and of the overall root of the tree typically accept much lower phylogenetic technical standards than are de rigeur for eukaryotes and that several questions widely assumed to be settled are not.

If the 16-RP basal branching order is completely wrong for eukaryotes in nine serious ways, it may also be completely wrong for archaebacteria and eubacteria, but because most biologists know no way of cross-checking prokaryote phylogeny other than sequence trees and tend uncritically to accept their results they would be harder to recognise. However, we must not reject RP trees altogether just because some have given ridiculous results. Our present study of 26- and 51-protein RP trees shows that one can with a carefully curated data set from 354 taxa obtain three-domain RP trees without any of the problems just enumerated in the eukaryote subtree. Our results are congruent for eukaryotes with the best independent evidence, but imply that most of the deep branches within that 16-RP tree for archaebacteria and eubacteria (Hug et al. 2016) are indeed false and totally misleading. We therefore present a genuinely new view of the tree of life with potentially more reliable conclusions.

If eubacteria are the only primary domain of life and neomura are their much more recent descendants, as palaeontology and indel and transition analysis all suggest, then it is important to have a more comprehensive robust eubacterial phylogeny to better understand life’s early evolution. We therefore assembled RP sequence data for all 29 eubacterial ‘phyla’ recognised by Ruggiero et al. (2015) to enable new site-heterogeneous phylogenetic analyses, wherever possible including several or at least two phylogenetically widely distinct representatives of each. We also included a 30th ‘phylum’ (Melainabacteria: Di Rienzi et al. 2013) discovered since Ruggiero et al. (2015). Our trees including 151 eubacteria allow us to conclude that no more than 14 (perhaps only 13) genuine phyla (each robustly supported by both site-heterogeneous and site-homogeneous methods) are needed to encompass the presently known phylogenetic diversity of eubacteria. Our 26-protein trees for the first time establish a robust phylogeny amongst most of them, greatly clarifying these and other phylogenetic questions, and highlight key remaining issues.

Another limitation of earlier three-domain site-heterogeneous RP trees is that they were weakly sampled for eukaryotes (18 species in Raymann et al. (2015), 35 in Lasek-Nesselquist and Gogarten (2013)) and excluded most protozoan phyla and poorly sampled all five eukaryotic kingdoms; they were also mutually contradictory with respect to the root position and internal phylogeny of eukaryotes—though they were greatly superior to the 16-RP ML tree (with far more taxa) criticised above. The two-domain neomura-only and three-domain trees of Raymann et al. (2015) were also mutually contradictory. Other site-heterogeneous three-domain multiprotein trees (predominantly including RPs but not restricted to them) included still fewer eukaryotes (10) and yielded strongly contradictory eukaryotic phylogenies (Williams and Embley 2014; Williams et al. 2012, 2013), most clearly wrong in comparison with taxonomically far richer (109–171 taxa) eukaryote multiprotein trees based on 187 conserved proteins (Cavalier-Smith et al. 2014, 2015a, b) and there were similar contradictions in eukaryote phylogeny and root between three-domain and neomuran trees (Williams et al. 2012). If these RP trees are clearly wrong for eukaryotes, how reliable are they for prokaryotes? As much experience indicates that taxonomically rich trees are more reliable than sparse ones, we decided to compare taxonomically rich eukaryote RP trees with the now mostly robustly resolved 187-gene trees (Cavalier-Smith et al. 2015a, 2018). To facilitate exact comparison this study focuses on the 51 RPs from our 187-protein alignments that are shared with archaebacteria. We constructed separate 51-protein trees for 143 eukaryotes representing all major lineages, 60 archaebacteria, and 203 neomura in order to determine whether or not inclusion of distant outgroups distorts two-domain trees. We also constructed 26-RP trees for all three groups as well as 26-RP three-domain trees to allow critical comparison between one-, two-, and three-domain trees. We constructed site-homogeneous and site-heterogeneous trees using 26 and 51 proteins for all three two-domain combinations as well as for three domains and for archaebacteria or eukaryotes only plus 26-protein trees for eubacteria. Though we found that 51-RP site-heterogeneous trees are slightly less good for eukaryotes than 187-protein trees and 26-RP trees a little less good, both taxon rich RP trees were much more congruent with 187-protein eukaryote trees than were published more sparsely sampled RP trees, which confirms that richly sampled site-heterogeneous RP trees can be relatively reliable—though site homogeneous maximum likelihood (ML) trees were more discordant. To better understand the strengths and limitations of RP trees, we compare the largely congruent, but partially conflicting, results of all these trees.

We discuss how our results clarify distortions of single-domain RP trees by foreign domain outgroups and the strengths and limitations of RPs for reconstructing the universal tree of life, and interpret results in the light of other evidence for rooting the entire tree and each domain. Our taxon-rich RP trees improve eubacterial internal phylogeny substantially, but we did not expect them to resolve the exact ancestry of neomura, though hoped more thorough eubacterial sampling would better define the limitations of RP for correctly placing neomura within eubacteria. Unsurprisingly, our trees show slightly contradictory positions for neomura within negibacteria, but are most consistent with an origin from Planctobacteria, which several other recent discoveries have favoured (Reynaud and Devos 2011). This agrees with a few previous rDNA trees that excluded faster evolving sites (Brochier and Philippe 2002) or used more accurate site-heterogeneous algorithms (Williams et al. 2012); both contradicted earlier site-homogeneous rDNA trees that grouped neomura with hyperthermophilic *Thermotoga* and/or *Aquifex* that was reasonably attributed to a long branch artefact; however, these earlier authors overlooked their trees’ evidence for a neomuran relationship with Planctobacteria as they incorrectly rooted them in the neomuran stem (explained: Cavalier-Smith 2006c).

Though sharing of phosphatidylinositol and proteasomes by actinobacteria and eukaryotes earlier favoured posibacterial actinobacteria as the closest eubacterial relatives of neomura (Cavalier-Smith 1987c; 2006c), discovery in posibacterial *Bacillus* of isoprenoid ether lipids with the same *sn*-glycerol-1-phosphate stereochemistry as in archaebacteria (Guldan et al. 2011) seemed to favour endoposibacteria (i.e. monoderm Endobacteria) instead as the sisters or ancestors of neomura, which is also more consistent with evidence from indels (Lake et al. 2009; Valas and Bourne 2011) and signal recognition particle structure (Cavalier-Smith 2010d). However, enzymes making *sn*-glycerol-1-phosphate were recently discovered to be widespread not only in both actinobacteria and endobacteria, but also in Sphingobacteria and more scattered in some members of the vast majority of negibacterial phyla (Coleman et al. 2019), so no longer specifically favour posibacteria as neomuran ancestors. Our RP trees give no support to the idea that neomura arose from any posibacteria or for posibacterial monophyly (Cavalier-Smith 1987c), and also confirm that endoposibacteria are probably polyphyletic—they must have had a more complex evolutionary history than was previously realised (Yutin and Galperin 2013) and cannot reasonably be placed beside the root of the tree of life as some do (Lake et al. 2009). Instead, RP trees best fit the idea that Planctobacteria (the phylum that embraces Planctomycetes, Chlamydiia, and Verrucomicrobia: Cavalier-Smith 1987b, 2002a) are ancestral to neomura, which implies that the secondarily wall-less intermediate ancestor on neomura created by murein loss simultaneously lost the planctobacterial OM, as we explain. Our trees show it is harder than often supposed to establish the roots of the archaebacterial and eukaryote subtrees, but are consistent with (a) a root for archaebacteria between Filarchaeota and Euryarchaeota, with differential character loss between them and (b) eukaryotes being sister to Archaebacteria rather than Filarchaeota, which better explains numerous character distributions across the three domains, including the origins of archaebacterial and eukaryote N-linked glycoprotein synthesis machinery than previous interpretations (Cavalier-Smith 1987c; Lombard 2016), as we shall explain in a new synthesis of the transitions between the three domains.

The better eubacterial taxon-sampling of our trees reveals that *Thermotoga* and *Aquifex*, whose relationship was previously highly controversial (Eveleigh et al. 2013), are each part of two separate ancient taxon-rich negibacterial thermophilic lineages, older Synthermota and younger Aquithermota, both ranked as phyla, which greatly simplifies eubacterial phylogeny. So also does our clear evidence for the unity of Endobacteria and of a broadened Proteobacteria, despite the marked internal morphological diversity of each. Our improved trees allow us to recognise as few as 14 distinctive and robustly monophyletic eubacterial phyla, rather than the hugely inflated 92 ‘phyla’ in the flawed 16-protein analysis (Hug et al. 2016). Furthermore, our site-heterogeneous trees have strong support for the relative branching order amongst them, except at one weakly supported node. These taxon-rich site-heterogeneous RP trees therefore provide a firmer basis for understanding eubacterial diversification and evolution than previously.

Having strengthened evidence for a planctobacterial origin for neomura, we present a new synthesis for origins of archaebacteria and eukaryotes, which explains better than hitherto how both originated and diverged so radically from each other and their eubacterial ancestors. In so doing, we clarify numerous past confusions and refute many widespread misconceptions about the tree of life. As this necessarily makes the paper very long, readers may first like to read the 26 major conclusions at the end.

## Methods

From previous alignments used for eukaryote 187-protein trees (Cavalier-Smith et al. 2014, 2015a, b, 2016), we selected the 51 RPs shared with archaebacteria from 143 eukaryotes that represent all major taxa except Microsporidia and Ectoreta (both excluded because of their exceptionally long-branches that might confuse trees with distant outgroups) and red algae (excluded because chromists are historically chimaeras of a heterotrophic host and an enslaved red alga some of whose genes might be overlooked and thus included for some chromist taxa instead of host genes causing them artefactually to attract red algae on trees: Cavalier-Smith et al. 2015a). From these RPs, we selected the 26 also shared with eubacteria and then added RPs from 60 archaebacteria and 151 eubacteria to these two core alignments, starting with the prokaryote RPs from Lasek-Nesselquist and Gogarten (2013) to which we added archaebacterial RPs from Eme et al. (2013) and numerous prokaryote RP sequences from GenBank. For archaebacteria, we added sequences representing the full diversity of DPANN taxa and lokiarchaeotes to represent Asgardia (both omitted in previous RP analyses). For eubacteria, we included sequences for all 29 ‘phyla’ recognised in Ruggiero et al. (2015) plus Melainabacteria, the majority not represented by earlier site-heterogeneous multiprotein RP trees. We also included a sample of chloroplast and mitochondrial sequences to enable arguments based of their position to be used for relative dating of some eubacterial branches compared with eukaryotes. Alignment was manual, by eye using MacGDE.

Phylogenetic analysis by maximum likelihood (ML) used RAxML-MPI v.7.2.8 PROTGAMMALGF with four gamma rates and 100 fastbootstraps. Site heterogeneous analyses (abbreviated as CAT) used PhyloBayes-MPI v.1.4e GTR-CAT-C-4 rates, the most accurate method readily available that can cope with so many taxa, and at least two chains. Trees were constructed for each chain plus a consensus tree for both after we removed early trees as burnin; the burnin cutoffs and degree of convergence varied amongst datasets as specified in individual figure legends. ML and CAT trees were constructed for eukaryotes, archaebacteria, and eubacteria, separately, for all three domains, and for all combinations of two domains, i.e. 7 distinct taxon samples. Except for eubacteria-only trees that used only 26 RPs, the other six were run separately for 26 and 51 RPs. Because of extremely long branches of the highly divergent mitochondrial sequences, they were omitted from these analyses, but we ran separate eubacterial analyses including mitochondria giving 28 separate analyses for overall comparison. Trees were run on 256 processors in parallel. ML trees took under 5 days but CAT trees were run for at least 10 days (up to a maximum of 45) until they fully converged or we became convinced that one or two branches were so strongly discordant between chains that they would never fully converge. We also ran PhyloBayes-MPI v.1.4e Poisson-CAT-C-4 rate trees for three-domain and one-domain trees for one RP selection in case this simpler but less accurate algorithm would allow quicker or more complete convergence.

We first consider the single-domain trees, then the two-domain trees, before the three-domain trees. As site-heterogeneous trees are theoretically and largely in practice more accurate, figures will show the CAT-GTR trees with support values for CAT-GTR, CAT-Poisson, and ML plotted on them, and major differences noted in the text. In general (especially for CAT-GTR), there were only a few differences between 51 gene and 26 protein trees for one taxon sample so 51-protein trees are discussed first before noting differences using fewer genes. Except for eubacteria, 26-protein trees are in supplementary material. After discussing individual trees, we evaluate their overall implications for establishing a universal tree of life, and better understanding prokaryote phylogeny and major steps in cell evolution, especially origins of neomura, archaebacteria, and eukaryotes.

Alignments for all 51 RPs and for SMC are in supplementary material, as are treefiles for Figs. 3, 4, 5, 6, 7, 8, 9, and 10 and 12.

**Fig. 3:**
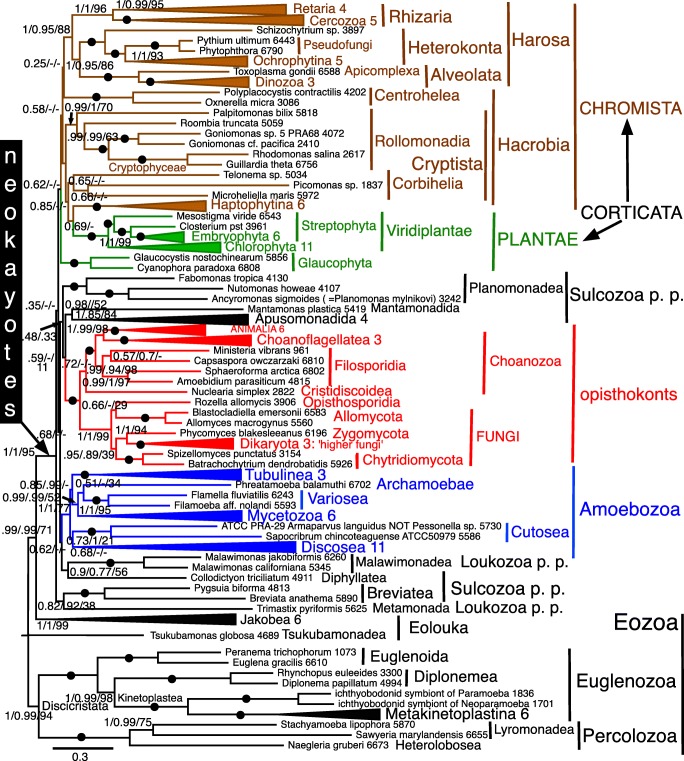
Site-heterogeneous PhyloBayes CAT-GTR tree for 51 ribosomal proteins from 143 eukaryotes representing all the most divergent lineages. Support values for bipartitions are from left to right: posterior probabilities for CAT-GTR, posterior probabilities for CAT-Poisson, RAxML bootstrap percentages for 100 pseudoreplicates; black blobs signify maximal support by all methods in this and all other figures. To fit the page branches for major taxa are collapsed and the number of species included in each given beside their label; their names are shown on uncollapsed trees in Supplementary material, e.g. Fig. S1. The CAT-GTR tree summed 103,304 trees after removing 40% as burn in; both chains converged satisfactorily - maxdiff 0.276977. The CAT-Poisson tree summed 201,391 trees after removing 20% as burn in, but its two chains had slightly different topology (see text)—maxdiff 0.96. The tree is rooted within Eozoa between discicristates and jakobids, but leaving their relative branching order compared with *Tsukubamonas* as an unresolved trifurcation as it is unclear whether *Tsukubamonas* is more closely related to jakobids or to discicristates or the deepest branching lineage (see Cavalier-Smith 2017, 2018). However, it remains controversial whether Eozoa is the basal eukaryote group as shown or whether it is a clade (see text and Fig. 6); in any case, the bifurcation between Eozoa and neokaryotes is the most strongly supported dichotomy on the basal backbone of the RP tree. Compared with Eozoa, whose deep branches are well spread out and fully resolved by all methods, basal branches of neokaryotes form an explosively rapid radiation that is necessarily relatively poorly resolved

**Fig. 4:**
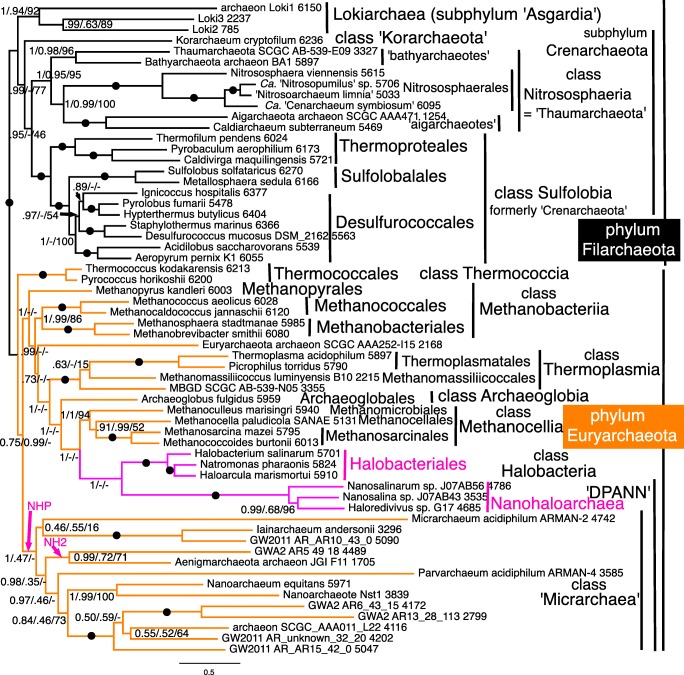
Site-heterogeneous PhyloBayes CAT-GTR tree for 51 ribosomal proteins from 60 archaebacteria representing all the most divergent lineages. Support values for bipartitions are from left to right: posterior probabilities for the CAT-GTR chain 1 analysis (50,437 trees summed after removing 40% of trees as burnin; chain 2 was identical except for rhe position of ‘Nanohaloarchaea’ which were sister to Aenigmarchaeota/GWA2_AR5 in the position shown by arrow NH2 as also on the ML tree), posterior probabilities for the CAT-Poisson (126,435 trees summed after removing 20% as burnin: maxdiff 1), RAxML bootstrap percentages for 100 pseudoreplicates. Arrow NHP shows the contradictory position of Nanohaloarchaea on CAT-Poisson analyses. Asgard archaebacteria are represented only by lokiarchaeotes as other sublineages were unavailable when our analyses began

**Fig. 5:**
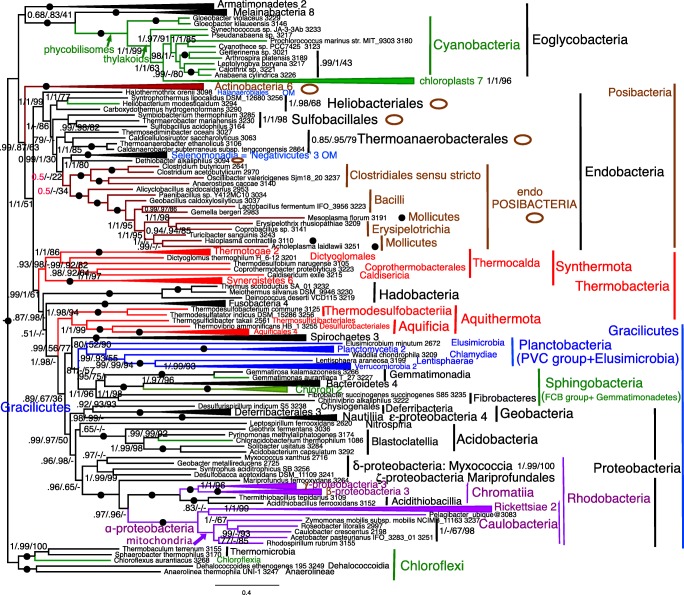
Site-heterogeneous PhyloBayes CAT-GTR tree for 26 ribosomal proteins from 151 eubacteria representing all the most divergent lineages with cultivated representatives plus Melainabacteria and chloroplasts. Support values for bipartitions are from left to right: posterior probabilities for the CAT-GTR, posterior probabilities (PP) for the CAT-Poisson, RAxML bootstrap percentages for 100 pseudoreplicates. To fit on the page branches for some major taxa are collapsed and the numbers of species included for each given beside their label; their names are shown on uncollapsed trees in Supplementary material, e.g. Figs. S9, S10. Despite 70,629 trees being summed after removing the first 30% of them as burnin the two chains did not converge (maxdiff 1) because of two persistent topological differences within Endobacteria at nodes where PP are shown in red. The CAT-Poisson tree did converge (maxdiff 0.328 after we removed 20% as burnin and summed 89,031 trees) on a slightly different topology that also implies five OM losses within Endobacteria; all 14 phyla were clades; branching order of phyla was the same except that Hadobacteria and Fusobacteria were sisters (0.6 support) and Sphingobacteria sisters (0.72) to Spirochaetes not Planctobacteria. The six probably ancestrally monoderm clades are marked by an open brown oval. All others were ancestrally negibacteria with a porin-bearing OM (the two in Endobacteria are labelled OM). Polyphyletic wall-less mollicutes are marked by a black blob beside their names

## Eukaryote ribosomal protein trees

As Fig. 3 shows, CAT topology for 51 RPs is remarkably similar to that with 187-proteins (Cavalier-Smith et al. 2014, 2015a, b, 2016). Most clades are maximally supported by CAT; 69 of these are also maximally supported by ML. Every one of these plus all those additional clades with at least 95% support by both methods was also found on previous 187-protein trees. The least well-supported clades are those at the base of corticates (i.e. Chromista and Plantae, notably affecting the basal branching of Plantae and Hacrobia, neither of which appears as a clade as they should; basal branching within chromist subkingdom Harosa is as robust as with 187 proteins) and at the base of scotokaryotes (primarily affecting the basal branching order within and amongst the protozoan phyla Sulcozoa, Neolouka, and Amoebozoa). Corticata are a weakly supported clade by CAT—but not by ML because of incorrectly intruding sulcozoan planomonads, which 187-protein trees show are deep-branching scotokaryotes (Cavalier-Smith et al. 2014, 2013a, b).

Almost all topological discordances between CAT and ML relate to the deepest branches in corticates (8 contradictions) and Amoebozoa (8 contradictions)—there are only two others: one within Filosporidia in opisthokonts, one within Jakobea in Eozoa. All these contradictions have frequently been noted in multigene eukaryote trees based on over a hundred proteins and stem from their involving numerous extremely closely diverging branches reflecting explosive early radiations. Even for the difficult phylum Amoebozoa, Fig. 3 CAT topology recovers all seven classes as clades as well as subphylum Conosa, exactly as in 187-protein trees (Cavalier-Smith et al. 2016) and even 325-protein trees (Kang et al. 2017). It differs from these only in the insignificantly supported position within Conosa of the archamoeba *Phreatamoeba* and in the weakly supported position of Cutosea relative to Tubulinea and Discosea. The position of Cutosea is slightly uncertain even with 325 proteins and was different for 187 proteins, so for Amoebozoa the 51 RP CAT tree is only slightly less good than with 187 or 325 proteins; discordant branches all have weak support, encouraging caution in interpretation. The ML tree corresponding to Fig. 3 had substantially lower support for many bipartitions and a less accurate topology (not shown), not only with respect to planomonads but also in wrongly placing Cutosea within Discosea making Discosea seem paraphyletic. Our CAT GTR 51-protein tree was markedly superior for Amoebozoa than a tree using the slightly less accurate CAT Poisson algorithm for only seven proteins that wrongly placed Cutosea within Conosa and Tubulinea within Discosea (Panek et al. 2016), though that tree more correctly placed Archamoebae as sisters of Mycetozoa—perhaps because it included eight Archamoebae, not just one.

The main weakness of the 51-protein CAT tree is that it does not resolve the base of Corticata or scotokaryotes accurately, and also shows scotokaryotes as weakly paraphyletic not a clade. However, these branches are relatively much closer and more numerous than in any prokaryote trees discussed below so the good performance of RPs for eukaryotes—if (and perhaps only if, given the discrepancies seen on previous sparser trees) they are taxonomically richly sampled—suggests that similar RP trees for prokaryotes ought to be reasonably reliable provided taxon sampling is sufficiently comprehensive. The corresponding Poisson tree was very similar but differed in some support values and a few branching orders for less well-supported clades. In three respects, Poisson was better (in comparison with the best 147-protein trees) than CAT: the moss *Physcomitrella* and pteridophyte *Selaginella* were correctly successive branches not sisters (0.98); the opisthosporidian protozoan *Rozella* was correctly sister to all Fungi and not weakly sister to Allomycota (0.78 support for exclusion from Fungi); *Nuclearia* was correctly sister to Fungi/opisthosporidia not holozoa. Poisson was worse in Corbihelia being scattered (differently on the two chains) not a clade. Sulcozoan phylogeny differed by Poisson but was not obviously overall better or worse: e.g. the deepest branching neokaryote apparent clade was *Mantamonas*/*Collodictyon* not Breviatea/*Trimastix*, and planomonads were sisters of opisthokonts (swapping position with apusomonads/*Mantamonas*). Within Amoebozoa Cutosea wrongly intruded into Discosea. As with CAT, hacrobian lineages intruded into Plantae near Viridiplantae but the chains were contradictory with respect to the positions of their subclades. Thus, both site-heterogeneous 51-RP trees were good (better than ML) but imperfect in slightly different ways.

However, the 26 protein CAT tree (Fig. S1) is generally somewhat less good: only 61 instead of 69 clades were maximally supported by both methods and support for other well-established clades was usually lower. Unlike in Fig. 3, there was no clear bipartition between corticate and scotokaryote clades as glaucophytes (Plantae) jumped from corticates into scotokaryotes as sister to the insignificantly supported false clade comprising breviates and *Trimastix* on CAT, whereas on ML trees (Fig. S2) glaucophytes were wrongly sister to breviates alone and planomonads wrongly intruded into corticates as with 51 genes. As with 51 genes by ML, Cutosea wrongly intruded into Discosea but with different overall topology. Despite these deficiencies, it is surprising quite how good the 26-gene RP tree is compared with 187-protein tree, as it correctly reconstructed a large majority of those clades that are well supported on trees using over 187 or more proteins and is only seriously defective for those that have been the most difficult of all to establish. In one respect, the CAT-GTR 26-protein tree is better than the 51-protein one: the opisthosporidian *Rozella* is sister to Fungi and does not incorrectly branch within Fungi, though ML still places *Rozella* incorrectly with Chytridiomycetes, making Fungi seem paraphyletic. The 26-protein CAT-GTR tree is clearly wrong only for branches that are also wrong or else rather weakly supported with 51 proteins. So even 26 RP CAT trees should be quite good for prokaryotes—better than ML.

## Archaebacterial ribosomal protein trees

The 51-protein CAT-GTR tree did not converge fully because of an irresolvable contradiction in the position of ‘Nanohaloarchaea’ between the two chains whose individual trees had otherwise identical topology. Chain 1 (Fig. 4) was identical to the two-chain consensus tree in placing them as sister of Halobacteriales with maximal support, as strongly shown by the rDNA tree (Narasingarao et al. 2012) and the 70-protein tree of Petitjean et al. (2015). However, chain 2 discordantly placed ‘Nanohaloarchaea’ with 0.97 support as sister to ‘Aenigmarchaeota’ (not included in the analysis of Petitjean et al. (2015)) within a DPANN clade that branched within Euryarchaeota as a sister to all core euryarchaeotes other than Thermococcales (Fig. S3). Figure 4 and the consensus tree by contrast both show all DPANN other than ‘Nanohaloarchaea’ as a single clade, that we here designate Microarchaea. Clade ‘Microarchaea’ had maximal support on chain 2, where nanoarchaeotes and ‘*Parvarchaeum*’ formed a subclade with 0.97 support that was sister to aenigmarchaeotes with 0.98 support; ‘*Micrarchaeum*’ grouped with ‘*Iainarchaeum*’ with insignificant (0.48) support. The bipartition between phyla Euryarchaeota and Filarchaeota was maximally supported by all methods. Within Filarchaeota, class Nitrososphaeria (=thaumarchaeotes) (always including aigarchaeotes nested within—not a separate group) was strongly supported as sister to Sulfolobia cl. n. by CAT-GTR, weakly by ML; this joint clade was sister to *Candidatus* ‘Korarchaeum’ and Asgardia were strongly supported as the deepest branch, sister to subphylum Crenarchaeota (i.e. ‘Korarchaeum’ plus Sulfolobia/Nitrososphaeria. Subphylum Crenarchaeota Cavalier-Smith 2002 is the correct formal name for what some later unnecessarily called the TACK clade; TACK stands for initial letters of four subclade names of subphylum Crenarchaeota, none nomenclaturally valid. Unreasonable rejection (see Tindall 2014) of class Crenarchaeota Cavalier-Smith 2002 means that this longstanding name can never again be legitimately used for a class, so our Taxonomic Appendix creates replacement name Sulfolobia for the class, but subphylum Crenarchaeota is not rejected and remains legitimate. Throughout the rest of this paper, we therefore use Nitrososphaeria to include all thaumarchaeotes and aigarchaeotes, and Crenarchaeota for the whole subphylum (Fig. 4), not just the invalid class; unavoidable invalid names are usually in quotes or lower case.

The ML 51-protein tree had only four differences, all in euryarchaeotes: (1) ‘Nanohaloarchaea’ moved into Micrarchaea to become sister of aenigmarchaeotes with insignificant (40%) support to form a DPANN clade with moderate (80%) support; (2) ‘*Parvarchaeum*’ moved to sister of ‘*Micrarchaeum*’ with insignificant (44%) support, almost certainly LBA as these are the tree’s two longest branches. Twenty-seven clades had maximal support by both methods; (3) *Methanopyrus* moved up a node to be sister to Methanococcales/Methanobacteriales, making class Methanothermea a clade. Most clades with less than 100% by ML were strongly supported; (4) *Ignicoccus* moved down one node with scarcely significant (50%) support. Only one ML clade unaffected by movement of these four branches was insignificantly supported. Classes Picrophilea and Protoarchaea (Cavalier-Smith 2002a) were maximally supported by both methods; indeed, all five euryarchaeote classes established by Cavalier-Smith (2002a) were distinct clades by ML, with only Methanothermea weakly supported, so it is odd that most papers ignore classes, labelling only the more numerous orders (e.g. Raymann et al. 2015). Clearly, they well reflected euryarchaeote large scale diversity before the discovery of Micrarchaea, which deserve to be made a sixth euryarchaeote class when species are described and it can be validly published by designating types. Even though all five were validly published at the time, they were unfairly rejected recently (Tindall 2014) and even had they not been they would be invalid as incorrectly formed under the new rules—as are all class level names suggested by Petitjean et al. (2015). Figure 4 therefore uses the new replacement class names established in the Taxonomic Appendix in conformity with current rules.

The 51-RP CAT-Poisson tree differed from CAT-GTR primarily in having a DPANN clade that was placed within euryarchaeotes as sister to SCGC AAA251-l15 which moved down four nodes so the joint clade was sister to all euryarchaeotes except Thermococcia. In addition, Lokiarchaea moved up two nodes to be within crenarchaeota as sister to Nitrososphaeria. Interestingly, new class Methanocellia was a strongly supported clade by all three methods, whereas previous site-homogeneous trees had often shown it as paraphyletic ancestors of Halobacteriales (Brochier-Armanet et al. 2011; Petitjean et al. 2015).

The 26-protein CAT tree (Fig. S4) converged well between chains (maxdiff 0.0666) and gave a broadly similar topology to 51-RPs but with often somehat lower support (only 23 clades had maximal support by both methods) and four differences in topology: (1) Thermococcales moved into Methanobacteriia as maximally supported sister to Methanococcales. (2) *Methanomassiliicoccus* moved one node to be sister to MBGD_SCGC_AB-539-N05; this change may be an artefact of low gene representation in these two taxa compared with most others. (3) ‘Nanohaloarchaea’ moved into ‘Micrarchaea’ to become sister of aenigmarchaeotes with strong (0.99) support. (4) The DPANN clade (i.e. ‘Micrarchaea’ plus ‘Nanohaloarchaea’) resulting from (3) was not within euryarchaeotes but separate. The 26-protein ML tree (Fig. S5) differed in three respects: (1) In Filarchaeota, ‘Korarchaeum’ moved up one node to be sister to Sulfolobia; (2) *Ignicoccus* moved down one node as with 51 proteins; (3) methanogens *Methanocella* and *Methanoculleus* interchanged positions.

For large scale phylogeny, the most important difference between 26- and 51-protein trees was the exclusion of DPANN from euryarchaeotes as a single clade rather than two internal clades. Relationships of DPANN lineages to other archaebacteria are controversial. When the tiny symbiotic ‘*Nanoarchaeum*’ was discovered, some took their apparent branching outside euryarchaeotes at face value and considered them primitive archaebacteria or even the most primitive cells, but others argued that their tiny cells and genomes were secondarily reduced and such exclusion a LBA. 50-protein RP ML trees strongly placed it outside shorter-branch euryarchaeotes as did 27-protein large subunit RP trees, whereas 23-protein small subunit trees and 18-protein large subunit trees that excluded nine proteins with discordant single-gene trees placed it within euryarchaeotes as sister to Thermococcales (Brochier et al. 2005). A CAT gamma recoded tree that included also ‘*Parvarchaeum*’ and ‘*Micrarchaeum*’ grouped ‘*Parvarchaeum*’ with ‘*Nanoarchaeum*’ in the same position but ‘*Micrarchaeum*’ was weakly placed just above Methanothermea (Brochier-Armanet et al. 2011). A site-homogeneous Bayesian tree for 32 RPs plus 38 other proteins also put *Nanoarchaeum* with Thermococcales but ‘*Parvarchaeum*’ and ‘*Micrarchaeum*’ as two sister clades to Picrophilea whereas ‘Nanohaloarchaea’ were maximally supported sisters of Halobacteriales (Petitjean et al. 2015): thus, there appeared to be four distinct ‘DPANN’ clades within euryarchaeotes on this 70 protein tree using 10,963 sites (their Fig. 4) that gave no evidence for DPANN being one clade and the same strongly supported position for ‘Nanohaloarchaea’ as in our Fig. 4; the other three ‘Micrarchaea’ clades could have been aggregated into one at the same position as in Fig. 2 by each crossing just one node (all weakly or insignificantly supported). A CAT-GTR tree for 45 archaebacterial proteins including a few RPs but excluding ‘Nanohaloarchaea’ and the longest branch DPANNs had a single maximally supported ‘Micrarchaea’ (Williams et al. 2017) (Fig. S2), with maximal support for it being within Euryarchaeota in the Fig. 2 position (both using all 10738 positions (Fig. S2) and a more stringent selection of 5920 (their Fig. S3)). In another tree including only 25 genes and the 10 most genomically complete DPANN, including *Nanosalina* and ‘*Micrachaeum*’, DPANN was a single clade but within Euryarchaeota with 0.97 support against euryarchaeotes minus DPANNs being a clade. Six further CAT GTR trees each using a separate DPANN subclade placed it within euryarchaeotes (5 with maximal support, one with 0.98 support: 3 in Fig. 4 ‘*Micrarchaeum*’ position, the others all different, only ‘*Nanoarchaeum*’ sister to Thermococcales). A tree for 29 proteins rooted on unspecified eubacterium/a also placed a single DPANN clade within euryarchaeotes as sister to all euryarchaeotes other than Thermococcales. There is therefore consistent support from all previous site-heterogeneous trees and from all cited site-homogeneous ones for all DPANN clades branching within a paraphyletic euryarchaea as shown on Fig. 4. Previous evidence for the position of ‘Nanohaloarchaea’ was more contradictory. Our eubacteria-rooted prokaryote trees (below) more decisively support the conclusion of Petitjean et al. (2015) that ‘Nanohaloarchaea’ are sisters of Halobacteriales, not within ‘Micrarchaea’ as some trees of Williams et al. (2017) implied, but suggest that all other DPANN are a clade, contrary to Petitjean et al. (2015).

## Eubacterial ribosomal protein trees

The 26-protein RP tree (Fig. 5) is taxonomically richer than any other, with 151 species representing all major lineages including many omitted from all previous trees. Both chains converged to exactly the same topology except for one persisting contradiction within Endobacteria, causing Maxdiff to remain at 1. Clostridiia sensu stricto were sisters of Bacilli plus mycoplasmas, making Clostridiales/Bacillia a clade with maximal support on one chain, but in the other chain Clostridiales s. s. moved down two nodes to join Thermoanaerobacterales. In marked contrast to rDNA trees, all bipartitions in the tree backbone were significantly supported except for that separating Hadobacteria and Fusobacteria, which therefore might really be a single clade (as they are with some taxon samples; see below). In a separate tree including also five mitochondrial sequences (Fig. S15), they branched within free-living α-proteobacteria in the position shown by the purple arrow on Fig. 5, not with Rickettsias, suggesting that grouping with Rickettsias on some published trees is a LBA artefact. Except for the position of *Leptospirillum*, delimitation of the proteobacterial subphyla is strongly supported by CAT. Adding mitochondria slightly altered the tree backbone by making Hadobacteria and Fusobacteria insignificantly supported sisters and this joint clade weakly sister to Synthermota, but changed no other relationships between the 14 major phyla (but increased support for Armatimonadetes being sister to Melainabacteria/Cyanobacteria from 0.68 to 0.91 and for *Elusimicrobium* being sister to other Planctobacteria from 0.8 to 0.95).

All 14 phyla were clades (strongly except for Planctobacteria) on the converged CAT-Poisson tree, but two moved slightly in position: Hadobacteria and Fusobacteria were sisters (0.6 support) and Sphingobacteria became sister of Spirochaete not Planctobacteria (unlike most non-Poisson trees). Within Endobacteria, Sulfobacillales were the deepest branch, not Halanaerobiales as in all other trees, and other subgroups rearranged. Within Proteobacteria *Leptospirillum* moved away from Acidobacteria to become sister of Rhodobacteria.

Topology is largely similar by ML (Fig. S6; only 12 differences, only one affecting the backbone: Aquithermota moved to be sister to Thermocalda with insignificant 29% support) but support for less robust branches tends to be lower. Fourteen major deep branching clades that may reasonably be considered phyla are strongly supported by both PhyloBayes site-heterogeneous methods, most maximally (Table 1) of which seven have maximal support by all three methods. Additionally, phyla Melainabacteria and Cyanobacteria are maximally supported as sister clades (here jointly made superphylum Oxybacteria: it is confusing to call Melainabacteria Cyanobacteria: Soo et al. 2017) and the position of chloroplasts within the more advanced cyanobacteria is maximally supported. Table 2 summarises the revised higher eubacterial classification proposed here; its simplicity with only 14 phyla, all phylogenetically sound, is enabled by proper use of intermediate categories (subkingdoms, superphyla, subphyla, infraphyla, superclasses) and greatly superior to the 114 phyla of the indigestible system of Parks et al. (2018), which fails to show relationships between the phyla. Later sections explain the most important of its innovations.

**Table 1:** Support on RP trees for the 14 eubacterial phyla and subphyla

14 phyla	Subphyla	CAT	Poisson	ML
Chloroflexi		1	1	100
	Chloroflexotia	1	1	100
	Dehalococcoidotia	1	1	100
Armatimonadetes		1	1	100
Cyanobacteria		1	1	100
	Gloeobacteria	1	1	100
	Phycobacteria	1	1	99
Melainabacteria		1	1	100
Actinobacteria		1	1	100
Endobacteria (often called Firmicutes)		1	1	99
Synthermota*		0.93	0.98	–
	Synergistetes	1	1	97
	Thermocalda*	1	1	86
Hadobacteria	(*Thermus*/*Deinococcus* group)	1	1	100
Fusobacteria		1	1	100
Aquithermota*	(Aquificia +Thermodesulfobacteriia)	1	0.98	94
Proteobacteria		0.99	0.97	50
	Rhodobacteria	1	0.67	–
	Acidobacteria^a^	0.65	–	–
	Geobacteria	0.98	0.99	–
Spirochaetae		1	1	100
Sphingobacteria (‘FCB group’ + Gemmatimonadetes)		1	1	–
	Fibrobacteres	1	1	98
	Chlorobia*	0.95	0.75	–
	(Chlorobi/Bacteroidetes/Gemmatimonadetes)			
Planctobacteria	(PVC group + Elusimicrobia)	0.80**	0.52	90
	Elusimicrobia		N/A	
	Euplancta (=PVC group)	0.99	0.93	55

Rhodobacteria comprise purple bacteria and their non-photosynthetic descendants, i.e. α-, β/γ-, proteobacteria plus Acidithiobacillia; plus ζ-, and δ- proteobacteria which likely had purple ancestors. Acidobacteria, their sister clade, is here extended to include *Leptospirillum* (class Nitrospiria) which usually groups with class Blastoclatellia (the classical Acidobacteria) on CAT trees

N/A not applicable, as represented on trees by only one species

*New phyla or subphyla names established here

**in Fig. 5; 0.95 in Fig. S15

^a^Acidoacteria excluding *Leptospirillum* (i.e. Blastoclatellia) have 1/1/100% support. Inclusion of Nitrospiria in Acidobacteria is the only significantly weakly supported feature of our phylum demarcation; however on a 49-protein Bayesian tree it has 90% support (Lücker et al. 2013) so its inclusion is justified. Geobacteria, the deepest branching proteobacterial clade, includes ε-proteobacteria and their sister clade Chrysiogenales plus Deferribacterales (neither deserves their common phylum rank)

**Table 2:** Revised higher classification of kingdom Eubacteria* and its four subkingdoms and 14 phyla

**Subkingdom 1. Chlorobacteria** Cavalier-Smith 1992 (as phylum) stat. n. 2019	
**Phylum Chloroflexi** Garrity and Holt 2002 em. Cavalier-Smith	
**Subphylum 1. Chloroflexotia** subphyl. n. Cavalier-Smith. **Description:** the clade including *Chloroflexus* and *Thermomicrobium* but not *Dehalococcoides* or *Ktenobacter*. Type class Chloroflexia.	
Class 1. Chloroflexia Gupta et al. 2013 (e.g. *Chloroflexus*, *Herpetosiphon*, *Roseiflexus*)	
Class 2. Thermomicrobia** Garrity and Holt 2002 (*Thermomicrobium*, *Sphaerobacter*, *Thermobaculum*)	
**Subphylum 2. Dehalococcoidotia** subphyl. n. Cavalier-Smith. **Description:** coccoid or filamentous non-photosynthetic thermophiles more closely related to *Dehalococcoides* (type genus) than to *Caldilinea*.	
Class 1. Dehalococcoidia Löffler et al. 2013 (e.g. *Dehalococcoides*, *Dehalogenimonas*)	
Class 2. Ktedonobacteria Cavaletti et al. 2007 (e.g. *Ktenobacter*)	
**Subphylum 3. Caldilineotia** subphyl. n. Cavalier-Smith. **Description:** filamentous non-photosynthetic thermophiles more closely related to *Caldilinea* (type genus) than to *Dehalococcoides*.	
Class 1. Caldilineia cl. n. Cavalier-Smith. **Description:** The clade consisting of the common ancestor of *Caldilinea* and *Anaerolinea* and all its descendants.	
Subclass 1. Caldilineidae subcl. n. Cavalier-Smith. **Description:** bacteria more closely related to *Caldilinea* than to *Anaerolinea*. Type order Caldilineales Yamada *et al.* 2006 (e.g. *Caldilinea*)	
Subclass 2. Anaerolineidae subcl. n. Cavalier-Smith. **Description:** bacteria more closely related to *Anaerolinea* than to *Caldilinea*. Type order Anaerolineales Yamada et al. 2006	
Class 2. Thermoflexia Dodsworth et al. 2014 em. Cavalier-Smith to include Ardenticatenales Kawachi et al. 2013 as well as Thermoflexales Dodsworth et al. 2014 (e.g. *Thermoflexus*, *Ardenticatena*)	
**Subkingdom 2. Eonegibacteria** Cavalier-Smith 2019 (sister group to Posibacteria plus Neonegibacteria)	
**Phylum Armatimonadetes** Tamaki et al. 2011	
Class 1. Armatimonadia Tamaki et al. 2011 em. (orders Armatimonadetales; Chthonomonadales)	
Class 2. Fimbriimonadia Im et al. 2012 (e.g. *Fimbriomonas*) (*Candidatus* Palusbacteriales of Ward et al. 2019 may belong here too as a third class; likewise ‘*Abditibacterium*’ of Tahon et al. (2018) as a fourth class or order; neither merits treatment as separate phyla)	
**Superphylum Oxybacteria** superphyl. n. Cavalier-Smith. **Description:** Eubacteria descended from the last common ancestor of *Vampirovibrio* and *Prochlorococcus*. **Etymology:***Oxy* (from oxygen) *+* bacteria as they are oxygenic photosynthesisers plus their closest relatives.	
**Phylum 1. ‘Melainabacteria’** Di Rienzi et al. (2013)	
**‘**Class’ 1. ‘Vampirovibrionia’ not validated (e.g. *Vampirovibrio*)	
‘Class’ 2. ‘Sericytochromatia’ invalid (uncultured)	
‘Class’ 3. Saganbacteria + Margulisbacteria (not 2 classes) invalid (Carnevali et al. 2019)	
**Phylum 2. Cyanobacteria Stanier 1974** (subgroup names follow botanical nomenclature rules)	
**Subphylum 1. Gloeobacteria** Cavalier-Smith 2002 (no thylakoids)	
Class Gloeobacteria Cavalier-Smith 2002 (*Gloeobacter, Aphanothece*) Mareš et al. (2013) provide evidence that these genera may not be distinct	
**Subphylum 2. Phycobacteria** Cavalier-Smith 2002 (with thylakoids)	
Class 1. Chroobacteria Cavalier-Smith 2002 (e.g. *Pleurocapsa*, *Oscillatoria*, *Prochlorococcus*)	
Class 2. Hormogoneae Thuret 1875 ex Cavalier-Smith 2002 (e.g. *Nostoc*, *Stigonema*)	
Subkingdom 3. Posibacteria* Cavalier-Smith 1987	
**Phylum 1. Actinobacteria** phyl. n. Margulis 1974 ex Cavalier-Smith. **Description:** ancestrally without OM or endospores; the last common ancestor of *Actinomyces* (type) and *Rubrobacter* and all its descendants.	
Class 1. Actinobacteriia Stackebrandt et al. 1987 spelling corrected and emend. Cavalier-Smith	
Class 2. Acidimicrobia Norris 2013 (*Acidimicrobium*)	
Class 3. Coriobacteriia König 2013 (e.g. *Atopobium, Olsenella*, *Slackia*, *Cryptobacterium*)	
Class 4. Nitriliruptoria Ludwig et al. 2013 (*Nitriliruptor*)	
Class 5. Rubrobacteria Suzuki et al. 2013 emend. Cavalier-Smith by including Thermoleophila Suzuki and Whitman 2013 (e.g. *Rubrobacter*, *Conexibacter*)	
**Phylum 2. Endobacteria** Cavalier-Smith 1998 as subphylum stat. n. (diderm classes D; monoderms M)	
Class 1. Halanaerobiia cl. n. Cavalier-Smith (e.g. *Halanaerobium*) D	
Class 2. Selenomonadia cl. n. Cavalier-Smith (=invalid Negativicutes, e.g. *Veillonella*) D	
Class 3. Clostridiia+ cl. n. Cavalier-Smith (e.g. *Clostridium, Heliobacterium, Sulfobacillus*) M	
Class 4. Bacillia cl. n. Cavalier-Smith M	
Subclass 1. Bacillidae* subcl. n. Cavalier-Smith (e.g. *Bacillus*, *Lactobacilllus*) M	
Subclass 2. Erysipelotrichiidae subcl. n. Cavalier-Smith (e.g. *Turicibacter*, *Mycoplasma*) M	
Subkingdom 4. Neonegibacteria* subk. n. Cavalier-Smith (the negibacterial sister group to Endobacteria)	
Infrakingdom 1. Thermobacteria* infrak. n. Cavalier-Smith	
**Phylum 1. Synthermota** phyl. n. Cavalier-Smith 2019	
Subphylum 1. Synergistetes** subphyl. n. Cavalier-Smith. Sole class Synergistia Jumas-Bilak et al. 2009 (e.g. *Anaerobaculum, Jonquetella, Synergistes*)	
**Subphylum 2. Thermocalda** subphyl. n. Cavalier-Smith 2019	
Class 1. Thermotogia** cl. n. Cavalier-Smith (e.g. *Thermotoga*, *Kosmotoga*, *Thermosipho*)	
Class 2. Dictyoglomia** Patel 2012 (e.g. *Dictyoglomus*)	
Class 3. Caldisericia** Mori *et al.*^2009^ em. Cavalier-Smith (e.g. *Caldisericum*, *Coprothermobacter*, *Thermodesulfobium*)	
**Phylum 2. Hadobacteria** Cavalier-Smith 1992	
Class 1. Deinococcia cl. n. Cavalier-Smith (replacement name for invalid class Deinococci Garrity and Holt 2002 with same type and description). Sole order Deinococcales Rainey et al. 1997 (*Deinococcus*, *Truepara*)	
Class 2. Thermia cl. n. Cavalier-Smith. **Description:** eubacteria more clsoely related to *Thermus* than to *Deinococcus*. Type and sole order Thermales Rainey and Da Costa 2002. (e.g. *Meiothermus*)	
**Phylum 3. Fusobacteria** Garrity and Holt 2012. Sole class Fusobacteriia Staley and Whitman 2012 (e.g. *Leptotrichia, Ilyobacter*)	
**Phylum 4. Aquithermota** phyl. n. Cavalier-Smith 2019 (sister group to infrakingdom Gracilicutes)	
Class 1. Aquificia** cl. n. Cavalier-Smith 2019 (e.g. *Aquifex, Persephonella*, *Hydrogenivirga*)	
Class 2. Thermodesulfobacteriia** cl. n. Cavalier-Smith 2019 (e.g. *Thermodesulfatator*)	
Infrakingdom 2. Gracilicutes* Cavalier-Smith 2006	
**Parvkingdom 1. Proteobacteria** parvk. n. Cavalier-Smith. Description as for phylum Proteobacteria.	
**Phylum 1. Proteobacteria** Stackebrandt et al. 1986 (as class) ex Cavalier-Smith 2002 (as phylum)	
**Subphylum 1. Rhodobacteria** Cavalier-Smith 2002 (purple photosynthetic bacteria and relatives)	
Class 1. Caulobacteria cl. n. Cavalier-Smith (ɑ-proteobacteria e.g. *Caulobacter*, *Rhodospirillum*, *Pelagibacter*)	
Class 2. Chromatiia cl. n. Cavalier-Smith (purple sulphur bacteria and relatives)	
Subclass 1. Acidithiobacillidae (γ-proteobacteria e.g. *Chromatium*, *Acidithiobacillus, Escherichia*)	
Subclass 2. Neisseriidae Cavalier-Smith subcl. n. (β-proteobacteria e.g. *Neisseria*)	
Class 3. Mariprofundia cl. n. Cavalier-Smith (ζ-proteobacteria e.g. *Mariprofundus*)	
Class 4. Myxococcia cl. n. Cavalier-Smith (δ-proteobacteria)	
Subclass 1. Mycococcidae subcl. n. Cavalier-Smith (e.g. *Myxococcus*,)	
Subclass 2. Geobacteridae subcl. n. Cavalier-Smith (e.g. *Geobacter*)	
Subclass 3. Oligoflexidae (*Bdellovibrio*, *Oligoflexus*)	
Class 5. Nitrospinia** cl. n. Cavalier-Smith (Nitrospinaceae: *Nitrospina*)	
Subphylum 2. Acidobacteria** Cavalier-Smith 2002	
Class 1. Blastocatellia Pascual et al. 2016 (e.g. *Chloracidobacterium*, *Holophaga*, *Terroglobus*)	
Class 2. Nitrospiria cl. n. Cavalier-Smith (e.g. *Nitrospira*, *Leptospirillum*, *Thermodesulfovibrio*)	
**Subphylum 3. Geobacteria** Cavalier-Smith 2002	
Class 1. Deferribacteria cl. n. Cavalier-Smith (orders Deferribacterales** Huber & Stetter 2002; Chrysiogenales** Garrity and Holt 2002, e.g. *Chrysiogenes*)	
Class 2. Nautiliia cl. n. Cavalier-Smith (ε-Proteobacteria** e.g. *Nautilia*, *Campylobacter*)	
**Parvkingdom 2. Spiroplanctia** parvk. n. Cavalier-Smith. **Description:** all eubacteria more closely related to spirochaetes and *Planctomyces* than to *Escherichia coli*. **Etymology** from included groups.	
**Superphylum 1. Spirochaetes** superphyl n. Cavalier-Smith description as for Spirochaetae	
**Phylum 1. Spirochaetae** Cavalier-Smith 2002 (the classes were published in 1992 but not validated)	
Class 1. Spirochaetia cl. n. Cavalier-Smith (e.g. *Spirochaeta*, *Treponema*)	
Class 2. Leptospiria cl. n. Cavalier-Smith (e.g. *Leptospira*)	
Superphylum 2. Planctochlora* superphyl. n. Cavalier-Smith 2019	
**Phylum 1. Planctobacteria** Cavalier-Smith 1987 em. (see Table 3 for 3 infraphyla, 6 classes, 7 orders)	
Subphylum 1. Elusimicrobia** subphyl. n. Cavalier-Smith. Sole class Elusimicrobiia cl. n. Cavalier-Smith (*Elusimicrobium, Endomicrobium*)	
**Subphylum 2. Euplancta** subphyl. n. Cavalier-Smith (e.g. *Planctomyces*, *Chlamydia*; 3 infraphyla)	
**Phylum 2. Sphingobacteria** Cavalier-Smith 1987	
Subphylum 1. Gemmatimonadetes** subphyl. n. Cavalier-Smith. Sole class Gemmatimonadia cl. n. Cavalier-Smith (*Gemmatimonas, Longimicrobium*)	
Subphylum 2. Calditrichae** subphyl. n. Cavalier-Smith. Sole class Calditrichia cl. n. Cavalier-Smith (e.g. *Caldithrix*)	
**Subphylum 3. Chlorobia** subphyl. n. Cavalier-Smith	
Infraphylum 1. Chlorobi** infraphyl. n. Cavalier-Smith	
Class 1. Chlorobiia** cl. n. Cavalier-Smith (e.g. *Chlorobium*, *Thermochlorobacter*, *Chloroherpeton*)	
Class 2. Ignavibacteriia** cl. n. Cavalier-Smith (e.g. *Ignavibacterium*, *Melioribacterium*)	
**Infraphylum 2. Bacteroidetes** ** infraphyl. n. Cavalier-Smith	
**Superclass 1. Bacteroidia** supercl. n. Cavalier-Smith	
Class 1. Cytophagia Nakagawa 2012 (e.g. *Cytophaga*, *Dyadobacter*, *Flexibacter*)	
Class 2. Bacteroidetia cl. n. Cavalier-Smith (spelling corrected for Bacteroidia Krieg 2012) (e.g. *Bacteroides, Alistipes*)	
Class 3. Flavobacteriia Bernardet 2012 (e.g. *Flavobacterium*, *Fluviicola*, *Riemerella*)	
Class 4. Sphingobacteriia Kämpfer 2012 (e.g. *Sphingobacterium*, *Pedobacter*, *Solitalea*)	
Class 5. Chitinophagia Munoz et al. 2017 (e.g. *Chitinophaga*, *Saprospira*)	
Superclass 2. Rhodothermae** supercl. n. Cavalier-Smith	
Class 1. Balneolia Munoz et al. 2017 (e.g. *Balneola*)	
Class 2. Rhodothermia Munoz et al. 2017 (e.g. *Rhodothermus, Salinibacter*)	
Subphylum 4. Fibrobacteres** subphyl. n. Cavalier-Smith. Sole class Fibrobacteriia Spain et al. 2010 (spelling corrected here from original Fibrobacteria; e.g. *Chitinivibrio*, *Fibrobacter*)	
Subphylum 5. Cloacimonetes** subphyl. n. Cavalier-Smith (e.g. *Candidatus* Cloacimonas acidaminovorans)	

Other descriptions of new groups are in the taxonomic appendix

*Probably paraphyletic (some just because neomura are thought to have evolved from within them); all other taxa thought to be clades

**Taxa often ranked unwisely as separate phyla

+Probably polyphyletic

Infrakingdom Gracilicutes comprising four major negibacterial phyla (Proteobacteria, Spirochaetes, Sphingobacteria, Planctobacteria) is well supported by CAT but only weakly by ML. All methods give even stronger support for a broader grouping of eight negibacterial phyla that we treat as new subkingdom Neonegibacteria (Gracilicutes with two partially photosynthetic phyla; plus the two major thermophilic phyla Aquithermota and Synthermota; and two minor heterotrophic phyla Fusobacteria and Hadobacteria). There is weaker support by all methods for a clade comprising Armatimonadetes plus Oxybacteria, which we call subkingdom Eoglycobacteria as its three phyla form the deepest branching clade of negibacteria with LPS (if the tree is correctly rooted between them and Chloroflexi whose OM lacks LPS). Instead of the earlier term Eobacteria, we refer to Chloroflexi plus Eoglycobacteria jointly as Eonegibacteria, arguably the four most ancient negibacterial phyla.

Another important conclusion is that posibacteria are multiply polyphyletic. Actinobacteria and Endobacteria are not sisters. On CAT trees, Endobacteria are maximally supported as sister to Neonegibacteria, whereas Actinobacteria are near maximally (0.99) sisters of Endobacteria plus Neonegibacteria, these two positions being also significantly (but weakly) supported by ML. Within Endobacteria, deepest branching clades are all negibacteria, within them are at least two distinct posibacterial clades with only one membrane. However, internal branching within Endobacteria was inconsistent between CAT, Poisson, and ML, so further work must define the branching order of the major robust subclades, as a later section explains in detail. Irrespective of that uncertainty, our trees strongly indicate that Actinobacteria and at least two subclades of Endobacteria lost the OM independently. Furthermore, mycoplasmas are robustly nested within Bacilli so there is no justification for continued treatment of Tenericutes and Firmicutes as separate phyla: Endobacteria is definitely a clade with endospores its ancestral character, but there have been multiple losses of OM and (independently) of murein. Our trees robustly group the mycoplasma *Mesoplasma* with *Erysipelothrix* and *Coprobacillus* (both in order Erysipelotrichiales), but grouped another mollicute clade comprising *Acholeplasma* and *Haloplasma* with maximal support with *Turicibacter* instead. Thus, there appear to be two independent major mycoplasma clades, so reductive evolution has been rampant in Endobacteria.

The PVC group (classical Planctobacteria) is near maximally supported and consistently groups with good support with *Elusimicrobium* which is morphologically similar and so here included in slightly broadened Planctobacteria. Aquithermota comprising classes Aquificia (=Aquificae) and Thermodesulfobacteriia is maximally supported as sister to Gracilicutes by CAT but that joint clade was not found by ML. By contrast, Synthermota including Synergistetes, Thermotogia, Caldisericia, and Dictyoglomia is a completely distinct thermophilic CAT clade that branches more deeply below both Fusobacteria and Hadobacteria, and is thus the deepest branching neonegibacterial subclade. By ML, Synthermota splits into two robust subclades: Synergistetes and Thermocalda (new subphylum names proposed here), which do not group together, Thermocalda moving to be insignificantly supported sister to Aquificia. Thus, our CAT trees firmly resolve the long-standing controversy over whether the two hyperthermophilic eubacterial groups (Thermotogales and Aquificales) are directly related (Eveleigh et al. 2013). They clearly are not, each being nested separately within a broader thermophilic group, which are not even sisters. As several authors have argued, Aquithermota are more closely related to Gracilicutes than to Synthermota and the erratic contradictory groupings of *Aquifex* and *Thermotoga* together or apart on early ML rDNA trees that varied with taxon sampling reflected insufficient taxon sampling and evolutionarily less realistic algorithms plagued by long-branch artefacts.

## Neomuran ribosomal protein trees

**Fig. 6.**
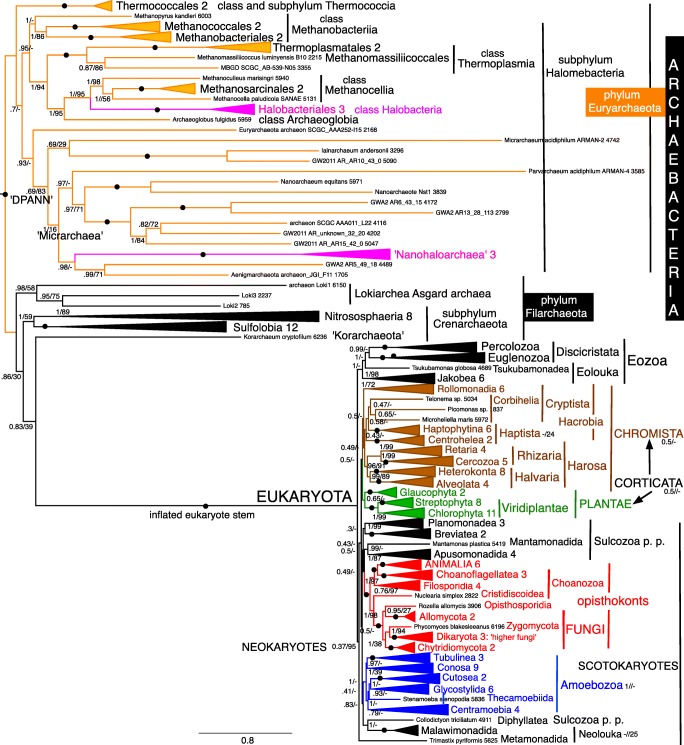
Site-heterogeneous PhyloBayes CAT-GTR tree for 51 ribosomal proteins from 203 neomura representing all the most divergent lineages. Support values for bipartitions are: posterior probabilities for CAT-GTR (left; 51,189 trees summed after removing 40% as burnin: maxdiff 1; convergence was prevented by four persisting contradictions deeply within neokaryotes), RAxML bootstrap percentages for 100 pseudoreplicates (right). To fit the page branches for major taxa are collapsed; all names are shown on uncollapsed trees in Supplementary material, e.g. Fig. S1. Includes all taxa from Figs. 3 and 4

When eukaryotes and archaebacteria are included in the same 51-protein CAT tree (Fig. 6), topology of each is only very slightly changed from their single-domain trees. Eukaryotes appear rooted between maximally supported clade Eozoa (all but one internal branch maximally supported with Fig. 3 topology) and an insignificantly supported (0.36) neokaryote clade. Corticata, Plantae, Chromista, and Corbihelia are weakly supported clades; opisthokonts, Animalia, Amoebozoa, Alveolata, Heterokonta, and Rhizaria maximally supported clades. Internal phylogeny of Amoebozoa differed in putting Cutosea within Discosea, not as its sister (from the 351-protein tree (Kang et al. 2017) probably neither is correct but Fig. 3 more nearly so). Though the consensus tree is better than the eukaryote-only tree (Fig. 3) in recovering clades Plantae and Chromista, the two chains did not fully converge because of a few contradictions within eukaryotes only: (1) on chain 2 *Trimastix* was strongly sister to Breviatea as in Fig. 6 but chain 1 put it alone as the deepest branching eukaryote with the root between it and all others; (2) Plantae and Chromista were strongly (1, 0.99) clades on chain 1 but on chain 2 a haptophyte/*Picomonas*/*Telonema* false ‘clade’ weakly disrupted Plantae and Planomonadida weakly intruded into the remaining chromists; (3) Centroheliozoa moved slightly; (4) within amoebozoan Discosea deep branching slightly differed.

Archaebacterial topology was identical to Fig. 4 with DPANN within euryarchaeotes, except for ‘Nanohaloarchaea’ being sisters of Aenigmarchaeota, not Halobacteriales and class Methanobacteriia being a clade as on 200-protein trees (Petitjean et al. 2015). Thus, both major methanobacterial classes were clades. Eukaryotes were strongly excluded from lokiarchaeotes and did not group with them. If the tree is rooted within archaebacteria between euryarchaeota (including DPANN) and Filarchaeota, eukaryotes appear to be sisters of ‘Korarchaeum’ with low (0.63) support. This may be artefactual as distant outgroups are often attracted to such long unbroken branches; eukaryotes would only have to cross this and another weakly supported node to join archaebacteria between Filarchaeota and Euryarchaeota, which may therefore both really be clades. Basal branches within archaebacteria are much more spread out and on average much longer than in eukaryotes, implying a faster evolution; they are also much more variable in length (and would be even more so if we had not excluded the longest branches to reduce artefacts; we also excluded the longest branch eukaryote taxa, but even allowing for that eukaryote evolutionary rates are generally much more uniform than for archaebacteria implying greater evolutionary constraints).

The ML tree differed from the archaebacteria-only tree in showing DPANN as sister to euryarchaeotes not within them and in moving ‘Nanohaloarchaea’ to sister of ‘Parvarchaeum’. Eukaryotes remained outside lokiarchaeotes as insignificantly (39%) sisters of ‘Korarchaeum’—they would have to cross only that branch and one other with trivial 30% support to join the tree between Filarchaeota and euryarchaeota/DPANN. Thus, neither tree convincingly supports eukaryotes branching within Filarchaeota. For eukaryotes, ML gives maximal support for paraphyly of Eozoa with eukaryotes being rooted between Percolozoa and Euglenozoa plus all other eukaryotes, i.e. within discicristates, clearly contradicting the site-heterogeneous tree—internal topology of Eozoa is unchanged except for *Seculamonas* and *Jakoba* not being sisters. For eukaryotes the ML tree was marginally worse than for eukaryotes only or CAT for some of the most weakly placed clades as *Collodictyon* intruded into Corticata as insignificant sister of glaucophytes and breviates wrongly grouped with apusomonads. However, the tree overall was not grossly distorted by either method by adding genetically extremely distant archaebacteria, strongly supported clades being the same, though CAT appears slightly more resistant to such perturbation.

With only 26 RPs, the eukaryote CAT tree (Fig. S7) was different in a few respects but previously strongly supported patterns generally remained strongly supported, notably internal phylogeny of Eozoa, opisthokonts, Amoebozoa, Viridiplantae, haptophytes, rollomonad cryptists, and Harosa. However, branching at the base of neokaryotes was less conserved, e.g. Plantae being disrupted by Viridiplantae moving into Chromista and Glaucophyta and Heliozoa outside corticates to weakly join *Collodictyon* and Planomonadida respectively. These difficult-to-place groups and the sulcozoan lineages also appear misplaced on the 26-gene ML tree. For archaebacteria, 26 RP trees rearranged euryarchaeotes, placing Themococcales within Methanobacteria—not as the deepest group—by both CAT and ML; both put DPANN as sister to not within euryarchaeotes. Both placed the eukaryote root between Percolozoa and other eukaryotes with strong support for its exclusion from all others (0.92; 98) and thus for paraphyly of Eozoa. However, ML and CAT were contradictory for where eukaryotes joined archaebacteria: ML still grouped them with ‘*Korarchaeum*’, but CAT put them as sister to ‘*Korarchaeum*’ plus all other Filarchaeota except Lokiarchaea, i.e. closer to the base of archaebacteria than in the 51-protein tree. This further emphasises the unreliability of the position of eukaryotes within Filarchaeota; contrary to a neomuran CAT-GTR tree using 55 RPs (Zaremba-Niedzwiedzka et al. 2017) and a greater diversity of Asgaardia but an unspecified number of eukaryotes (likely fewer than in ours), none of our neomuran trees grouped eukaryotes with lokiarchaea. Our analyses agree with theirs in having a thaumarchaea/Sulfolobia clade, but theirs effectively have eukaryotes one node lower than in any of ours. Thus taking their analysis and ours together eukaryotes appear in three different contradictory places deep within Filarchaeota.

Contradictions amongst these trees with respect to the root of eukaryotes and where they join archaebacteria are unsurprising given that on Fig. 5, the stretched eukaryote stem that separates them represents a mean of 2.83 substitutions per site. As some sites are invariant and others evolve much faster than average most variable positions will have been overwritten many times since eukaryotes and archaebacteria diverged; scarcely any will have retained phylogenetically informative information about where the two ends of the stem historically joined each crown group. Very likely, chance convergences in the most variable positions will overwhelm genuine ancestral phylogenetic signal. The longest included unbroken DPANN branches are even longer, corresponding to a mean of nearly four substitutions per site, so one expects LBA artefacts to be serious for them (as others have convincingly argued: Brochier et al. 2005) and reasons for disbelieving the exclusion of DPANN from euryarchaeotes and separation of the two groups of halophilic bacteria on some RP trees. By contrast, mean branch length of crown eukaryotes represents only about 0.656 substitutions per site so a substantial amount of phylogenetically informative sequence information must remain. But because of explosive radiation at the base of eukaryotes, there was too little time between deepest branch points for many phylogenetically informative mutations to accumulate, so basal branch order is necessarily less well supported than in the more spread out deep eubacterial tree. The 26 universal RPs shared with eubacteria underwent almost as much change (mean 2.6 substitutions per site in the eukaryote stem) and show a similar disparity in rate patterns as the 51 neomuran ones.

## Prokaryote ribosomal protein trees

**Fig. 7:**
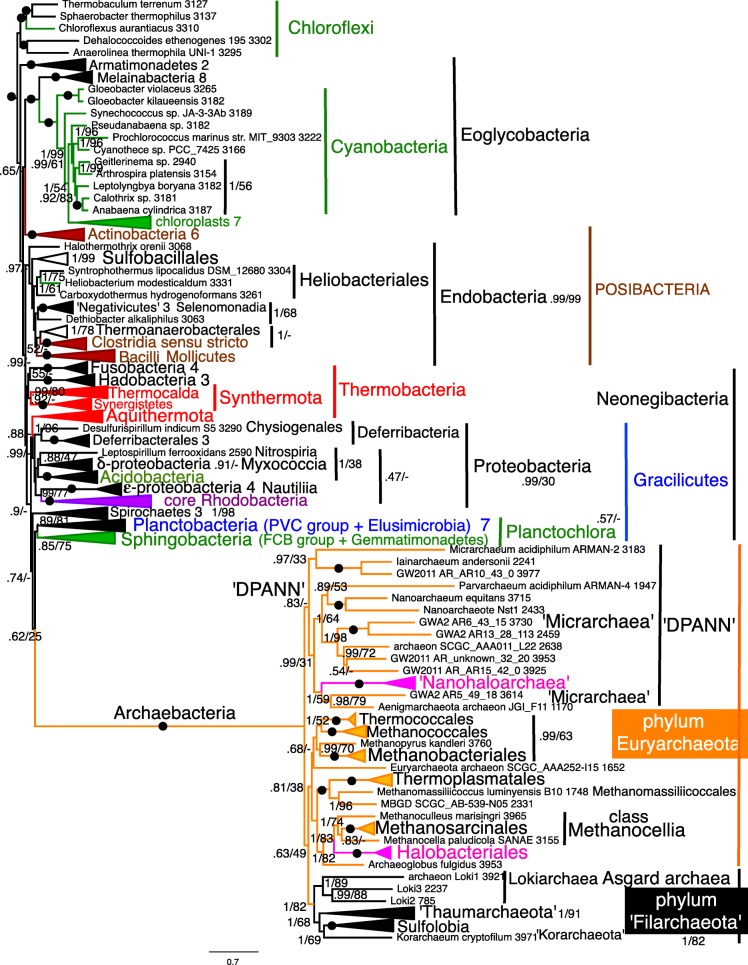
Site-heterogeneous prokaryote PhyloBayes CAT-GTR tree for 26 ribosomal proteins from 60 archaebacteria and 151 eubacteria representing all the most divergent lineages. Consensus of two chains; support values for bipartitions are posterior probabilities for the CAT-GTR (left; after removing 40% as burnin 179,537 trees summed; maxdiff 0.179537), RAxML bootstrap percentages for 100 pseudoreplicates (right). To fit on the page branches for major taxa are collapsed; their names are on uncollapsed trees in Supplementary material, e.g. Fig. S9. Archaebacteria are strongly excluded from Posibacteria and branch within Neonegibacteria. weakly as sister to Planctochora

There was a marked difference in archaebacterial deep branching and the apparent position of their root according to whether CAT trees used 51 or 26 archaebacterial RPs. With 51, ‘Nanohaloarchaea’ were sister of Halobacteriales within euryarchaeotes with maximal support on both chains which converged on the same topology within archaebacteria (Fig. S9) and ‘Micrarchaea’ were weakly sisters of Filarchaeota, so there was no DPANN clade. With only 26 RPs (Fig. 7) by contrast, there was a DPANN clade and the root appeared between it and other archaebacteria. These trees were also contradictory for a few parts of the eubacterial backbone (but showed all the same major clades). Both showed archaebacteria emerged from eubacteria as weakly supported sisters of Planctochlora, the joint Planctobacteria/Sphingobacteria clade. That is consistent with evidence discussed below that Planctochlora ancestrally had prenyl diether membrane lipids in addition to acyl esters and thus are credible eubacterial ancestors for archaebacteria. With 26 RPs, both chains supported that position. But when 51 archaebacterial and 26 eubacterial RPs are combined in a prokaryote tree (Fig. S9), they conflicted: chain 2 put Archaebacteria as sisters to Planctochlora (negligible 0.43 support), whereas chain 1 grouped them weakly (0.6) with Gracilicutes plus Aquithermae. In Fig. 7, the stem joining eu- and archaebacteria has a mean of 4.3 amino acid substitutions per site. Therefore, it is highly improbable that archaebacterial RPs retain enough ancestral-clade-specific information to place this long stem with precision within the roughly 20 major eubacterial lineages. Its apparent position is almost certainly lower in the tree than its true position, as the faster-evolving parts of its sequences needed to fix it relationship to more recent eubacterial branches must be overwritten; only the slowest evolving regions could retain useful phylogenetic information, and others may largely reflect vagaries of multiple overwriting of ancestral sequences—as previously argued for rDNA (Cavalier-Smith 2002a).

Despite such multiple overwriting, addition of the long eubacterial outgroups, and eubacterial data being absent for 25 of its RPs, internal phylogeny of archaebacteria on Fig. S9 has scarcely changed from Fig. 4. In both, Filarchaeota are maximally supported as a clade, with internal phylogeny identical except for the maximally supported position of *Ignicoccus*, in a moderately supported slightly different position with this crenarchaeote subclade in Fig. 4. Within Micrarchaea, basal branching order is identical but much more strongly supported in Fig. S9, which differs only in the basal part of the 5-member environmental DNA subclade that is sister to the *Nanoarchaeum* clade; its Fig. S9 topology is markedly more strongly supported. Within short-branch euryarchaeotes, there are only two differences: (1) *Methanomassiliicoccus* maximally sister to an environmental lineage that weakly branches one node lower with very weak support; (2) Thermococcales move up one node to be sister to *Methanopyrus*. In these respects, except for the position of Thermococcales, the Fig. 7 topology is better supported and more consistent between chains, implying that adding eubacteria despite their distance stabilised topology by breaking up the basal stem, perhaps allowing better reconstruction of ancestral states for some branches. These features of Fig. S9 topology may therefore better reflect internal phylogeny of the three major groups. The only other difference is the position of Micrarchaea: sister to Euryarchaeota in Fig. S9, within it as sister to all except Thermococcales in Fig. 3. Given weak support for Micrarchaea being sister to Filarchaeota in Fig. S9 and its long branches, we suggest addition of an extremely distant outgroup may have pulled it artefactually one node away from its position within euryarchaeotes and misplaced the root by one node through long-branch attraction towards it. If so, Micrarchaea should really be within euryarchaeotes one node higher than Thermococcales, as in Fig. 4, and the archaebacterial root should be between Euryarchaeota and Filarchaeota, not sister to DPANN as in Williams (a possible long-branch attraction (LBA) artefact) or within short branch Euryarchaeota as in Raymann (a possible artefact of taxonomic undersampling).

When prokaryote trees are restricted to the 26 RPs shared with eubacteria (Fig. 7), archaebacterial CAT topology unsurprisingly changes slightly. Filarchaeota remain maximally supported with the same internal topology except that *Ignicoccus* moves one node. Euryarchaeote phylogeny is changed not only by exclusion of ‘Nanohaloarchaea’ (and their grouping with Aenigmarchaeota within Micrarchaea) but also by Thermococcales and *Methanopyrus* separated and intermingling with the Methanobacteria, and one change within the problematic environmental DNA clade. We suggest that for archaebacteria, the Fig. S9 topology is more reliable, being based on nearly twice as many genes and more concordant with the major euryarchaeote phenotypes. CAT 26-RP trees put the archaebacterial root between DPANN and other archaebacteria but support is low for the likely artefactual non-DPANN clade (0.63).

ML gives the same archaebacterial topology with 51 as with 26 genes but with often lower support; both also place the root within DPANN between the *Micrarchaeum*/*Iainarchaeum* clade and the rest, but are contradictory as to which is the deepest branch—*Micrarchaeum*/*Iainarchaeum* with 51 RPs and other DPANNs with only 26. Support is insignificant for both; both are likely to be artefacts and less accurate than CAT trees. There is no reason to prefer the ML topology to the evolutionarily more realistic CAT ones.

Positioning archaebacteria within eubacteria was also sensitive to gene sampling and method. With 51 proteins, ML put them as sister to a spurious (19%) Sphingobacteria/Spirochaete ‘clade’ (different from the CAT positions) with insignificant (18%) support. 26 proteins (Fig. S10) put them as sister to Sphingobacteria only (ML: insignificant 30%). Thus, ML tends to group archaebacteria with Sphingobacteria, with trivial support, whereas CAT does so with Sphingobacteria/Planctobacteria, with weak but higher support.

## Eukaryote-eubacterial two-domain ribosomal protein trees

**Fig. 8:**
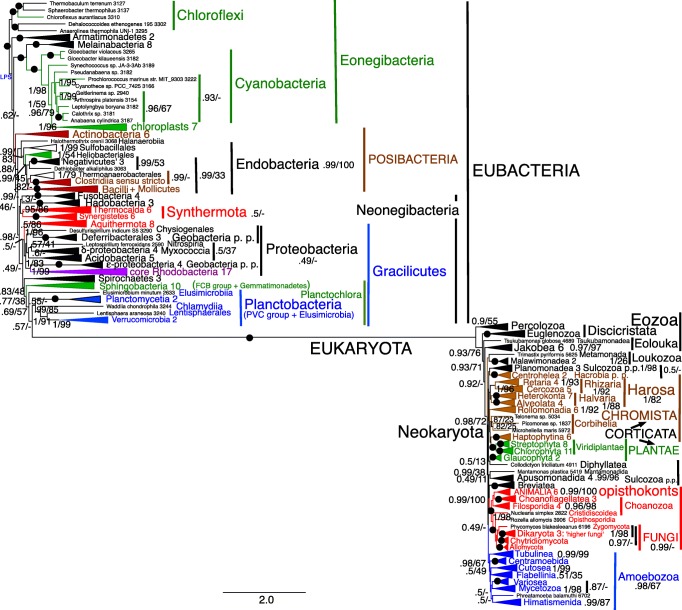
Site-heterogeneous 2-domain PhyloBayes CAT-GTR tree for 51 ribosomal proteins from 143 eukaryotes and 26 ribosomal proteins from 151 eubacteria representing all the most divergent lineages. Support values for bipartitions are from left to right: posterior probabilities for the CAT-GTR (left), RAxML bootstrap percentages for 100 pseudoreplicates (right). To fit on the page branches for major taxa are collapsed; their names are shown on uncollapsed trees in Supplementary material, e.g. Fig. S12. Despite 33,393 trees being summed after removing the first 17,893 as burnin the two chains did not converge (maxdiff 1) because of a few persistent topological differences (with 0.5 support or less) at the base of neonegibacteria and neokaryotes; both strongly excluded eukaryotes from Posibacteria and placed them within gracilicute Neonegibacteria. The root of eukaryotes beside Percolozoa within Eozoa was the same on both chains; one chain placed eukaryotes within Planctobacteria as on the consensus tree, but more strongly so, whereas the other put them more weakly two nodes more deeply as sister to Planctochlora

If there were no long-stem problems, these two-domain trees should theoretically be as reliable as the two preceding ones for rooting eukaryotes and correctly placing the neomuran stem within eubacteria. But in practice, one might expect them to be less reliable as the stem connecting eubacteria and eukaryotes is even longer: from Fig. 8, it has mean of 10.7 amino acid substitutions per site. In theory, eukaryotes should be placed within eubacteria in the same position as archaebacteria if there were a genuine phylogenetic signal able to show their correct position. However, eukaryotes appear within Planctobacteria only, as sister to the PVC group (exluding *Elusimicrobium*); the apparent position of the eukaryote root is within Eozoa between Percolozoa and all other eukaryotes (moderate support 0.84 and by ML 76%). ML puts eukaryotes within Planctobacteria as sister to Planctomycetales plus *Elusimicrobium* (insignificant 18%) a likely false clade. Reducing the eukaryote data to the 26 shared genes (Fig. S11), puts eukaryotes as insignificantly sisters of Planctomycetia only (0.49%) and the eukaryote root more narrowly within Percolozoa between *Naegleria* only and all other eukaryotes, both unlikely; the corresponding ML tree (Fig. S12) has the eukaryote root between holophyletic (54%) Percolozoa and the rest (77% for non-percolozoan eukaryotes being a clade) and shows eukaryotes as sister to all Planctobacteria except *Elusimicrobium*. Thus, these two-domain trees consistently support the theory that eukaryotes evolved from Planctobacteria (Reynaud and Devos 2011). Though the prokaryote trees instead suggest a slightly deeper position as sister to Planctochlora as a whole, both sets are weakly supported, as expected from the inferred degree of substitutional overwriting. More importantly, both two-domain trees strongly exclude neomura from both Actinobacteria and Endobacteria and thus clearly contradict a posibacterial origin of neomura (Cavalier-Smith 1987c, 2002a) and strongly indicate that their ancestors were neonegibacteria, and more weakly that they were most likely gracilicutes of Planctochlora subclade, rather than any of the deeper-branching hyperthermophilic neonegibacteria (Thermobacteria) as had been suggested by some three-domain rDNA trees. The weakness of the signal for their precise position within Planctochlora is emphasised by the two CAT chains being contradictory: chain 2 grouped eukaryotes with Planctobacteria (0.85) as sister to all except *Elusimicrobium* (0.68) whereas chain 1 put them as sister (0.55) to all Planctochlora, as were archaebacteria on the prokaryote tree, but excluding them from Planctobacteria insignificantly (0.45).

Eukaryote internal phylogeny is no more obviously disturbed on the 51 or 26 RP tree by adding the much more divergent eubacteria than it was for adding archaebacteria, so we shall not describe the eukaryote parts of these trees in detail: they exhibit similar tendencies for corticate, chromist and plant holophyly to be degraded and planomonads to intrude wrongly into chromists.

Eubacterial internal phylogeny is also very little changed by adding the 51 eukaryotic RPs. The relative branching order of the six deepest branching phyla (Chloroflexi, the three eoglycobacterial phyla and Actinobacteria and Endobacteria) is identical, and the closer relationship of Actinobacteria to Endobacteria plus Neonegibacteria than to Eoglycobacteria is more strongly supported (0.99 not 0.62). Except for Endobacteria whose deep branching order was poorly supported on Fig. 5, their internal phylogeny is identical (but one minor difference within chloroplasts). Neonegibacteria contains the same major clades with almost identical internal phylogeny but their relative positions are somewhat altered, probably because the long-stem eukaryotes branch within them as weakly supported (0.63) apparent sisters of Planctobacteria. Thus, eukaryotes do not branch in either of the two positions found for archaebacteria in the prokaryote tree. This conflict suggests that there were too many amino acid substitutions along the stem joining eukaryotes or archaebacteria to eubacteria for their correct position to be consistently determined. Despite eukaryotes branching within Gracilicutes, the relative branching order within Gracilicutes of all subgroups is identical and thus rather stable. However, unlike Fig. 5 where Gracilicutes were strongly supported as a clade (0.99) as were Aquithermota (1), the two chains placed Aquithermota contradictorily, so their position as sister to Proteobacteria in the consensus tree (Fig. 8) is a weakly supported compromise. In chain 2, Aquithermota were a maximally supported clade strongly supported (0.99) as sister to strongly supported (0.98) Proteobacteria, whereas in chain 1, Aquificia separated from Thermodesulfobacteriia and entered Synthermota as weak (0.78) sister to Thermocalda, whereas Thermodesulfobacteriia entered Gracilicutes as sisters (0.82) of δ-Proteobacteria (now much more weakly, 0.49, supported as a clade). As Aquithermota remain a well supported (86%) clade by ML outside Gracilicutes, its discordant splitting in one CAT chain is probably artefactual, perhaps caused by the very different eukaryote sequences. The relative branching order of Synthermota, Hadobacteria, and Fusobacteria also differ from Fig. 5.

The ML tree insignificantly groups all three major clades of thermophilic bacteria together, but Synthermota is not a clade. Actinobacteria move up the tree away from Endobacteria, insignificantly sisters of Hadobacteria. Within Endobacteria, Bacilliia plus Clostridiales sensu stricto (i.e. classical posibacterial endobacteria) are weakly (56%) supported as a clade unlike in Fig. 8. Eukaryotes appear within Gracilicutes but move to within Planctobacteria as sisters (no support: 17%) of a probably false grouping of *Elusimicrobium* and Planctomycetales.

With only 26 RPs the CAT tree (Fig. S11) did not fully converge as the two chains had a strongly supported conflicting topology within eubacteria. One chain gave essentially the same topology as Fig. 8; the other is basally very different, as Melainabacteria/Cyanobacteria moved upwards to become strongly sisters of Fusobacteria and Actinobacteria moved up to be strongly sister of Hadobacteria. Strong support for this aberrant topology made it dominate the consensus tree (Fig. S11). Despite these contradictions, both chains agreed in placing eukaryotes within Planctobacteria as sister to all Planctobacteria other than *Elusimicrobium* (0.67, 0.68 support) and in putting the eukaryote root within Percolozoa between *Naegleria* as in Fig. S11. Thus, although the main eubacterial clades are not altered by addition of eukaryotes, the backbone branching pattern of eubacteria is destabilised more by adding 26 eukaryote RPs with eubacterial relatives than by adding 51 eukaryote RPs. It is as if the presence of the 25 neomuran-specific proteins without eubacterial partners prevents the eukaryote sequences from destabilising the eubacterial part of the tree. With ML for 26 RPs (Fig. S12), the Melainabacteria/Cyanobacteria clade remains as in Figs. 5 and 8, but Actinobacteria move up to join Hadobacteria with insignificant (30%} support; the eukaryote root is within Eozoa between Percolozoa and the rest and eukaryotes are insignificantly (21%) sister of the probably false grouping of *Elusimicrobium* and Planctomycetales.

## Universal three-domain ribosomal protein trees

**Fig. 9:**
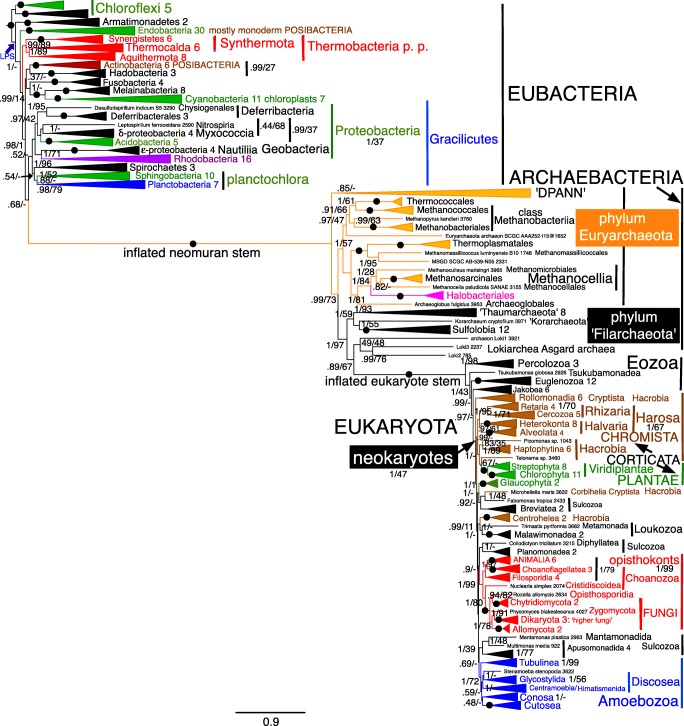
Site-heterogeneous universal three-domain PhyloBayes CAT-GTR tree for 26 ribosomal proteins from 143 eukaryotes, 60 archaebacteria, and 151 eubacteria representing all the most divergent lineages. Support values for bipartitions are from left to right: posterior probabilities for the CAT-GTR (left), RAxML bootstrap percentages for 100 pseudoreplicates (right). To fit on the page, branches for major taxa are collapsed; their names are shown on uncollapsed trees in Supplementary material, e.g. Fig. S1. As the chains did not converge, this figure is for chain 2 with ML support values also mapped on to it. After removing the first 20% as burnin, the remaining 19,165 trees were summed. Deep branching order of prokaryote phyla is markedly more disturbed than in 2-domain trees (Figs. 6, 7, and 8)

On both CAT-GTR and ML trees, irrespective of whether 51 or 26 neomuran RPs were used, the apparent eukaryote root was between Percolozoa and all others (Fig. 9) as in the eubacteria-rooted tree (Fig. 8), not between Eozoa and neokaryotes as in the neomuran tree (Fig. 6). However, the position of eukaryotes within archaebacteria and of neomura within eubacteria varied, as did the apparent root of archaebacteria, and the branching order of eubacteria was generally more distorted compared with Fig. 5 than in two-domain trees and eukaryote topology also worse. Overall, three-domain trees appear notably less trustworthy than single and two-domain trees, making it unfortunate that they have been largely exclusively relied on in most previous work on the tree of life, except for the comparisons of Raymann et al. (2015).

The 26 RP CAT trees did not converge for the position of neomura, so Fig. 9 for a single chain and Fig. S13 for a consensus tree exemplify the two contradictory topologies CAT yielded for 26 RPs. In Fig. 9, neomura are sister to Gracilicutes as a whole not just to Planctochlora or the subset of Planctobacteria as in two-domain trees. In this tree, internal phylogeny of Gracilicutes is standard but non-gracilicute phyla are drastically rearrranged: Aquithermota enter Synthermota as sister to Thermocalda and Actinobacteria and Melainabacteria/Cyanobacteria move upwards to join Fusobacteria and Hadobacteria respectively as in the aberrant chain described in the previous paragraph. Chain 1 by contrast had normal positions for Actinobacteria and Melainabacteria/Cyanobacteria but greater aberrations for the thermophiles—Aquithermota are split: Thermodesulfobacteriaceae entered Gracilicutes as sisters (0.99) of δ-Proteobacteria whereas Aquificia move to be sisters of Thermocalda (weakly). Neomura are strongly (0.97) sister to that probably false Thermocalda/Aquificia clade. A broadly similar phylogeny is seen in the consensus tree (Fig. S13) but support for this position of neomura is negligible (0.38) and Hadobacteria are attracted within Synthermota also. Figure 9 put eukaryotes as sister to Lokiarchaea (0.89), whereas Fig. S13 put them within Lokiarchaea, weakly sister to Loki1 (0.48), contradicting neomuran trees that mostly grouped them with ‘Korarchaeum’. Basal branching of eukaryotes was almost completely unresolved, with maximal support for contradictory but maximally supported branching order at almost every backbone node—though most eukaryotic subgroups are well supported apart from problems as usual at the base of Hacrobia and scotokaryotes.

**Fig. 10:**
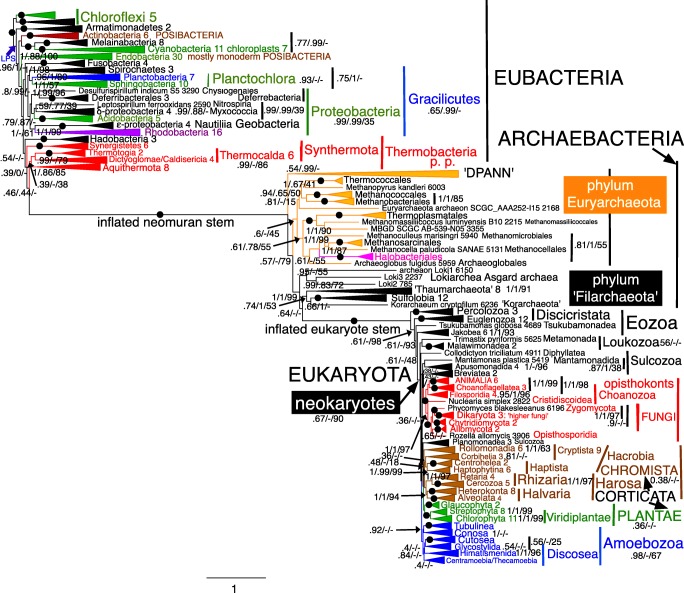
Site-heterogeneous universal three-domain PhyloBayes CAT-GTR tree for 51 ribosomal proteins from 143 eukaryotes and 60 archaebacteria and 26 ribosomal proteins from 151 eubacteria representing all the most divergent lineages. Support values for bipartitions are from left to right: posterior probabilities for the CAT-GTR (84.962 trees summed from four independent chains after removing 4035 trees as burnin: maxdiff 1), posterior probabilities for the CAT-Poisson (29,287 trees summed after removing 9,872 treees as burnin), RAxML bootstrap percentages for 100 pseudoreplicates. To fit on the page branches for major taxa are collapsed; their names are shown on uncollapsed trees in Supplementary material, e.g. Fig. S1

With 51 neomuran RPs CAT-GTR three-domain trees, we ran four separate chains that also did not converge but there were markedly fewer distortions within eubacteria and eukaryotes; none showed the aberrant upwards movement of Actinobacteria and Melainabacteria/Cyanobacteria but all differed in the position of neomura. Chain 4 put neomura within Gracilicutes as sister to the Spirochaete/Planctochlora clade (0.63) with negligible support for their not being closer to Planctochlora (0.43) and maximal support for eukaryotes as sister to all Filarchaeota except lokiarchaeotes; 1-3 related eukaryotes in contradictory ways to the rearranged non-gracilicute thermophiles. Chain 1 put neomura as sister (0.44) to Thermocalda, eukaryotes as sister to all Filarchaeota except lokiarchaeotes (0.87); chain 2 put neomura as sister to Thermocalda/Aquithermota (0.58) and eukaryotes as sister to ‘Korarchaeota’ (maximal support); chain 3 put neomura as sister to Thermocalda/Aquificia/Hadobacteria and eukaryotes as sister to Lokiarchaea (maximal support). These four contradictory positions for neomura and three for eukaryotes confirm the conclusion from 26 RP trees that three-domain trees cannot reliably position either. For what it is worth (not much), the consensus tree for all four chains (Fig. 10) puts neomura as insignificant (0.44) sister to Thermocalda/Aquithermota and eukaryotes as weakly (0.64) sister to all Filarchaeota except Lokiarchaea. Figure 10 with 51 neomuran RPs weakly (0.54) supports DPANN as a clade (including Micrarchaea and ‘Nanohaloarchaea’) and only weakly (0.57) places it as the deepest archaebacterial branch; with only 26 RPs, Figs. 9 and S13 (strongly 0.98, 0.99) have DPANN as the deepest archaebacterial branch. The corresponding CAT-Poisson trees also did not fully converge (maxdiff 1; 40% burnin; 2 chains with 29,287 trees summed) but both chains rooted eukaryotes within Amoebozoa between Tubulinea and other eukaryotes with strong support and put eukaryotes as sister to Loki2/3 with fairly strong support and rooted archaebacteria within non-DPANN euryarchaeotes in two contradictory places; in all these respects, they contradicted all CAT-GTR trees, which are theoretically more accurate. One chain put neomura as sister to Synthermota, the other within Synthermota as sister to *Caldisericum*/*Coprothermobacter* only (0.52), adding two more conflicting positions thus confirming the inability of RP trees to place neomura or root archaebacteria or eukaryotes consistently amongst methods. Despite all these conflicts, the internal branching order of eubacteria was essentially as in Fig. 5 and that of eukaryotes largely consistent with Fig. 3, indicating that the theoretically inferior reconstructive ability of CAT-Poisson was mainly confused by neomuran hyperaccelerated and eukaryote stems not by an inability to reconstruct intradomain branches correctly.

ML places neomura insignificantly (40%) sister to Sphingobacteria, using 26 or 51 RPs, with strongly supported Planctobacteria the next branch. Eubacterial and eukaryote backbone branching orders are insignificantly supported but most subclades are as in single-domain CAT trees. For 26 RPs, Fig. S14 has Melainabacteria/Cyanobacteria in the normal position as sister to Armatimonadetes with insignificant (40%) support, Aquithermota and Thermocalda are both clades, so in these respects, the ML tree is less perturbed by long branch neomuran and eukaryote stems than CAT-GTR. However, ML shows Actinobacteria as weakly (27%) sisters of Hadobacteria, so this likely artefact is consistent between these methods (not seen on CAT-Poisson). ML put eukaryotes weakly (67%) sister to Lokiarchaeota and rooted archaebacteria inside DPANN beside the ‘Iainarchaeum’/‘Micrarchaeum’ clade (negligible support for this as the deepest clade: 34%). When 51 neomuran RPs are included, ML puts eukaryotes more weakly (59%) sister to lokiarchaeotes, and the archaebacterial root between the ‘Micrarchaeum’/‘Iainarchaeum’ clade and the rest (even weaker support: 24%), almost certainly an LBA artefact. But for inclusion of neomura, Gracilicutes are a clade with the same internal branching as in all other trees. Aquithermota groups (insignificant support) within Synthermota as sister to Thermocalda and Actinobacteria are sisters of Hadobacteria with insignificant support (33%).

## Overall pattern and limitations of the universal ribosomal protein tree

The tree is effectively three densely branched multistem bushes (crown eukaryotes, archaebacteria, eubacteria) interconnected by two long unbranched stems. It can be interpreted correctly only by mapping it onto the fossil record and understanding the reasons for the two immensely long bare stems.

The depth of the eubacterial bush corresponds to 3.5 Gy, from the age of RuBisCo-based carbon fixation given by isotopic ^13^C/^12^C ratios in Archaean kerogen, which at least as long ago as 3.41 Ga is sometimes associated with plausible morphological microfossils (Wacey et al. 2011) or stromatolites (Tice and Lowe 2004), and depth of the eukaryote bush (the only one certainly a clade) only to ~ 850 Ma. The earliest generally accepted crown eukaryote cellular fossils are only ~ 760 My old (likely corticate scales and likely scotokaryote amoeba tests; see Cavalier-Smith 2013a). The oldest known steranes, commonly viewed as eukaryote markers even though several disparate eubacteria make simple steranes, are in rocks dated 820-720 Ma (Brocks et al. 2017) suggesting that eukaryotes were not abundant before 820 Ma.

But all early crown eukaryote fossils are neokaryotes; Eozoa the earliest branch on our RP trees do not fossilise well, so if Eozoa are older than and ancestral to neokaryotes as suggested by a majority of our trees that put Percolozoa most deeply (Figs. 8, 9, 10, S7, S8, S11, S12, and S13 using proteins of eukaryote host origin), the last eukaryote common ancestor (LECA) is somewhat older, as previously suggested (Cavalier-Smith 2010b, 2013a, 2014, 2017). However, if Eozoa are a sister clade to neokaryotes, as suggested by one of our neomuran trees (Fig. 6) and a similar outgroup-rooted tree using proteins of eubacterial origin likely derived via mitochondrial symbiogenesis (He et al. 2014), then Eozoa and neokaryotes would be essentially the same age. A later analysis using mitochondria-derived proteins concluded instead that Eozoa are a clade that is sister to Corticata (Derelle et al. 2015); if that were historically correct, Eozoa would be effectively the same age as Corticata (probably ~ 745 Mya based on our RP tree proportions) and thus a little younger than LECA, so the absence of eozoan fossils would not bias the inferred age of LECA. On Fig. 6, LECA appears only marginally older than the neokaryote clade; from the Fig. 6 tree proportions, if neokaryotes were 800 My old, LECA would be dated ~ 816 Ma by applying a uniform eukaryotic molecular clock; if neokaryotes are only 760 Ma, the LECA date would be only 775 Ma. But if our trees placing the eukaryotic root instead within Eozoa between Percolozoa and all others were correct (which we doubt; see below), the inferred age for LECA would be older—from Fig. 9 proportions ~ 1.0 Gy. This illustrates the importance of knowing the position of the eukaryote root for mapping sequence trees onto the fossil record.

On present evidence, it remains unlikely that crown eukaryotes are older than ~ 850 ± 30 My, the same as argued earlier when mapping rRNA trees onto the fossil record (Cavalier-Smith 2002a, 2006a). If the depth of the eubacterial crown represents 3.5 Gy, and that of the eukaryote crown ~ 0.85 Gy, they should have a ratio of ~ 4.1 if amino acid substitution rates were the same in both. In fact (ignoring the accelerated longer branches of chloroplasts and cellular endoparasites like Rickettsias and mycoplasmas), the ratio is only ~ 1.7 so most eubacterial RPs have evolved about 2.3 times more slowly than most eukaryotic RPs, implying that selection against change is stronger in eubacteria. Figures 3, 4, 5, 6, 7, 8, 9, and 10 omitted mitochondrial RPs as they evolve far faster than chloroplast RPs and have immensely longer branches, presumably because purifying selection preventing random divergence is weaker. Nonetheless, the point where the mitochondrial stem diverges from within the α-proteobacterial clade (Fig. S15 and arrow on Fig. 5) gives an upper bound to the age of both LECA and stem eukaryotes. Applying a constant eubacterial molecular clock to the Fig. S15 RP tree, we estimated the upper bound of age of the first mitochondria and therefore LECA to be ~ 1.18 Ga, but the actual age of LECA is likely younger.

The age of archaebacteria is less clear as they have no morphological fossils and the oldest direct evidence for their age is ~ 820 My old isoprenoids from halophilic archaebacteria, at least some of which were probably methanogens as indicated by the presence of crocetane (Schinteie and Brocks 2017). Given that some methanogens can be halophilic and the possibility that other early archaebacterial clades might have been also, these lipids cannot be regarded as specific markers for the halophilic euryarchaeote clade shown on Fig. 4, which appears to be over 30% younger than the last archaebacterial common ancestor (LACA), which was likely a methanogen if Fig. 4 topology is correct. However, if we assume that these lipids did come from the base of that clade then we could use them to set an upper bound to LACA's age: 1.17 Ga. In a later section, we use an LGT from viridiplant chloroplasts to ‘Cenarchaeales’ (Petitjean et al. 2012) to date the euryarchaeote/filarchaeote divergence at 1.18 Ga. Thus, three independent phylogenetic/fossil calibrations give the same young ages for eukaryotes and archaebacteria: both are less than 1.2 Gy and more than 0.85 Gy old, i.e. ~ 1.0 ± 0.15 Gy old. Thus, present evidence is compatible with the idea that eukaryotes and archaebacteria are sisters of equal age, as Cavalier-Smith (1987c, 2002a, 2006a, 2014) long argued; but if they actually branch within archaebacteria, either within or as sisters to Filarchaeota, archaebacteria would be slightly older. As there is no other credible evidence for the actual age of archaebacteria, there is no reason to think they are as old as eubacteria. Although all our trees weakly suggest that the eukaryote stem emerges near the base of Filarchaeota—but in several contradictory places, resolution is not good enough to eliminate the idea that eukaryotes and archaebacteria are sisters, which many aspects of cell evolution favour, and that euryarchaeotes and filarchaeotes mutually diverged at essentially the same time as archaebacteria and eukaryotes in an unresolvable trifurcation. Certainly, there is no evidence from RP trees or from palaeontology that archaebacteria are substantially older than stem eukaryotes. Neomura are likely about three times younger than eubacteria. Fallacious arguments for greater archaebacterial antiquity stem from methanogenesis and their (non-unique) lipids, whose relatively recent evolution is explained in detail in later sections. The chimaeric origin of reverse DNA gyrase from two eubacterial enzymes has long been evidence that archaebacteria evolved from and thus are younger than eubacteria (Cavalier-Smith 2002a)—we argue below that their reverse gyrase most likely came from Aquithermota which must therefore be older than archaebacteria, as must Planctobacteria, if neomura evolved from them (as Fig. 8 suggests).

If archaebacteria are of similar age to eukaryotes, their longer branches imply that RPs of shorter branch archaebacteria evolve ~ 2.5× faster than RPs in most eukaryote lineages and thus about 5.8 times faster than most eubacteria. Some archaebacterial lineages, notably many DPANN, evolve much faster still, which makes their accurate placement problematic (see below); RP evolutionary rate disparity within DPANN is greater than shown on our trees as the longest branches were omitted to reduce long-branch artefacts—in eubacteria, except for mitochondria, we omitted none for that reason so they are genuinely more clock-like than archaebacterial RPs. A few extra-long eukaryotic branches (notably free-living Foraminifera and genomically reduced intracellular parasitic microsporidia and retarian *Mikrocytos*) were omitted for the same reason, but most eukaryote lineages have more uniform branch lengths even than eubacteria, indicating that even though *mean* amino acid substitution rates are higher than for eubacteria their relative rates are mostly more constrained than in eubacteria.

The difficulty of deciding whether eukaryotes are sisters of archaebacteria or branch deeply within them proves that they cannot be as much as 3–4 times as old as eukaryotes, as eubacteria probably are: if they were, eukaryotes should branch shallowly within them with maximal support, which no sequence trees show. Thus, the relative proportions of ribosomal trees combined with fossil evidence for eukaryote recency have long proved that archaebacteria cannot be as old as eubacteria, as Cavalier-Smith (1987c) first emphasised and later elaborated in detail (Cavalier-Smith 2002a, 2006a, d). Therefore, archaebacteria are much younger than eubacteria. Most evidence indicates that they are also substantially younger than cyanobacteria which almost certainly evolved before the great oxygenation event (GOE) of 2.4 Gy ago that made the atmosphere oxidising and which left the best eubacterial morphological fossils (but see later discussion of SMC protein evolution claimed to show cyanobacteria as younger than archaebacteria). Contrary to their name, archaebacteria are the youngest, not oldest major bacterial group and are irrelevant to the origin of life. They have often been assumed to be ancestrally anaerobic (Weiss et al. 2016), but more critical reevaluation of the evolution of aerobic respiratory chains in a later section shows that they were not and were ancestrally facultative aerobes that evolved a novel kind of methanogenesis different from the likely earlier aerobic version recently discovered in eubacteria (Teikari et al. 2018).

Widespread, but mistaken, beliefs that archaebacteria are as old as eubacteria stem from misinterpreting the significance of the two long bare stems on rRNA and some protein trees (including RPs) located between (1) archaebacteria and eubacteria (called the neomuran stem as it is at the base of the neomuran clade: Cavalier-Smith 2002a) and (2) between the ancestral prokaryotes and derived eukaryotes (the eukaryote stem), as well as similar long bare stems that join the subtrees of protein paralogue trees of molecules like protein synthesis elongation factors (EF) (Cavalier-Smith 2002a, 2006c, 2014). EF subtrees also have long bare internal neomuran and eukaryote stems (Baldauf et al. 1996); as in RPs, the neomuran stem is longer than the eukaryote stem, indicating greater sequence change in ribosome-related proteins during the origin of neomura than during the origin of eukaryotes, but the interparalogue stem is longer still. That greater length does not imply a longer time span, but much faster evolution during a brief time than occurred within any of the three terminal bushes. Ultrarapid evolution for a short period followed by deceleration is the general explanation for the greater length of these stems than of the bushes. Episodic hyperacceleration also explains the bareness (no side branches) as ultrarapid evolution was so shortlived that no radically different subgroups evolved before rates returned to the normal low ones maintained by strong purifying selection: for detailed explanation, see Cavalier-Smith (2002a, 2006c); Cavalier-Smith et al. (2018) use Foraminifera that display similar inflated stems on multiprotein trees but have billions of well-preserved and well-dated fossils to prove that this explanation of long bare stems applies equally to them.

The great length of the EF interparalogue stem was caused by rapid adaptive evolution to make two different proteins with substantially different functions (EF-Tu and EF-G); during that divergent adaptation directional selection for novelty was strong, but once the two distinct GTPase functions were largely perfected most selection was against further change so evolutionary rates plummetted to a low level throughout eubacteria (dependent largely on the relative strengths of mutation pressure and purifying/stabilising selection). That divergent change happened before the last universal common ancestor of all life (LUCA), which fossil evidence and sequence trees (summarised above) in conjunction with much cell biology tell us must have been the same as the last eubacterial common ancestor, not an imaginary ‘progenote’ as postulated by Woese and Fox (1977a, b). By contrast, episodic hyperacceleration in the neomuran stem did not occur close to LUCA, as wrongly assumed without any evidence (Woese and Fox 1977a, b), but ~ 2.5 Gy later and must have been caused by novel changes during the neomuran revolution when cotranslational synthesis and secretion of N-linked glycoproteins evolved after eubacterial murein was lost, which entailed coevolutionary changes in the signal recognition particle (SRP) and the evolution of all the neomuran RPs for which homologues are unknown in eubacteria—the most radical change in protein synthesis in the history of life. The major SRP protein (SRP54/Ffh) and its receptor (SRα/FtsY) also arose by gene duplication and great divergence in LUCA during which Ffh evolved a new C-terminal extension and FtsY a new non-homologous N-terminal extension (Gribaldo and Cammarano 1998); the SRP/receptor paralogue tree for the shared region also has a longer neomuran than eukaryote stem but the interparalogue stem is intermediate in length implying that its ancient pre-LUCA divergent sequence change was less than the far more recent change during the origin of neomura.

Woese and Fox realised that the long neomuran and eukaryote stems must be caused by temporary ultrarapid evolution, much faster than that within the branched bushes, but wrongly assumed that both accelerations took place close to the origin of life before the basic machinery of translation was perfected and proper cells evolved. Both then and later, they ignored fossil evidence that crown eukaryotes are so much younger than eubacteria indicating that this assumption cannot possibly be true, and that the long stem for eukaryotes at least must have been caused by radical changes to ribosomes billions of years after LUCA. They expressed the prejudice that such radical change could only occur close to the origin of life and continued to believe that for most of its history ribosomal molecules have been accurate chronometers.

The case of mitochondria tells us how radically wrong that was. Their ribosomes evolved from α-proteobacterial ribosomes roughly 2.5 billion years after the first eubacterium, which had only 54 RPs, yet before LECA are inferred to have had 72 RPs, having evolved 19 new RPs not found in prokaryotes and probably lost one proteobacterial RP (Desmond et al. 2011). Numerous other major changes occurred in mitoribosomes by loss and addition of RPs in many eukaryote lineages, as well as large changes in mt rDNA sequences greater than those differentiating the three domains. In most eukaryote mitochondria, SRPs have been lost and opisthokont mitoribosomes are permanently attached to the inner membrane, largely making membrane proteins. Their 82 RPs are more even than the 79-80 cytosolic ones (Bieri et al. 2018; Greber and Ban 2016). This means that radical changes in ribosome structure are possible long after LUCA and occurred several times; even though such changes may cause translational errors, these errors do not prevent conservation of encoded protein sequences as they do not change the DNA germline. Probably more can be tolerated in mitochondria (where few different proteins are made), so purifying selection is less stringent for mitoRPs than cytoRPs, e.g. in mammals mitoRPs evolve 13 times as fast. The reader can see from Fig. S15 how non-clock-like mitochondrial RPs are compared with the far more slowly evolving eubacterial ones. The situation is even more dramatic than that figure shows because for most eukaryotes mitoRPs were even more divergent and so immensely harder to align and we omitted them from our analysis; many omitted species would have even longer branches. Figure S15 also emphasises that for mitochondria, most acceleration occurs in the crown part of the tree, not in the stem whose length is 4–8 times shorter than the crown—the exact opposite to the eukaryote and neomuran stem acceleration which are relatively much longer and so will have erased phylogenetic signal more than happened for mitochondria whose stem is relatively short. Note that the eukaryote crown is immensely longer for mtDNA than for nuclear DNA even though both must be the same age, proving systematic gross acceleration for mitochondria and deceleration for nuclear RPs since LECA.

Many others appear ignorant of both Woese’s assumption of early rapid acceleration and Cavalier-Smith’s (2002a) identification of neomuran and eukaryote stem hyperacceleration instead and of the contradictions amongst protein paralogue trees as to the position of the root; so mistakenly (a) place the root in the neomuran stem, and so fundamentally misunderstand early cell evolution, and (b) apply a single clock to the whole tree, leading to absurdly inflated age estimates for archaebacteria and eukaryotes (e.g. Betts et al. 2018; Blank 2009; Sheridan et al. 2003; with others, a later section criticises in detail). Gogarten-Boekels et al. (1995) accepted 10-fold acceleration in the neomuran stem (probably an underestimate) but even so imagined that the long neomuran stem indicated a billion or so years of evolution and speculated that the absence of any side branches in that imaginary billion years was caused by meteorite bombardment extinguishing all earlier radiating life except for two lineages that diversified to form eubacteria and neomura a billion or more years after LUCA. That interpretation is incompatible with the accurate dating of cyanobacterial origins from the RP tree on the assumption of episodic hyperaccelaration in the neomuran and eukaryote stems involving manyfold faster amino acid substitution than the much more nearly clock-like diversification within crown eubacteria. Episodic hyperaccelaration by a much greater factor in the neomuran and eukaryote stems simultaneously explains more simply than highly speculative meteorite bombardment, for which there is no evidence, why both stems are bare; only accepting radically different stem and crown rates by at least two orders of magnitude allows accurate detailed mapping of the whole RP tree onto the fossil record and only that explains why the eukaryote stem plus crown branch is so much longer than the archaebacterial branch. Episodic ultrafast evolution affecting some molecules not others explains why the relative proportions of the same parts of the universal tree are so different for some molecules than for others. A later section gives a new example of a protein that has undergone radically different local accelerations from RP but to which a single clock has also been wrongly applied globally. Fundamental misinterpretation of universal trees by the entirely false assumption of a universal molecular clock is a pervasive problem for virtually all sequence trees. Refuting that assumption hundreds of times, as has been done, has sadly had no effect on many who calculate dates by computers, ignoring evidence for more massive rate changes than their algorithms can model, so obtain results exemplifying the principle ‘garbage in garbage out’.

The evidence from mapping first rRNA and now RP trees onto the fossil record shows that the grossly stretched neomuran and eukaryote stems both reflect two much more recent episodic hyperaccelerations in ribosomal evolution that took place billions of years later, most likely > 2.5 Ga after the origin of life. The scale of the stretching is so great that it explains why ribosomal trees are so bad at accurately reconstructing the root of eukaryotes and archaebacteria or their precise eubacterial ancestors even though the much slower evolving crown sequences of all three domains make these molecules very good for resolving their internal phylogeny so long as one uses numerous RPs and site-heterogeneous trees. They are worst for basal eukaryote phylogeny because its divergences were more sudden than the equally numerous eubacterial ones for which we believe Fig. 5 gives the most accurate tree to date.

Unfortunately Woese’s mistaken assumptions and ill-defined erroneous notion of a progenote lying midway along the neomuran stem have been so pervasively influential that many archaebacterial researchers similarly ignorant of fossil and other evidence against it still imagine that archaebacteria are ancient, as do some others who refuse to take the evidence against it seriously (e.g. Koonin (see his comments as a referee of Cavalier-Smith (2006c)) and certain others who have axes to grind for interpretations that are entirely untenable and at variance with the evidence summarised here).

## Taxon-rich multi-RP trees are reasonably accurate within domains

We expected eukaryote 51 RP trees to be less accurate than earlier studies with 187 proteins and 26 RPs trees to be less accurate still and ML to be less accurate than CAT. We also expected topological deviations from 187-protein trees would be mainly in areas where numerous branches diverge almost simultaneously, traditionally the hardest to yield consistent results: notably at the base of Hacrobia, Plantae, and scotokaryotes. Our RP trees confirm all four expectations as explained above. We also expected that the more distant the outgroups, the more likely would internal phylogeny of eukaryotes be perturbed. As predicted, eukaryotes-only or neomuran-only trees were generally more concordant with 187-protein trees than were three-domain trees; the worst trees for eukaryotes, with the lowest basal resolution and highest contradictions between chains, were the three-domain trees for 26 proteins. Yet even these were markedly more accurate for eukaryotes than most previously published three-domain trees with much sparser taxon sampling. Internal phylogeny of nearly all major clades was the same for both 51 and 26 RP trees as with 187 proteins, and except in the three difficult regions relationships amongst them were the same. Most were strongly or maximally supported with 51 RPs, but some were lower with only 26 genes. Despite this, our trees include a very few seriously wrong placements of major eukaryote branches with high support, but markedly fewer instances than on previous multidomain trees—all undersampled for eukaryotes.

We conclude that eukaryote *taxon-rich* trees for 51 RPs are reasonably accurate provided site-heterogeneous methods are used, but are not perfect and thus cannot be a substitute for trees with hundreds of genes. This is primarily because basal eukaryote branches are so numerous and so tightly clustered that only small amounts of still conserved phylogenetically informative changes can have occurred in the stems of the deepest branches. It is therefore not worth discussing the few deviations from genically more comprehensive trees in detail. A combination of hundreds of proteins, site-heterogeneous methods, and care to exclude the fastest evolving positions is necessary to establish accurately the most difficult parts of eukaryote branching topology (Kang et al. 2017). The extremely tight clustering of basal eukaryotic lineages on RP trees confirms earlier arguments that the basal eukaryotic radiation was indeed explosive, a pattern not dismissable as an artefact of substitution saturation.

That is strikingly shown by the basal branching of eubacteria, which are about four times as old, being much more spread out and thus likely inherently more gradually divergent. This difference is striking on Figs. 8, 9, and 10, where especially for neokaryotes, basal radiation resembles an explosive big bang (as previously emphasised for rDNA: Philippe and Adoutte 1996, 1998) that is necessarily inherently difficult to resolve. Within eubacteria, that problem is less, for basal branches on Fig. 5 are almost all strongly supported by CAT, though markedly less by ML. Stronger support for the eubacterial tree backbone stems primarily from their basal branches being more spread out in time, so more differences could accumulate between successive branches between phyla than possible for the basal neokaryote radiation that probably took only a few tens of million years around 800 Ma. A second reason why basal eubacterial branching is highly credible is that RP evolutionary rates must be only about half as fast in eubacteria as in eukaryotes (because the eubacterial crown is only on average about twice as deep as the neokaryote crown despite being four times older: 3.5 Gy. That age is set by the age of the ^13^C/^12^C isotopic ratios in ancient hydrocarbons interpreted as evidence for RuBisCo photosynthetic carbon fixation that is restricted to eubacteria (specifically Negibacteria)). Only one feature of the eubacterial backbone appears doubtful (relative positions of the non-photosynthetic negibacterial phyla Hadobacteria and Fusobacteria: sometimes successive, sometimes sisters). Its overall pattern appears robust, much more so than past rDNA trees and in places differing distinctly from them as detailed below.

Robustness of the eubacterial tree allows us to conclude that some eubacterial phyla are much younger than others. For example, given the rooting shown, cyanobacteria (ancestors of chloroplasts) are notably younger than Chloroflexi, Endobacteria, or any gracilicute phyla, assuming a mean molecular clock (reasonably as eubacterial branch lengths are broadly similar, differing by less than twofold; unlike for neomura). Taking the mean of the Gloeobacteria and somewhat longer tip lengths of subphylum Phycobacteria (i.e. cyanobacteria with thylakoids: Cavalier-Smith 2002a) to represent the present, the Fig. 5 tree proportions suggest an age of ~ 1.3 Gy for crown cyanobacteria and ~ 2.3 Gy for stem cyanobacteria. As this is closely similar to the GOE (~ 2.4 Gya), it is likely that oxygenic photosynthesis originated close to divergence of Cyanobacteria and Melainabacteria. This close agreement of fossil evidence and our RP tree rooted on Chloroflexi itself supports our rooting. There would be no such agreement if (as far too many suppose) it were rooted halfway along the neomuran stem. The chloroplast stem emerges from cyanobacteria later, at ~ 1.0 Ga but that could be an overstimate if its longish branch is artefactually deep because of LBA. Likewise, α-proteobacteria, the ancestors of mitochondria, whose age sets an upper limit to that of eukaryotes, appear to be > 2 Gy younger than negibacteria, consistent with their last common ancestor being aerobic and giving an extreme upper bound to the origin of crown eukaryotes of ~ 1.1 Ga; the position of the mitochondrial stem within proteobacteria on Fig. S15 corresponds to ~ 0.97 Ga. Even this may be a bit too old for crown eukaryotes if the long mitochondrial branch is somewhat too low within α-proteobacteria as can happen by LBA. A slightly younger date would fit the absence of eukaryote-like steranes before 820 My (Brocks et al. 2017) and of definitely neokaryote cellular fossils before 760 Ga (Cavalier-Smith 2013a) and the idea that neomura date back only to ~ 850 My (Cavalier-Smith 2002a). Having a robuster tree, we can map other evolutionary events onto it and better evaluate claims for LGT. For example, later sections argue that the role of LGT has been exaggerated in evolution of photosynthesis, respiration, and nitrogen fixation, and that LUCA was a negibacterial anaerobic photosynthesiser with nitrogen fixation and respiratory electron transfer abilities, and eubacterial flagella.

For basal archaebacteria, the RP tree is markedly less well resolved, for three reasons. First, the deep branches are part of an explosive radiation, as in eukaryotes, not well spread out as in eubacteria, so fewer ancient changes can have occurred between them. Second, archaebacterial RPs evolve faster than eubacterial or eukaryote ones: their tree branches are longer than those of eubacteria and around three times longer than those of eukaryotes, despite the oldest fossil evidence for archaebacterial lipids (820 My ago) suggesting they are the same age as eukaryotes (for which fossil steranes of complexity indicating eukaryotes are no older than 720–820 Ma: Brocks et al. 2017) as does the LGT from chloroplasts noted above. Third, they are markedly less equal in evolutionary rate than in eubacteria. DPANN lineages (secondarily miniaturised archaebacteria with exceptionally diverse rates, probably because of their simplified genomes) are a nuisance for tree reconstruction as they have likely lost most information that would accurately place them (see below). However, despite these difficulties, the bipartition between Euryarchaeota/DPANN and Filarchaeota is consistently strong in archaebacteria-only trees, and the majority of their branching topology other than for DPANNs appears to be reliable at least for site-heterogeneous trees (somewhat better for 51 than for 26 proteins).

## Major improvements to the eubacterial tree

**Fig. 11:**
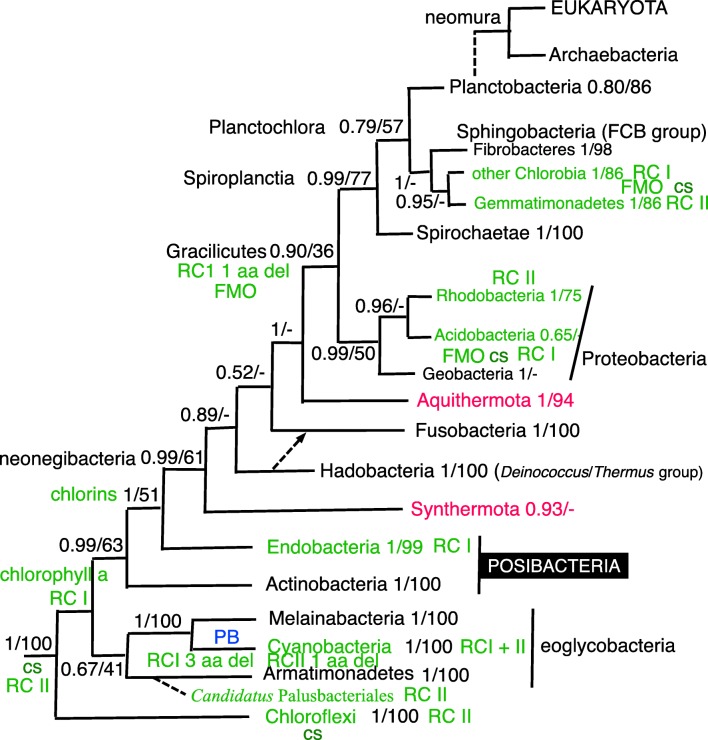
The 14 eubacterial phyla recognised here. For two exceptionally diverse phyla (Proteobacteria, Sphingobacteria) their three major subbranches, here ranked as subphyla (but often treated as several smaller phyla), are also shown. Support for the monophyly of each (from Fig. 5) is extremely high as is CAT-tree support from Fig. 5 for their relative branching order, except for the position of Hadobacteria which sometimes appear as sister to Fusobacteria (dashed arrow). Their branching order is otherwise very stable on site-heterogeneous trees restricted to Eubacteria, but adding one or both highly divergent neomuran groups on multidomain trees makes branching order less stable, there being a strong tendency for the major thermophilic phyla (Aquithermota, Synthermota) to group together or become partially intermixed with Hadobacteria/Fusobacteria; these changes are likely artefacts. Phyla with some photosynthetic members are in green; the different types of photosynthetic reaction centres (RC and characteristic deletions) and presence of FMO, chlorins, phycobilisomes (PB) and chlorosomes (cs) are mapped onto the tree; it is unknown if uncultured *Candidatus* Palusbacteriales (‘Eremiobacteria’: Ward et al. 2019) has chlorosomes—as not in our analyses, its likely position in Armatimonadetes (dashed line) is only weakly established; its discovery increases the likelihood that ancestral eubacteria (i.e. LUCA) had RCII. The position of neomura (dashed line) is based on two-domain RP trees (see text)

RP trees agree with rDNA trees in showing with maximal or near maximal support the monophyly and deep distinctiveness of 10 established major groups: the eight phyla Chloroflexi, Armatimonadetes, Cyanobacteria, Hadobacteria (=*Deinococcus*/*Thermus* group), Fusobacteria, Spirochaetae, Planctobacteria (largely = PVC group), Sphingobacteria (largely = FCB group); and subphyla Actinobacteria and Endobacteria, which in light of our RP trees showing they are not sisters, we now rank as separate phyla. Unlike many recent eubacterial ‘phylum’ names in common use, all these taxon names were validly published (Cavalier-Smith 2002a; Tamaki et al. 2011) even though the International Code of Prokaryote Nomenclature (ICNP) does not apply to categories ranked above class (Parker et al. 2014). Though Hadobacteria was recently rejected as a class name (Tindall 2014) for unspecified reasons that may be invalid, we use it here at its original non-rejected phylum rank (Cavalier-Smith 1992b, 1998b) as it is less cumbersome than the three-word ‘group’ name. Our trees confirm that candidate phylum ‘Melainabacteria’ (lacking cultured representatives (Di Rienzi et al. 2013; Utami et al. 2018) except for predatory *Vampirovibrio* (Soo et al. 2015b)) is sister to Cyanobacteria and show for the first time that their joint clade is probably sister to Armatimonadetes. Eoglycobacteria is a suitable new name for this robust clade comprising Cyanobacteria, Melainabacteria, and Armatimonadetes, as it is apparently the earliest branching glycobacterial clade, best ranked in formal classification as a subkingdom. Glycobacteria was introduced as the infrakingdom name for all eubacteria with outer membranes containing LPS (Cavalier-Smith 1998b). Our RP trees also reveal two major previously unrecognised thermophilic clades (Synthermota including hyperthermophilic Thermotogales; Aquithermota including hyperthermophilic Aquificales; formally established as two new phyla in the Taxonomic Appendix) and confirm that Proteobacteria are phylogenetically much wider than has been generally appreciated, supporting the broadening of Proteobacteria in the eubacterial classification of Cavalier-Smith (2002a). Contrary to the trees of Yutin et al. (2012) and Boussau et al. (2008b), but in agreement with most rDNA trees, Thermotogia and Aquificia are not sisters. Contrary to Lasek-Nesselquist and Gogarten (2013), Thermotogia, Aquificia, and Synergistetes are not a clade. Raymann et al. (2015) excluded Aquificia, Synergistetes, and Fusobacteria. Our trees also show that Elusimicrobia are better included in Planctobacteria and Gemmatimonadetes in Sphingobacteria than treated as separate phyla as in the past. Thus, the whole diversity of major named eubacterial groups can now be included in just 14 robustly monophyletic phyla as summarised in Fig. 11 and Table 1, a great simplification compared with 29 in Ruggiero et al. (2015).

Our trees strongly support monophyly of clade Gracilicutes established at infrakingdom rank to embrace Proteobacteria, Spirochaetae, Planctobacteria, and Sphingobacteria based on a combination of indels, rDNA trees, and ultrastructure (Cavalier-Smith 2006c), but show that their branching order then deduced by cladistic arguments is almost certainly incorrrect. In all our eubacterial trees, Planctobacteria and Sphingobacteria are sisters, forming a clan here designated Planctochlora that is robustly sister to Spirochaetae, Proteobacteria always being sister to Spirochaetae plus Planctochlora. That is precisely the same gracilicute branching order as Yutin et al. (2012) found using 50 RPs and FastTree, which is slightly less accurate than RAxML used here (Price et al. 2010), and must be substantially less accurate than PhyloBayes CAT (their WAG evolutionary model is also less accurate than LG used for ML here). This exact branching order and Gracilicutes as a clade were all strongly supported in the 56-protein eubacterial ML tree of Boussau et al. (2008b) though they did not sample all major planctochloran groups. This gracilicute branching order is conserved in nearly all our multidomain trees so is robust to inclusion of highly divergent neomuran relatives. (We refer to Planctochlora as a clan not a clade as many multidomain trees imply that neomura evolved from Planctochlora; if that is correct, they are paraphyletic.) The pioneering multidomain site-heterogeneous trees of Lasek-Nesselquist and Gogarten (2013) and Raymann et al. (2015) also found a Planctochlora clade but spirochaetes were not its sisters but branched one node lower, possibly because they included a much narrower range of Proteobacteria than we did.

The non-gracilicute part of the trees differ from those of Yutin et al. (2012) in numerous ways. A highly misleading feature of their results discordant with almost all other studies (references in Davis et al. 2013) is that Mollicutes were grouped with Fusobacteria, not placed within Bacilli as in our trees and those of Yutin and Galperin (2013), Boussau et al. (2008b), and Davis et al. (2013). Lasek-Nesselquist and Gogarten (2013) and Raymann et al. (2015) both excluded Mollicutes. Eubacteria-only trees robustly place Aquithermota as sister to Gracilicutes, whereas Fusobacteria, Hadobacteria, and Synthermota branch successively more deeply; they also robustly show that these five groups collectively form a major clade that we call Neonegibacteria, as it embraces all negibacteria except the deep branching Eonegibacteria (Chloroflexi, Armatimonadetes, Cyanobacteria, Melainabacteria) and two lineages belonging in Endobacteria. These findings will be discussed individually after considering Endobacteria (often confusingly called Firmicutes), long an evolutionarily and taxonomically confusing group as it includes both negibacterial and posibacterial phenotypes—as our trees strongly confirm. So many important evolutionary questions are raised by endobacterial diversity that we treat them in seven sections.

## Striking evolutionary diversification of Endobacteria

When Actinobacteria and Endobacteria were established as subdivisions (=subphyla), they were assumed to be sisters, as some but not a majority of rDNA trees had shown, and were grouped together in phylum Posibacteria believed to be ancestrally characterised by a shared thick murein wall (Cavalier-Smith 2002a). Conceptually, Posibacteria (originally ranked as phylum: Cavalier-Smith 1987b) included all eubacteria then believed to lack an outer membrane (OM) (Cavalier-Smith 1987c) and did not refer to their Gram-positive staining as it was clear at the outset that some posibacteria (notably Mollicutes) stained Gram negatively and some negibacteria with OMs had thicker walls and stained Gram-positively (e.g. *Deinococcus*)*.* It was assumed that endospore-forming bacteria (e.g. *Selenomonas*) that stain Gram negatively because they lack a thick wall had an outer membrane (OM) with lipopolysaccharide (LPS) and were ancestral to Posibacteria, postulated to have arisen from them by a single loss of murein (Cavalier-Smith 1987b, c), so ‘Selenobacteria’ were excluded from Posibacteria and tentatively grouped (as subphylum) with Fusobacteria and Fibrobacteria as new glycobacterial phylum ‘Eurybacteria’ (Cavalier-Smith 1998b). After it was found that all Heliobacteria made endospores (Kimble-Long and Madigan 2001), their relationship to ‘Selenobacteria’ appeared stronger despite no LPS having been found in Heliobacteria (Beck et al. 1990). As it then appeared that the earlier assumption that ‘Selenobacteria’ had an OM was mistaken, both groups were transferred to the new posibacterial subphylum Endobacteria and placed in class Togobacteria on the assumption that the toga of Thermotogales was an S-layer as the outermost layer of Heliobacteria appeared to be (Cavalier-Smith 2002a). Later, evidence accumulated that ‘Selenobacteria’ actually have an OM not an S-layer, so both were removed from Posibacteria and grouped with Fusobacteria as a phylum ‘Eurybacteria’ (Cavalier-Smith 2006d), which though used subsequently (Cavalier-Smith 2009, 2010a, 2014) was never validated nomenclaturally and eventually abandoned as polyphyletic (Ruggiero et al. 2015).

Most ‘Selenobacteria’ including *Selenomonas*, *Sporomusa*, and other endospore-forming genera and close relatives clearly having an OM were recently formally grouped as class Negativicutes (Marchandin et al. 2010). However, that class is not now valid under the latest edition of ICNP which requires that class names are formed by adding -ia to the stem of the type order of the class (here Selenomonadales). We therefore establish new class Selenomonadia in accord with that rule (Taxonomic Appendix). Genome sequencing confirmed that ‘Negativicutes’ have an OM with LPS (Campbell et al. 2014) and led to their classification into three orders (Campbell et al. 2015). Selenomonadia (=Negativicutes) is invariably a robust clade always nested within unimembranous groups without an OM. Their sister is Dethiobacteria; the only electron micrograph (Sorokin et al. 2008) is too fuzzy to show whether its outermost dense layer is an OM or an S-layer (which we consider more likely as we found no genomic evidence in GenBank for OM-related proteins). Genome sequencing gave no evidence for an OM in *Heliobacterium*, which on our trees groups not with Selenomonadia but strongly as sister to *Syntrophothermus* (classified with it in Clostridiales) and *Carboxydothermus* placed in the separate order Thermoanaerobacterales. Genome sequencing also gave no evidence for an OM in *Carboxydothermus* or *Syntrophothermus* (Djao et al. 2010; Wu et al. 2005); ultrastructurally *Carboxydothermus* clearly has only a single membrane and peptidoglycan is thin (Wu et al. 2005); *Syntrophothermus* also appears to have an outer S-layer and thin murein (though micrographs are fuzzier) but no OM (Sekiguchi et al. 2000). Thus, the maximally supported clade comprising *Carboxydothermus*, *Syntrophothermus*, and *Heliobacterium* (here all grouped in new order Heliobacteriales) apppears to be uniformly monoderm in phenotype, without an OM, yet with much thinner murein than in the robust clostridial subclade comprising *Clostridium*, *Oscillibacter*, and *Anaerostipes*, which we refer to as Clostridiales sensu stricto (s. s.); as noted below, the heliobacterial clade appears to lack teichoic acids unlike thick-walled endobacteria. The 50-RP ML tree of Yutin and Galperin (2013), using Treefinder LG+G, also excluded *Carboxydothermus*, *Syntrophomonas*, and *Heliobacterium* from both Clostridiales s. s. and Thermoanaerobacterales, though they did not form one clade. However, in a taxonomically immensely richer PhyloBayes CAT analysis of 21 RPs from Clostridia only, *Carboxydothermus*, *Syntrophothermus*, and *Heliobacterium* were a robust clade branching in the same order (Kunisawa 2015); that study also robustly showed Clostridia s. s. as a clade.

Thus, in all three studies, *Carboxydothermus* does not group with other Thermoanaerobacterales, which on our trees form a completely robust clade within Endobacteria comprising *Thermosediminibacter* (flagellate Gram-negative thermophilic anaerobes, with no thin-section EM and no mention of OM proteins in genome: Pitluck et al. 2010), *Thermoanaerobacter* (thermophilic anaerobes some with endospores and no OM), *Caldicellulosiruptor* (flagellate asporogenous hyperthermophilic anaerobes with posibacterial type cell walls), and *Caldanaerobacter* (anaerobic spore formers). Cavalier-Smith (2006c), considering Clostridiales too diverse, published a separate order Heliobacteriales (not yet validated). However, these Thermoanaerobacterales appeared paraphyletic as Clostridiales s. s. grouped within them in (Kunisawa 2015). The position of Clostridiales s. s. was inconsistent on our CAT trees: sister either to Thermoanaerobacterales or to Bacilli/Mollicutes. Kunisawa’s analysis in this respect is probably more reliable because of its richer taxon sampling (though he excluded Mollicutes), so we suspect that Clostridiales s. s. and Thermoanaerobacterales are a joint clade with Thermoanaerobacterales ancestral to Clostridiales. That would be consistent with both having thick murein walls and being anaerobic, whereas thick-walled Bacilli are largely aerobic. Now it is certain that *Heliobacterium* does not group with Clostridiales s. s. and *Carboxydothermus* does not group with Thermoanaerobacterales s.s., we expand Heliobacteriales to include *Syntrophothermus* and *Carboxydothermus* (Taxonomic Appendix).

The deepest branch in Endobacteria is Gram-negative *Halothermothrix* (order Halanaerobiales), whose genome reveals a typical glycobacterial OM with lipopolysaccharide (LPS) and typical endosporulation genes. The second deepest branch comprises *Symbiobacterium*, *Thermaerobacter*, and *Sulfobacillus*, whose branching topology is maximally supported, yet are all also classified in Clostridiales, showing Clostridiales to be deeply paraphyletic (or polyphyletic: see below). Often Gram-negative *Thermaerobacter* lacks an OM (Spanevello et al. 2002) and has no spores. *Sulfobacillus thermophilus* is spore forming; neither its genome nor that of five other species gave evidence of an OM or LPS. *Symbiobacterium* forms endospores (Ueda et al. 2004) but its genome does not evidence an OM or LPS. Thus, this clade appears uniformly monoderm in membrane topology; we remove it from Clostridiales as separate new order Sulfobacillales (Taxonomic Appendix). Kunisawa (2015) included *Thermodesulfobium* and *Coprothermobacter* in his analysis which were then assumed to be Clostridiia (Ludwig et al. 2009b). Our trees all decisively exclude them from Endobacteria and show that they are successively sisters with strong support to *Caldisericum*, often unwisely placed in its own phylum; they further show that this joint clade is robustly sister to *Dictyglomus*, also unwisely given its own phylum, and that this wider clade is robustly sister to Thermotogia forming thermophilic clade Thermocalda, which on most of our trees is strongly sister to Synergistia, also unnecessarily treated as a separate phylum. This negibacterial clade is here called phylum Synthermota (see Taxonomic Appendix). Our analyses therefore fully confirm for the first time Kunisawa’s suspicion based on gene order and gene absence that *Thermodesulfobium* and *Coprothermobacter* are neither endobacteria, nor sisters, but far away on the tree.

## Polyphyly of Mollicutes

Our trees strongly show that Mollicutes nest firmly within Bacilli, so must be derived from them by murein loss; the first rDNA trees grouped *Mycoplasma* with Clostridia/Bacilli but lacked resolution to pinpoint their origin (Fox et al. 1980). Our trees robustly group the mycoplasma *Mesoplasma* with *Erysipelothrix* and *Coprobacillus* (both in order Erysipelotrichiales), but grouped another mollicute clade comprising *Acholeplasma* and *Haloplasma* with maximal support with *Turicibacter* instead. *Turicibacter sanguinis* is a non-flagellate, anaerobic, walled Gram-positive bacterium (Bosshard et al. 2002) having genes for (non-observed) sporogenesis (Cuiv et al. 2011), which previously was found to group with *Haloplasma* (neither methods nor tree shown) (Auchtung et al. 2016). On 16S rRNA trees, *Acholeplasma* and *Haloplasma* did not group together, though *Haloplasma* did group with *Turicibacter* and numerous environmental DNA lineages including mollicute *Candidatus* Izimaplasma (Skennerton et al. 2016). Our trees strongly show that Mollicutes are polyphyletic and evolved twice from different Bacilli by two independent wall losses; this was first found by Davis et al. (2013) but slightly less convincingly as the *Acholeplasma*/‘*Phytoplasma*’ clade was isolated and did not group with *Turicibacter*, a normal walled endosporogenous bacterium. On our ML trees, *Acholeplasma* (with much longer branch than *Haloplasma*) also failed to group with *Haloplasma*. Asserting Mollicutes to be monophyletic (Grosjean et al. 2014) was mistaken; their polyphyly needs to be recognised in future studies of their reductive evolution from Bacilli. A 34-RP ML tree showed that *Spiroplasma* is related to *Mycoplasma*, but that *Acholeplasma* and *Mycoplasma* form a separate clade which however did not group with *Turicibacter* (Davis et al. 2013). On our ML trees also *Acholeplasma* failed to group with *Turicibacter* (but support for that alternative is weak), whereas *Haloplasma* always did by both methods. We attribute these ML discrepancies to *Acholeplasma*-associated long-branch artefacts.

Yutin and Galperin (2013) found that the robust *Mesoplasma*/*Mycoplasma* clade was sister to *Erysipelothrix* plus *Clostridium ramosum* and *spiroforme*; they correctly believed both should be excluded from *Clostridium* (unfortunately their new genus *Erysipelatoclostridium* seems not yet validly published). That is entirely consistent with our trees, where *Acholeplasma* never groups with *Mesoplasma* or the *Erysipelothix*/*C. ramosum* subclade but was deeper; but as they did not include *Turicibacter*, they did not realise that Mollicutes evolved twice from two independent branches of the walled bacterial family, Erysipelotrichaceae. *Erysipelothrix* has distinctive murein peptidoglycan chemistry (Schubert and Fiedler 2001). Cladistically, therefore, mollicutes are secondarily simplified Bacillia and do not merit a separate class Mollicutes, which anyway would be polyphyletic. Still less do they deserve a separate phylum, which was first also called Mollicutes (Gibbons and Murray 1978), but later (confusingly) Tenericutes (Murray 1984). Separate phylum status was correctly strongly criticised by Davis et al. (2013). We urge that class Mollicutes and phylum Tenericutes be both abandoned and that Mycoplasmatales and Acholeplasmatales, their two oldest orders, are placed directly within a here broadened class Bacillia (see Taxonomic Appendix). Here, we group them with their ancestral (paraphyletic) order Erysipelotrichales as a new subclass Erysipelotrichidae embracing all three orders, which together form a strong clade on our RP trees and those of Davis et al. (2013); Erysipelotrichia Ludwig et al. 2010 was established as a class (Ludwig et al. 2009a) to contrast with another new class Bacilli (Ludwig et al. 2009a). However, it was then not appreciated how shallowly and robustly Erysipelotrichia nest within Bacilli, as shown by our trees and those of Davis et al. (2013). Excluding Erysipelotrichales from Bacilli and mollicutes from Erysipelotrichia and splitting the longest established endobacterial class Firmibacteria (=Teichobacteria Cavalier-Smith 2002) into separate classes Clostridia and Bacilli (Ludwig et al. 2009b) unwittingly simultaneously made three non-holophyletic classes, Clostridia, Bacilli, and Erysipelotrichia. Our decision now to abandon Erysipelotrichia and Mollicutes as classes eliminates one polyphyletic and two paraphyletic endobacterial classes, replacing them by one broadened holophyletic class, here renamed Bacillia to conform with rule 8 of ICNP, a change that also will prevent confusion with Bacilli excluding mollicutes. Class Clostridia remains non-holophyletic, but was recently made phenotypically more homogeneous by excluding Negativicutes (now called Selenomonadia) as a separate class (Marchandin et al. 2010). Despite rejecting class Mollicutes for formal taxonomy, we recommend retaining ‘mollicutes’ without capitals as a very useful vernacular term to refer to wall-free Endobacteria, an important polyphyletic grade of organisation for which a general term remains necessary. Discontinuing class Mollicutes also solves the problem that this name (like Bacilli here maintained informally for walled Bacillia) is not valid as it contravenes rule 8 of the current ICNP for classes (Parker et al. 2014).

Mollicute classification has been confused ever since they were put in separate order Mycoplasmatales (Freundt 1955). Most recently, five orders have been in use (Ruggiero et al. 2015). However, our trees and those of Davis et al. (2013), Gundersen et al. (1994), and Skennerton et al. (2016) suggest this is excessive as only three distinct mollicute clades are apparent. From these collectively, it is clear that *Ureaplasma* (sometimes placed in a separate order Ureaplasmatales, but not acccepted in Bergey’s Manual) and *Spiroplasma* (often segregated with *Mesoplasma* in a separate order Entomoplasmatales) belong in the same clade as *Mycoplasma* and that *Mycoplasma* is itself a polyphyletic genus. We therefore abandon Ureaplasmatales and Entomoplasmatales as separate orders, placing their genera and families all in Mycoplasmatales. That makes Mycoplasmatales a clade and solves the problem of demarcation between Mycoplasmatales and Ureaplasmatales. As our trees robustly show that *Haloplasma* is related to *Acholeplasma*, there was no justification for a separate order Haloplasmatales, here abandoned, formally transferring Haloplasmataceae Rainey et al. in Antones et al. 2016 to Acholeplasmatales. As *Anaeroplasma* is robustly related to *Acholeplasma* we also transfer it from Anaeroplasmatales and abandon Anaeroplasmatales. *Asteroplasma* formerly in Anaeroplasmatales is clearly not closely related to any other mollicutes, and likely represents a third independent loss of cell walls possibly from a deeper branching part of Bacillia rather than from Erysipelotrichales (see Davis et al. 2013; Gundersen et al. 1994) but it is premature to create a third mollicute order for it until genome data and site-heterogeneous multiprotein trees are available.

## New class Halanaerobiia

Halanaerobiales have an OM, unlike all other Clostridia left in the class after removing Selenomonadia. As that is contrary to the original definition of Clostridia, formed for endobacteria with Gram-positive walls and no OM, we establish a new class Halanaerobiia to segregate them from typical Clostridia with no OM (see Taxonomic Appendix). Together with the exclusion of Selenomonadia, this for the first time makes class Clostridiia (spelling here corrected) uniformly with only a single membrane by restricting it to orders Clostridiales, Thermoanaerobacterales, Heliobacteriales ord. n., and Sulfobacillales ord. n. The first two of these orders generally have thick murein walls as in Actinobacteria, whereas the others have thinner walls as in negibacteria. We argue that their thin walls and those of Halanaerobiia are the ancestral condition for Endobacteria and that the thicker walls of non-mollicute Bacillia and Clostridiales/Thermoanaerobacterales are secondarily derived independently of the thick walls of Actinobacteria. Thus Endobacteria now comprise two classes (Halanaerobiia, Selenomonadia) with typical negibacterial envelopes (OM and thin murein) and two classes (anaerobic Clostridiia, often aerobic Bacillia) without an OM but with murein that may be thick, thin, or absent. A thin-walled *Bacillus* mutant shows that even thick-walled endobacteria can exist in a thin-walled state and that a thin wall is present all around the prespore cell during sporulation (Tocheva et al. 2013). We suggest that endosporulation evolved in a thin-walled ancestral endobacterium similar to Halanaerobiia and that the same thin-walled sporulation mechanism persisted after OM losses and after polyphyletic secondary thickening yielding a thick-walled posibacterial state convergently with Actinobacteria.

This four-class classification better reflects endobacterial fundamental diversity in cell organisation than previously. We do not agree with Yutin and Galperin (2013) that Selenomonadia nesting within other endobacteria requires their suppression as a class. Their referring to the results of sequence trees and morphological contrasts being ‘contradictory’ is misleading. Both are informative about different aspects of evolution and can be reconciled with a judicious evolutionary classification as done here. The widespread Hennig-initiated prejudice against all paraphyletic taxa is evolutionarily illogical (Cavalier-Smith 1998b, 2010a) and should not be a barrier to retaining ancestral class Clostridiia—if they were truly paraphyletic rather than polyphyletic. Some ancestral groups are taxonomically unavoidable in a sensible taxonomy that aims to classifiy organisms according to both their common ancestry and phenotypic disparity, given that evolution created derived groups from sometimes radically different ancestral groups that still survive.

At first sight, the presence of two negibacterial and two posibacterial classes in the same phylum is confusing. How did evolution produce this mixture in which the two negibacterial clades do not group together but are separated by (probably more than two) posibacterial ones which also do not all group together? One possibility is that Selenomonadia got their OM by lateral gene transfer (LGT); Campbell et al. (2014) suggested from BLAST results that they may have got their OM-related genes by LGT from Proteobacteria. However, it is highly unlikely that a complex OM with necessary bridges from the cytoplasmic membrane and export machinery to enable LPS, lipid, and protein transport to the OM could have evolved in one step by LGT of scores of necessary proteins. More likely, the frequency of top hits to Proteobacteria is an artefact of the vast numbers of proteobacterial sequences in GenBank compared with those for Halanaerobiia, the most likely relatives on the standard assumption of vertical inheritance. It is much more likely that the halanaerobial OM is the ancestral condition for Endobacteria and OMs were independently lost by Clostridiia and Bacillia.

## Polyphyletic losses of the endobacterial outer membrane

The number of such evolutionary losses of the OM is not entirely clear as the relative branching order of clostridial orders, and with the clearly holophyletic Bacillia, is inconsistent on our CAT RP trees, e.g. one chain has Selenomonadia as sister to Bacillia, whereas the other shows Bacillales s. s. as their sister, both with maximal support. As there is another maximally supported contradiction within Clostridiia, whichever version of the tree were correct, we should have to postulate four separate losses. But if instead some hypothetical combined version of these trees were correct, one could reduce the number of losses to three or even two. The taxon-rich but site-homogeneous Bayesian tree of Kunisawa (2015) for Firmibacteria (i.e. excluding mollicutes) weakly makes Selenomonadia sister of Bacillia and has Clostridiia as an insignificantly supported clade. If it were correct only two losses would be necessary. Whether there were two, three, or (more likely) four OM (or even five if *Dethiobacter* has no OM) losses within Endobacteria, we must ask: why did it happen more than once in this phylum, given only two other inferred losses in the history of life (in Actinobacteria and, as argued below, independently in the neomuran ancestor)?

The answer we suggest lies in the unusual morphogenetic mechanism recently discovered for sporogenesis in Selenomonadia. Cryotomography of sporulating and germinating cells of the selenomonad *Acetonema longum* shows that during sporogenesis when the mother cell engulfs the prespore cell, only its inner cytoplasmic membrane (CM) grows around the prespore cell (Tocheva et al. 2011). Its growing lips whilst enwrapping the prespore cell pass round it within the peptidoglycan layer of the prespore. Being thus inside the OM of the prespore the growing CM lips therefore exclude the old prespore OM, which is not passed on directly to the daughter cell as it is in all non-endobacterial negibacteria. Instead, *Acetonema* loses the OM during every sporulation and a new OM is regenerated from the enwrappping mother cell CM during spore genermination. Thus, it remains true that the OM develops by growth and division of a preexisting membrane, in conformity with the universal principle *omnis membrano e membrano* (Blobel 1980). However, *Acetonema* provides a clear exception to the idea argued previously that all OMs, including those of mitochondria and chloroplasts, have arisen from preexisting OMs since the origin of life (Cavalier-Smith 1987b, c, d, 2004). We expect the same mechanism to be found in all endosporulating Selenomonadia and Halanaerobiia and predict that all negibacterial Endobacteria switch identity of the former mother cell CM to OM, some time after it enwraps the prespore murein but prior to the final stages of germination. This developmental identity switch from CM could be done by preexisting prespore bridge proteins and OM protein and lipid export machinery that has been separated from the old OM by the enwrapping mother cell CM. Topology of enwrapment generates the same OM topology before OM-specific molecules are inserted into it. Therefore, although this unique identity switch is an exception to the general rule for OM biogenesis, it adds support to the argument that membrane topology is often primary for membrane heredity, and chemical composition often secondary (Cavalier-Smith 2000, 2004).

The necessity for a CM-to-OM identity switch at every sporulation to maintain the OM into the next generation simply explains why Endobacteria is the only phylum that lost the OM more than once. Identity switching is a complex process, which like any complex mechanism cannot be perfect. It must sometimes fail through cross bridges and OM transporters not inserting properly or their insertion being so slow that a daughter cell without OM inserting molecules is generated. Sometimes such a developmental accident will survive and produce a viable endobacterium without an OM. The fact that several clades of endobacteria with no OM and only a thin murein layer exist means that they are not intrinsically non-viable. We therefore argue that four such losses in Endobacteria are much more likely than would be LGT to the ancestor of Selenomonadia. Establishing a LPS-containing OM by LGT would be so much more difficult; it almost certainly never happened in the entire history of life. After LGT, unlike in endobacterial CM-OM identity switching, a donor CM with properly assembled export and bridge complexes would not already be there, and a topologically correct OM would not already be present; even if LGT of scores of the requisite genes ever did occur (unlikely), it would almost certainly fail to make an OM morphogenetically. Too often, people underestimate the relative ease of multiple losses of complex characters than of their convergent gain. It is entirely wrong to estimate their probablity by parsimony counting of events. One must also evaluate event complexity to realistically guage their likelihood.

If our analysis is correct, Clostridiia are polyphyletic and ought eventually be subdivided into monophyletic units (whether holophyletic or paraphyletic), but that cannot be done sensibly until their internal branching order is more firmly established. For that, extremely taxon-rich Endobacteria trees with suitable outgroups and probably over 200 proteins may be necessary.

Though we currently accept OM loss only within Endobacteria and in the independent direct ancestors of Actinobacteria and neomura, we draw attention to the extremely thick Gram-positive murein wall of the chloroflexan *Thermobaculum terrenum* where micrographs are too poorly contrasted to reveal whether or not it has an OM like more typical Gram-negative chloroflexi (Botero et al. 2004). If it has an OM, the question arises how it gets its lipids and proteins across the thick wall. More likely, it and a minority of other chloroflexans are monoderm, some perhaps secondarily.

## Rooting the prokaryote tree within monoderm Endobacteria is evolutionarily implausible

Although there are strong reasons, especially those concerned with the origin of the eubacterial flagellar motors from OM proteins why the eubacterial tree must be rooted within negibacteria, there has been a longstanding assumption (dating back at least to the early ideas of Haldane and Oparin) that their ancestor was a simple anaerobic *Clostridium* or mycoplasma-like fermenting cell, so many have been reluctant to concede that the cenancestral eubacterium was so complex as to have had two membranes sandwiching a peptidoglycan wall. The evidence that mollicutes are secondarily simplified by multiple losses of the peptidoglycan wall is now overwhelming. Our arguments for a unique ease of OM loss by endobacteria make it highly probable that ancestors of monoderm endobacteria were generated by OM loss as Blobel (1980) first suggested and one of us repeatedly argued and assembled extensive evidence (Cavalier-Smith 1987b, c, 1991a, b, 1992b, 2001, 2002a, b, 2006a, c, 2010a). If one were to place the eubacterial root within monoderm Endobacteria, it would likely be within Clostridiia which are mostly anaerobic, not Bacillia that appear to be ancestrally aerobic. But wherever within Clostridiia it were placed, one would have to invoke polyphyletic origins of the LPS-containing OM, which we consider evolutionarily incredible. Previously even one origin of an LPS-containing OM direct from a monoderm posibacterium was judged an evolutionary highly unlikely transition, compared with the origin first of an OM of standard phospholipids followed later by the evolution of the extremely complex LPS biosynthesis. To invoke two such origins independently is entirely unreasonable. Assuming one followed rapidly by an LGT to make a second within Endobacteria relatively soon after the first is highly implausible.

## Polyphyly of classical Posibacteria

Until we realised the ease with which OM could be lost by endosporulating negibacteria as deduced from recent morphogenetic studies (Tocheva et al. 2011), it seemed unjustified to assume more than one loss of the OM unless phylogenetic evidence for Actinobacteria being unrelated to Endobacteria were stronger than it has been since some rDNA sequencers first supposed they were not directly related; no rDNA tree convincingly established eubacterial basal topology and some show Actinobacteria and Endobacteria as sisters (e.g. Mori et al. 2003). Therefore, all Posibacteria were argued to derive from a unimembanous common anestor (Cavalier-Smith 1987c, 2002a, 2006a, c) and Posibacteria have figured as a supposedly monophyletic eubacterial subkingom or infrakingdom in several prokaryote classifications (Cavalier-Smith 1989b, 1992b, 1998b, 2002a, 2006d; Ruggiero et al. 2015). Even some site-homogeneous multiprotein RP trees can group Endobacteria and Actinobacteria together as sisters (Lasek-Nesselquist and Gogarten 2013 fig. 8). But this never happened on our more accurate site-heterogeneous RP trees (the most taxon rich) or that of Lasek-Nesselquist and Gogarten (2013 fig. 5). However, Raymann et al. (2015) found a maximally supported Endobacteria/Actinobacteria clade in their less sampled two-domain Fig. 3 tree, but not with three-domains (their Fig. 5). Substantial agreement of our one- and two-domain trees, and their taxon-richness and the strong support for basal branching topology in our eubacteria-only tree give us enough confidence to now conclude that Actinobacteria and Endobacteria are most likely not sisters. Endobacteria are maximally supported as sister of Neonegibacteria, whereas Actinobacteria are near maximally supported as sisters of Endobacteria plus Neonegibacteria. That implies that Actinobacteria are somewhat older than Endobacteria (if rooting on Chloroflexi is correct) and that ancestral actinobacteria lost the OM and became monoderm before any Endobacteria did so. Furthermore, our strong demonstration that even within Endobacteria there have probably been about four OM losses means that we must accept polyphyly of monoderm Posibacteria; we must either cease to use it as a taxon or modify the concept of posibacteria to include diderm Endobacteria as did Ruggiero et al. (2015). But it now makes no sense to include Chloroflexi under the term Posibacteria as was done by (Cavalier-Smith 2014) and Ruggiero et al. (2015).

Abandoning Posibacteria as a phylum name entails raising its former subphyla, Endobacteria and Actinobacteria, each a maximally supported clade, to phylum rank (Taxonomic Appendix). As the introduction explained, Firmicutes was invented to embrace Actinobacteria and exclude mollicutes, but is now used in two contradictory senses, which is very confusing, as also is the fact that in neither sense does it refer to a unique ancestral shared character. By contrast Endobacteria as here emended refers to the ancestral character that first distinguished the phylum from all other prokaryotes, making it more distinctive and a better unambiguous name for the phylum than Firmicutes, even though endospores were secondarily lost by some included lineages.

If posibacteria are not a clade, we must explain how Actinobacteria and Bacillia/Clostridiales both share teichoic acids and a sortase for making lipoproteins unknown in any strictly negibacterial phyla but important for their shared thick wall structure. A possibility we favour is that both arose in their common ancestor after it diverged from its Melainabacteria/Cyanobacteria sister clade and were lost in the common ancestor of neonegibacteria. As both were lost at least twice in mycoplasmas, loss is possible. We searched for the teichoic acid synthesising protein TagB in GenBank and found it appears non-universal in Actinobacteria and Endobacteria so either losses occurred in both or teichoic acids orginated in one and key enzymes moved to the other by LGT. Teichoic acids appear general in Bacillia except mycoplasmas, Clostridiales sensu stricto and in most but not all of Selenomonadia, but seem absent in the two deepest endobacterial branches and rare in the next two deepest, suggesting frequent losses rather than LGT. It is now confirmed that a variety of Actinobacteria can make teichoic acids (Colagiorgi et al. 2015). Key synthetic glycosylases like TagB and TagF from Actinobacteria and Endobacteria are mutually more closely related than they are with more distant glycosylases in negibacterial phyla. That teichoic acids can exist in Selenomonadia shows that they are compatible with negibacterial envelopes so could have evolved before Actinobacteria and Endobacteria lost the OM, making them a preadaption for wall thickening, which can be regarded as parallel evolution from similar related ancestors rather than pure convergence. The same may be true of sortases. Because the common ancestor of Actinobacteria and Endobacteria probably had teichoic acid and one sortase, and these are successive branches on the tree we can regard these two phyla collectively as a monophyletic group characterised by the origin of these two wall properties and retain paraphyletic subkingdom Posibacteria, so long as we exclude Chloroflexi (unlike Ruggiero et al. 2015). If however Posibacteria were a clade as on Fig. 5 of Raymann et al. (2015), this would explain their unique sharing of sortases and teichoic acids. This possibility ought to be tested further by 200-300-protein site-heterogeneous eubacterial trees.

Though we consider it no longer useful to use Negibacteria as a taxon, negibacteria remains useful as the best vernacular term to refer collectively to all eubacteria with a porin-containing OM, irrespective of whether it contains LPS (most phyla) or not (all Chloroflexi; some Synthermota, some Hadobacteria, some spirochaetes, some Proteobacteria). The old term Gram-negative bacteria is not useful in this way and best reserved for the empirical results of Gram staining; as noted above, some bacteria that stain Gram-negatively are actually posibacteria without OM (e.g. mollicutes) and some that stain positively are actually negibacteria, e.g. *Deinococcus*. For clarity, it remains essential to maintain the subtle and too often ignored distinction between negibacteria (based on ultrastucture) and Gram-negative bacteria, and posibacteria (based on ultrastructure) and Gram-positive bacteria. Gram-negativity or positivity descriptors of empirical staining for light microscopy are not equivalent to the ultrastructurally defined terms posibacteria and negibacteria, which were never synonyms for the older terms. Gram staining is useful as an ancillary method for identification but not for large-scale taxonomy, unlike ultrastructure. Using Posibacteria now to include Gram-negative endobacteria makes it even less likely to be confused as a synonym with Gram-positive.

## New phylum Aquithermota

The second edition of *Bergey's Manual* established new order Aquificales and class and phylum Aquificae for highly thermophilic negibacterial chemolithoautotrophs related to the hyperthermophile *Aquifex*. It also established a new phylum and class Thermodesulfobacteria for another new order (Thermodesulfobacteriales) then containing only *Thermodesulfobacterium* (now including *Thermodesulfatator* on our trees and four additional genera), a thermophilic negibacterial heterotrophic sulphate reducer ultrastructurally similar to Aquificales. It is curious that two separate phyla are still retained for such similar thermophiles especially now that sulphate reduction is known in Aquificales and there are several genera of chemoautotrophic Thermodesulfobacteriales, and the latter can group strongly with Aquificales rather than with Thermotogia, Hadobacteria, or Chloroflexi on 16S rDNA trees. As our taxon-rich CAT RP trees invariably place class Aquificia (spelling corrected to conform with ICNP), including *Thermosulfidibacter* whose inclusion is strongly supported despite being questioned by Gupta and Lali (2013), as sister to class Thermodesulfobacteriia (spelling here corrected to conform with ICNP) with maximal support, there is no reason to keep separate phyla for these classes. We therefore establish a new phylum Aquithermota (see Taxonomic Appendix) to group both classes together and establish order Thermosulfidibacterales for *Thermosulfidibacter* as its previous inclusion in Aquificales made the order as emended by Gupta and Lali (2013) polyphyletic and transferring it to physiologically more similar Thermodesulfobacteriia would have made them paraphyletic. Aquificia now has three orders and 16 genera; Thermodesulfobacteriales just 6 genera. Thus, Aquithermota have four orders and 22 genera. As they are remarkably homogeneous ultrastructurally and physiologically and certainly a clade, there can be no justification for splitting them into two or more phyla. From our RP phylogeny, we deduce Aquithermota were ancestrally anaerobic thermophiles, with hyperthermophilic microaerophilic Aquificales a derived clade.

It has long been controversial whether Aquificales are more closely related to Thermotogia (here corrected spelling for Thermotogae) or to Proteobacteria. Our trees show decisively that they are not specifically related to either. Instead, Aquithermota are maximally supported by CAT RP trees as sister to infrakingdom Gracilicutes, which includes Proteobacteria, Spirochaete, Planctobacteria, and Sphingobacteria and therefore, Aquificia are no more closely related to Proteobacteria than are the other three gracilicute phyla. Putting Aquificales in Proteobacteria (Cavalier-Smith 2002a, 2006c) was incorrect. This firm position implies that the 4-amino insertion shared by *Aquifex* and all Gracilicutes except Spirochaetae (Cavalier-Smith 2002a) was an ancestral character of clade Aquithermota/Gracilicutes lost secondarily by ancestral spirochaetes, which illustrates the hazard of using single indels alone to group phyla. Thermotogia, which lack that insertion, are robustly phylogenetically more distant, grouping with other thermophiles. Phylogenetic unity and likely ancestral thermophily of Aquithermota is suggested by both its major branches having reverse DNA gyrase just as do Archaebacteria (Brochier-Armanet and Forterre 2007). Concordantly with Boussau et al. (2008a), the common ancestor of Aquithermota and Thermotogia was not a hyperthermophile. If Fig. 5 is correct, it may not even have been a thermophile—unless neonegibacteria were ancestrally thermophilic and mesophily evolved repeatedly secondarily.

## New phylum Synthermota

Thermotogae also were made a separate phylum in Bergey’s 2nd Edition just because they do not group reliably with other clades on rDNA trees. However, a 44-protein neighbour joining tree (Nishida et al. 2011) showed that they group strongly with three other thermophilic negibacterial groups: (1) anaerobic hyperthermophilic *Dictyoglomus* (non-motile chemoorganotrophs of class Dictyoglomia (Patel 2011) often treated as separate phylum Dictyoglomi); (2) thermophilic proteolytic fermenter *Coprothermobacter*, usually misclassified in Clostridia but recently put in new class Coprothermobacteria and phylum Coprothermobacteriota (Pavan et al. 2018); and (3) more distantly with class Synergistia comprising a mostly mesophilic family of amino acid digesters (also often treated as a separate phylum (Jumas-Bilak et al. 2009)). Our CAT trees strongly confirm that grouping to be a clade, and show that it also includes *Caldisericum*, an anaerobic sulphur-compound respirer recently put in class Caldisericia and phylum Caldiserica merely because of divergence on a crude 16S rDNA tree (Mori et al. 2009) as well as *Thermodesulfobium*, moderately thermophilic chemoautotrophic negibacterial respirers (Mori et al. 2003) currently misclassified in Thermoanaerobacterales in Clostridia. Thus, five groups related as a robust clade on eubacteria-only site-heterogeneous RP trees have been unnecessarily treated as separate phyla merely because of poor resolution of single-gene rDNA trees. Given much greater resolution attainable with RP multiprotein trees, separating them into five phyla was premature. We now group all five ‘phyla’ plus Thermodesulfobiaceae as one new negibacterial phylum Synthermota divided into two new subphyla, Synergistetes and Thermocalda, each maximally supported as clades on RP CAT trees. Our trees clearly show that contrary to Nishida et al. (2011) and Cavalier-Smith (2002a), they are not specifically related to Endobacteria, nor to Fusobacteria (Cavalier-Smith 2006d). Instead Synthermota branch with maximal support one node above Endobacteria as sister to all other Neonegibacteria. Virtually, all but *Thermodesulfobium* catabolise amino acids unlike the largely autotrophic Aquithermota.

Largely non-thermophilic Synergistetes has only class Synergistia with LPS biosynthetic enzymes related to those of *Dictoglomus* and ‘Atribacteria’ (Antunes et al. 2016; Sutcliffe 2010), whereas Thermocalda includes four former classes: Thermotogia, sufficiently distinct in their sheath-like toga partially separated from CM by a very wide periplasmic space and loss of LPS to retain class rank (now with three orders: Bhandari and Gupta 2014); Dictyoglomia, also morphologicaly distinct enough to merit class rank; plus Coprothermobacteria and Caldisericia. But Coprothermobacteria, Caldisericia, and Thermodesulfobiaceae are not mutually distinctive enough in morphology, physiology, or chemistry to be separate classes, and invariably form a strongly supported clade on RP trees; therefore, we merge all three into class Caldisericia, chosen as having the shortest name most appropriately descriptive of this robust thermophilic clade (it is also the oldest established of these classes, though ICNP does not require retention of the oldest class when merging them as it does for orders). These three groups all have relatively normal negibacterial cell envelope morphology, unlike Thermotogia and Dictyoglomia; Antunes et al. (2016) found no LPS enzymes in *Caldisericum* and the typically weak OM staining in this broadened Caldisericia makes it possible that LPS is absent. Coprothermobacterales (one family, one genus, two species), Caldisericales (one family, genus, species), and new order Thermodesulfobiales (in Caldisericia: see Taxonomic Appendix) are sufficiently highly ranked as orders. One does not need a phylum for each genus! *Caldisericum* is unusual in having an obvious cortical ribosome-free layer inside its CM which suggests a novel submembrane skeleton, so merits separate ordinal rank to reflect this uniqueness (Mori et al. 2009), which also emphasises the distinctiveness of cell envelopes in all Thermocalda.

Making these few quite similar species four classes or phyla greatly overrates their distinctiveness and unnecessarily complicates classification which should be kept as simple as is practicable and phylogenetically sound. The purpose of classification is to simplify biodiversity so that we can readily grasp it intellectually. *Candidatus* Cryosericum, sister to *Caldisericum*, has ridiculously been proposed as a new phylum purely because of sequence divergence (Martinez et al. 2018), but is important in showing that not all Thermocalda are thermophiles. When making Synergistetes a phylum, Jumas-Bilak et al. (2009) suggested that ‘a [eu]bacterial phylum is formed to accommodate a group of bacteria that cannot be aggregated to any taxon except *Bacteria*’; clearly that does not apply to Synergistia, and even more strongly not to any other taxa here aggregated as phylum Synthermota, which all have fundamental similarities in envelope organisation, making them distinct from Aquithermota and Gracilicutes. One of us long ago criticised the widespread practice of making phyla or ‘candidate phyla’ merely because of the low resolution of 16S rDNA trees and predicted that most candidate divisions, especially the thermophilic ones of Hugenholtz et al. (1998a, b) ‘when studied by good multiple-protein trees, will turn out to belong’ to already known phyla (Cavalier-Smith 2002a p. 67). This prediction is now fully borne out: only one of those candidate phyla turned out to be justifiably separate: Armatimonadetes. The others all group with previously known phyla on our trees.

Dictyoglomales, despite currently having only one genus and two closely related named species, are unique in having an OM well separated from the murein wall and CM by prominent hexagonally arrayed pegs (~ 80 nm long) (Hoppert et al. 2012). These pegs may be related to the 49 nm OMP-α rods that span the periplasmic space of Thermotogia (Lupas et al. 1995); unlike Thermotogales other than *Fervidobacterium* (Huber et al. 1990), *Dictyoglomus* is most unusual in being able to make giant ‘rotund bodies’ by repeatedly dividing their CM/murein without dividing their OM and additionally makes intermediary spindle-shaped assemblies of numerous protoplasts within a single membrane (Hoppert et al. 2012). Rotund bodies were first reported in the hadobacterium *Thermus* whose OM is also separated from murein by a wide clear periplasmic space across which fine bridges are visible (Brock and Edwards 1970), which we suggest may be distant homologues of OMP-α as some appear to have globular heads at the murein end like OMP-α. Brock and Edwards (1970) assumed that rotund bodies form by OM fusion of separate cells, but retention of daughter protoplast/murein rods within a single OM, as apparently generates the *Dictyoglomus* spindle-shaped assemblies, seems more likely to be a shared mechanism for both Thermocalda and Hadobacteria. Ability to make rotund bodies is deep-seated in Hadobacteria as phylogenetically distant *Oceanithermus* have them (Mori et al. 2004). But, as Hadobacteria are not sisters of Synthermota and rotund bodies have not been seen in Synergistia, it is unclear whether ability for partial disassociation of OM and murein, necessary for making them, evolved separately in these two phyla or reflects an ancestral mechanism in the common ancestor (i.e. the ancestral neonegibacterium), e.g. simple mutual attachment dependent only on Omp-α. Some Synergistia have close attachment of murein to the OM, e.g. *Acetomicrobium*, formerly *Anaerobaculum*, *mobile* (Magot et al. 1997), but *Dethiosulfovibrio* has a wider space in which thin rod-like bridges like Omp-α are visible (Magot et al. 1997), suggesting such simple coiled-coil bridges may be ancestral for neonegibacteria. *Acetomicrobium* (=*Anaerobaculum*) *thermoterrenum* grown on complex medium makes terminal sheaths bulging away from the protoplast (Rees et al. 1997) similarly to *Thermotoga*, suggesting that a structurally based potential for local separation of OM from murein may be ancestral for Synthermota as well as Hadobacteria. Possibly, more complex and varied attachments evolved after Hadobacteria and Synthermota diverged from the common ancestor of Fusobacteria (which have typically gracilicute-like envelopes), Aquithermota and Gracilicutes, giving them more consistently closely attached OM: the gracilicute *Bacteroides* seems to have both single and double bridges between OM and murein (Ushijima 1967).

Greatly differing envelope properties of Synthermota (nearly always organotrophs, rarely chemoautotrophs) and Aquithermota (nearly always chemoautotrophs, rarely heterotrophs) is consistent with being separate non-sister phyla. Synthermota may not have been ancestrally thermophilic, unlike Aquithermota. In agreement with that, a reverse gyrase tree suggests that *Dictyoglomus* got reverse gyrase laterally from Aquificales, whereas that of *Thermotoga* is less closely related (Brochier-Armanet and Forterre 2007). Given the Fig. 5 phylogeny, it appears that LPS was lost separately in Thermotogales and Caldisericia (Antunes et al. 2016); however, though core LPS-making enzymes were not identified in *Caldisericum*, 3-OH fatty acids suggested possible presence of LPS (Mori et al. 2009).

## Eubacterial origins of hyperthermophily

It was once supposed that hyperthermophily arose in the ancestral archaebacterium and hyperthermophilic eubacteria such as Thermotogales and Aquificales evolved later by acquiring hyperthermophilic enzymes from them by LGT (Forterre et al. 2000). But evolution of reverse DNA gyrase, the most characteristic marker for hyperthermophily, does not support that; there is a single weakly supported bipartition between eubacterial and archaebacterial subtrees with no evidence for LGT between them (Brochier-Armanet and Forterre 2007; Campbell et al. 2009). As reverse gyrase is a chimaera of a eubacterial DNA helicase and eubacterial type of DNA topoisomerase I (Forterre 1996), it provided one of the strongest early proofs that archaebacteria are evolutionarily younger than and evolved from eubacteria (Cavalier-Smith 2002a). In agreement with that, a reverse gyrase distance tree rooted on topoisomerases places the archaebacterial clade within the eubacterial sequences with 66% support (Campbell et al. 2009). As the most divergent reverse gyrase sequences are from Aquithermota, which as noted above ancestrally had this enzyme, we argue that hyperthermophily and reverse gyrase most likely first evolved in stem Aquithermota and were acquired by the ancestral archaebacterium and other hyperthermophilic eubacteria by independent LGTs. The archaebacterial tree is consistent with vertical inheritance within archaebacteria (Brochier-Armanet and Forterre 2007), but the eubacterial reverse gyrase tree does not fit the eubacterial tree deduced here from RPs, arguing against vertical inheritance coupled with numerous losses—especially within Endobacteria and Proteobacteria. Contrary to RP trees, sequences of reverse gyrases of Thermotogales, ε-proteobacteria, *Thermus* (Hadobacteria), and hyperthermophilic endobacteria all nest within those of Aquithermota (Brochier-Armanet and Forterre 2007; Campbell et al. 2009). All Aquithermota on our trees have reverse gyrase (as do numerous other genera), whereas in Thermocalda *Thermodesulfobium* and *Coprothermobacter* lack it, but it is present in *Dictyoglomus*, *Caldisericum* and Thermotogales. As it is absent from most non-thermophilic Synergistia, Synthermota were probably not ancestrally thermophilic, in marked contrast to Aquithermota. The two deeply divergent paralogues in *Aquifex* are consistent with reverse gyrase having originated in ancestral Aquithermota prior to the origin of archaebacteria; two separate paralogues evolved independently in early crenarchaeotes (Brochier-Armanet and Forterre 2007). The *Thermus* sequence (on a plasmid, consistently with LGT) is related to the same *Aquifex* paralogue as that from *Dictyoglomus*, which is not closely related to those of *Thermotoga* (Brochier-Armanet and Forterre 2007), suggesting multiple LGTs to Thermocalda from Aquithermota. Not all LGT need have been directly from Aquithermota to other targets. The close relationship of *Thermotoga* and endobacterial sequences makes it likely that LGT also occurred directly between these two phyla. Taxon-richer trees might better establish the number and direction of eubacterial LGTs.

It might be argued that if neomura evolved from Aquithermota/Thermocalda as suggested by some three-domain trees (CAT only, e.g. Fig. S13; not ML, Fig. S14), but not by two-domain trees, reverse gyrase might have been acquired vertically by archaebacteria (rather than by LGT as we propose). However, as explained above, three-domain trees are much more distorted compared with single-domain trees than are two-domain trees and therefore likely less trustworthy than two-domain trees, so we judge that both the grouping of neomura with Aquithermota/Thermocalda and placement of Aquithermota within Synthermota as sister to Thermocalda on some CAT three-domain trees are likely LBA artefacts. Alternative grouping of neomura with Planctochlora (Planctobacteria, Sphingobacteria) on two-domain trees is technically more credible and strongly supported by many independent lines of evidence discussed in detail below for a likely genuine evolutionary relationship between eukaryotes and Planctobacteria in particular. This relationship clarifies greatly numerous previously poorly understood aspects of eukaryogenesis as well as origins of archaebacteria. Before treating this major evolutionary question, we briefly discuss the composition of Planctochlora and Proteobacteria and in somewhat more detail implications of our trees for the eubacterial evolution of photosynthesis.

## Phylum Planctobacteria broadened by adding Elusimicrobia

Phylum Elusimicrobia recently established for tiny deeply branching fermentative negibacteria now includes just two genera (*Elusimicrobium* and *Endomicrobium* from animal guts) assigned respectively to classes Elusimicrobia and Endomicrobia (Geissinger et al. 2009; Herlemann et al. 2009; Zheng and Brune 2015; Zheng et al. 2016). Their affinity was previously unclear as a 22-protein ML tree weakly grouped them with Synergistia (Herlemann et al. 2009) whereas a 31-protein ML analysis including both genera put them as sister to spirochaetes; neither analysis reported bootstrap support nor used a site-heterogeneous algorithm. Our CAT analyses strongly show that Elusimicrobia are sister to Planctobacteria, the phylum initially established for free-living planctomycetes and intracellular parasitic *Chlamydia* on the assumption that both lacked murein (Cavalier-Smith 1987b, 1998b). Later the related Verrucomicrobia possessing murein were added to Planctobacteria (Cavalier-Smith 2002a) and it was discovered that both planctomycetes and *Chlamydia* have peptidoglycan remnants also (Pilhofer et al. 2008), planctomycetes and *Protochlamydia* complete sacculi but Chlamydiaceae only a ring at the division septum (Rivas-Marin et al. 2016). Although everyone accepts that these three groups are related, others have ranked each as separate phyla: Wagner and Horn (2006) grouped them with three other claimed phyla (Lentisphaerae (Cho et al. 2004), which ought to have been made a class within Planctobacteria sensu Cavalier-Smith 2002a; ‘Poribacteria’, and Omnitrophica (=OP3) as the ‘PVC superphylum’, compositionally equivalent to phylum Planctobacteria, apparently ignorant of its earlier establishment.

Superphylum rank was pointless taxonomic inflation that disregards their unifying characters, so we continue to treat Planctobacteria as a phylum. Uniquely in the living world, Planctobacteria share a small RNA-binding protein (sRp) of similar folding pattern to ribosomal L30, which uniquely is absent from Planctobacteria, so the unique protein may therefore be a group-specific substitute (Lagkouvardos et al. 2014); Gupta et al. (2012) found the same protein throughout Planctobacteria except Poribacteria, which did not group with other PVCs on their 16-protein tree so they questioned their inclusion in the group. An 83-protein tree for Gracilicutes weakly grouped ‘Poribacteria’ with *Candidatus* Hydrogenedentes, this clade being sister to Elusimicrobia plus *Candidatus* Aerophobetes (Kamke et al. 2014), these four groups being sister to ‘core PVC’. That well-sampled tree therefore supports Elusimicrobia and Poribacteria being part of the sister clade to core PVCs. BLAST revealed two sRp-homologues in Aerophobetes but none in Elusimicrobia. We found no convincing evidence by BLAST of L30 in any of them, the few strong hits in *Chlamydia*s, and single one to Omnitrophica most likely being contamination or LGT from other eubacterial phyla. This is consistent with Elusimicrobia and these three environmental groups (too highly ranked as ‘phyla’) being sister to classical Planctobacteria, as our RP trees robustly show for *Elusimicrobium*, so we now include them all within Planctobacteria as new subphylum Elusimicrobia, and establish subphylum Euplancta with five classes to embrace classical Planctobacteria. ‘Phylum Kiritimatiellaeota’ (Spring et al. 2016) originally within Verrucomicrobia would have been more judiciously ranked as subclass; we rank Verrucomicrobiia as only a class together with two others within new infraphylum Opitutae (Table 3). By reducing the rank of Elusimicrobia to subphylum within phylum Planctobacteria, instead of 9 separate phyla as before, we now have just one: broadened Planctobacteria—a single robust clade on RP trees. This clade shares the unique propensity of often having a partially or greatly swollen periplasm through loosening attachment of murein to the CM (or loss of the sacculus altogether except at the septum). L30 appears to have been lost or replaced by sRp in their common ancestor, though sRp may not be present (or too divergent to recognise) in all subphylum Elusimicrobia.

**Table 3:** Revised Classification of Phylum Planctobacteria Cavalier-Smith 1987 em.

**Subphylum 1. Elusimicrobia** Cavalier-Smith subphyl. n.	
**Class Elusimicrobia** Cavalier-Smith cl. n.	
Order Elusimicrobiales Geissinger *et al.* 2010	
**Subphylum 2. Euplancta** Cavalier-Smith subphyl. n. (=‘PVC group’)	
**Infraphylum 1. Planctomycetia** Cavalier-Smith infraphyl. n.	
**Class Planctomycetia** Cavalier-Smith cl. n. (former Planctomycetes)	
Order Planctomycetales Schlesner and Stackebrandt 1987	
Order Phycisphaerale*s* Fukunaga et al. 2010	
**Infraphylum 2. Chlamydiia** Cavalier-Smith infraphyl. n.	
**Class Chlamydiia** Horn 2016	
Order Chlamydiales Storz and Page 1971	
**Infraphylum 3. Opitutae** Cavalier-Smith infraphyl. n.	
**Class 1. Verrucomicrobiia** Cavalier-Smith cl. n.	
Order Verrucomicrobiales Ward-Rainey et al. 1996	
**Class 2. Lentisphaeria** Choo et al. 2012	
Order Lentisphaerales Choo et al. 2004	
**Class 3. Opitutia** Cavalier-Smith cl. n.	
Order Opitutales Choo et al. 2007	

It is completely unjustified to treat these seven orders as seven phyla as is often done

From recent cryotomographic studies of planctobacteria with sacculi, we argue that, in marked contrast to Synthermota where the OM tends to balloon away from the murein layer to form a sheath, in Planctobacteria, the often-inflated periplasm stems from greater weakness of the bridges between murein and the CM. Thus, the often swollen periplasm is not homologous in the two phyla: in Synthermota, the bridges between murein and OM are the structurally weaker link, whereas in Planctobacteria, it is bridges between murein and CM that are often broken in evolution. This consistent difference between the two phyla fits earlier arguments that differences in cell envelope organisation are key aspects of eubacterial evolution that merit great weight in higher classification (Cavalier-Smith 2002a). Weakness of the CM/murein bridges probably extends back to the planctobacterial ancestor as the periplasm is irregularly widened in *Elusimicrobium* (Geissinger et al. 2009) and especially *Endomicrobium* (Zheng et al. 2016) unlike the regular narrow state in Proteobacteria. Weak CM/murein links could have predisposed planctobacteria to their multiple losses of the sacculus and also helped the simultaneous losses of murein and the OM during the likely origin of neomura from planctobacteria as a later section explains. Like Planctomycetes, Elusimicrobia have a cell cycle involving budding, otherwise rare in eubacteria.

Our trees robustly group class Verrucomicrobiia and *Lentisphaera* as sisters; their joint clade (here new infraphylum Opitutae) is robustly sister to *Chlamydia* (here in new infraphylum Chlamydiia). Table 3 summarises the new planctobacterial classification. On rDNA trees ‘*Candidatus* Omnitrophica’ (misuse of the term *Candidatus* that properly refers only to prospective species (Parker et al. 2014)) is sister to Verrucomicrobia (Spring et al. 2016); whether it should be a subclass of Verrucomicrobia or a third class of Opitutae will depend on its phenotype and multiprotein CAT trees—but it should certainly not be a phylum.

## Phylum Sphingobacteria broadened by adding Gemmatimonadetes

Sphingobacteria was the phylum name given to unite the Bacteroidetes/*Flavobacterium* clade, many of which have sphingolipids, and Chlorobiales (also with sphingolipids) assuming sphingolipids, gliding motility, and absence (as then thought) of flagella were shared ancestral characters (Cavalier-Smith 1987b), and formally made a phylum with classes Flavobacteria (including Bacteroidetes and *Fibrobacter*) and Chlorobea (Cavalier-Smith 1992b). The class names were eventually validly published (Cavalier-Smith 2002a), but later seemingly arbitrarily rejected (Tindall 2014); following Garrity et al. (2005) who invalidly split Flavobacteria into three classes (one confusingly called Sphingobacteria), most authors treat these bacteria as separate phyla Chlorobi and Bacteroidetes (Krieg et al. 2011b) even though they group together on rDNA trees. Our CAT and ML trees all group Chlorobi and Bacteroidetes as sisters with maximal support. The idea of a common ancestry was further substantiated by shared indels, some shared with class Fibrobacteriia, so all three were agreed to have a common ancestry and designated the FCB group (oddly ignoring then valid phylum Sphingobacteria) which was strongly holophyletic on an RNA polymerase C tree (Gupta 2004). Later, proteins uniquely shared by FCB taxa were identified (Gupta and Lorenzini 2007). Our RP trees do not have an FCB clade. Instead CAT trees strongly put Gemmatimonadales as sister to Chlorobi/Bacteroidetes whereas ML puts Gemmatimonadales (ranked too highly as ‘phylum’ Gemmatimonadetes: Zhang et al. 2003) weakly as sister to Fibrobacteriia. Thus, both strongly place Gemmatimonadales *within* FCB, so FCB alone is not a clade. However, both methods very strongly support holophyly of an FGCB group that also includes Gemmatimonadales.

As this group is extremely robust and clearly one of the four major clades within superphylum Gracilicutes, we redefine phylum Sphingobacteria to include not only Chlorobi/Bacteroidetes (each placed in new infraphyla within new subphylum Chlorobia) and Fibrobacterales (in new subphylum Fibrobacteria, a small reduction in rank) but also Gemmatimonadales (in new subphylum Gemmatimonadetes; now invalid as a class name) (see Table 2). Sphingobacteria as thus revised is congruent with RP trees and does not overrank its subgroups as they are if treated as four separate phyla. We argue below that the novel form of anaerobic photosynthesis in *Gemmatimonas* without chlorosomes (Dachev et al. 2017) arose following LGT of photosynthetic genes from Proteobacteria and is the most convincing example of such transfer. Fibrobacterales are non-flagellate so probably ancestrally lost flagella, but flagellar genes are present in deep-branching rhodothermian bacteroidetes *Rhodothermus* and *Salinibacter*, in Gemmatimonadales and Ignavibacteriia (*Ignavibacterium*, *Melioribacter*), and NCIL-2 from the thermophilic clade OPB56, so it is likely that the main bacteroidete subclade (new superclass Bacteroidia) and Chlorobiales lost flagella separately. Contrary to probably misrooted early rRNA trees, Ignavibacteriia are sister to OPB56 not to Bacteroidetes and this joint clade robustly sister to Chlorobea (Hiras et al. 2016), a now rejected class name replaced here by Chlorobiia to conform with the rules. OPB56 should therefore be placed in Ignavibacteriia, a here thus broadened class, which clearly belongs in Chlorobi, which deserves no higher rank than infraphylum (as here) or superclass—making Ignavibacteriae a separate phylum (Podosokorskaya et al. 2013) was unjustified rank inflation.

The ML 83-protein tree of Kamke et al. (2014) and 43-protein tree (Rinke et al. 2013) both had Gemmatimonadetes within FCB as insignificantly/weakly supported sister to *Fibrobacter* as in our ML trees and both strongly suggest that environmental DNA groups ‘Marinimicrobia’, ‘Latescibacteria’, ‘Cloacimonetes’ (excessively highly ranked as phyla) also should be put in Sphingobacteria; that is clearest for the first two, which branch within Sphingobacteria, but less so for Cloacimonetes that is their sister. Both strongly support clade Sphingobacteria as here emended (Taxonomic supplement). More richly sampled CAT trees are needed to check whether the alternative topology of our CAT trees is correct as we predict it will be. Though the 38-protein tree (Rinke et al. 2013) failed to resolve any deep relationships between our 14 phyla (or even show monophyly of Proteobacteria as here defined), the 83-protein tree strongly supports now-broadened Sphingobacteria and Planctobacteria being sisters (i.e. clade Planctochlora). Although most Sphingobacteria have closely parallel OM and CM (e.g. *Bacteroidetes* (Ushijima 1967), *Ignavibacterium* (Iino et al. 2010), like Proteobacteria) and no inflated periplasm, *Gemmatimonas*, has patches of inflated periplasm where the murein layer (incorrectly labelled plasma membrane on their Fig. 1b) more widely separates from the CM (Zeng et al. 2015); was this propensity present even in the common ancestor of Planctochlora and lost by other Sphingobacteria independently of Proteobacteria?

Krieg et al. (2011a) established phylum Bacteroidetes with three new classes: Bacteroidia, Cytophagia, and Sphingobacteriia. However, discoveries of closer phenotypic similarities between infraphyla Chlorobi and Bacteroidetes than previously supposed and the intermediate character of NCIL-2 (Hiras et al. 2016) make earlier inclusion of both in the single phylum Sphingobacteria, with *Fibrobacter*, taxonomically superior. All multiprotein trees strongly support inclusion of *Fibrobacter* and *Gemmatimonas* in the same phylum as Chlorobia (i.e. the invariably robust Chlorobi/Bacteroidetes clade).

We also add to Sphingobacteria the heterotrophic flagellate Calditrichales (*Caldithrix* and *Calorithrix*) as new subphylum Calditrichae, as rDNA and protein trees show they are sister to Chlorobia (Kublanov et al. 2017; Kompantseva et al. 2017). Separate phylum Calditrichaeota (Kublanov et al. 2017) was unnecessary and exaggerates their distinctiveness.

## Proteobacteria comprise subphyla Rhodobacteria, Acidobacteria, and Geobacteria

In an earlier classification recognising only seven eubacterial phyla not 14 as here, Cavalier-Smith (2002a) considered that Proteobacteria to be monophyletic must include many negibacterial groups not previously assigned to that phylum, and therefore subdivided Proteobacteria into three subphyla (Rhodobacteria, Geobacteria, Thiobacteria) so as to include them. This broadened view of Proteobacteria comprising all predominantly gracilicute groups with uniformly narrow periplasm that ancestrally had external flagella (not periplasmic ones like spirochaetes) did not become widely accepted as 16S rDNA lacked the resolution to confirm or refute it. Accordingly, others later made three conjecturally proteobacterial lineages separate rDNA-defined phyla: Deferribacteres, Chrysiogenetes, Acidobacteria. Our RP trees now confirm that all three must be included in Proteobacteria if the phylum is to be monophyletic. This is so because ε-proteobacteria are so phylogenetically distant from the other nominal proteobacteria, being sister to Chrysiogenales plus Deferribacterales, whereas Acidobacteria are sisters to α-δ-proteobacteria. Cavalier-Smith had grouped ε- and δ-proteobacteria together as Thiobacteria. As our trees confirm earlier evidence that it is not a clade, we now abandon suphylum Thiobacteria and transfer δ-proteobacteria (as new class Myxococcia) to suphylum Rhodobacteria and ε-proteobacteria (as new class Nautiliia) to revised subphylum Geobacteria.

Our trees are fully concordant with a recent 98-protein study showing that Acidithiobacillia merited separation from γ-proteobacteria (Williams and Kelly 2013) and confirm that *Acidithiobacillus* and *Thermothiobacillus* are a robust clade (Hudson et al. 2014) that is sister to the β/γ-proteobacterial clade. As class names Betaproteobacteria and Gammaproteobacteria are no longer valid under ICNP, and we still think this joint clade should be one class (Cavalier-Smith 2002a), we establish new class Chromatiia to include β/γ-proteobacteria and Acidithiobacillia as three new subclasses: Acidithiobacillidae, Neisseriidae (β-proteobacteria), and Pseudomonadidae (γ-proteobacteria). The 98-protein tree put *Mariprofundus*, iron-oxidising lithoautotrophs (Makita et al. 2017), as distant sister of Chromatiia with strong support and grouped that joint clade with α-proteobacteria with insignificant support (Williams and Kelly 2013). Our CAT and ML trees both maximally support Chromatiia, *Mariprofundus* (ζ-proteobacteria) and α-proteobacteria (Caulobacteria cl. n.) jointly being a clade but are contradictory as to their relative branching order. ML agrees with previous ML trees in grouping *Mariprofundus* with Chromatiia, but much more weakly (64% support), whereas CAT strongly shows Chromatiia and α-proteobacteria (each of which contains photosynthetic purple bacteria) as a clade (i.e. Rhodobacteria sensu Cavalier-Smith 2002a). We suggest that the ML position of the long unbroken *Mariprofundus* branch is a long-branch artefact, caused by the long-branch α-proteobacterial clade being pulled one node too deeply. The likely correct CAT topology would have allowed us to retain Rhodobacteria in its original sense, which are almost certainly ancestrally photosynthetic (Imhoff et al. 2017), but the strong grouping of *Mariprofundus* with them and fairly strong grouping also of δ-proteobacteria with their joint clade (on CAT but not ML) makes it sensible to broaden Rhodobacteria to include Mariprofundales and δ-proteobacteria also even though neither clade is yet known to include purple photosynthetic bacteria. Inclusion of Mariprofundales in Rhodobacteria is robust to method and taxon sampling, but the position of δ-proteobacteria is sensitive to both and requires confirmation by independent evidence as they often instead group with *Leptospirillum* as sister to Acidobacteria. However, their grouping as sister to undoubted Rhodobacteria had over 90% support on a Mr Bayes protein tree and over 70% by ML (Lücker et al. 2013), so is probably correct. Their Bayesian tree equally strongly had *Nitrospina* as sister to that clade rather than to Acidobacteria. We therefore also include *Nitrospina* in Rhodobacteria as new class Nitrospinia (despite their ML tree having it weakly sister to Acidobacteria); treating it as separate phylum Nitrospinae (Lücker et al. 2013) was unwarranted taxonomic inflation that fails to show how it relates to other proteobacteria.

It is undesirable to further extend Rhodobacteria to include the next deepest clade (Acidobacteria plus *Leptospirillum*), because Acidobacteria now include *Chloracidobacterium* which is a moderately thermophilic, microaerophilic green photoheterotrophic bacterium with chlorosomes (Tank and Bryant 2015), not a purple bacterium. *Leptospirillum* was included in Geobacteria but appears not to have been formally placed in a family or order. NCBI classification assigns it together with *Nitrospira* and *Thermodesulfovibrio* and ‘*Candidatus* Magnetobacterium’ to ‘family Nitrospiraceae’, ‘order Nitrospirales’, ‘class Nitrospira’, and ‘phylum Nitrospirae’, though none of these higher groups appears to have been effectively or validly published. Presumably, the widespread use of ‘phylum Nitrospirae’ is based on the suggestion from a 16S rDNA tree entirely unresolved at the base that ‘*Leptospirillum*’ (then not a valid genus), *Nitrospira*’, and a clade including *Candidatus* ‘Magnetobacterium bavaricum’ may be a distinct ‘phylum Nitrospira’ (Ehrich et al. 1995). A 31-protein ML tree yielded maximal support for ‘Nitrospirae’ being a clade excluded from δ-proteobacteria, but only trivial support for it being sister to the sole included acidobacterial species, this clade being insignificantly supported as sister to δ-proteobacteria (Lin et al. 2014). Our CAT trees have moderate support for *Leptospirillum* (‘Nitrospirae’) being sister to Acidobacteria and stronger support for that joint clade being sister to Rhodobacteria rather than to ε-proteobacteria, but near maximal support for all these taxa forming a clade that also includes Deferribacterales and Chrysiogenales; apart from it strongly excluding *Aquifex*, this clade corresponds exactly to Proteobacteria sensu Cavalier-Smith (2002a). Therefore, ‘Nitrospirae’ cannot reasonably be excluded from Proteobacteria unless ε-proteobacteria are also excluded, which would be an undesirable break with past classifications. We therefore instead establish new class Nitrospiria and group it with former class Acidobacteria (now Blastocatellia) as new subphylum Acidobacteria within phylum Proteobacteria. This revised classification is as conservative as we could make it, as subphylum Acidobacteria has exactly the same circumscription as former phylum Acidobacteria Thrash and Coates 2012 (Thrash and Coates 2011), just slightly lower rank.

As validly published Acidobacteria Cavalier-Smith 2002a was rejected as a class name for no apparent reason (Parker et al. 2014), we replace it by new class Holophagia in conformity with Rule 8 of ICNP; however, that rejection does not stop continued use of Acidobacteria as a phylum or using it as subphylum as we now do. We do not agree that the relatively small additional divergence of Holophagales compared with other Acidobacteria was an adequate reason for separating them as class Holophagae (Fukunaga et al. 2008). Instead Acidobacteria sensu Cavalier-Smith (2002a) should remain one class, for which we adopt class name Blastocatellia (Pascual et al. 2015) in a broadened sense as the older alternative Holophagae is invalid under ICNP rule 8. Order Acidobacteriales Cavalier-Smith 2002a was also nomenclaturally rejected without sound reason (Tindall 2014). These unwise rejections caused taxonomic confusion within Acidobacteria as Acidobacteriaceae ceased to have a valid order or class. As class Acidobacteria explicitly included *Holophaga*, *Acidobacterium*, and *Geothri*x, which collectively merit only one class, we rectify that problem by establishing new order Terriglobales for former Acidobacteriales, as order Acidobacteriales must no longer be used (but it still is, e.g. Foesel et al. 2016). We also create new family Nitrospiraceae, order Nitrospirales, and class Nitrospiria. As *Nitrospira* and *Thermodesulfovibrio* are genetically as divergent as Chromatiia and α-proteobacteria (Lin et al. 2014), and more divergent than any Holophagales are from each other we establish a separate order Thermodesulfovibriales that also includes ‘Magnetobacterium’. We group Nitrospiria and emended Blastocatellia as sole classes in new subphylum Acidobacteria of Proteobacteria (see Taxonomic Appendix) and provide a new formal description for Rhodobacteria. Thus revised, Acidobacteria and Rhodobacteria are sister clades.

Class Ferrobacteria and its type order Geovibrionales validly published by Cavalier-Smith (2002a) also were eventually unfairly nomenclaturally rejected without a specific reason (Tindall 2014), so alternative names Deferribacteres and Deferribacterales (not validly published until later in 2002) are now widely used for the same groups. However, Deferribacteres is now invalid under ICNP rule 8, as is class Chrysiogenetes with sole order Chrysiogenales of anaerobic arsenate-respiring flagellate negibacteria (Garrity and Holt 2001b). As Chrysiogenales and Deferribacterales are strongly sisters on our CAT and ML RP trees and have rather similar phenotypes (Deferribacterales subclade comprising *Denitrovibrio* and *Seleniivibrio* can also respire arsenate (Denton et al. 2013; Rauschenbach et al. 2013)), making both separate phyla (Chrysiogenetes, Deferribacterales: Garrity and Holt 2001a, b) was unjustified rank inflation. We therefore group both orders of metal reducers in the same new class Deferribacteria within a reestablished proteobacterial subphylum Geobacteria, which comprises Deferribacteria plus ε-proteobacteria, fairly strongly supported sisters on CAT but separated on ML RP trees (see Taxonomic Appendix). As Epsilonproteobacteria (Waite et al. 2017) is now an invalid class under rule 9 of ICNP, and Epsilobacteria published earlier (Cavalier-Smith 2002a) was later rejected, we make new class Nautiliia for ε-proteobacteria, which are markedly different in flagellar structure and motility from those proteobacteria classified in Rhodobacteria (Beeby 2015). Flagella of Deferribacteria like those of Nautiliia are polar or bipolar and cells spiral or curved, *Flexistipes* being the only non-flagellate filamentous genus (Fiala et al. 1990); it is important to study their flagellar structure in detail as it is possible that distinctive features of Nautiliia flagella and motility are characteristic of all Geobacteria. Flagella of Acidobacteria need similar study to see if this might be the ancestral state for all Proteobacteria and those of peritrichous Rhodobacteria like *Escherichia coli* secondarily simplified.

## Defects of ranking prokaryote taxa by arbitrary rDNA divergence

For decades, microbiologists have used rDNA similarity as a practical rule of thumb for assigning new prokaryote ‘species’ to existing orders, classes, and phyla. Commonly if it robustly groups on a 16S rDNA tree with an existing clade widely accepted as a phylum, it is assigned to that phylum, but if grouping is uncertain, it is often made the basis for a new phylum. The number of supposed ‘phyla’ has mushroomed, 39 listed on a popular website as of 19 July 2018 (http://www.bacterio.net). When this practice is (a) formalised by adopting an arbitrary numerical cutoff of 75% 16S rDNA identity as a threshold of divergence claimed to be sufficient reason to split prokaryotes into separate phyla (Yarza et al. 2014) and (b) extended to uncultivated environmental sequences to propose ‘candidate phyla’, supposed phylum numbers explode to 118 (Hug et al. 2016), which is scientifically unsound and taxonomically unwise.

The Candidatus concept when applied to partly studied species whose names are not validly published is practically useful, but extending it to phyla is seriously harmful to science, nomenclature, and taxonomy, as it tends to formalise ignorance rather than knowledge, and divert attention from the need for better evaluating the reasons for giving high ranks to some taxa. In traditional taxonomy, merely quantitative divergences like size, numbers of bristles on an insect leg, or flowers on a stem were treated as minor differences valuable for distinctions at low ranks only. Phyla were based on major evolutionarily very stable qualitative differences in shared body plan, as in chordate, arthropod, or molluscan animals or vascular plants versus green algae. In eukaryotic microbes, the same is done using a combination of ultrastructural and molecular characters, only 8 phyla now being recognised in kingdom Protozoa and 8 in kingdom Chromista (Cavalier-Smith 2018). That conservative approach ought to be applied in prokaryotes too to make phyla biologically meaningful and practically valuable for grouping definitely related subgroups more clearly and economically. Spirochaetes, Cyanobacteria (excluding Melainabacteria), Actinobacteria, Chloroflexi, and Endobacteria exemplify good, sensible eubacterial phyla, and the eu/archaebacterial distinction an excellent supraphyletic one, each of whose body plans are very different from the others. But establishing 34 new ‘candidate phyla’ in a ‘candidate phyla radiation’ (CPR) (Hug et al. 2016) of unknown body plans is a *reductio ad absurdum* of ranking by numerical thresholds (Yarza et al. 2014). As Candidatus by definition is of indeterminate rank, the phrase ‘candidate-phyla’ is thoughtless self-contradiction. CPR lineages are all miniaturised eubacteria (ultrastructurally negibacteria) with tiny genomes that are likely to be rapidly evolving (thus exaggerating their significant divergence) and likely mutually related as a single clade; the idea that they and convergently miniaturised DPANN archaebacteria (see below) are early primitive life forms (Castelle and Banfield 2018) will probably eventually be shown to be a serious misinterpretation as was Woese’s similar idea based just on hyperaccelerated rDNA trees that microsporidia were the most primitive eukaryotes (Vossbrinck et al. 1987). Subdividing them into phyla merely because many branches exceed the scientifically meaningless 75% difference threshold is taxonomically harmful. In our present state of ignorance, the ranks of CPR cannot be sensibly discussed, but there is no reason yet to think that more than one phylum will ever be needed for CPR as a whole. None of their genomes was available when our analyses began. But that probably does not seriously limit our conclusions, because more likely than not the whole CPR clade really belongs in a well-known phylum, e.g. Proteobacteria, and their separate position on the published ML tree is a LBA towards neomura caused by their accelerated evolution. Merely having accelerated sequence evolution through reductive evolution is not a rational reason for subdivision into numerous phyla.

That would be equivalent to subdividing parasitic long-branch microsporidia into lots of phyla merely because their 16S rDNA evolved so much faster than in other protozoa (Bass et al. 2018). But protozoologists avoid the mistake of believing that rDNA is a molecular chronometer and that mere differences in evolutionary rate is of any deep evolutionary or taxonomic significance; examples of major accelerations in rDNA evolution associated with marked cell miniaturising exist in rhizarian chromists—though temporarily taxonomically confusing their extreme rDNA divergence was no reason to establish new phyla (Stentiford et al. 2017). It would be ridiculous to set arbitrary levels of sequence divergence for ranking in protists as some do in prokaryotes. Degree of divergence must be taken into account by taxonomists, *but not arbitrarily preset* (Yarza et al. 2014) or overvalued compared with biologically more meaningful characters.

The recently described genus *Abditibacterium* (name effectively but not validly published) exemplifies current low standards of prokaryote higher level taxonomy, as new ‘phylum Abditibacteriota’ was proposed on the weak basis of rDNA trees including no other cultured bacteria but Armatimonadetes and protein trees including only Armatimonadetes and Chloroflexi (or additionally with *Deinococcus*) (Tahon et al. 2018) and added uncritically to NCBI ‘taxonomy’. This lineage may just be a deep-branching member of phylum Armatimonadetes meriting no higher rank than order or class; the more reliable protein trees do not rule that out and the rRNA trees show abditibacteria and WS1 as long branches that might have been artefactually excluded from Armatiomonadetes. The phylum was described thus: ‘defined based on the phylogenetic analysis of 16S rRNA gene sequences. Members of this phylum form a stable lineage separate from candidate lineage WS1 and Armatimonadetes’. That is totally inadequate and almost meaningless as that ‘description’ could apply to every phylum except Armatimonadetes. It neither specifies the clade included nor gives any characters it possesses. Most papers naming phyla are even worse as their ‘definitions’ seldom mention any taxa included or excluded! This paper was too recent for us to include its RPs in proper bacteria-wide trees and better evaluate it.

More patience and more knowledge are needed before a stable prokaryote higher taxonomy is possible. The purpose of hierarchical ranking is to simplify classification by keeping the number of highest ranked taxa as low as we reasonably can so it is easier for human brains to grasp the big picture of biodiversity (Cavalier-Smith 1998b). Earlier, Cavalier-Smith (1992b) recognised 13 eubacterial and two archaebacterial phyla, later reducing them to 7 and 1 (Cavalier-Smith 2002a), modified to 12 eubacterial ones in Cavalier-Smith (2006d). Cavalier-Smith (2002a) argued that many candidate eubacterial phyla then being proposed from environmental DNA sequencing of hot habitats would prove really to belong in known phyla when better resolving multigene trees were available. That has turned out to be true, Armatimonadetes being the only one that still merits that rank. Recent discoveries and the results of our RP trees now mean that we recognise 14 distinct eubacteria phyla (Fig. 11); in archaebacteria, only Euryarchaeota and Filarchaeota should be accepted as phyla, making 16 prokaryote phyla, the same as the number now recognised in kingdoms Protozoa and Chromista collectively. Nine of the 14 current eubacterial phyla were already represented as clades on the classic early 16S rDNA trees, which also showed Chlorobiales plus Bacteroidetes and Planctomycetes plus chlamydiae as clades (Woese 1987). However, the branching order on that pioneering rDNA tree amongst ‘phyla’ was almost entirely wrong, as shown by our more accurate completely resolved CAT RP tree, except for one thing: Planctobacteria and Sphingobacteria being sisters. Thus, site-heterogeneous RP trees are a much better basis for prokaryote evolutionary taxonomy than were site-homogeneous 16S rDNA trees. But even if a sister relationship exists with high support, as between Melainabacteria and Cyanobacteria, that alone is not a sound reason for lumping such microbes into one phylum (Utami et al. 2018), if distinctions between them are important enough to merit separate phyla as originally suggested (Di Rienzi et al. 2013).

Many relationships unclear from 16S rDNA are now well established by multiprotein trees. But even they can be confused and seriously biased by hyperaccelerated evolution in secondarily miniaturised cells like many parasites, as exemplified in eukaryotes by microsporidia and *Mikrocytos* which belong in phyla Opisthosporidia and Retaria respectively and do not merit separate phyla as sequence divergence alone would misleadingly tell a mindless computer. Eubacterial Dependentiae (SM6; often endoparasites of eukaryotes), none with validly published names so not includable in formal taxonomy but unwisely called a ‘candidate phylum’ (McLean et al. 2013; Deeg et al. 2019), are a prokaryote example, almost certainly just a branch of phylum Proteobacteria that might eventually deserve class or subclass rank within subphylum Acidobacteria.

## Multiprotein sequence divergence ranking: illusory objectivity

Problems of false topology and extremely idiosyncratic rate changes would be reduced but not eliminated if multiprotein trees were used rather than rDNA, especially if some correction is made for rate differences across taxa, as in a eubacterial study of 120 proteins (Parks et al. 2018). Their elaborate computer-based approach concluded that the then 65 CPR ‘phyla’ collectively merited no more than one phylum, exactly as we did by a few minutes’ thought by one human brain, so their normalisation method to allow for rate differences is clearly superior to the naive rDNA distance approach. However, the method is not as objective as they supposed and has many arbitrary aspects. First, their divergence estimates depend on knowing the root position, the most controversial phylogenetic inference of all; they sidestepped the problem by using midpoint rooting and averaging, which is not evolutionarily or scientifically correct but merely computationally convenient, and necessarily biases estimates. Secondly, linear interpolation of divergence times is arbitrary as rates undoubtedly change with time in unpredictable ways. Thirdly, there is no particular evolutionary or biological significance of degree of sequence divergence, so using it as the sole criterion for ranking is not objective but a subjective choice just done for convenience. In fact, they did not choose a specific degree of divergence for establishing phyla, classes, orders, etc. objectively. Instead, they calculated the median degree of divergence for existing taxa of a given rank (presumably based on the hodge-podge NCBI taxonomy, but not explicitly stated) and used that to assign ranks of clades on their new trees. Therefore, their supposed objective method largely perpetuates the errors in judgement made earlier by erratic RNA distance ranking criticised above—not precisely, because their trees will be better and they will have been able to reduce polyphyly, and the spread of degrees of sequence divergence associated with different ranks is less. Furthermore, they did not apply the results consistently but made various manual adjustments (not individually specified or justified). Phylogenetic computer programmes all have a subjective basis of partially incorrect or arbitrary assumptions and of choice of input data or of algorithms. Reassuringly for the present study, Parks et al. (2018) found that using only 16 RPs gave almost as accurate trees as 120 proteins, 16S rDNA alone being much less accurate. The 26 RPs for eubacteria and 51 RPs for archaebacteria used here should be even closer to 120-protein trees. Our CAT trees are probably better than their trees because of the site-heterogeneous model. All our trees are likely to be less biased by long-branch artefacts as we excluded the CPR ‘phylum’ whose presence (together with that of neomura) probably explains why the eubacterial backbone of the Hug et al. (2016) tree has almost no bootstrap support.

It is extremely hard to evaluate their higher taxonomy as Parks et al. (2018) do not even list the excessive 99 eubacterial phyla (114 on website plus 11 archaebacterial) in their system and their website is extremely opaque—I could not find any such list or list of which classes are in each phylum comparable to our Table 2 or see how one could assess the effects of their computer output on particular groups of interest, and so on. From their Fig. 2, it appears that a given degree of normalised sequence divergence can correspond to two different ranks, and some classes even on their taxonomy can have the same degree of divergence as some phyla or some orders, so it is misleading to imply that it applies one objective standard to ranking. However, even though more sensible for CPR, it has grossly inflated the number of other taxa at each rank compared with NCBI, which for phyla (and arguably classes) at least is the opposite of what is required for a good sensible taxonomy that makes things simpler without compromising phylogenetic accuracy. Partly because they want to be able to feed every genome into one tree, the phylogenetic methods were chosen for computer speed not accuracy. It is good to use multiprotein trees rather than rDNA as a guide for establishing higher taxa, but better to use a representative sample to enable more accurate methods and study of artefacts, e.g. of taxon sampling, and to integrate results with other evidence, as here, when deciding on ranking, and not to delegate that important taxonomic function to an arbitrarily programmed computer. Parks et al. (2018) is not a practically useful contribution to taxonomy as none of their presumably numerous new names is validly published or individually explained. Such methods may be useful to genome sequencers wishing to assign quickly an unknown genome to approximately the correct place in the tree, but are inadequate as a general reference taxonomy, for which the eclectic classical approach used here is greatly preferable. Of the supraspecific names they used only 18% are validly published. There is a great risk that ranking by automated methods with opaque assumptions will cause thoughtless splitting, unnecessary name changes and overcomplications with no clear rationale. For sound higher taxonomy, human thought and expert taxonomic judgement is needed, which should not be pejoratively labelled subjective. It is always based on objective evidence. A posteriori ranking by one brain based on all available evidence is superior to a priori ranking by arbitrary numerical thresholds which give different results with different algorithms and data samples.

The excessive number of phyla and rank inflation generally in Parks et al. (2018) arises because they do not even consider the possibility of using intermediate-ranked categories like subphyla, infraphyla, superclasses, subclasses, superorders, suborders, as standard in eukaryote taxonomy, which would greatly improve prokaryote classification if more widely adopted by reducing drastically the number of phyla and classes, thus increasing comprehensibility and providing a better quick overview of bacterial diversity, as Table 2 exemplifies. We should not lose sight of the primary simplifying purpose of classification, best served by severely limiting the number of highest ranked taxa and keeping numbers relatively small at each higher rank by proper use of intermediate categories, especially in ultradiverse groups like Proteobacteria.

## Supraphyletic prokaryote taxa

We here treat Prokaryota as a superkingdom or empire with Eubacteria and Archaebacteria ranked as kingdoms as in the seven-kingdom system of Cavalier-Smith (1986) and Ruggiero et al. (2015) and therefore now rank Euryarchaeota and Filarchaeota as phyla, the only two in the kingdom. The 14 eubacterial phyla need grouping in higher-level taxa. Earlier subkingdom Unibacteria, grouping posibacteria and archaebacteria (Cavalier-Smith 1998a), is polyphyletic and Negibacteria is multiply paraphyletic so we abandon them. The most fundamental and likely ancient contrast is between the primitively LPS-free Chloroflexi, here assigned to new subkingdom Chlorobacteria originally a phylum (Cavalier-Smith 1992b). The other 13 are here divided into three subkingdoms (two new) whose common ancestor ancestrally had an OM with LPS, but which multiple character losses made phenotypically heterogeneous. Earliest branching bacteria with LPS are the new subkingdom Eoglycobacteria, the clade comprising Armatimonadetes plus the Cyanobacteria/Melainabacteria subclade, invariably with murein sacculus and an OM with LPS. They are sisters to a much larger group that is more heterogeneous in envelope structure, here divided into two paraphyletic subkingdoms: Posibacteria (Actinobacteria, Endobacteria) and Neonegibacteria (infrakingdoms Gracilicutes and Thermobacteria infrak. n.). Why evolution makes acceptance of some ancestral (paraphyletic) groups like prokaryotes or Gracilicutes necessary or desirable was explained previously (Cavalier-Smith 1998b, 2010a).

Infrakingdom Gracilicutes (Cavalier-Smith 2006a) is now thoroughly established as monophyletic but RP trees show the other two infrakingdoms in that interim classification are polyphyletic, so we abandon them but establish paraphyletic infrakingdom Thermobacteria to embrace ancestrally or largely thermophilic phyla Aquithermota, Synthermota, Hadobacteria plus Fusobacteria that though not thermophilic nests somewhere within them. We establish superphylum Planctochlora for Planctobacteria plus Sphingobacteria, which are always a robust clade on site-heterogeneous eubacteria-only trees.

Battistuzzi and Hedges (2009) using less accurate site-homogeneous methods for 25 proteins thought they had established two major ‘clades’ of the less thermophilic eubacteria, but our RP trees imply that neither is a clade. Their ‘Terrabacteria’ comprise Chloroflexi, Cyanobacteria, Posibacteria, and Hadobacteria which never form a clade on our trees. Essentially, they comprise the most basal lineages on our trees plus Hadobacteria, so are polyphyletic. They are not a clade partly because of the inclusion of Hadobacteria (that likely artefactually grouped with Actinobacteria by ML) and also because their tree was misrooted in the neomuran stem; if our rooting is correct, even if we excluded Hadobacteria from terrabacteria they would be paraphyletic. Their group hydrobacteria is identical to Gracilicutes but as the name Gracilicutes was proposed three years earlier they should have used it and cited the indel evidence for it previously explained by Cavalier-Smith (2006a). Their tree had a phylum Sphingobacteria clade but wrongly put both Sphingobacteria and Spirochaetes within Planctobacteria, so failed to show the Planctochlora clade, which is inconsistent not only with our trees but most other recent multiprotein studies. In contrast to our trees that better sampled deep phylogeny, Thermotogales and Aquificales were sisters and jointly sister to Fusobacteria. Gracilicutes/hydrobacteria are not a clade, because neomura probably evolved from them as shown by our two-domain trees. GenBank should stop using the polyphyletic Terrabacteria group. Table 2 provides a better higher classification of eubacteria.

## Eubacteria were ancestrally photosynthetic

The seven phyletically distinct eubacterial groups possessing photosynthesis are found in five different phyla: Chloroflexi, Cyanobacteria, Endobacteria, each with only one major kind of photosynthesis, plus Proteobacteria and Sphingobacteria, each with two distinct types. As photosynthetic reaction centres (RC) are all homologous (Sadekar et al. 2006), photosynthesis evolved once only, so we have to explain why none of these phyla is sister of another, all being interspersed with the nine entirely non-photosynthetic ones. Woese (1987) suggested that the ancestral eubacterium was possibly photosynthetic and Cavalier-Smith (1987b, 1992b, 2001, 2002a, 2006a, c) more strongly argued that the first eubacterium was photosynthetic. If so, photosynthesis was lost independently by immediate ancestors of all nine non-photosynthetic ones. As evolutionary loss is very easy by simple deletion and would often have been selectively advantageous in producing specialised heterotrophs or chemoautotrophs, it is entirely reasonable that numerous losses occurred—nine is far fewer than the number of photosynthesis losses inferred in eukaryote kingdom Chromista, though additional losses must have occurred within the four non-cyanobacterial phyla just listed. Yet ever since the reality of LGT was demonstrated, many have preferred to invoke LGT is an alternative explanation of the scattered distribution of photosynthesis, but have usually done so with extremely weak evidence or even no explicit suggestion of a source or sink of postulated transfers. Figure 11 shows how the two RC types map onto the now robust RP tree.

If the tree is rooted on Chloroflexi, the cenancestral RC was heterodimeric type II with distinct L/M paralogues, as previously argued (Cavalier-Smith 2002a, c), which would have evolved from a pre-LUCA homodimeric ancestor of L/M with identical subunits having five transmembrane helices that itself probably evolved from a simpler single-helix protein such as the light-harvesting (LH) antenna of Chloroflexi and Proteobacteria (Olson 2001). RC II proteins are shorter and simpler than RC I proteins and transfer electrons to (bacterio)phaeophytin quinones that could have been available prebiotically and are thus mechanistically more plausible than RC I as ancestral, contrary to a widespread view (Cardona 2017; Martin et al. 2018; Olson 2001). RC I appears to have evolved in an ancestor of cyanobacteria by duplication of RC II followed by a gene fusion linking it with the 6-transmembrane helices of a CP43-like protein to make the 11 transmembrane helices of RC I (Murray et al. 2006). As 6-helix CP43/CP47-like proteins are restricted to cyanobacteria, they probably arose at least as early as the stem lineage preceding the divergence of Cyanobacteria and the Endobacteria/neonegibacteria branch of the tree. This fusion could have occurred in the precyano/endobacterial stem after it diverged from Armatimonadates or one node earlier on the RP tree in the stem of all glycobacteria after it diverged from Chloroflexi. CP43/CP47 proteins may have played a central role in evolution of a second reaction centre (pre RC I) before the gene fusion that made RC I.

The great antiquity of photosynthesis by L/M reaction centres is reinforced by an eighth lineage known only from environmental DNA sequencing (‘Eremiobacterota’ = WPS-2 ‘candidate phylum’: Ji et al. 2017) which is sister to Armatimonadetes on 38-protein trees by FastTree (less accurate than ML) apparently having distinctive L/M reaction centres and RuBisCo in four sublineages from boreal mosses (Holland-Moritz et al. 2018; Ward et al. 2019). L and M proteins are most closely related to those of Chloroflexi but so distant that we cannot infer LGT from one to the other, making it probable that the common ancestor of Chloroflexi and WPS-2 was photosynthetic and both lineages multiply lost photosynthesis. Their bacteriochlorophyll synthesis gene *bchY* evolves rapidly like those of Heliobacteria and Chlorobi so it is likely that their grouping together on the tree is a long-branch artefact; anyway, this tree gives no evidence of LGT to or from other phyla and shows deep divergence from all. If this novel anoxygenic photosynthetic lineage were genuinely sister to Armatimonadetes, the second deepest branch after Chloroflexi on our trees, it could with advantage be made a new armatimonad subphylum rather than a novel phylum, which would increase the number of ancestrally photosynthetic phyla to six. Alternatively, as WPS-2 were as close to Chloroflexi as to Armatimonadetes by rDNA (https://www.biorxiv.org/content/10.1101/534180v2), it might just be a highly divergent chloroflexan lineage deserving subphylum rank, as RC trees imply—Chloroflexi were nearly as close as Armatimonadetes on the 38-protein tree. It is vital to culture eremiobacteria to test inferences from metagenomes and study all aspects of their biology, including cell envelope structure and whether they have chlorosomes as they apparently branch so close to the inferred base of the tree. If they turned out to lack an OM, they would be candidates for a primitively monoderm lineage ancestral to negibacteria, thus the most divergent bacteria of all.

On this view, the ancestral L/M reaction centre was inherited vertically by Proteobacteria but was lost by Heliobacteria, Chlorobi, and Acidobacteria that kept the new RC I instead (in its original homodimeric form; only in the ancestor of cyanobacteria did RC I undergo duplication and divergence to make heteromeric (PsaA/B) photosystem I). The only plausible example of RC LGT between phyla to date is for the sphingobacterium *Gemmatimonas* (in Sphingobacteria: Fig. 5), whose L and M proteins both nest on trees within those of Rhodobacteria, implying that its reaction centre came by LGT from a proteobacterium after Proteobacteria and Sphingobacteria diverged. In contrast to *Gemmatimonas*, the presence of RC I related to that of Chlorobi in *Chloracidobacterium* cannot confidently be attributed to LGT, as it is not nested within Chlorobi, but is their sister just as it would be if it had been inherited vertically from the common ancestor of Gracilicutes, which could have possessed both RC I and RCII, and one cannot rule out the possibility that RC I was lost several times within Proteobacteria as RCII clearly has been. Thus vertical inheritance and lineage sorting by differential loss can explain RC present distribution with minimal LGT. Only cyanobacteria kept both RC I for photosystem I and RC II for oxygenic photosystem II. This interpretation is fully compatible with the distribution of indels in RC proteins which rules out many theoretically possible LGTs.

The gene fusion had to involve only one of the cenancestral RC proteins L and M, but it is impossible to determine which fused to make RC I as they are equidistant from RC I on trees. Though cyanobacteria kept RC II it was not the ancestral L/M version. Sequence phylogeny and RC 3D structure decisively show that an independent cyanobacterial D1 and D2 arose by a duplication of RC II independent of the one that generated L and M (Cardona 2015). It is simplest to suppose that D1 and D2 arose at the same time as the origin of RC in the same stem lineage. Indeed, the very same RC II duplication that preceded the gene fusion (whether of L or M) could have been serial and yielded four copies: one could have fused with CP43-like to make RC I, two could have diverged to make D1 and D2. The three structural regions unique to D1 and D2 (Cardona 2015) must have arisen in their immediate common ancestor (likely a secondarily homodimeric intermediate) before the duplicates diverged. D1 and D2 both have homologously attached peripheral chlorophylls that allow excitation energy transfer from core antenna to RC that are also present in RC I (Cardona 2017). This sharing of chlorophyll-coordinating histidines in homologous regions of RC I and D1/D2 (but not the ancestral L/M RC II) is simply explained if the same duplicated subunit (whether L or M) was ancestral to both RC I and D1/2 and this sequence signature arose in their common ancestor after it diverged from L/M in the glycobacterial stem lineage prior to the D1/D2 divergence. Thus, it is not necessary to suppose that photosystem II is chimaeric as recently argued (Cardona 2017), nor that the last common ancestor of all photosynthesisers had two RCs.

This model for RC evolution is therefore simpler than any previous ones and allows more gradual evolution and successive increases in complexity as well as later simplifications of non-chloroflexan anoxygenic lineages. This exemplifies how evolution becomes simpler to understand if one has a robust correctly rooted tree, and maps innovations carefully onto it, and how incorrect rooting can make things appear over-complex. Our interpretation implies a period of multiple RC II duplications and mutational divergence in the glycobacterial stem after it diverged from Chloroflexi but before the origin of oxygenic photosynthesis in the cyanobacterial stem, followed by differential losses as glycobacteria radiated.

Though RC evolution was largely vertical, one clear example of LGT exists within Chloroflexi: transfer from the *Roseiflexus* subclade (suborder Roseiflexineae) of the major exclusively photosynthetic subclade (order Chloroflexales of class Chloroflexia) to an unnamed member (CP2_42A) of the predominantly non-photosynthetic Anaerolineidae. This LGT is particularly convincing as it involves an unusual secondarily fused L/M fusion gene that evolved at the base of the *Roseiflexus*/*Kouleothrix* subclade (Roseiflexineae) and it involves a non-controversial serious mismatch between the RNA polymerase phylogeny which probably roughly represents organismal and cell lineage evolution, and the RC II tree. However, a second claimed LGT of an unfused operon from Chloroflexineae into Anaerolineidae involving the common ancestor of *Candidatus* Rosilinea gracile and JP3_7 is likely a misinterpretation, as discordance between these trees is markedly less: moving the fusion subclade across just one node on the RC tree (for which no statistical support is given) would make it congruent with the organismal/polymerase tree. If our interpretation of vertical inheritance of the Rosilinea RC is correct that would make photosynthesis the ancestral condition for Chloroflexi in accord with our view that photosynthesis extends back to LUCA. That would imply numerous losses of photosynthesis within Chloroflexia similarly to the numerous losses that most now accept occurred within purple bacteria (subphylum Rhodobacteria) of Proteobacteria. A slightly inaccurate sparsely sampled RC II tree plus vertical descent seems to us at least as likely as LGT. Though Ward et al. (2018) regarded multiple losses as ‘more complex’, losses are probably mechanistically simpler than LGTs yet there appears to be a subjective bias towards invoking LGT rather than losses in many bacterial papers.

Before chloroflexan RC IIs outside Chloroflexia were known, Shih et al. (2017) claimed to have demonstrated recent LGT of anoxygenic photosynthesis into Chloroflexi, but that was based purely on Chloroflexales nesting relatively shallowly within Chloroflexi and deeper branching photosynthetic lineages such as ‘Rosilinea’ appearing to be unknown. In other words, it depended on assuming that such deep branching lineages never existed and the assumption that Chloroflexi RCs were never lost. They did not specify a possible ancestor for that purported LGT so their assuming LGT was explanatorily empty and devoid of direct evidence. The only known bacteria with L/M RC II that could possibly be donors are Rhodobacteria and *Gemmatimonas*. If the donor was either, then Chloroflexi RCs should nest clearly within those of Proteobacteria as do those of *Gemmatimonas*; indeed, they should nest even more shallowly if the LGT were as recently as 867 Ma as claimed, as crown Rhodobacteria are probably over three times that age, RP trees like Fig. 5 implying they are somewhat older than stem Cyanobacteria. They do not, whether on separate L and M trees or on a concatenated tree (Imhoff et al. 2017), but are invariably distant sisters. This directly rules out both possible sources of LGT for Chloroflexi RCs. On the concatenated tree (Imhoff et al. 2017), the relative length of the Chloroflexi and rhodobacterial sister branches is as expected if they diverged early at the very base of our RP tree.

Furthermore, if Chloroflexi got RCs by LGT, they also would have had to get bacteriochlorophyll synthesis genes; but their BchX and BchL proteins (subunits of protochlorophyllide oxidoreductase (POR) and chlorin reductase (CR), the two enzymes that make the bacteriochlorin precursor of bacteriochlorophyll *a*) are more closely related to those of Chlorobi than to those of Proteobacteria (Gupta 2012). Evolution of bacteriochlorophyll genes is complicated and was often interpreted in terms of ill-specified LGTs (Xiong et al. 2000), but trees are confused by using paralogue rooting which is extremely unreliable and biased by long-branch attraction (LBA) when stems between paralogues are very long (Cavalier-Smith 2002a, 2006d), as is so for POR and CR (Gupta 2012; Xiong et al. 2000). If POR and LR subunit trees are rooted on Chloroflexi as here, instead of by extremely distant outgroups subject to LBA artefact as before (Gupta 2012; Xiong et al. 2000), all are congruent with the RP trees, so no LGT need be invoked. Cyanobacteria have two very different POR BchL paralogues, one sister to the proteobacterial proteins and one related to *Heliobacterium* BchL (Gupta 2012; Gupta and Khadka 2016). This implies a BchL duplication in the cyano/endobacterial stem (or one node earlier) and differential loss of one of the two paralogues in subsequent lineages. On that interpretation, the BchL tree is congruent with our RP tree rooted on chloroflexi. The paralogue shared by clade C cyanobacteria and Proteobacteria has a unique glutamate insertion (Gupta 2012), so the other paralogue (found in Chloroflexi, Chlorobi, Heliobacteria) must be the ancestral version if Chloroflexi diverged first. Thus, no LGT is required to explain the patchy distribution of (bacterio)chlorophyll synthesis proteins other than LGT from proteobacteria to *Gemmatimonas* (which has the glutamate insertion (Gupta and Khadka 2016)) as for its RC, which could have been mediated by one transfer of the entire photosynthetic gene cluster including RC and Bch genes. Previous more extensive LGT assumptions stem from misrooting the tree and failing to recognise distinct paralogues.

The actinobacterium *Rubrobacter* though non-photosynthetic has two of the three POR proteins (BchN and B); trees for both show that they do not nest within any photosynthetic phyla, so give no evidence for LGT (Gupta and Khadka 2016). It lacks CR but has homologues of all three units of magnesium chelatase (BchD, H, I) the enzyme that inserts Mg++ into protoporphyrin IX the first unique step in bacteriochlorophyll synthesis, which is homologous with the 3-subunit cobalt chelatase, not to POR/CR; BchI is not homologous with the other subunits but with the huge and ancient AAA+-ATPase family (Sousa et al. 2013a). BchD is homologous with von Willibrand factor A (WfA) and its tree is congruent with RP trees if rooted on Chloroflexi (not spuriously by WfA (Sousa et al. 2013a)). The presence of these enzymes in one of the deepest actinobacterial branches means that other actinobcteria lost them and is consistent with the RP trees and our argument that Actinobacteria and all other non-photosynthetic eubacteria lost photosynthesis secondarily. These proteins may be relics of their inferred eubacterial photosynthetic ancestry retained through acquiring other uses. BchI phylogeny is complicated by there being two ancient paralogues in Chloroflexi and Chlorobi, but their joint tree was apparently incorrectly rooted (Sousa et al. 2013a); we root it between subclade A comprising only Chloroflexi, Chlorobi, and Proteobacteria (including Acidobacteria) and subclade B containing Chloroflexi, Chlorobi, and Heliobacteria (Endobacteria). If A and B are treated as separate clades, A is precisely congruent with the RP tree if rooted on Chloroflexi; B is more complex having two seemingly paralogous Chlorobi subclades, but if the longer of these (likely to be LBA-sensitive and topologically misleading) is omitted, B topology is also identical to the RP tree. That implies vertical descent since LUCA of both A and B BchI paralogues and that Sousa et al. (2013a) misrooted the tree within the B paralogue. We speculate that *Rubrobacter* lost the large subunit BchB of POR and evolved a simplified dimeric enzyme with different function from trimeric POR. The alternative assumption that the putative BchN/B dimer was a precursor of photosynthesis (Gupta and Khadka 2016), not a relic, could be true only if the universal tree were rooted within Actinobacteria, which indel evidence strongly rejects (Gao and Gupta 2005, 2012; Gao et al. 2006) and would require LPS to have been secondarily lost by Chloroflexi.

All three POR and CR subunits are homologous with those of the three-subunit nitrogenases discussed below and must have a common origin. BchI only is homologous also with ParA, the ATPase that functions for segregating chromosomal DNA in all eubacterial phyla and many archaebacteria (Barillà 2016). As ParA function almost certainly evolved preLUCA and like POR/CR and nitrogenase is present in Chloroflexi and works as a simple homodimer, we suggest it is likely ancestral to BchI and evolved before the trimeric homologues arose by gene duplication preLUCA. On that interpretation, protein-coded photosynthesis and nitrogen fixation both evolved before LUCA.

Chlorosomes are glycosyldiacylglycerol lipid monolayer vesicles containing thousands of molecules of bacteriochlorophyll *c* whose self-assembled stacks are exceptionally efficient at harvesting dim light in Chlorobi, some Chloroflexi, and the proteobacterium *Chloracidobacterium* (Hohmann-Marriott and Blankenship 2007; Orf and Blankenship 2013). They are attached to the cytoplasmic membrane by a homo-oligomeric base-plate protein (CrmA) that contains bacteriochlorophyll *a* (universal in anoxygenic phototrophs) and transmits excitation energy from its antenna chlorophylls to RCs (Oostergetel et al. 2010). Chlorosomes include 10% carotenoids that enhance antenna assembly and also contribute some excitation to RCs, and differ in the three groups, and some quinones that help survival in oxidative conditions and are simplest (just menaquinone) in Chloroflexi. CrmA homology and unique chlorosome structure shows that chlorosomes evolved once only. LGT in contradictory hypotheses was frequently supposed to ‘explain’ their phylogenetically patchy distribution (Olson and Blankenship 2004), but evidence for any seems absent.

As multiple losses are mechanistically easy and would be advantageous in lineages specialising in bright light habitats, we argue that preLUCA chlorosome origin and universal vertical inheritance coupled with numerous losses in chlorosome-free lineages is a better explanation. Before the ozone layer developed after cyanobacteria made enough oxygen, UV radiation would have been so intense that photosynthetic bacteria were probably confined to deep or extremely well-shaded habitats where benefits of chlorosomes would be at a premium. Only after the 2.4 Ga great oxidation event (GOE) could phototrophs invade brighter habitats and polyphyletically evolve new antenna complexes adapted to different light regimes: phycobilisomes of cyanobacteria, bacteriochlorophyll *g* of Heliobacteria, and novel purple carotenoids of Rhodobacteria. Chloroflexi suborder Roseiflexineae (Gupta et al. 2013), a shallow subclade much younger than GOE (Shih et al. 2017), arguably lost chlorosomes secondarily. Chlorosomes of suborder Chloroflexineae transfer excitation to RC via ringshaped integral membrane LH complex B808-866 that contains γ-carotene and two polypeptides related to those of the carotene-containing rhodobacterial ring LH (Xin et al. 2005). By contrast, Chlorobi and *Chloracidobacterium* use water-soluble Fe/S Fenna–Matthews–Olson (FMO) protein trimer instead, which must have evolved in a common ancestor and was not transferred by LGT between them. Previous LGT ideas, from or to Chloroflexi (Olson and Blankenship 2004), are incompatible with this dichotomy. Our well-resolved RP tree enables simpler interpretation by vertical inheritance: the chloroflexan ring LH is the ancestral state retained by Rhodobacteria, but FMO evolved in the ancestral gracilicute (likely from RC I PScA, which we argue evolved well after Chloroflexi: Olson 2004) and was retained by Chlorobi and *Chloracidobacterium* with chlorosomes, where FMO replaced the ring LH, whereas rhodobacteria lost chlorosomes and FMO but kept the ring LH. Chlorobi can also be considered derived as their chlorosomes often have bacteriochlorophylls *d* and/or *e* as well as *c*, unlike the other two green bacterial groups (Hohmann-Marriott and Blankenship 2011). The idea that cyanobacteria arose by fusing two lineages was never mechanistically plausible as bacterial cells never fuse (except in some actinomycete filaments within a species). Vertical inheritance, gene duplication in the precyano/endobacterial or glycobacterial stem, and subsequent divergences and losses fully explain their origin, as elaborated above. Sousa et al. (2013a) also refuted the fusion hypothesis.

## Molybdenum-dependent nitrogenase evolved before LUCA

Like photosynthesis, evolution of nitrogen fixation has been misinterpreted and LGT too often invoked through misrooting the tree and misunderstanding paralogues. Nitrogen fixation is known in euryarchaeotes and 12 eubacterial phyla including Chloroflexi but not in two small phyla (Armatimonadetes, Hadobacteria as suggested by our GenBank searches for Nif genes), so the claim that nitrogenase is not generally found ‘in deeply rooted linages’ (Boyd and Peters 2013) is mistaken. The nitrogen-fixing enzyme has two parts: a homodimer homologous to BchL and BchX that donates electrons, and a heterotetrameric acceptor with subunits homologous to BchN/Y and BchB/Z. The three related nitrogenase families use different metals: vanadium (V) by Vnf nitrogenases in Cyanobacteria, Endobacteria, Proteobacteria, and Euryarchaeota only, iron (Fe) by Anf nitrogenases in Proteobacteria, Sphingobacteria, and Euryarchaeota only, molybdenum (Mo) by Nif nitrogenases in all seven phyla. All species with Fe or V nitrogenases also have Mo nitrogenases, which occur additionally in Chloroflexi, Actinobacteria, Aquithermota, Synthermota, and Planctobacteria. As the taxonomically rarer V/Fe nitrogenases always group within Mo nitrogenases on concatenated sequence trees rooted on BchLNB proteins and have shorter branches, we infer that Mo nitrogenases evolved prior to LUCA, that V nitrogenases evolved no later than the cyano/endobacterial stem, whereas Fe nitrogenases evolved from a V-nitrogenase later still in the gracilicute stem from which euryarchaeotes inherited them vertically. This is consistent with isotopic evidence for a Mo-based nitrogen cycle going back at least 3.2 Ga (Stüeken et al. 2015), and with the combined sequence phylogenetic and palaeontological evidence that archaebacteria are at least three times younger than photosynthetic negibacteria (see below). However, Boyd et al. (2011a) claimed non-Mo nitrogenases to be ancestral and first evolving in euryarchaeotes and after transfer by LGT into eubacteria that Mo-nitrogenases only evolved later after the GOE. As we explain below, both conclusions were entirely unjustified phylogenetically; indeed, a few months later, three of the same authors (Boyd et al. 2011b) contradictorily but correctly concluded that Mo enzymes were ancestral, yet still kept the erroneous idea that they began in methanogens (Boyd and Peters 2013). Their errors probably stem partly from supposing that the universal root is between eubacteria and archaebacteria, but especially from misinterpreting paralogue trees, as nitrogenase evolution is complicated by multiple paralogues, e.g. two distinct paralogues occur in Chloroflexi, five in methanogenic archaebacteria, four in Endobacteria, and about four in Proteobacteria. We attribute most paralogues to early duplications and divergence but identify one clear case of LGT.

The metal cofactor of one subclade containing only endobacterial and methanogen paralogues has not been identified; but groups within the Mo enzymes, so does not affect our argument that Mo use was ancestral. The long-branch Chloroflexales subclade was also assumed not to be assigned to a particular metal cofactor (Boyd et al. 2011b), but we argue is almost certainly Mo-dependent as Chloroflexales also have genes for NifE and NifN subunits of the NifEN heterotetrameric scaffold essential for assembling the FeMo cofactor, and are more closely related to NifD and NifK respectively than to Anf or Vnf proteins (Boyd et al. 2011a). Their joint tree strongly suggests that NifD and NifK diverged from each other long before the AnfK/VnfK common ancestor diverged from NifNK (Boyd et al. 2011a fig1C). That is to be expected if the Mo-nitrogenase DK heterodimer evolved from a preexisting BchYZ that in turn arose before LUCA (as argued above) and if the Anf/VnfK branch arose later in the cyano/endobacterial stem. If the NifN and Nif/Anf/VnfK subtree is considered separately Anf/VnfK branches within NifK, suggesting that their cofactor assembly scaffold evolved secondarily from the FeMo cofactor scaffold. On the NifD and NifE/Anf/VnfD subtree Anf/VnfD branches within the homologous NifD, not within NifE, suggesting that the D paralogue of non-Mo nitrogenases also arose secondarily from a Mo-dependent ancestor. VnfE and N branches are very long and group together, not with either NifE or NifN from which one might have expected they evolved. We suggest that their grouping together and the long VnE/N branch is either an artefact of LBA and ultrapid evolution of the scaffold associated with V/Fe cofactor assembly or else one of these proteins is misannotated and might actually be orthologues of N or of E (one each of Anf and Vnf). Whether no AnfE/N homologues being identified stems from an even greater divergence or because cells use NifE/N for this function needs investigation. But the foregoing evidence for Anf and Vnf proteins both being secondarily derived from Nif proteins invalidates the assertion that ‘“VnfEN” branch near the root of the tree’ (Boyd et al. 2011a).

Applying molecular clock algorithms to an even more highly paralogous tree combining all these Nif proteins with BchNBYZ was extremely unwise and could not possibly have given sensible dates for anything given the clear evidence from the paralogue trees for hyperaccelerated evolution in most stems of the trees. This error was compounded by misrooting the fundamentally non-clock-like tree of VnfEN, the most recent in-group of all. The absurdity of that pseudo-clock analysis is shown by two things. First, the base of the crown of the supposedly most ancient VnfEN clade was assigned the youngest age (~ 0.7 Ga from Fig. 4 of (Boyd et al. 2011a)—consistent with their having evolved after both Nif and Bch as we argue, but not with non-Mo scaffold having being the most ancient of these proteins. Second, the base of the crown of BchZ, which must have preceded LUCA as explained above, is dated as only about 1.75 Ga and no Bch crowns are dated as older than the GOE. Third, all nitrogenase subclades are dated as < 2 Ga, inconsistent with isotopic evidence that Mo-nitrogenase is > 3.2 Ga. None of this makes evolutionary sense; careful cross comparison of evidence, as we attempt here, should have revealed the fundamental flaws of that meaningless ‘temporal’ analysis of paralogue trees that so dramatically flout oversimplified assumptions of ‘clock’ algorithms—useful only if applied to relatively uniformly evolving *single* orthologues and calibrated by fossil dates needing no signifcant extrapolation beyond the direct evidence (neither true here).

In concatenated nitrogenase HDK trees rooted on BchXYZ the single V/Fe subclade is maximally supported and nests within ancestral (paralogous) Mo-nitrogenases comprising two ancient paralogues (Boyd et al. 2011b; Boyd and Peters 2013). Within the Fe-nitrogenase subclade, the sole archaebacterial sequence (*Methanosarcina*) is sister to the gracilicute clade (Chlorobi/Proteobacteria), which does not support their claim that eubacteria got nitrogenase from archaebacteria. Within V-nitrogenases *Methanosarcina* nests weakly within eubacteria (Cyanobacteria/Endobacteria/Proteobacteria), thus also not supporting that claim. On one tree, V and Fe nitrogenases are sisters (Boyd and Peters 2013); on the other, V-nitrogenase is weakly ancestral suggesting they are of fairly equal age but the taxonomically restricted Fe form evolved somewhat later. The probably Mo-dependent nitrogenases of Chloroflexales are sister to the well-supported major Mo subclade of two major subclades (here designated A and B) each of which contains a maximally supported deep-branching endobacterial clade (that does not nest within any other phyla) as well as Proteobacteria and contrasting sets of negibacterial phyla. The dual position of Endobacteria and Proteobacteria cannot reasonably be attributed to LGT and likely represents a gene duplication involving all three proteins before Endobacteria and Proteobacteria. Clade A includes the endobacterial *Heliobacterium*/*Deulfitobacterium* subclade (i.e. Peptidococcaceae: Antunes et al. 2016), Cyanobacteria, Aquificales, and four proteobacterial subclades; though cyanobacteria and Aquificales appear within Proteobacteria (contrary to RP trees) this may be poor tree resolution not LGT. Clade B includes a well-supported eubacterial subclade with *Methanosarcina* its sister; eubacteria comprise a different endobacterial subclade (e.g. *Clostridium*) that is sister to a maximally supported clade comprising three gracilicute phyla (Planctobacteria, Sphingobacteria, Proteobacteria) plus the chloroflexan *Dehalococcoides* (Boyd et al. 2011b). The eubacterial part of subclade B implies vertical inheritance plus one relatively late LGT from the sphingobacterial stem to Chloroflexi. *Methanosarcina* appears to be sister to clade B eubacteria which is discordant with our prokaryote RP trees where archaebacteria branch with the gracilicute subclade planctochlora. This may indicate that it represents a third ancient subclade or that it branches too deeply because of unusually fast evolution and LBA. In a tree omitting Chloroflexales, a methanogen-only Mo-dependent clade (*Methanococcu*s/*Methanobacterium*) is maximally supported sister to V/Fe nitrogenase. Contradictorily, their earlier tree put it as the most divergent of all nitrogenases (no significant support), presumably partly why they clung to the groundless belief that nitrogenase evolved in archaebacterial methanogens. However, as nitrogenase is unknown in Filarchaeota, we cannot strictly disprove two independent LGTs of Mo-nitrogenase from eubacteria, but the fact that neither nests within any eubacterial phylum makes that unlikely; therefore, we suggest that the methanogen-only Mo clade may represent another early diverging vertically inherited paralogue that diverged from the ancestral V/Fe paralogue before GOE, but after these clades diverged from Chloroflexales. The Endobacteria/methanogen subclade of unknown metal cofactor, which from its depth and non-grouping with the Fe/V clade we suspect is Mo-dependent, also nests within the Mo-nitrogenases; within this subclade, methanogens nest within Endobacteria, suggesting either that archaebacteria evolved from Endobacteria (Valas and Bourne 2011) or, as we suggest through its discordance with our RP trees, that Methanobacteria obtained this paralogue from Endobacteria by LGT (opposite to the LGT direction claimed by Boyd et al. (2011b)). The best sampled tree shows all three methanogen clades nested firmly within different eubacterial paralogue subtrees (Boyd and Peters 2013). Therefore, if their inheritance were vertical, eubacteria are ancestral to archaebacteria, as all neo- and palaeontological evidence when correctly interpreted shows (Cavalier-Smith 2006a, c, 2013a, 2014).

Paralogue trees combining BChl and Nif/Anf/VnF proteins have been completely misunderstood. Collectively, they have not just the 18 proteins with different names, but at least 15 more Nif paralogues of non-universal distribution. It is naive to suppose that they can all be rooted by adding a single outgroup such as ParA, the most likely ancestor, as this could only join the tree in one place (if itself a single paralogue) yet in fact each subparalogue has its own subtree and root—and roots will be of different ages depending on where in the tree the duplication generating the younger one occurred. To interpret such trees, one must identify each paralogue and recognise that evolutionary rates are often so much greater in paralogue subtree stems than in crowns that LBA will usually give spurious roots for each on the composite tree, possibly wrong in different ways. Previously, nobody attempted to disentangle such matters as done above, so earlier ideas were mutually contradictory and at variance with other evidence. We have inferred that duplications that generated BChl and all three subunits from an ancestor like ParA, as well as later duplications that generated the five named Nifs that are homologous with them, must all have occurred before LUCA; duplications making Vnfs probably postdated Cyanobacteria/Chloroflexi divergence, and Anfs arose after Endobacteria and Cyanobacteria diverged. Our interpretations are simpler than those previously, with many fewer LGTs, and compatible with the RP (likely organismal) tree and the early Archaean isotopic evidence for RuBisCo-based photosynthesis and Mo-based nitrogenase about a billion years before GOE and billions more years before archaebacteria evolved.

Not only does nitrogenase and FeMo scaffold phylogeny decisively disprove an archaebacterial ancestry for nitrogenase, but so does phylogeny of NifB, which is essential for making the FeMo cofactor. NifB is unrelated to nitrogenase in most eubacteria (Cyanobacteria, Actinobacteria, many Endobacteria, Sphingobacteria, and Proteobacteria); it exists as a gene fusion between an N-terminal domain from the S-adenosyl methionine (SAM) protein family and a C-terminal domain related to the NifX/NafY family (Boyd et al. 2011b). The only eubacteria in which NifB has the presumably ancestral state of separate unfused SAM- and NifX-related genes are Chloroflexi and Peptidococcaceae (Endobacteria). The simplest interpretation is that the SAM/NifX fusion occurred in the cyano/endobacterial stem after it diverged from Chloroflexi and that Endobacteria alone initially retained both unfused and fused versions, which were differentially lost in its sublineages, the unfused version being lost independently in Cyanobacteria, Actinobacteria, and Neonegibacteria. Euryarchaeotes also lack NifB fusion proteins: *Methanococcus* has separate SAM and NifX-like proteins, but only SAM genes were found in *Methanosarcina* (Boyd et al. 2011b). Rooting the SAM domain tree on the chloroflexan *Dehalococcoides* would make methanogen sequences branch from the cyano/endobacterial stem, the very point where the major nitrogenase gene duplications occurred, making it possible that they represent an ancient unfused version of NifB that persisted in the backbone of the tree until after all neonegibacterial phyla evolved. Alternatively, the methanogen genes may have evolved faster (suggested by failure to find NifX) and simply branch too low on the tree. Boyd et al. (2011b) used paralogue rooting with endobacterial molybdenum biosynthesis protein MoaA as the outgroup, which being very distant would likely have caused LBA to misroot the tree within the methanogen subtree, thereby contributing to the misconception that nitrogenase itself came from methanogens despite there being no direct phylogenetic evidence for that.

## Planctobacterial origin of Neomura

Our two-domain RP trees are contradictory concerning the eubacterial ancestors of neomura. Eukaryotes always appeared within Planctobacteria but in slightly different places (none strongly supported). Prokaryote trees were less consistent, placing archaebacteria slightly lower, either beside or near the mostly robust Planctobacteria/Sphingobacteria clade: with 26 genes, CAT-GTR put archaebacteria weakly as sister to Planctobacteria/Sphingobacteria, but with 51 genes did not fully converge, one chain putting them as sister to Planctobacteria/Sphingobacteria, the other more deeply as sisters of Gracilicutes plus Aquithermota. Less accurate ML put archaebacteria within gracilicutes, but Planctobacteria were one node lower: thus, with 26 proteins, archaebacteria appeared as sisters of Sphingobacteria only and with 51 proteins to a likely artefactual clade comprising Sphingobacteria and Spirochaetes. All trees therefore placed neomura unambiguously with, almost all within, Gracilicutes; most with Planctobacteria and/or Sphingobacteria their sisters. Though such a grouping with Planctochlora was not found previously for RPs, three published three-domain rDNA trees if correctly rooted beside Chloroflexi put neomura as sisters of Planctomycetes (Brochier and Philippe 2002; Whitman 2009; Williams et al. 2012); we know none grouping them with Sphingobacteria. Two of them took more effort to avoid LBA than the generality of rDNA trees that mostly use site homogeneous methods without excluding fastest evolving sites, and therefore tended to put neomura with Aquithermota and/or Synthermota.

All two- and three-domain RP trees exclude with maximal or near maximal support neomura from within Actinobacteria or Endobacteria (which collectively include all certainly monoderm eubacteria). All place neomura strongly (CAT) or weakly (ML) within Neonegibacteria (typically with/within Planctochlora on two-domain trees or on three-domain trees with them or Aquithermota and/or Thermocalda), not sister to any monoderms. On site-heterogeneous trees, for neomura to group with either posibacterial phylum would require them to cross at least two, maximally or near maximally supported, clades. Even on ML trees, archaebacteria do not have to cross any significantly supported nodes to be sister of Planctobacteria—usually only one unsupported node. Even though the huge rate acceleration in neomuran and ribosomal stems means that a large majority of the ancestral information concerning their position must have been lost, our taxonomically extremely comprehensive site-heterogeneous RP trees are the strongest sequence tree evidence yet that neomura did not evolve from monoderm posibacteria as was long argued on parsimony grounds to minimise OM losses (Cavalier-Smith 1987c, 2002a, 2014). Instead, they provide strong support for neomuran origin from gracilicute negibacteria by simultaneous loss of murein and the OM. We therefore now abandon the idea that neomura evolved from posibacteria by loss of murein only, as happened during the polyphyletic origins of mycoplasmas. The OM was therefore lost more frequently than once supposed. As an endobacterial ancestry is excluded, loss could not have involved endospores as did multiple OM losses in Endobacteria. Nor is there any evidence that murein hypertrophied to make an extra thick wall as is likely for the ancestral actinobacterium.

Instead OM loss probably involved mutations breaking or inactivating OM lipid transport mechanisms associated with the bridges linking CM and OM. As there is no cell biological or other reason to regard Sphingobacteria as likely ancestors of neomura, but many arguments for a direct evolutionary link between Planctobacteria and eukaryotes, as a later section explains, we argue that our trees placing eukaryotes within Planctobacteria are likely historically correct, whereas those putting them slightly lower as sister to Planctobacteria/Sphingobacteria or to Sphingobacteria or more rarely with Aquithermota/Thermocalda may be misleading. As well as Planctobacteria (comprising Planctomycetia, Verrucomicrobia, Chlamydiia, Elusimicrobia, and other less studied lineages) being a very robust clade on RP trees (and nearly all other published trees), their shared cell envelope features make them particularly good candidates for simultaneous loss of murein and OM. Their periplasmic space is usually inflated and much thicker than in other negibacteria, thus with many fewer strong connections directly between murein and the CM. Moreover, many have undergone partial loss of murein, which in Planctomycetia and *Chlamydia* especially is so sparse it was originally thought entirely absent (Cavalier-Smith 1987b). Therefore, many planctobacterial cells probably depend less on either murein or the OM for mechanical support than do typical negibacteria, so their simultaneous loss may have been less traumatic than the original assumption of neomuran descent from posibacteria (Cavalier-Smith 1987c).

As argued in the next two sections, many features of the eubacterial rod-like cell growth pattern and division mechanism were retained throughout the inferred planctobacterial to archaebacterial transition. As later sections explain, the intermediate almost certainly had cortical microtubules (mts) like those of the verrucomicrobial *Prosthecobacter*, which additionally would have stabilised stem neomuran cells during evolution of their new glycoprotein walls/surface coats from a preexisting planctobacterial S-layer. Therefore, origin of neomura from a planctobacterial ancestor is mechanistically less traumatic than would have been origin via a posibacterial wall-less L-form in the original model for earliest stem neomura (Cavalier-Smith 1987c). Retention of so many eubacterial features during the transition explains why the archaebacterial cell cycle is so fundamentally similar to that of their eubacterial, specifically planctobacterial, ancestors.

Two shared features of archaebacteria and eukaryotes previously rationalised in terms of an actinobacterial ancestry are proteasomes and serine/threonine (ST) kinases, both crucial for the origin of eukaryotic cell cycle controls. Both can now be explained as well or better by a planctobacterial origin of neomura. ST kinases are even more abundant in Planctobacteria than in Posibacteria but not restricted to these groups, found more sparsely in Chloroflexi, genus *Myxococcus* of δ-proteobacteria (where their presence led to the mistaken notion of this group being involved in eukaryogensis by cell fusion), Spirochaetes, and Gemmatimonadia. On an ML tree, neomuran ST kinases (and the sole spirochaete one) group within those of Planctobacteria, whereas *Myxococcus* and posibacterial ones branch more deeply closer to Chloroflexi (Arcas et al. 2013), essentially congruently with the RP tree. Eubacterial proteasomes were originally thought to be only in Actinobacteria (Maupin-Furlow 2012) and are still only well studied in them (Becker and Darwin 2017), but the recent genome sequencing explosion shows 26S proteasome components in every prokaryote phylum except Spirochaetes, so they evolved before LUCA and must have been lost in some proteobacteria, e.g. *Escherichia coli*. Thus, proteasomes are no longer a reason for singling out actinobacteria as neomuran relatives.

The ubiquitin system that labels proteins for proteasomal digestion was once thought eukaryote-specific, but ubiquitylation is now known in diverse prokaryotes, but may not be the ancestral protein-tagging mechanism; for such labelling, distinct prokaryotic ubiquitin-like proteins (Pup) used by Actinobacteria and related Ubact system requiring different conjugases from ubiquitin may be older, being found in Armatimonadetes, a few Proteobacteria, and many Planctobacteria (Lehmann et al. 2017). Archaebacteria and hadobacterium *Thermus* have a tagging mechanism whose tags (SAMPs) are distantly related to ubiquitin, but sampylation requires only the E1 enzyme, not E1, E2, and E3 like eukaryotic ubiquitylation (Fu et al. 2016). A few filarchaeote archaebacteria (some Asgards, and thaumarchaeote *Candidatus* Caldiarchaeum subterraneum) have genuine ubiquitylation (Fuchs et al. 2018); though that was assumed to be ‘ancestral’, ubiquitylation more likely evolved in eubacteria as E2 homologues abound in Planctobacteria and also occur in Posibacteria, Cyanobacteria, and *Myxococcus*. Attributing all these eubacterial ubiquitylating enzymes to multiple LGTs from eukaryotes (Arcas et al. 2013) seems just to reflect the widespread, essentially evidence-free, prejudice that the universal root is in the neomuran stem: in fact on their ML tree, the eukaryotic E2s are a clade robustly within paraphyletic eubacteria and closer to Planctomycetia than to most posibacterial and cyanobacterial sequences (Arcas et al. 2013).

Thus, ubiquitylation probably evolved as early as the common ancestor of Cyanobacteria and Actinobacteria and passed vertically to neomura from their planctobacterial common ancestor, likely together with probably younger sampylation (post neonegibacteria). Their present distribution is explicable as differential losses of one or other functionally equivalent tagging machinery in different lineages, e.g. loss of sampylation by eukaryotes and ubiquitylation by most euryarchaeotes (scattered distribution of ubiquitylation components across the entire archaebacterial tree (Adam et al. 2017) is best explained by ancestral presence and multiple losses). Early origins, functional redundancies, and differential losses shaped cell evolution much more than is generally recognised.

Apparently unaware of the neomuran theory (Cavalier-Smith 1987c) or of the strong evidence that the universal tree is actually rooted within eubacteria (Cavalier-Smith 2002a, 2006d), Devos and Reynaud (2010) listed numerous planctobacterial characters shared with eukaryotes that they interpreted as evidence that planctobacteria may be phylogenetically closer to neomura than are any other eubacteria. These similarities were all dismissed as superficial convergence (or results of hypothetical LGTs) and against phylogenetic evidence (McInerney et al. 2011). On the contrary, our RP two-domain trees are the first reasonably clear sequence tree evidence for a planctobacterial ancestry for neomura, especially eukaryotes, as Reynaud and Devos (2011) explicitly suggested. For the first time, we show that a planctobacterial origin is NOT contrary to phylogenetic evidence but fully consistent with it and may actually be correct. In criticising Devos and Reynaud (2010) and the neomuran and other versions of phagotrophic origin of eukaryotes (Cavalier-Smith 2009; De Duve 2007), McInerney et al. (2011) misleadingly asserted that these ideas do not ‘involve the participation of archaebacteria’ and ‘offer no account of the obvious and extensive sequence similarity that many eukaryotic genes share with archaebacterial homologues’—egregious distortions of neomuran theory. The authors either misunderstood or misrepresented it, perhaps to promote Martin’s phylogenetically discredited hypothesis of mitochondrial origins (Martin and Müller 1998).

From the outset, neomuran theory explicitly explained the origin of shared neomuran characters absent in eubacteria as shared derived characters that arose in the neomuran stem and have been stably inherited ever since (Cavalier-Smith 1987c), as repeatedly explained in great detail (Cavalier-Smith 2002a, c, 2006a, c, 2007a, 2009, 2010d, 2014). It was designed to explain that very sharing. To imply that it denies them is nonsense. Admittedly, Devos and Reynaud (2010) were much less explicit about that, but their paper implicitly recognised a shared neomuran ancestry and did not argue that a possible relationship of eukaryotes with planctobacteria contradicts their long-established relationship with archaebacteria. It does not; that should have been recognised by any fair criticism of their paper, which clearly implied that both archaebacteria and eukaryotes could be related to planctobacteria. If the root of the overall tree of life is within eubacteria, as the neomuran interpretation always explicitly argued, eukaryotes can be both cladistically closer to archaebacteria than to any other prokaryotes and cladistically closer to Planctobacteria than to any other eubacteria as Reynaud and Devos (2011) explicitly suggested. The rest of our paper highlights major merits of this revised neomuran theory in which Planctobacteria are substituted for posibacteria in the original version as the direct eubacterial ancestors of neomura. This is the best phylogenetic interpretation of the whole tree of life and offers more gradual and mechanistically more comprehensible transitions between the three domains than any previous scenario. The next two sections apply this to archaebacterial diversification and origin, later ones to eukaryotes.

A central feature of neomuran theory was the argument that N-linked glycoproteins arose in stem neomura at the very time of murein loss and that key involvement of N-acetylglucosamine (GlucNac) in oligosaccharide linkage to glycoprotein asparagines and to oligopeptides in peptidoglycan suggests that glycoprotein synthesis in part evolved from murein synthesis relics when the stem neomuran mutationally lost muramic acid biosynthesis and consequently murein peptidoglycan (Cavalier-Smith 1987c). Neomuran isoprenoid carrier dolichyl phosphate was argued to have evolved from the only slightly different eubacterial undecaprenol phosphate. Phylogeny of the 16 enzymes and the transmembrane flippase mediating eukaryotic N-linked glycoprotein synthesis shows that all have homologues in eubacteria, though a specific relative for Alg1 could not be identified (Lombard 2016). By contrast, only 9 of these 17 had homologues in archaebacteria; individual archaebacterial lineages had many fewer than that. Furthermore, almost all archaebacterial enzymes and both their contrasting flippase families have eubacterial homologues. That means (1) that eukaryotes could not have got their N-linked glycoprotein synthesis from archaebacterial ancestors but could have got it from a eubacterial ancestor like a planctobacterium and (2) that archaebacteria could also have got almost all necessary enzymes from eubacteria. The first three enzymes in the eukaryote pathway (Alg7, Alg14, Alg13) are homologues of the first two in eubacterial murein synthesis, MraY and MurG; Alg14 and 13 correspond to two halves of MurG. Thus, the first two enzymes of eubacterial murein synthesis were in fact taken over by eukaryotes (their descendants) and MurG split after eukaryotes diverged from archaebacteria. Most archaebacteria lack homologues of these enzymes, but *Sulfolobus* uses homologues of MraY and MurG for the first two enzymes for glycoprotein synthesis, retaining the ancestral unsplit version of Mur G as Saci1262. As flippase, eukaryotes use a homologue of the negibacterial Wzy-dependent flippase Wzx used in LPS synthesis (and some other negibacterial envelope structures), as does the euryarchaeote *Halobacterium*, whereas the euryarchaeote *Archaeoglobus* instead has a different flippase of the family (Wzt/Wzm) used by the LPS synthesis ABC-transporter as well as O-glycosylation in negibacteria and teichoic acid synthesis in Endobacteria—some negibacteria (e.g. *Escherichia coli*) use both flippase types for capsule synthesis.

The simplest interpretation of this is that the eubacterial ancestor that lost murein to make stem neomura was a negibacterium with murein and both types of flippases for LPS synthesis, and that when murein and OM (including LPS) were both simultaneously lost, some enzymes for murein synthesis and both flippases and some enzymes for LPS synthesis were retained for making neomuran N-linked glycoproteins. As eukaryotes and archaebacteria diverged, some enzymes/flippases were differentially lost in different descendant lineages. Stem eukaryotes lost Wzt/Wzm homologues but in archaebacteria flippase losses postdated crenarchaeotes. Numerous other eubacterial murein/LPS-making enzymes were differentially lost as different archaebacterial lineages evolved radically different surface structures: some retained glycoprotein S-layers (both in euryarchaeotes and filarchaeotes), some supplemented or replaced them by novel envelope molecules, e.g. pseudomurein in many euryarchaeotes, or more specialised molecules in more restricted lineages, e.g. *Thermoplasma* polysaccharide glycocalyx, sulphated heteropolysaccharide in *Halococcus*, halomucin in *Haloquadratum* (Klingl 2014). There is now little doubt that N-linked glycoproteins were ancestrally present in both archaebacteria and neomura and that their biosynthesis had a negibacterial ancestry. The earlier idea of an actinobacterial/posibacterial ancestry (Cavalier-Smith 1987c) is less likely as some key enyzmes have not been identified in endobacterial posibacteria (and fewer in actinobacteria) but their derivation from eubacterial ancestors is confirmed. Lombard (2016) adhered to the erroneous view that LUCA is in the neomuran stem and therefore failed to see that his results give extremely strong support to the idea of a eubacterial origin of neomura. Instead he interpreted them to mean that eukaryotes got these enzymes from numerous different sources—from archaebacteria and by LGT from many different eubacteria. This again shows how misrooting the tree makes evolution seem more complicated than it was. The here-modified neomuran theory with the root within negibacteria and a planctobacterial ancestry for neomura gives a far simpler picture: all neomuran flippases and glycoprotein-making enzymes could have come vertically from a planctobacterium after murein/OM/LPS loss.

Uniformity of eukaryote glycoprotein biogenesis and its retaining a much higher proportion of the eubacterial enzymes cannot be explained as simply by the old assumption of archaebacteria being ancestral to eukaryotes, whose sole evidence is often contradictory sequence trees some of which nest eukaryotes deeply within filarchaeotes (but in ever changing positions as successive papers are published). But differential retention of eubacterial enzymes is the natural consequence of the neomuran logic in which eukaryotes and archaebacteria diverged as sisters immediately after the origin of N-linked glycoproteins, core histones, more complex signal recognition particle (SRP), and other characters shared by eukaryotes and both archaebacterial phyla (Cavalier-Smith 1987c, 2002a, 2014), now including ESCRTIII membrane-scission proteins that became useful when murein growth could no longer divide the CM, as well as protein ubiquitination that was later coopted as a primary method of novel eukaryote cell cycle controls. If also euryarchaeotes and filarchaeotes diverged almost immediately after the first archaebacterium evolved isoprenoid tetraether lipids as an adaptation to hyperthermophily, as explained below, then the neomuran tree’s base is almost a star phylogeny in which eukaryotes, euryarchaeotes, and filarchaeotes diverged at almost the same time. Simulations show that truly star phylogenies can give high statistical support for false basal resolution (Yang 2007). Thus, the neomuran interpetation always expected the branching order of these three neomuran groups to be almost impossible to resolve with confidence, especially as the problem is exacerbated on many trees by ultrarapid evolution in the long eukaryote stem that destroys most relevant historical evidence and by the rapid early radiation of both archaebacterial subgroups and the extra long branches of some of them such as DPANN and some filarchaeotes.

By contrast, the alternative theory that LUCA is in the neomuran stem and archaebacteria are as old as eubacteria and that neomuran divergence took place in a mythical virtually precellular progenote (Martin and Russell 2003), if integrated with the strong fossil evidence that eukaryotes are at least three times younger than eubacteria would predict that eukaryotes should nest extremely shallowly and consistently within one archaebacterial subgroup with strong support and sequence trees would easily identify a specific archaebacterial ancestral lineage for eukaryotes. On the contrary, three-domain multiprotein sequence trees ( including all our RP trees) strongly disprove the idea that eukaryotes are substantially younger than archaebacteria, which would have to be true if archaebacteria were as old as eubacteria, contrary to all the evidence (Cavalier-Smith et al. 2018). The idea that LUCA was a precellular progenote and that archaebacterial and eubacterial membranes and walls originated independently (Sousa et al. 2013b, Fig. 1b) was always cell biological and evolutionary nonsense and is refuted above for walls and for membranes has been refuted by numerous papers showing that both major lipid types exist in archaebacteria and eubacteria (see below) and that numerous membrane proteins are shared between them by vertical descent—in particular, both have membrane-based respiratory systems and all four prokaryotic trans-membrane secretory systems: the Sec system (used by SRP secretion) for unfolded proteins, TAT system for folded proteins that both use class I signal peptides, and class II secretory system used by eubacteria for lipoproteins and archaebacteria for various enzymes (Soo et al. 2015a), and the class III signal peptides used for type IV pili. Thus, at the eubacteria/archaea transition, cells were advanced and fully prokaryotic in secretory mechanisms, not progenotes; eukaryotes by contrast lost type II and type III secretion, presumably when secretion became almost completely cotranslational during the origin of the rough endoplasmic reticulum (ER) (Cavalier-Smith 2009), which has no prokaryote equivalent—despite repeated earlier claims to the contrary in planctobacteria, now decisively refuted (Devos 2014a, b; Santarella-Mellwig et al. 2013). Enzymatic continuity across the eubacterial/archaebacterial divide applies not only to the glycoprotein and murein/LPS enzymatic relationship but also to membrane skeleton GTPases and ATPases involved in prokaryote growth and division, as the next section explains, making it nonsense to suppose that the transitional form was a progenote (Martin and Russell 2003). It was a bacterium billions of years younger than LUCA with highly complex cell envelope and rod-shaped structure, elements of which were conserved during the neomuran revolution despite destablising murein and OM/LPS loss.

Whether the planctobacterial ancestor had eubacterial flagella and lost them together with murein and OM or had already lost them (as commonly happened across eubacteria, e.g. in stem cyanobacteria) may never be determined. Either way, archaebacteria evolved archaella from duplicated type IV pili genes and eukaryotes evolved cilia and centrioles by duplicating planctobacterial tubulin genes in their stem lineages after their mutual divergence but before either of these sister groups evolved separate phyla. Thus, each of the three domains has a non-homologous major motility organelle that arose independently in their stem lineages—only bacterial flagella before LUCA. The contrasting evolutionary paths of diverging eukaryote and archaebacterial sisters stem from the fundamentally different nutritional modes they adopted. Archaebacteria evolved a new method of methanogenesis, we suggest from preexisting methylotrophic planctobacterial precursors, thus remained osmotophs like eubacteria and used glycoproteins to rigidify their S-layer and thus retained fundamentally prokaryotic DNA segregation and division machinery but lost planctobacterial mts, though three diverse archaebacterial lineages kept one tubulin for non-mt cytoskeletal functions; see later section). Eukaryotes evolved phagotrophy instead and thereby internalised their DNA/membrane attachment sites and so coopted planctobacterial mts to segregate their internalised chromosomes by mitosis and used planctobacterial membrane-coat proteins to make coated vesicles, giving an unprecedented method of cell growth, nuclear pores, and cilia—all three of which fundamental eukaryotic innovations depend absolutely on β-propeller/β-solenoid proteins known in prokaryotes only in Planctobacteria (Santarella-Mellwig et al. 2010, 2018). The need to study all these novel eukaryotic processes from the intracellular coevolutionary perspective emphasised by neomuran theory (Cavalier-Smith 2014) is shown by involvement of vesicle coat proteins in nuclear pore complexes and of nuclear pore proteins in protein transport into both nuclei and the ciliary compartment (Cavalier-Smith 2014) and in mitotic spindle mt assembly (Yokoyama et al. 2014). None of these could have evolved if eukaryotes evolved directly from archaebacteria (Cavalier-Smith 2014).

## Archaebacterial phylogeny and eubacterial ancestry

As in previous work, our archaebacteria-only trees suffer from the problem of DPANN having very long branches (the longest of all were omitted) so did not consistently resolve the question whether DPANN are a clade or are two distinct deep branches within euryarchaeotes. The simplest and biologically most plausible explanation is that DPANNs are not a separate clade but degenerate euryarchaeotes with miniaturised cells and highly reduced genomes that as a consequence of coding for many fewer proteins underwent faster than usual RP evolution, as suggested by Brochier et al. (2005). In eukaryotes also similar cellular and genic miniaturisation generated two lineages with ultrafast RP evolution: the rhizarian *Mikrocytos* and the major subclade of microsporidia. Our most convincing CAT trees suggest that such reduction occurred twice: once in halophiles to generate ‘Nanohaloarchaea’ that on our more credible trees are sister to Halobacteriales, and once in non-halophiles to make ‘Micrarchaea’, which we suggest are probably sisters of all euryarchaeotes other than Thermococcales. In most two- and three-domain trees, LBA to the very distant outgroup arguably artefactually pulls both ‘DPANN’ groups out from within euryarchaeotes, clustering them together as a distinct deep-branching false clade. Therefore, ‘DPANN’ is a mathematical artefact that should not be made a taxon. On this interpretation, the root of the archaebacterial tree lies between Euryarchaeota (including the two distinct ‘DPANN’ clades) and Filarchaeota (TACK and Asgard, probably sister clades). However, discovery of numerous novel archaebacterial lineages not included in our alignment and much contradictory evidence concerning rooting (Adam et al. 2017) means that much more study is needed of the difficult question of the archaebacterial root. A recent extremely thorough study using 278 proteins, rich euryarchaeote sampling, and heterogeneous as well as homogeneous methods convincingly shows that ‘Nanohaloarchaea’ do not group with ‘Micrarchaea’ but branch robustly within Methanocellia as sister to Methanocellales whereas other ‘Halobacteria’ are sister to Methanomicrobiales (Aouad et al. 2018). Thus, two related methanogen lineages gave rise independently to extreme halophiles and there were at least two independent euryarchaeal cell miniaturisations which have confused archaebacterial early phylogeny.

There is much evidence for differential gene loss within archaebacteria as well as massive gene loss during the origin of archaebacteria from eubacteria. Even non-DPANN archaebacteria typically have much smaller genomes than most eubacteria. Gupta (1998a) listed 40 genes shared by eubacteria and eukaryotes absent in archaebacteria, e.g. Hsp90, DNA polymerase I; it was argued that these and all eubacterial genes not found in neomura (e.g. enzymes making murein components muramic acid and diaminopimelic acid) were lost during archaebacterial origin (Cavalier-Smith 2002a). Archaebacterial genome sizes are generally lower than for eubacteria and much less than for eukaryotes—averaging 1.86 Mb in archaebacteria versus 2.61 Mb in eubacteria (Li and Du 2014), so many more genes were probably lost then. An earlier estimate inferred a 4–5-fold reduction in genome size (Cavalier-Smith 2007a), assuming 1500 genes in ancestral archaebacteria and 6000–8000 genes in a presumed actinobacterial ancestor. Though RP trees now rule out an actinobacterial ancestry, if the ancestor was a planctobacterium as argued below, stem archaebacteria probably did lose thousands of genes, for like Actinobacteria ancestral planctobacteria probably had larger genomes than average for eubacteria. Assuming the LACA encoded ~ 2000 proteins and its planctobacterial ancestor encoded ~ 6000 proteins, 4000 genes would have been lost. Planctobacterial genomes encode 5–10,000 proteins and Verrucomicrobia 5–7000. The highly reduced genomes of the endoparasitic Chlamydiia are irrelevant to the origin of archaebacteria.

Differential gene loss between major archaebacterial lineages is evident by comparing distribution of ancestral eubacterial genes, e.g. GTPase FtsZ (tubulin homologue) and ATPase MreB (actin homologue) and their relatives. Both form cytoskeletal filaments in the inner face of the cytoplasmic membrane with an ancestral function of shaping rod-shaped cells and spatially controlling growth of the eubacterial murein wall. MreB filaments guide longitudinal growth of peptidoglycan filaments, whereas FtsZ guides them during transverse septation and is tethered to the CM at the septal divisome by FtsA, another actin homologue that must have diverged from MreB and more distantly related Hsp70 ATPase before LUCA. When the ancestral murein wall was lost, triggering the neomuran revolution, FtsZ, MreB, and FtsA were lost by some archaebacterial lineages (notably Sulfolobia) but retained by others even after glycoproteins evolved—in marked contrast to eukaryotes that lost them. Their retention implies a similar retained function or new ones despite murein loss. Some or all of these ancestral cytoskeletal proteins were lost independently by most mycoplasmas when they lost murein, and MreB was lost in several walled bacteria that lost a rod shape. We therefore suggest they were retained by archaebacterial lineages that kept a rod-shaped growth form during the neomuran revolution (e.g. most euryarchaeotes) but were lost by lineages that modified their cell shape/division mode (notably Sulfolobia). Another eubacterial divisome protein SepF was also kept by almost all archaebacteria except Sulfolobia (Makarova et al. 2010). Our GenBank BLAST searches suggest that SepF is absent from Chloroflexi, but present in most Armatimonadetes, Melainabacteria, Cyanobacteria, Actinobacteria, and Endobacteria, but is retained only by some neonegibacterial lineages, being only sparsely present in many, notably Proteobacteria. In Actinobacteria, FtsA is absent, SepF having its FtsZ-tethering role and in Endobacteria, if FtsA is experimentally deleted, SepF can take it over. We suggest SepF evolved at the same time as LPS in the ancestor of all prokaryotes except Chloroflexi, i.e. in the ancestral glycobacterium. Scattered distribution of numerous proteins across archaebacterial lineages was called ‘puzzling’ (Adam et al. 2017). It is not, but easy to understand if we accept a high frequency of differential losses of their ancestral characters (both eubacterial and novel neomuran ones) as archaebacteria diversified explosively and restructured their cell envelopes and cell cycles immediately following murein loss and evolution of novel tetraether lipids and methanogenesis.

Ancestral archaebacteria were clearly rod-shaped walled cells using the same originally eubacterial proteins (FtsZ, FtsA, MreB, SepF) as most eubacteria to control wall growth and divison. As Greek *bacterion* means rod and they ancestrally retained the rod-making machinery, it was misleading and confusing to change their name from Archaebacteria to Archaea. It would have been more rational to have deleted the erroneous prefix archae and renamed them Metabacteria or Neobacteria (Cavalier-Smith 1989a) as they are (probably by billions of years) the youngest major prokaryote lineage. They are not a third form of life, but fundamentally prokaryotic. Contrary to Woese’s early writing, the absence of murein is no more significant than its secondary absence in endobacterial mycoplasmas. Their lipids are not uniformly unique: many have enzymes for making acyl ester lipids in addition to isoprenoid ethers and conversely some endobacteria have enzymes for making isoprenoid ethers in addition to acyl esters (Guldan et al. 2011; Coleman et al. 2019). Archaebacterial metabolism, regulation, population genetics, and ecology are essentially indistinguishable from those of eubacteria (Doolittle and Zhaxybaeva 2013). They are not the only prokaryotes that make methane as some eubacteria can do so by a simpler and probably older mechanism, e.g. cyanobacteria (Teikari et al. 2018)—even thaumarchaeotes have this mechanism, likely the most ancient one. Even reverse DNA gyrase once supposed to be unique to archaebacteria is widely found in thermophilic eubacteria (Brochier-Armanet and Forterre 2007), and a secondary adaptation to thermophily, and must have originated in eubacteria having arisen by fusion of two eubacterial genes (Cavalier-Smith 2002a); the sequence tree has a clear bipartition between eubacterial and archaebacterial sequences, consistently with vertical inheritance from eubacteria to archaebacteria and contrary to the authors’ assumption of multiple LGTs from archaebacteria to different eubacterial phyla (Brochier-Armanet and Forterre 2007). Not only was their cell growth and division machinery ancestrally fundamentally eubacterial, so is their DNA segregation which depends on P-loop ATPase ParA dimers, which were lost by eukaryotes when they evolved mitosis instead, whose spindle mts are almost certainly of eubacterial not archaebacterial ancestry (see below).

A major branch of euryarchaeotes retains eubacterial DNA gyrase as well as or instead of reverse gyrase, but being absent from filarchaeotes, it was assumed to have been acquired by a single LGT ‘from an unidentified [eu]bacterium’ (Raymann et al. 2014), but there is no good evidence for that. More likely, it was inherited vertically from Planctobacteria and lost independently by the ancestor of filarchaeotes and the other euryarchaeotes that lack it, which would need many fewer losses than the dozen or more that must be accepted for methanogenic enzymes if they were ancestral for archaebacteria as is generally accepted. The euryarchaeote sequences are nested within eubacteria, apparently as sister to Planctobacteria (though hard to be certain as some branches were confusingly collapsed on the tree). DNA gyrase and reverse gyrase are exceptions to the rule that archaebacterial DNA-handling enzymes are markedly different from eubacterial ones—a third one discussed below (where we explain why) is chromosomal SMC proteins.

Very few archaebacterial features are truly unique. Apart from a handful of relatively minor biochemical novel features, their flagella (archaella), tetraether lipids, and methanogenesis mechanisms are the only major properties of achaebacteria marking them out from both eubacteria and eukaryotes (Banerjee et al. 2015). Archaella evolved from type IV pili found in all eubacterial phyla (Berry and Pelicic 2015) so these precursors must predate LUCA; evolving archaella would have been no more complicated than making cyanobacterial phycobilisomes, and less so than oxygenic photosynthesis. Archaella proteins are mostly secreted as preproteins using class III signal peptides, cleaved by a signal peptidase distantly related to those used for eubacterial pili, their likely ancestor. FlaF the protein linking them to the S-layer has a strong structural resemblance to an S-layer protein of the endobacterium *Geobacillus* (Banerjee et al. 2015); we suggest FlaF evolved from a planctobacterial S-layer protein. Their unique flagella (archaella), different methanogenesis mechanism, and tetraether lipids would provide no reason for ranking archaebacteria collectively higher than a phylum.

## Planctochloran origin of archaebacterial lipids

It was long thought that prenyl ether lipids are unique to archaebacteria (even recently some mistakenly think such lipids unique to them: Caforio and Driessen 2017) and that they cannot make fatty acids. Some have even claimed that prenyl ether and acyl ester lipids that predominate in eubacteria and eukaryotes are incompatible in the same membrane and that these two types of membrane must have originated independently (Martin and Russell 2003). However, Cavalier-Smith (1987b, c) argued that all membranes had a common ancestor (which almost all now agree) and intermediates between eubacteria and archaebacteria must have had both lipid types and that the archaebacterial ancestor alone emphasised membranes of stabler prenyl ethers, especially tetraethers as a secondary adaptation for hyperthermophily and acidophily.

Three enzymes make archaebacterial membrane lipids: glycerol-1-phosphate dehydrogenase (G1PDH) which makes *sn*-glycerol-1-phosphate; geranylgeranylglyceryl phosphate synthase (GGGPS) which adds the first polyprenyl chain via an ether link; and digeranylgeranylglyceryl phosphate synthase (DGGGPS) which adds the second. All three have now been found throughout posibacteria and sphingobacteria (Coleman et al. 2019) and at least one, more often two, and sometimes three occur in all eubacterial phyla recognised here except Cyanobacteria and Aquithermota, but all three are missing in many subgroups, especially secondarily genomically reduced ones like mycoplasmas and chlamydias—they also appear missing in many DPANN archaebacteria and in lokiarchaeotes. It is therefore highly probable that many posibacteria and sphingobacteria and some other negibacteria can make so called archaebacterial lipids in addition to acyl esters and thus are realistic candidates from a lipid standpoint for eubacterial ancestors of archaebacteria.

Inserting archaebacterial lipids into the negibacterium *Escherichia coli* provides much experimental proof that archaebacteria-like glycerol-1-phosphate prenyl ether and eubacterial glycerol-3-phosphate acyl ester lipids are physiologically compatible (Jain et al. 2014). When G1PDH, GGGPS, and DGGGPS from the sphingobacterial cloacimonete are inserted into *E. coli*, it makes prenyl ether lipids indistinguishable from those of archaebacteria without growth impairment (Villanueva et al. 2018).

Conversely, most archaebacteria make fatty acids and a phylogenetically diverse scatter of them have one or both of the alternative enzymes for making the glycerol-3-phosphate backbone of acyl ester lipids (GpSA or GlpA/GlpD) and some have glycerol-3-phosphate acyltransferase PlsY that adds the first acyl group and some have 1-acylglycerol-3-phosphate O-acyltransferase (PlsC) that adds the second fatty acid chain (Coleman et al. 2019). Thus, there is no mechanistically necessary ‘lipid-divide’. Most discussions of ancient lipid evolution uncritically accept Woese dogma that LUCA was between eubacteria and archaebacteria and seem blissfully unaware of the better arguments and evidence based on integrated phylogeny and palaeontology for a eubacterial ancestry of archaebacteria (Cavalier-Smith 1987c, 2002a, 2006a, b) so make things more complicated than they need be (e.g. Jain et al. 2014; Lombard 2016; Villanueva et al. 2017; Sojo 2019). It is highly unlikely that glycerolipids evolved independently in eubacteria and archaebacteria as Sojo et al. (2014) speculated.

The simplest interpretation is that acyl ester lipids evolved as the main membrane lipids in the negibacterial ancestor, i.e. LUCA, which also made prenyl ethers as a minor stabilising component that was frequently lost in eubacterial lineages having other stabilisers like hopanoids, but became dominant only when the first archaebacteria colonised the hyperthermophily niche. By contrast, their eukaryote sisters lost prenyl ethers when they evolved phagotrophy, focusing instead on sterol stabilisers (also made from isoprenoids). Thus, prenyl ether lipids did not first evolve in archaebacteria but in ancestral eubacteria, archaebacteria inheriting them by vertical descent. It is wrong to call prenyl ether lipds ‘archaebacterial’ as they are general prokaryote properties absent in eukaryotes that have been also lost in many eubacterial sublineages and likely even in many DPANN archaebacteria and lokiarchaea (Villanueva et al. 2017; Coleman et al. 2019).

Lipids apparently unique to archaebacteria are C_40_-polyisoprenoid tetraether glycerolipids. Unfortunately, their biosynthesis is not understood (Jain 2014) so genetics cannot yet clarify the eubacterial ancestry of their biosynthetic enzymes. However, as C_20_ prenyl ether lipids were likely present in the planctochloran ancestor, this would have made tetraether origin a relatively simple step. We suggest that advantages of tetraether lipid monolayer membranes for increasing membrane thermal stability and decreasing proton leakage through them (Feyhl-Buska et al. 2016) provided the key selective force favouring the first archaebacterial invasion of the hyperthermophile adaptive zone immediately after the neomuran revolution when the OM was lost (and cotranslational synthesis of glycoproteins associated with extra rapid ribosomal evolution, and histones evolved). If tetraethers evolved once only, this would explain why archaebacteria alone lost ancestral acyl esters. Archaebacteria that later became mesophiles reverted to bilayer membranes for greater fluidity but could not readopt acyl esters: neither selection nor LGT can do anything useful without the right phylogenetic cellular precursors. Archaebacteria originated as an adaptive modification of a secondarily monoderm eubacterial derivative; they are not a non-adaptive leftover of early cell evolution, so tell us nothing about the origin of life or LUCA.

Trees for enzymes making acyl esters are extremely poorly resolved being virtually a star radiation, so cannot tell us whether archaebacteria simply kept planctobacterial enzymes in some lineages (as we suspect) or acquired them from eubacteria by multiple LGT as Coleman et al. (2019) claim—or possibly both for different lineages. Most eukaryote sequences group together weakly, but some are more scattered probably mainly because of unavoidable weak resolution for short proteins (Coleman et al. 2019; supplementary figs 13-15). These trees are entirely consistent with our thesis that archaebacteria are sisters of eukaryotes and got their acyl ester lipids by direct descent from planctobacterial ancestors; they support neither the widespread notion of eukaryote evolution from archaebacteria nor the speculation that their lipids came from the α-proteobacterial ancestor of mitochondria (Martin 1999), as eukaryote sequences do not nest within proteobacteria or within an archaebacterial clade; archaebacterial sequences are more scattered and do not form a major clade. For GpSA (159 amino acids), the main eukaryote clade is sister to a sphingobacterial sequence (from a latescibacterium) with 0.72 support. For Glp (190 positions), the main eukaryote clade groups with 0.57 support with 14 eubacteria (from eight phyla, including planctobacteria, whose sequence groups with 0.55 support with the eukaryote *Spironucleus*, possibly LBA) and one archaebacterium, a lokiarchaeote which seems misplaced as it fails to group with any of three main archaebacterial clades; for Glp, LGTs from eubacteria are more plausible than for GpSA. Unsurprisingly, the PlsC tree (53 positions) is too ill-resolved for sensible conclusions.

‘Archaebacteria-like’ enzyme trees though largely star radiations are slightly better resolved and a bit more illuminating. They provide no evidence whatever that eubacteria got their prenyl ether synthesis genes from archaebacteria. Coleman et al. (2019) attempted overoptimistically to root them by two outgroup-independent methods. They claim both put the G1PDH (190 positions) root either between eubacteria and archaebacteria or within eubacteria, though their Fig. 2 actually labels both roots between the two eubacterial clans and thus within eubacteria, where it was long thought to lie (Cavalier-Smith 1987b, c); the grouping of two small long-branch clades, one archaebacterial (from both phyla) and one eubacterial (entirely posibacteria), is likely a long-branch artefact. The main archaebacteria clade is no closer to posibacteria than to Planctobacteria. The simplest interpretation of this would be to accept a root within eubacteria for this enzyme and that archaebacteria evolved from a eubacterium by vertical descent, which they avoid—perhaps because they uncritically accept Woese’s mistaken, evidence-free view that both are ‘primary’ domains. GGGPS (129 positions) reveals two paralogues, one present in euryarchaeotes and posibacteria, the other in both archaebacterial phyla, posibacteria, sphingobacteria, planctobacteria (Elusimicrobia only), and a few uncultivated eubacteria. The simplest inference is that at least one paralogue had evolved before the last common ancestor of eubacteria (i.e. LUCA), as Chloroflexi have GGGPS not included in the tree), and both paralogues evolved before the common ancestor of posibacteria and neonegibacteria and were retained by many posibacteria and Planctochlora, being inherited vertically from the latter by archaebacteria but probably lost independently by Armatimonadetes, Cyanobacteria, Hadobacteria, Aquithermota, and eukaryotes. Additional losses of both or just one paralogue must also have occurred within phyla. DGGGPS (119 positions) reveals a long branch comprising Actinobacteria and three random archaebacteria plus a star radiation including both archaebacterial and eubacterial phyla except actinobacteria. Unsurprisingly, both rooting methods place the roots in the longest internal branches of the D/GGGPS trees, which is a phylogenetically meaningless expresssion of the fact that such long stems violate an implicit assumption of the methods (that degree of change is proportional to time), similar to the artefact that makes typical paralogue rooting of the tree of life completely misleading for all genes exhibiting an inflated neomuran stem (Cavalier-Smith 2002a, 2006c) and which misled Woese into inventing the profoundly misleading three-domain theory.

### Weakness of outgroup-free rooting

It is surely pointless applying these methods to such poorly resolved and biased single-gene trees. It is doubtful that they could give credible results for any single-gene trees or for any multigene ones with accelerated internal stems—like RPs. The authors correctly conclude that LUCA had prenyl ether lipid synthesising proteins (but incorrectly cling to the entirely unsupported idea that LUCA was between eubacteria and archaebacteria not a negibacterial eubacterium close to Chloroflexi). They probably exaggerate the amount of LGT and underestimate the frequency of loss. The absence of a clearcut bipartition or long stem between eubacteria and archaebacteria on any of their trees is yet another example of our thesis that metabolic enzyme genes generally are more clock-like and better indicate relative timing than those like RPs and rDNA that have temporally grossly misleading stretched neomuran or eukaryote stems (Cavalier-Smith 2002a). Two other outgroup-free rooting methods were applied to rooting concatenated rDNA trees which should be more resolving (Williams et al. 2015), yet biased. For the three-domain rDNA tree, the NR model put the root in the stretched neomuran stem, showing the same long-branch bias as most protein paralogue trees (and archaebacteria as sisters not ancestors of eukaryotes); contradictorily, the HB model (which performed more accurately on a small test case where the answer is known from taxon-rich outgroup trees) put the root (and LUCA) within the negibacterial eubacteria as we argue is correct but placed eukaryotes as sister to Filarchaeota, which we think incorrect. Both trees used only 16 sequences and had a grossly wrong topology for both eukaryotes and eubacteria so neither could possibly tell us where in eubacteria the root may be. For rooting archaebacteria, which should be easier as there is no transiently hyperaccelerated internal stem and more reliable as they used 30 taxa, HB put the root between Filarchaeota and Euryarchaeota (inluding DPANN) exactly as we argue is probably correct.

Archaebacteria are not a third form of life, merely specialised ancestrally hyperthermophilic bacteria that arose independently of Aquithermota and Thermotogia and unlike them ancestrally ceased to use acyl esters in their membranes, and whose neomuran ancestors lost OM and murein, evolved histones with repercussions on DNA-handling enzymes, and modified SRPs and ribosomes to focus on cotranslational protein secretion (Cavalier-Smith 2002a). What separates archaebacteria from other prokaryotes is not their truly unique features, which are very few—no more than those distinguishing eubacterial phyla like Cyanobacteria, Endobacteria, or Planctobacteria—but that they share many ribosomal and DNA handling properties plus core histones with conserved nucleosomal organisation (Mattiroli et al. 2017), and N-linked glycoproteins more closely with eukaryotes than with eubacteria. Their uniqueness lies primarily in a unique combination of non-unique properties: ancient eubacterial and derived neomuran ones that arose in stem neomura billions of years after primordial characters shared with eubacteria evolved. That sharing and seeming character mosaicism is not a consequence of great antiquity (Cavalier-Smith et al. 2018) nor of chimaerism, but of Archaebacteria being younger than eubacteria, retaining most characters with little change but radically altering others, creating a mosaic of ancient and modern characters as in *Archaeopteryx*. Lipid evolution enzymes provide no evidence for archaebacteria being as old as eubacteria or being ancestral to eukaryotes.

Though total replacement of archaebacterial lipids by mitochondrial lipids as suggested by Martin (1999) is mechanistically implausible and would probably encounter some lipid/protein incompatibilities, we do not agree with Sojo (2019) that protein-lipid mismatch is the main reason for the ‘lipid-divide’, which is primarily a simple phylogenetic accident resulting from the facts that cells ancestrally had membranes largely of acyl esters and that (almost as ancient) prenyl ethers remained a minor constituent (often lost) in all lineages, until stem archaebacteria became hyperthermophiles. Lineages that lost prenyl ethers may never have regained them if other lipids gave sufficient stability. There is no reason to suspect that LGT of lipid synthesis is so rampant and the selective force for replacing them would be so great that one expects LGT to completely replace existing lipids. Nor is there any reason to think that LGT was perpetually ‘trying’ to introduce photosynthesis into archaebacteria and that lipid incompatibility is why it failed, as Sojo imagines (LGT of photosynthesis is far rarer and harder within eubacteria than he assumes; as argued above it may never have been thus acquired by a heterotroph and perhaps only once or twice by replacement). Sojo is trying to give an unnecessary explanation for a non-problem—phylogenetic inertia that pervades all evolution: stabilising and purifying selection generally keep most things essentially the same and LGT hardly ever makes really drastic changes (symbiogenesis can, but extremely rarely: Cavalier-Smith 2013b); moreover, complex characters depending on many genes hardly ever evolve twice in the same way. In our view, there was only one major loss of acyl ester membrane lipids in the history of life and there are no proven examples of wholesale lipid substitution by LGT or sound reasons to expect it.

## LGT from chloroplasts to ‘Cenarchaeales’ supports archaebacterial recency

Widespread assumptions that archaebacteria are ancient are solely based on ribosomal and protein paralogue trees dominated by misleading long-branch artefacts (Cavalier-Smith 2002a, 2006c). An LGT of the DNAJ-Fer protein from chloroplasts of Viridiplantae into stem ‘Cenarchaeales’ (Petitjean et al. 2012) within thaumarchaeotes (best considered a class of archaebacterial phylum Filarchaeota (Cavalier-Smith 2014)) is important evidence for archaebacteria being the youngest bacteria. If correct and a single ancestral transfer, it proves that crown ‘Cenarchaeales’ are younger than Viridiplantae. If Viridiplantae are ~ 740 Ma (as we estimate from the eukaryote part of the RP tree), we can use that to set an upper bound to the age of archaebacteria. We first calculate an upper bound for the date of the euryachaeote/filarchaeote cenancestor using the ratio of ‘Cenarchaeales’ crown depth to the distance between its crown base and the euryarchaeote/filarchaeote ancestor on Fig. 10. That gives ~ 1.18 Ma as an upper bound for euryarchaeote/filarchaeote divergence. If DPANN are genuinely older than Euryarchaeota, we get ~ 1.26 Ga for the crown archaebacterial age, but if they really belong within euryarchaeota (as our more convincing one- and two-domain trees suggest) that figure would be inflated. As these are upper bounds, archaebacteria are likely younger. A date of ~ 1 ± 0.15 Ga for neomura and archaebacteria would be concordant with all the most obvious fossil and sequence tree evidence.

These calculations are consistent with earlier arguments (Cavalier-Smith 2006a) against assuming that the ~ 1.45 Ga increase in fossil cell size signifies stem eukaryotes (Cavalier-Smith 1990) not large prokaryotes. Late divergence of stem eukaryotes very close to the base of crown archaebacteria in RP trees disproves the idea that archaebacteria are 2–3 times older than eukaryotes, which would have to be true were archaebacteria as old as eubacteria. If the Fig. 9 position of the neomuran stem as sister to Lokiarchaeota were correct (unlikely), its fractional depth compared with total depth from the base of crown archaebacteria (accepting DPANN early) to the mean of the lokiarchaeote branch tips represents a divergence at 86% of the depth of crown archaebacteria, i.e. an upper bound of 1.06 Ga for stem eukaryotes. Though one cannot date crown archaebacteria directly from fossils, that relative depth (assuming uniform substitution rates between archaebacterial crown base and lokiarchaeote tips) makes crown archaebacteria only ~ 1.16× older than stem eukaryotes. Comparably recent ages would be deduced if we used the likely more reliable two-domain trees that do not group eukaryotes with Asgard archaea.

## Evolution of eubacterial and archaebacterial methanogenesis and methylotrophy

Methanogenesis genes in novel filarchaeote subgroups ‘Bathyarchaeota’ (Evans et al. 2015) and ‘Verstraetearchaeota’ (Vanwonterghem et al. 2016; Berghuis et al. 2019) now imply that methanogenesis (otherwise predominantly in euryarchaeotes, also phyletically more diverse than once thought: Borrel et al. 2019) evolved before the last archaebacterial common ancestor (LACA) and was lost in lineages that lack it. Evidence from cytochrome oxidase phylogeny discussed below implies that before methanogenesis evolved stem archaebacteria were facultative aerobes, and that LACA was an adaptable organism that could switch between aerobic respiration and anaerobic growth using methanogenesis, but most archaebacterial lineages became more specialised by losing one of these. Discovery of eubacterial methanogenesis (Teikari et al. 2018) using different mechanisms from archaebacteria overturns the classical assumption that methanogenesis is unique to archaebacteria or first evolved in them but does not alter the likelihood that archaebacterial methanogenesis evolved from eubacterial methylotrophy, we suggest specifically from their likely planctobcterial ancestors.

This relatively recent ~ 1 Ga age for crown archaebacteria makes methane generated by archaebacterial methanogens entirely irrelevant to Palaeoproterozoic and Archaean climates, as previously argued (Cavalier-Smith 2002a, 2006a). Irrespective of whether eukaryotes are sisters of archaebacteria (most likely, as Forterre (2015) also argues, correctly distrusting the inconsistent trees that suggest otherwise) or branch within but close to the base of crown archaebacteria, archaebacterial methanogenesis cannot be much older than eukaryotes. The likely absence of archaebacterial methane on early earth makes it unwise for palaeoclimatologists to rely on biogenic methane for solving the problem of why with an early faint sun there was not permanent global freezing (Haqq-Misra et al. 2008; Pavlov et al. 2000). Assuming archaean archaebacterial methanogenesis is phylogenetically incorrect, and climatologically unnecessary—other ways can solve the faint sun paradox, e.g. high carbonyl sulphide (OCS) levels in the Archaean atmosphere for which there is recent sulphur isotopic evidence (Ueno et al. 2009). Climatologists need to address the faint sun paradox primarily with CO_2_, OCS, and water vapour as greenhouse gases with minor contribution from abiogenic methane, for whose abiotic synthesis several plausible mechanisms exist (Sherwood Lollar and McCollom 2006); abiotic mechanisms, e.g. serpentinisation (McCollom 2016) are the major source of Archaean methane in the latest atmospheric model (Laakso and Schrag 2017).

^13^C/^12^C ratios in some late Archaean kerogen samples (~ 2.7–2.8 Gy ago) that are unusually light (Pavlov et al. 2001) are often cited as evidence for archaebacteria being that ancient. However, Hayes (1994), who first suggested that such light kerogen might in principle have been produced by a two-stage carbon isotope fractionation, first by methanogenesis then by methano- or methylotrophy, called this interpretation a ‘speculative hypothesis’ and based it in part on the erroneous assumption of Woese and Fox that methananogenic archaebacteria are as ancient as eubacteria. Given that in principle an ecosystem comprising only eubacteria could produce similar ^13^C-depletion in several different ecological scenarios involving two-step fractionation (Strauss et al. 1992) and that inorganic means of fractionation also exist (McCollom 2013, 2016), it is incorrect to cite these data as ‘evidence’ for archaean archaebacteria. They are a geochemical observation needing explanation, which is difficult as so little is known about biology, ecosystems, and biogeochemical cycles then. Before the GOE, atmospheric hazes in principle could develop at appropriate CH_4_/CO_2_ ratios and such atmospheric processes would have been capable of causing these light kerogens (Pavlov et al. 2001). 3.5 Gya ^13^C-depleted fluid inclusions also were claimed as evidence for early methanogens (Ueno et al. 2006), but hydrothermal processes could have generated both examples of low ^13^C/^12^C ratios (Sherwood Lollar and McCollom 2006) and abiotic mechanisms can provide as broad a range of ^13^C/^12^ C as can archaebacterial methanogenesis. Estimates of the likely abiogenic methane flux are conflicting (sometimes suggested to have been as great as biologically nowadays), but it is premature to rule out other explanations than the classic Hayes hypothesis. A key point is that the most ^13^C-depleted ratios could have been produced biologically only by two stage enrichment in ^12^C. Neither RuBisCo nor autotrophic methanogenesis can do that in one stage, so biological explanations are based on suppositions of local recycling out of atmospheric equilibrium. Fractionation by archaebacterial methanogenesis alone depends on carbon source: if acetate (now quantitatively most important but restricted to Methanosarcinaceae and Methanosaetaceae) the most ^13^C depletion is *less* than with RuBisCo; for autotrophic CO_2_/H_2_, it is very variable with species and can be as low as for RuBisCo or much higher; if methanol, it is greatest. However, ability to use methanol and thus generate the strongest fractionation is taxonomically restricted to Methanosarcinaceae and *Methanosphaera* (Penger et al. 2012), both relatively recently evolved taxa (Brochier-Armanet et al. 2011), and unlikely to have been the ancestral method; this heterotrophic method could not have been the basis for an extensive ecosystem. If the archaebacterial root is between euryarchaeotes and filarchaeotes, the most likely ancestral methanogenesis mode would be reduction of methyl compounds by hydrogen as in Methanomassiliicoccales (Borrel et al. 2014) and ‘bathyarchaeotes’; as this is not intrinsically autotrophic, this is consistent with our next section arguing that ancestral archaebacteria were facultative aerobes with at least two, more likely three modes of energy generation between which they could switch and that differential losses as soon as they diversified created more specialist lineages. Nowadays, all methanogens are strict anaerobes, and most strictly dependent on methanogenesis but at least two lineages can also live by fermentation and in bathyarcheotes, there is evidence for recent losses of methanogenesis and reversion to fermentation via the Wood-Ljungdahl pathway (Borrel et al. 2016), but most methanogenic lineages lost methanogenesis early on and were never able to regain alternative modes of energetics. A small subclade of *Methanosarcina* relatively recently replaced standard acetoclastic methanogenesis dependent on acetyl-Co synthetase by a novel higher-throughput version using acetate kinase and phosphoacetyl transferase by getting these adjacent genes (present in no other archaebacteria) by LGT from cellulosolytic Clostridiia (Fournier and Gogarten 2008); it seems less likely that the host for the LGT previously used one of the other methanogenic mechanisms known in Methanosarcinales. Rothman et al. (2014) dated the LGT to ~ 250 Ma, but as a later section explains unjustified assumptions seriously inflated that age and associated ‘dating’ of archaebacterial methanogenesis.

The original Hayes model involved global aerobic methanotrophy as a second step (Hayes 1994), but as evidence against a strongly oxidising atmosphere (apart from the extreme upper atmosphere) prior to the GOE is now stronger than then (Farquhar et al. 2007), it could be argued that anaerobic methanotrophy might have evolved before the GOE, but aerobic methylotrophy only afterwards. Aerobic methanotrophy is restricted to the α- and γ-Proteobacteria and to the verrucomicrobial branch of Planctobacteria (Sharp et al. 2012, 2014); as all are subclades of Gracilicutes (Fig. 5), aerobic methanogenesis most likely originated in the gracilicute cenancestor, which if Fig. 5 is correctly rooted evolved significantly after Cyanobacteria and thus after the GOE. Denitrifying eubacteria of clade NC10 (‘*Methylomirabilis*’) can oxidise CH_4_ anaerobically in the absence of archaebacteria (but in the presence of miscellaneous eubacteria) by generating their own oxygen by splitting nitric oxide (Ettwig et al. 2010); as no external O_2_ is needed, such methanotrophy might in principle have used abiotic methane and have provided extra-light carbon to photosynthetic bacteria to generate extra-light hydrocarbons (Ettwig et al. 2010). However, as NC10 is a subclade of Proteobacteria sensu lato (Chistoserdova 2016), they probably evolved only after cyanobacteria if Fig. 5 is correctly rooted, so unless this mechanism also occurs in deeper-branching eubacteria, it is not a plausible explanation for 2.7 Gya light carbon. Anaerobic methanotrophy is also done by relatives of methanogenic archaebacteria using the methanogenic pathway in reverse, but only syntrophically in the presence of sulphate-reducing eubacteria. If archaebacteria arose only ~ 1 Gya, this also could not have generated that ancient light C signal. It is also questionable whether there would have been enough sulphate or N_2_O before the GOE to serve as oxidants for either mechanism of anaerobic methanotrophy.

## Stem Archaebacteria were facultative respirers

If archaebacteria are not significantly older than ~ 1 Gy, it follows that they arose from a eubacterial ancestor and diverged from it long after the GOE, i.e. billions of years after the origin of photosynthesis and aerobic respiration. Like most eubacterial phyla, archaebacteria comprise a mixture of interspersed anaerobic and aerobic lineages. Were they ancestrally anaerobic or aerobic? Phylogeny of respiratory electron transport chains and terminal oxidases makes it highly likely that all were ancestrally aerobic (likely facultatively) and that obligate anaerobiosis was secondarily derived many times independently by multiple gene losses. Every prokaryote phylum includes lineages having copper-containing terminal oxidases and a cytochrome (=Cyt) *bc* respiratory complex (either Cyt *bc*_*1*_ as in mitochondria or Cyt *b*_*6*_*f* as in chloroplasts, or both) and also other lineages in which both are absent. Sequence phylogeny of these respiratory proteins shows the same major clusters as do RP trees and that their relative branching order largely is as congruent as can be expected for single gene trees with RP multiprotein trees. That means that inheritance of both was largely vertical with LGT playing little or no role in the overall pattern (Brochier-Armanet et al. 2009; Dibrova et al. 2013, 2017), despite possibly occurring for some minor paralogues.

The simplest interpretation of this is that LUCA had a respiratory electron transfer chain based on cytochrome *bc* and a Cu-cytochrome terminal O_2_ reductase and differential loss of both led to numerous independent evolutions of fermentative obligate anaerobes in every phylum except cyanobacteria. Additionally, every well-defined phylum except spirochaetes contains lineages with a non-homologous terminal O_2_ reductase comprising a cytochrome *bd* (Cyt *bd*) complex using FeS or haem electron carriers not Cu-cytochromes. Its phylogeny is also congruent with almost exclusive vertical inheritance, so LUCA arguably had two distinct terminal O_2_ reductases which were differentially sorted during radiation of prokaryote phyla. This well fits the idea that LUCA was a photosynthetic negibacterial eubacterial cell and that both photosynthesis and respiration were differentially lost many times, but inconsistent for example with the speculation that different branches of Oxybacteria evolved aerobic respiration independently merely because of their different cytochromes (Soo et al. 2019). Electron transfer chains probably first evolved for anoxygenic photosynthesis and the earliest heterotrophs would have included both fermenters and anaerobic respirers with a diversity of electron sinks. Before cyanobacterial oxygenic photosynthesis and GOE, there would have been too little O_2_ for extensive aerobic respiration, but could have been enough to support very low levels at least locally or transiently (Haqq-Misra et al. 2011). The presence of apparently vertically inherited terminal oxidases in all putatively earliest diverging lineages is most simply explained if they initially evolved in LUCA primarily as a protectant against harmful effects of even low levels of abiotic O_2_; only after GOE would they have been able to become a quantitatively major source of ATP or reducing power for lipid synthesis. If first cells were fermenters as Haldane and Oparin assumed, primitively fermentative lineages must have died out before LUCA, contrary to assumptions by those who erroneously believe the root of the universal tree to be in the neomuran stem, that archaebacteria are as old as eubacteria, and that primitively anaerobic prokaryote lineages lacking electron transfer chains still exist (e.g. Weiss et al. 2016). The assumption that aerobic respiration evolved polyphyletically only after Cyanobacteria (Fox et al. 1980; Soo et al. 2017) is probably incorrect, but it remains likely that it was not a major source of energy before then.

Dibrova et al. (2017) highlight that the Cyt *b*_*6*_*f* complex characteristic of Cyanobacteria and the endobacterial Heliobacteria has short Cyt *b* with subunit IV a separate protein (coded by a distinct gene in the same operon), whereas Cyt *bc*_*1*_ of Proteobacteria, Chlorobi, and most non-photosynthetic phyla (e.g. Actinobacteria, Planctobacteria, Aquithermota) have a long version of Cyt *b* where in most lineages the distal extension is clearly homologous with subunit IV. They reasonably suggest that the short Cyt *b*_*6*_*f* condition is ancestral and evolved for photosynthesis and that the longer version evolved by fusion of adjacent *cyt b* and subunit IV genes. They say several independent fusions are needed to explain their data, but if Fig. 5 RP tree is correct, only two independent fusions are needed—one at the base of Actinobacteria and one at the base of the Neonegibacteria/neomura clade.

Dibrova et al. (2017) argued that the Cyt *bc* complex evolved in photosynthetic eubacteria and was ‘later aquired by’ archaebacteria. They appeared to believe acquisition was by LGT; that seems true for Halobacteriales (for which there is much evidence for eubacterial gene acquisition by LGT) but is likely mistaken for the major clade of archaebacterial Cyt *b* comprising euryarchaeotes and Sulfolobia. This and the seemingly separate thaumarchaeote/Korarchaeum and Aigarchaea clades (which group together but with an insignificantly supported intrusion of a miscellaneous eubacterial long-branch clade) uniformly have the long Cyt *b* version. By contrast, halobacteria form two quite separate clades: one mixed with Actinobacteria (which might have donated their genes) has long Cyt *b* (this joint clade is sister to Hadobacteria), whereas the other more distant halobacterial clade has short branch Cyt *b* with adjacent separate subunit IV in its operon, whose structure suggests LGT from Endobacteria. Apparently, Halobacteriales obtained their Cyt *b* by two independent LGTs, one from Actinobacteria, one from Endobacteria. But the tree provides no evidence for LGT into *non-halobacterial* archaebacteria and is fully compatible with vertical descent—the tree used neighbour joining, an inferior method to PhyloBayes CAT that would be more troubled by long-branch artefacts; one cannot expect that method applied to a single short gene to identify the eubacterial ancestor of archaebacteria—indeed the basal part of their tree and thus the relative branching order of the main phyla is totally unresolved. However, both protein and operon structure are consistent with the RP trees suggesting neomura may have evolved from Planctobacteria. Thus, Dibrova et al. are probably correct in arguing that Cyt *b* evolved in eubacteria and archaebacteria acquired them later—ancestrally by vertical inheritance, but apparently twice independently by LGT by Halobacteriales only. Whether the other long-branch paralogues (clades GHL) also represent LGT or are simply artefactual long-branch pseudoclades (more likely) might be established by PhyloBayes CAT analysis but is not germane to the question of archaebacterial origin from eubacteria.

Phylogeny of Cu-cyt O_2_ reductases is complicated by there being three distinct subfamilies (A–C), each in several phyla, and all being as closely related to nitric oxide reductase (NOR) as to each other. We agree with the interpretation that paralogue A found in all prokaryote phyla is likely ancestral and that NOR (largely restricted to Proteobacteria) probably evolved secondarily from an O_2_ reductase (Brochier-Armanet et al. 2009; Dibrova et al. 2013, 2017), and therefore should not be used to root the overall tree as is sometimes done. We also agree that paralogue B likely evolved in Sulfolobia and was transferred by LGT to various eubacteria and (perhaps via some of them) to Halobacteriales, but we think that in addition, multiple B paralogues must have evolved early in Sulfolobia (more than one being retained by some species) and LGT occurred from at least two different C paralogues. We agree that paralogue C (mainly in Proteobacteria) is almost certainly not ancestral. However, their suggestion that it evolved in Proteobacteria and was laterally transferred independently to several other phyla is less likely than the alternative that it evolved from paralogue A by gene duplication at the base of the gracilicute clade and underwent immediate gene duplication to make two C paralogues that were differentially lost during divergence of the four gracilicute lineages (Proteobacteria, Planctobacteria, and Bacteroidetes/Chlorobi retain both paralogues but the spirochaete *Leptospira* only one). That interpretation needs many fewer LGTs—just one to *Synechococcus*, one to *Symbiobacterium*, and probably a third from a proteobacterium to *Salinibacter* (Bacteroidetes). Therefore, only paralogue A is directly relevant to the transition between eubacteria and archaebacteria. In our view, LGT was also less frequent than they assume for that paralogue. More than one paralogue of catalytic subunit version A seems to have evolved in some groups (probably three in Proteobacteria, two in Endobacteria (Brochier-Armanet et al. 2009; Dibrova et al. 2013, 2017)); these comments refer to Brochier-Armanet et al. (2009), which conservatively included 401 positions—another study using 529 positions but sampling many fewer phyla oddly showed four actinobacterial clades (Soo et al. 2017) not one, and may be less reliable). Thus, multiple Cu-cyt O_2_ reductase paralogues evolved relatively early in eubacterial evolution; much of the seeming noncongruence with the simple RP tree stems from this coupled with differential loss amongst paralogues as lineages diversified. If that is accepted, very few cases of LGT need be invoked, notably one from an α-proteobacterium to the planctomycete *Rhodopirellula*, one from a δ-proteobacterium to *Leptospira*, and another from a proteobacterium to *Chloroflexus aurantiacus*. In contrast to these clear examples of LGTs between eubacteria, there is no evidence of LGT from or to archaebacteria. All archaebacterial sequences (12 euryarchaea, 7 Sulfolobia, 2 Nitrososphaeria) form one clade, albeit with insignificant bootstrap support (22% on ML tree). As the tree has no significant basal resolution, we cannot decide the closest eubacterial relative, but it is consistent with vertical evolution and presence of the A paralogue in the ancestral archaebacterium.

Mechanistically, the A-family is more efficient at proton pumping and has two proton channels (N and K), but can only work in high O_2_ levels. The B- and C-families have only the K channel but can work in low pO_2_; it is argued that they independently lost the N channel as adaptations to lower O_2_ levels (Han et al. 2011). On this interpretation, the immediate ancestor of archaebacteria originated after the GOE and was ancestrally a facultative aerobe able to cope with high O_2_ level and the B-family probably evolved in an early sulfolobian as secondary adaptation to low O_2_.

## Eubacterial origin of archaebacterial cell cycles

Most archaebacterial cell cycle properties are typically prokaryotic, but histone origin led to radical changes in DNA replication machinery (substitution of DNA polymerase III by a repair polymerase and major modifications to the clamp and to the replication fork helicases giving them radically different sequences but conserving fundamental 3D structure). In all organisms, DNA initiation proteins are central to cell cycle regulation by linking replication initiation to growth and termination to division (Scholefield et al. 2011). Eubacterial DnaA (recognising the replication origin DNA locus oriC) and neomuran ORC (origin recognition complexes) are AAA+ ATPases (Iyer et al. 2004) with exactly the same domain structure and similar roles despite great sequence divergence and different names (Costa et al. 2013). Archaebacterial ORC is a single protein that binds to specific oriC-like DNA regions, and interacts with DNA polymerase (Zhang et al. 2009), as does DnaA, thus specifically prokaryotic. By contrast more advanced and complex eukaryote cell cycles have duplicated the ATPase and have a heteromeric ORC of six different proteins, only three being AAA+ ATPases, and thousands of DNA replication origins that lack the prokaryotic sequence specificity (Scholefield et al. 2011). ORC functions by recruiting the replicative DNA helicase ATPase Mcm to the replication fork where it actively separates parental DNA strands to serve as single-standed templates (Shin et al. 2007). Mcm is a ring-shaped hexamer—hexahomomeric in archaebacteria, heteromeric with six different paralogous subunits in eukaryotes (Liu et al. 2009). Eubacterial replicative helicase DnaB though also a homohexameric DNA-dependent ATPase is not in the AAA+ superfamily, so probably not ancestral to Mcm, but appears more closely related to the RecA DNA recombinase, the hexameric RecA/DnaB family probably sharing a common ancestry with the also hexameric ATP synthesising F1 ATPase than with AAA+ ATPases (Leipe et al. 2000). Leipe et al. (2000) thought that DnaB evolved from RecA as they mistakenly believed the universal root to lie in the neomuran stem. But if it is beside Chloroflexi (or anywhere else within eubacteria), the simpler interpretation is that DnaB is ancestral to RecA and was lost by LACA after functionally equivalent Mcm took over helicase function in stem neomura. DnaB is smaller, thus simpler than RecA so should have been easier to evolve; its essentiality for replication is biologically more fundamental than recombination and more likely to have evolved first. One can hardly have had a viably replicating DNA chromosome without a helicase that would have been a prerequsite for the origin of more complex RecA. If eukaryotes are sisters of archaebacteria, not derived from them, they could have vertically inherited their DnaA/DnaG primase gene fusion from a bacteriophage infecting the planctobacterial ancestor of neomura, without needing to invoke a bacteriophage to eukaryote LGT as did Leipe et al. (2000).

The annular sliding clamps that ensure replication processivity of all cells have a sixfold pseudosymmetry with identical protein folds even though homodimeric in proteobacteria but homotrimeric in neomura; presumably adjacent gene boundaries changed during the neomuran revolution. They are loaded onto DNA by fundamentally similar DNA-dependent AAA+ ATPase clamp loaders (Costa et al. 2013); the DnaA/ORC family diverged from the clamp-loader one by preLUCA gene duplications (Iyer et al. 2004); five other AAA+ families produced by preLUCA duplications including the eubacterial ancestors of Mcms are more closely related (by sharing a a Helix 2 insert) to the eubacterial MoxR family of eubacterial protein chaperones from which dynein probably evolved in the eukaryote stem (Iyer et al. 2004). Given these and other fundamental similarities between eubacterial and archaebacterial DNA replicative proteins, the idea that DNA replication evolved independently in eubacteria and neomura close to the origin of life (Koonin 2006; Forterre 2015) is completely untenable, as well as being refuted by the recency of neomura and inconsistent with apparent vertical inheritance of reverse DNA gyrase between eubacteria and archaebacteria (see above). Moreover, purely RNA genomes could not have retained the replicational fidelity to maintain the highly conserved nature of the 1500 or more genes inferred to have been present in the neomuran stem. Large changes in replication consequential on the evolution of histones can simply explain divergence of neomuran from the simpler ancestral eubacterial system without having to accept Woese’s refuted idea of archaebacterial antiquity—unfortunately that persistent but erroneous paradigm continues to mislead interpretations by many.

Also shared by some archaebacteria with eukaryotes to the exclusion of eubacteria are homologues of the ESCRTIII protein Snf7 involved in membrane scission and of the AAA+ ATPase VPS4, which in eukaryotes disassembles ESCRTIII complex allowing its recycling (Makarova et al. 2010). Snf7 and VPS4 are found in many Filarchaeota, and a few phylogenetically scattered euryarchaeotes, so were clearly present in the ancestral archaebacterium and were lost by numerous lineages, e.g. by Thermoproteales in Sulfolobia. Irrespective of whether eukaryotes are sisters of archaebacteria (as we and Forterre (2015) argue) or are derived from early archaebacteria as some trees (heavily criticised by Forterre 2015) suggest (Spang et al. 2018; Williams et al. 2012, 2013; Zaremba-Niedzwiedzka et al. 2017), it follows that some functions related to ESCRTIII membrane scission arose in the neomuran stem but were lost by numerous archaebacterial lineages. As membrane scission during eubacterial division was by murein growth, it is unsurprising that new mechanisms had to be found when murein was lost. More than one new mechanism probably evolved, likely including scission by actin-like filaments and by ESCRTIII coiled-coil filaments. Given redundancy of scission mechanisms, different ones were kept in different lineages and others lost as neomuran cytoskeletons and walls diversified: for example within Sulfolobia Thermoproteales evolved crenactin but lost ESCRTIII whereas Desulfurococcales and Sulfolobales lost actin homologues (Makarova et al. 2010) but kept ESCRTIII—known to be used for *Sulfolobus* cytokinesis and budding (Liu et al. 2017). All Sulfolobia lost FtsZ, SepF, MreB, and FtsA so more radically replaced eubacterial cell growth and division proteins than did other archaebacteria, most of which retained FtsZ/FtsA/SepF (almost all euryarchaeotes, some thaumarchaeotes, Korarchaeum, and Asgards), and some of which kept MreB. As the mostly rod-like euryarchaeotes kept most of the eubacterial division machinery, most probably lost actin, except for the wall-less *Thermoplasma* (Hara et al. 2007).

Eukaryote F-actin has two helical parallel protofilaments, thereby differing from MreB that has two antiparallel protofilaments and the more structurally divergent paralogue FtsA with just one protofilament (Wagstaff and Löwe 2018). MreB and FtsA must have diverged from each other and from much longer Hsp70 that shares the same ATPase fold before LUCA. Neomuran actins group on sequence trees more closely with eubacterial filament proteins MamK and ParM, both with two parallel protofilaments, than to MreB (Hara et al. 2007; Lindås et al. 2014; Yutin et al. 2009). MamK makes the filament of magnetotactic proteobacteria (Lefevre et al. 2013) that supports the magnetosome, a bag-like invagination of the CM containing magnetic greigite or magnetite crystals (Grant et al. 2018), also found in the planctobacterial ‘Omnitrophica’ lineage (Lefevre et al. 2013). More widely distributed ParM filaments that segregate plasmids in many Proteobacteria and Endobacteria have homologues in many other eubacteria (e.g. Cyanobacteria, Chloroflexi) and some euryarchaeotes. Actin of the secondarily wall-less euryarchaeote *Thermoplasma acidophilum* has 2 parallel protofilaments and is so much more structurally similar to eukaryote actin than is MreB that it can be regarded as a true actin. It is also structurally much closer to ParM than to MreB (Hara et al. 2007; Lindås et al. 2014). Crenarchaeote actin also groups more closely with eukaryote actin than with MreB, but though structurally like actin has a long insertion that stops it forming adjacent protofilaments so exists as one protofilament. The simplest evolutionary interpretation is that ancestral neomuran actin was double stranded with parallel protofilaments and it evolved from a planctobacterial MamK ancestor, but stem Sulfolobia evolved single-stranded crenactin by an insertion mutation. ParM is much more variable in sequence than actin or MamK, likely faster evolving, and probably older (MreB evolves more slowly; Hsp70 slower still). *Thermoplasma* actin is so similar structurally to ParM of endobacterial plasmid pSK41 from *Staphylococcus* that Wagstaff and Löwe (2018) speculated it entered *Thermoplasma* by LGT. However that lacks convincing phylogenetic support: *Thermoplasma* actin is clearly closer to eukaryote actin than to proteobacterial ParM on one tree (Hara et al. 2007); though on another tree it and other euryarchaeote actins group with ParM rather than crenactins (Yutin et al. 2009), the basal branch of the ParM *Thermoplasma* clade is so weakly supported than we cannot infer LGT—the euryarchaeote *Archaeoglobus* lineage is comparably deep so ancestral euryarchaeotes may have had a 2-protofilament actin. The marked difference in proteobacterial and endobacterial ParM 3D structure is consistent with ancient divergence of these phyla on RP trees and largely vertical inheritance across prokaryotes. ParM perhaps evolved by MreB duplication before LUCA.

Histone origins radically affected DNA replication but did not fundamentally alter the standard chromosomal DNA segregation machinery mediated by transiently DNA-binding ParA ATPase mentioned above and the ‘centromeric’ DNA-binding protein ParB (Hu et al. 2015), both of which were lost when eukaryotes evolved mitosis instead. Prokaryotic chromosomal segregation and that of a few plasmids is mediated not by protein filaments as in other plasmids or by mts as in eukaryotes, but by a simpler Brownian ratchet or proteophoresis. In eubacteria, ParB dimers load onto DNA and become trapped at centromeric DNA ParS sequences (Debaugny et al. 2018). Then this centromeric complex is moved proteophoretically by diffusion reaction with a polar gradient of soluble ParA (Hu et al. 2015; Walter et al. 2017). Archaebacterial segregation is well studied only in *Sulfolobus*, which has two systems. The ancestral chromosomal one also uses just two proteins: ParA-homologue SegA and SegB which has no sequence homology to ParB and is distinctly smaller but like ParB is in the same operon as ParA (Schumacher et al. 2015). As SegB homologues occur throughout filarchaeotes and euryarcheotes, it was ancestral for archaebacteria. We suggest that evolving histones affected binding of ParB to ParS DNA so seriously that it was either radically truncated or replaced by a smaller ParS-binding protein that like ParB also functions as a dimer. *Sulfolobus* plasmid ParB is longer than in eubacteria, having an extra domain with some affinity to centromeric histone CenPA (Barillà 2016; Schumacher et al. 2015); its N-terminal DNA remains homologous to ParB, its C-terminal CenPA-like domain helps bind AspA, another dimeric DNA-binding protein with structural similarity to PadR transcriptional regulators, from one of which it likely evolved by radical sequence change. Thus, *Sulfolobus* exhibits alternative ways of modifying ParB but both are clearly modified from eubacterial ones and fundamentally conserve prokaryotic segregation principles, despite the plasmid system having become more complex by adding a third protein and the CenPA-like domain. We suggest that the CenPA domain evolved in the ancestral neomuran simultaneously with histones and was later recruited for kinetochores during eukaryogenesis (see below) and that the first steps in mitosis originated before the ancestral eubacterial segregation system was lost.

Eukaryotes, eubacteria, and most archaebacteria have ‘structural maintenance of chromosome’ (SMC) proteins, which in prokaryotes are needed primarily for DNA segregation and often called condensins as they help nucleoid condensation. Typical SMCs are long molecules of 1,100–1,200 amino acids with a long coiled-coil region connecting two terminal globular ATPase domains that bind to the ends of shorter kleisin proteins to make a composite loop structure that can encircle coiled DNA strands. Near replication initiation in prokaryotes ParB loads SMC/condensins onto the DNA (Barillà 2016; Kamada and Barillà 2018). Prokaryotes normally have only one SMC, but eukaryotes have six different SMC paralogues of different function that must have arisen from the single prokaryote SMC by gene duplications during eukaryogenesis. Eukaryotes only evolved cohesin by further SMC duplication, whose loop is loaded onto DNA at replication initiation and which functions to hold sister chromatids together until it is digested to initiate mitotic anaphase (Nasmyth and Haering 2009). The relatively small change in archaebacterial SMC compared with replication enzymes probably comes about because replication requires strand separation that is impeded by histone, but the chromosome interactive phase of loading of SMC does not involve DNA strand separation so would be less radically changed by DNA winding around core histones. In marked contrast to RPs, whose trees exhibit a very long neomuran stem that deeply separates all eubacteria from all archaebacteria, SMC trees show archaebacteria and eubacteria as intermixed (Soppa 2001), which stems from the relatively small change in archaebacterial compared with eubacterial SMC so they cannot be cleanly separated on sequence trees by a single long stem. In our view, this intermixing results from poor resolution by such single-gene trees coupled with numerous LBA artefacts, but has been misinterpreted as evidence for SMC LGT. As this purported LGT led Wolfe and Fournier (2018) to claim that archaebacteria are far older than all other evidence discussed above indicates, the next section has to refute their arguments in detail before we can discuss other aspects of neomuran evolution.

## Chromosomal SMC protein evolution and molecular ‘clocks’

Cavalier-Smith (2002a) pointed out that the extreme paucity of fossil evidence for archaebacterial dates might in principle be circumvented by using LGT to obtain relative dates between them and eubacteria or eukaryotes. But achieving this depends on firm evidence for LGT, good trees, and reliable fossil calibrations, a very rare combination. Soppa (2001) and Cobbe and Heck (2004) claimed that SMC proteins had undergone LGT from euryarchaeotes to Aquificales and Cyanobacteria and Wolfe and Fournier (2018) attempted to use relaxed molecular ‘clock’ (RMC) programmes and the assumption of SMC LGT to date euryarchaeote methanogenesis. Assuming a previously questioned identification of 2 Ga fossils as nostocalean ‘akinetes’, Wolfe and Fournier inferred a date of 4.53 ± 0.24 Ga for euryarchaeotes long before earth was inhabitable (and dating crown cyanobacteria at 2.93 before the GOE (~ 2.4 Ga)), both absurd. Using a lower 1.2 Ga age for akinetes instead as sole cyanobacterial calibration, they inferred 4.17 ± 228 for euryarchaeotes, older than any direct evidence for life and likely before the earth was habitable and 2.32 Ga for crown bacteria—older than another RMC inference of 2.0 calibrated by 8 fossil dates (including the too early 1.17–1.22 claim for red algae based on *Bangiomorpha* considered by us likely misidentifed cyanobacteria: Cavalier-Smith 2006a). These euryarchaeal dates are so much earlier than more direct evidence noted above that we must critically evaluate these inferences, which have several flaws and are unjustified. One problem is that none of these studies included any filarchaeote SMCs (presumably because of oft repeated incorrect statements that crenarchaeotes have no SMCs (e.g. Kamada and Barillà 2018) and numerous eubacterial phyla were also omitted by Soppa (2001) and Cobbe and Heck (2004) and virtually all except euryarchaeotes and the claimed LGT groups were omitted by Wolfe and Fournier, who uncritically assumed that the earlier claims for LGT site-homogeneous analyses were correct; none explicitly rooted the SMC tree.

**Fig. 12:**
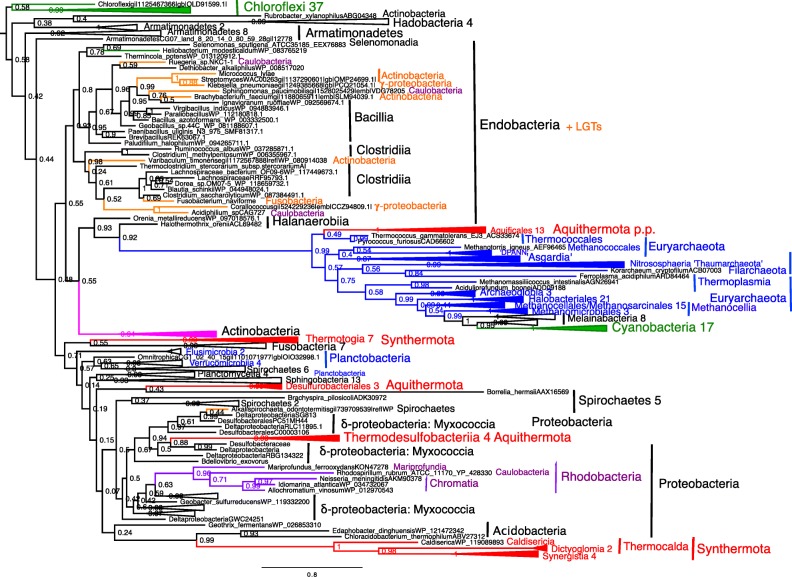
SMC site-heterogeneous PhyloBayes CAT-GTR phylogeny for 305 prokaryotes using 448 amino acid positions. Two chains were run and summed; after convergence (maxdiff. 0.209461) 40% of trees were removed as burnin. To fit on the page many clades were collapsed (numbers of species in each noted on the right); all names are on the corresponding uncollapsed tree (supplementary Fig. S17). Bipartition support values are posterior probabilities

Using name searches and BLAST (with *Synechocystis* gb BAA17371 as query) against GenBank, we identified SMC homologues throughout Filarchaeota and in all eubacterial phyla we recognise. Figure 12 is the first prokaryote-wide site-heterogeneous SMC phylogeny using the most conserved 448 amino acids from the two globular ATPase ends of the molecule. Unlike Soppa (2001) and Cobbe and Heck (2004), Fig. 12 omits eukaryotic paralogues as they are more divergent and their longer branches might have caused LBA artefacts, but a few are included in Fig. S16 together with some longer-branch prokaryotes omitted for the same reason. In well-studied examples, this binding of both ends to the short kleisins is facilitated by a central hinge domain that divides the long central region into two separate subequal coiled-coil domains. The hinge domain can be aligned across several phyla and was included together with the terminal domains in previous trees (Soppa’s distance trees included 527 amino acids and Wolfe and Fournier’s ML 729) but it is too divergent in some previously unstudied phyla or subgroups to be aligned with the majority, so we excluded it. Wolfe and Fournier’s study concatenated SMC with the much shorter ScpA kleisin (220–280 amino acids) and ScpB that binds it and helps load the ring onto chromosomes and are in the same operon, claiming that all three genes underwent LGT together.

The Fig. 12 SMC tree shows a majority of eubacterial phyla with short branches and similar topology to the RP tree (Fig. 5), with much lower basal support as expected for a single protein, but some phyla and some subphyletic lineages have much longer branches. These unsurprisingly show radically different topology, implying severe LBA artefacts. Most completely short-branch phyla form single clades, e.g. Chloroflexi, Sphingobacteria, Fusobacteria, Hadobacteria, but Armatimonadetes and Melainabacteria incorrectly appear paraphyletic. Phyla with mixed short-branch and long-branch taxa mostly wrongly appear polyphyletic (Spirochaetes, Actinobacteria, Synthermota, Aquithermota). The main short-branch spirochaete cluster correctly groups with Planctobacteria/Sphingobacteria and more distantly Proteobacteria as a gracilicute clade, but spirochaete *Borrelia* has a much longer branch that falsely groups with aquithermote long-branch subclade *Desulfurobacterium*/*Thermovibrio* (i.e. order Desulfurobacteriales). Synthermota broke up into the relatively short-branch Thermotogales that is weakly sister to Fusobacteria, whereas other Synthermota (*Dictyoglomus*, Caldisericia, Synergistia) are an extremely long maximally supported clade, wrongly with near maximal (0.99) support within Proteobacteria and Acidobacteria as sister to the long *Edaphobacterium*/*Chloracidobacterium* (acidobacterial) subbranch. Aquithermota splits into three long-branch clades in quite different parts of the tree; *Thermovibrio*/*Desulfurobacterium* with *Borrelia*; *Thermodesulfatator*/*Thermodesulfobacterium* another strongly supported clade that wrongly intrudes into Proteobacteria (within Myxococcia) and Aquificales that group extremely weakly with euryarchaeote Thermococcales. Wrong position of two thermophilic ‘clades’ within Proteobacteria is readily explicable as LBA artefacts, but could not be explained as LGT from proteobacteria (as both are basal and paraphyletic to their phyla by RP and LGT; the RP tree suggests that both may be older than proteobacteria, and LGT would not explain their long branches). There is equally no reason to invoke LGT to explain separation of *Borrelia* from short-branch spirochaetes or of long-branch *Rubrobacter* from short-branch Actinobacteria. By contrast, short-branch *Alkalispirochaeta* nesting shallowly within endobacteria is clearly either an LGT from endobacteria or misannotation.

Within Endobacteria also are several short-branch strains whose names belong to other phyla: Actinobacteria, α- and γ-Proteobacteria. These might all represent LGTs from Endobacteria to these phyla (orange branches: Fig. 12), but some or all might simply be misidentified/misannotated lineages. We excluded 3 other near identical short-branch strains that grouped within Endobacteria but were annotated from three different phyla, which seem likely misdentifications, and from Melainabacteria excluded several strains annotated as *Clostridium* or *Fusobacterium*, almost identical to genuine melainabacterial sequences that might represent very recent LGTs or (more likely) misannotations.

Most problematic is the huge long-branch composite clade comprising all archaebacteria plus Aquificales and the Melainabacteria/Cyanobacteria clade that we refer to as AAMC. This corresponds to the part of the tree from which Soppa and others inferred LGT from euryarchaeotes to Aquificales and Cyanobacteria. We argue that composite AAMC also is more readily explicable as a severe LBA artefact rather than LGT. It is striking that there is no distinct archaebacterial or filarchaeote or euryarchaeote clade. Instead, there are three filarchaeote and four euryarchaeote clades intermingled with each other and the two eubacterial clades. Thus, the SMC tree lacks the resolution to separate euryarchaeotes and crenarchaeotes and the basal branching order of archaebacteria and relative positions of Aquificales and archaebacteria is weakly supported. We conjecture that the SMC stems of the Melainabacteria/Cyanobacteria clade (Oxybacteria), Aquificales, and Archaebacteria independently accelerated so these three clades artefactually group together, similarly to the equally strongly supported false grouping of basal Synthermota with long-branch Acidobacteria on Fig. 12. On the much more reliable RP tree (Fig. 5), crown Synthermota occupy a much larger fraction of the phylogenetic depth of the eubacterial crown than do crown Acidobacteria and thus are arguably almost certainly an objectively older group, yet they nest within the younger group with 0.99 support. That cannot be explained by LGT from crown acidobacteria into the older stem Synthermota. Instead, it must represent a distortion by LBA between the long-branch Synthermota (which does not branch with its shorter branch true relatives, Thermotogia) that yields a blatantly false topology. One cannot correctly deduce the order of evolution of nested clades when LBA so strongly distorts the SMC tree that it gives a false topology with near maximal support—high statistical support is not an index of truth, but may simply mean that an untruth also can be is highly reproducible, as is mathematically proven for LBA. The misleading AAMC ‘clade’ is even longer than the clearly false Acidobacteria/Synthermota clade (it is the longest branch on the tree, ~ 5× the length of the shortest one) so we argue its topology is almost certainly also a consequence of grossly misleading LBA. The similarly temporally contradictory nesting of oxybacteria (which all other evidence we cite indicates are older than archaebacteria) within the *Archaeoglobus*/Methanomicrobiales part of euryarchaeotes is more likely to result from LBA than for LGT from archaebacteria to the ancestors of oxybacteria (which we dated above at ~ 2.3 Ga), making the premise of the attempted dating of methanogenesis (Wolfe and Fournier 2018) fallacious. If contrary to our best judgement that LGT did occur, it would imply that archaebacteria are older than 2.3 Ga; as the *Archaeoglobus*/Methanomicrobiales clade is only about two thirds the depth of the archaebacterial clade the inferred date would be > 3.45 Gy ago and if oxybacterial SMCs are really sister to Methanomicrobiales alone as Fig. 12 implies even older (~ 3.8 Gy). Above, we made three other independent estimates of crown archaebacterial age by mapping the more reliable RP sequence trees logically onto the fossil record; this gave three concordant dates of < 1.18 Ga (from position of the mitochondrial ancestor), < 1.18 (from chloroplast to cenarchaean LGT), < 1.17 (using halophile lipid age), all about three times younger than that from assuming an SMC LGT. The most obvious explanation of that big discrepancy is that the SMC LGT to oxybacteria never occurred and is a mathematical artefact of the gross disparity in branch lengths on the SMC tree. Another fallacy biasing the Wolfe and Fournier (2018) conclusion is the assumption that isotopically light methane trapped in 3.5 Ga zircons was biogenic (Ueno et al. 2006); as this methane could have originated abiotically (Sherwood Lollar and McCollom 2006), it was unjustifiable to use it as a lower bound on euryarchaeote age.

The ML SMC tree (Fig. S16) was broadly similar, better in some respects (e.g. Actinobacteria were not placed within Endobacteria and apart from the likely LGTs were a clade including *Rubrobacter*, and Endobacteria were a clade apart from their frequent LGTs and AAMC did not intrude within them), worse in others (e.g. the most divergent chloroflexan sequence separated from the rest). AAMC was maximally supported but put with insignificant support as sister to Fusobacteria, not within Halobacteriales, the deepest branching endobacterial subclade as with CAT. Probably neither position of this probably false clade has any historical meaning. AAMC had 7 distinct methanogenic subclades and four distinct filarchaeote subclades interspersed within them. Grouping of Aquificales with Thermococcales was stronger and of Cyanobacteria/Melainabacteria with Methanomicrobiales markedly weaker with ML than CAT. The two long branches of basal Synthermota and Aquithermota were less decisively supported as within Proteobacteria, though false grouping of basal Synthermota with long-branch acidobacteria remained strong.

Figure S17 includes five eukaryote sequences and 12 highly divergent prokaryote sequences omitted so as not to confuse the previous trees. Eukaryote SMCs group in two separate positions with shorter branch Asgard sequences not with five much longer Asgard sequences that form a likely entirely artefactual ‘clade’ with six ultralong-branches: five Sulfolobia, the euryarchaeote *Methanopyrus*, and *Coprothermobacter* a synthermote eubacterium. This pseudoclade is far longer than any others in the tree emphasising how grossly accelerated some SMC evolution can be. It would be a mistake to interpret this branch, arguably completely phylogenetically and temporally misleading, as evidence for LGT from Sulfolbia or *Methanopyrus* to *Coprothermobacter* rather than as an example of extreme LBA.

If contrary to our interpretation Aquificales SMC is genuinely related to that of Thermococcales, it might have been related by vertical inheritance or by LGT from stem Aquificales to stem Thermococcales, rather than the reverse as Wolfe and Fournier (2018) assumed. If any of these were true, the fact that it branches so close to the apparent base of all archaebacteria, not shallowly nested within them, means that it would not be much younger than archaebacteria so its age might be used to date them. From Fig. 5, Aquificales is a much younger clade than basal Aquithermota. Equating crown eubacteria depth to 3.5 Gy allows us to estimate from Fig. 5 proportions the age of crown Aquithermota as ~ 2.2 Ga (using the mean of the tips of the four basal branches to represent the present) and stem Aquificales as 1.9 Gy, and crown Aquificales as 0.94 Ga (using their longer tips to represent the present). Their separation from Archaebacteria would be between 1.9 and 0.94 Gy ago, contradictorily much less than the estimates of Wolfe and Fournier (2018) based on cyanobacteria, but entirely consistent with the other three estimates. Thus four, independent tree/fossil age estimates clearly contradict the seriously flawed SMC one.

Mean RMC estimate of the age of the split between Melainabacteria and Cyanobacteria ranges from 2.2 to 2.6 Ga depending on calibration assumptions (Shih et al. 2016). Thus, even with the likely most reliable 2.2 Ga date (compatible with proportions of our RP tree: Fig. 5), any purported LGT to the Cyanobacteria/Melainabacteria stem would have to be before ~ 2.2, possibly significantly before. Yet on Fig. 12, this clade appears to be nested much more shallowly within archaebacteria than is the much younger Aquificales, which could make archaebacteria seem as old as the earth and the root of the Fig. 12 SMC tree twice the age of the earth. This strong contradiction reached by assuming LGT rather than LBA is a reductio ad absurdum of applying a single clock to the SMC tree whose branches have evolved at hugely different rates. Objectively, SMC is much worse than multi-RP trees for establishing either topology or the relative dates of prokaryote phyla for two reasons: (1) very long branch accelerations were much more frequent; (2) being a single gene, basal branching order of the more conserved and thus more reliable short branches is much more weakly supported, allowing topology to be more profoundly distorted by LBA and false relationships involving long branches to receive higher ‘support’ than true ones based on short branches that are remarkably concordant with the RP trees.

If neomura diverged at the base of the PVC Planctobacteria as Fig. 5 suggests, they must be younger than the PVC clade, which Fig. 5 proportions date at ~ 2.5 Ga—a fifth age constraint contradicting the imagined SMC LGT. It would be more reasonable to estimate the age of Verrucomicrobia the only planctobacteria known to have mts, as the position of eukaryotes on Fig. 6 at the base of Euplancta is likely at least somewhat too low because their immense branch length will tend falsely to pull them towards the tree base. Moreover, if eukaryote mts are derived from planctobacterial ones, they probably really diverged within Verrucomicrobia, for whose crown we estimate an age of ~ 1.8 Gy from Fig. 5 proportions. That probably overestimates the likely age of neomura as mts are unlikely to have evolved as early as the ancestral verrucomicrobium as they may be taxonomically restricted to *Prothecobacter*. Thus RP trees, much less subject to erratic LBA than SMC, suggest (if correctly rooted in Fig. 5) that neomura are probably substantially younger than the Melainabacteria/Cyanobacteria clade, and archaebacteria younger still by an unknown amount, so the LGT postulated by Wolfe and Fournier (2018) would have been temporally impossible. The Wolfe and Fournier (2018) analysis ignored the major part of the SMC eubacterial tree other than the likely false AAMC ‘clade’; applying their ‘clock’ reasoning to the excluded part of the tree would make it about twice the age of the earth. Evolutionary rates varied so greatly and erratically across Fig. 17 that applying RMC analysis to the SMC tree was unjustified.

Earlier attempts to apply RMC to archaebacteria are equally unreliable and biased to excessive antiquity, e.g. Blank (2009) recognised that the Ueno et al. (2006) assumption of light zircon-trapped methane as of biotic origin is unjustified so did not use it, but believed the old 2.7 Ga biomarker dates and used them as the minimum age for euryarchaeotes for the sole calibration even though Cavalier-Smith had argued they were discordant with other evidence and must be misinterpreted. It is now generally accepted that they are the result of modern contamination and totally misleading—the several-fold younger dates cited above are the oldest of those since the contamination problem was recognised. Rothman et al. (2014) accepted that and instead assumed that archaebacteria were 3.5–3.9 Ga, citing two old papers to support that excessively early date (Feng et al. 1997; Sheridan et al. 2003) which both assumed a fixed ‘clock’ and that the universal root was between archaebacteria and eubacteria and that they are of equal age despite strong arguments to the contrary (Cavalier-Smith 1987c, 1989a, 2002a). However, Sheridan et al. (2003) did use a 2.7 Ga likely contaminated biomarker date for calibration to get their date of 3.46 for crown archaebacteria from the crudest possible 16S rDNA distance tree. Feng et al. estimated archaebacterial/eubacterial ‘divergence’ (a misleading term if eubacteria are ancestral) at 3.8 Gy from 25 enzymes by back extrapolation many-fold simply from 100–450 Ma vertebrate fossil dates and to infer divergence of archaebacteria and eukaryotes from 8 enzymes at 2.4 Ga and of eubacteria and archaebacteria, both unreasonably assuming a fixed clock; these contradictory estimates of archaebacterial age imply evolutionary acceleration in the neomuran stem inflating the former. Multigene trees now tell us that euryarchaeotes are not significantly older than eukaryotes, so if Rothman et al. had wanted to believe Feng’s grossly oversimplified calculations, they should have used 2.4 (or 2.1 the contradictory date given in the text) not 3.9. Thus, Rothman et al. (2014) do not use any direct evidence at all for archaebacterial age and effectively admit that their assumption is based on ‘scenarios’ for early evolution, not evidence (of the three they cite, two are highly speculative and mutually contradictory, one accepting Woese’s disproved progenote idea (Martin and Russell 2003) the other correctly rejecting it (Gogarten-Boekels et al. 1995), but most researchers accept neither scenario). Rothman et al. (2014) disingenuously claimed to be ‘unaware of any seriously considered scenarios for a much later origin of the major domains’ despite a radically later origin of neomura having been seriously advocated in great detail for over 30 years (Cavalier-Smith 1987c, 1989a, 2002a, 2006a, c, 2014), and others also arguing that archaebacteria evolved from eubacteria and so are younger, e.g. Reynaud and Devos (2011), Valas and Bourne (2011), and no serious students of cell evolution accepting that eukaryotes evolved in the early Archaean! Neglect of strong evidence for neomura being derived stems from pervasive uncritical repetitions of Woese’s erroneous ideas, past palaeontological misinterpretations, and substitution of common preconceptions for actual evidence for dates. We are unaware of any convincing evidence that archaebacteria are anywhere near eubacteria in antiquity—most likely they are 2–4 times younger. A younger more realistic age for archaebacteria than was arbitrarily chosen by Rothman et al. (2014) would make the LGT acquisition of acetoclastic methanogenesis by *Methanosarcina* much later than 250 Ma and thus irrelevant to the end-Permian extinction, contrary to their speculation.

The more careful RMC estimate of 2.42–2.88 for crown euryarchaeotes based on 29 proteins of which 12 were RPs (Betts et al. 2018) radically contradicts the billion year older assumptions/inferences of Blank (2009) Wolfe and Fournier (2018) and Rothman et al. (2014). But Betts et al. (2018) also is deeply flawed by applying the same clock to the crown groups and the grossly stretched stems and by rooting the tree in the middle of the neomuran stem which would tend to inflate neomuran dates and contract eubacterial ones. Fossil calibration was also defective as at least three of the 11 minimum dates used are highly suspect and probably grossly inflated. Betts et al. (2018) wrongly accepted *Bangiomorpha* as a red alga and used their so-called minimum date of 1033 Gy for crown eukaryotes, crown cyanobacteria, and crown α-proteobacteria. That and using 1619 Ma acritarchs from the Changcheng group (Peng et al. 2009) as a minimum age for stem eukaryotes would inflate all early eukaryote dates and likely cause knock-on inflation for archaebacterial ones. None of these acritarchs have any morphological features that could not have been produced by prokaryote machinery so may simply be eubacterial; some might be large bacterial cells, others curled sheets of bacterial mat fragments. Using 3.225 Ga Barberton banded iron formations (BIF) as a minimum date for stem cyanobacteria is highly likely to be erroneous: it is not credible that they are that old given their shallow branching on RP trees and the 0.8 Gy later date of the GOE, and will have distorted many inferences; causes of these Archaean BIFs remain controversial; many do not accept them as evidence for cyanobacterial photosynthesis—there could have been other unknown sources of alternating oxidation and reduction. Accepting their date and our rooting of the tree would put its root before the earth was formed! Their assumptions put LUCA unacceptably early at 4.519–4.477 Ga. None of their other calibrations except 3.4 Ga for LUCA (close to the 3.5 Ga we assume but topologically seriously misplaced on the neomuran stem) are older than 550 My for animals. Their four PhyloBayes trees were taxonomically much less rich than ours, did not converge, and were topologically inferior for eukaryotes in putting *Giardia* as the most divergent eukaryote and showing both Cnidaria and Metamonada as paraphyletic (whether GTR+G or CAT-GTR+G). All of them wrongly showed Harosa as strongly polyphyletic so it was disingenuous to claim their trees ‘reflect current consensus relatively well’. For eukaryotes, their topology is exceptionally bad. All our trees had a strongly supported Harosa clade (like virtually all well sampled eukaryote multigene trees) and none put metamonads deeply. For Eubacteria, these trees were worse than ours or those of Boussau et al. (2008b) in failing to show Gracilicutes or Planctochlora as clades and in wrongly putting with maximal support *Gloeobacter* within the thylakoidal cyanobacteria. Their protein choice, alignments, or analyses cannot have been very good. Simple manual dating inferences made here by treating crown domains and the two inflated stems separately are superior to RMC computer inferences with so many grossly mistaken assumptions.

## Phagotrophy, eukaryogenesis, and mitochondrial fallacies

A planctobacterial origin of eukaryotes does not imply that planctobacteria ingested the α-proteobacterial ancestor of mitochondria, as McInerney et al. (2011) wrongly asserted. That is because planctobacteria must have been radically altered to become stem neomura by the loss of murein, and origin of N-linked glycoproteins, core histones, and ESCRTIII before the later origins of archaebacteria and mitochondria. Thus, the immediate ancestor of eukaryotes was not a eubacterium, but a substantially different *stem neomuran*—logically *neither planctobacterium nor archaebacterium*, but a transient evolutionary intermediate between both. Moreover, according to the phagotrophic interpretation of eukaryote origins the endomembrane system and phagocytosis evolved before mitochondria and provided the easy mechanism for engulfing their ancestor. Therefore, it was doubly wrong for McInerney et al. to imply our interpretation requires *planctobacterial* ingestion of the mitochondrial ancestor. Martin has repeatedly obfuscated the debate about eukaryogenesis by misrepresenting supporters of a phagotrophic origin (see Cavalier-Smith (2014) for some examples). The mechanistically implausible idea that mitochondria evolved first and stimulated later origin of the endoplasmic reticulum (ER), as Margulis originally claimed (refuted in detail by Cavalier-Smith 1983b) but later rejected, was revived in modified form by Martin and colleagues and still promoted by McInerney et al. (2011). It provides no mechanism for uptake of mitochondria, since as Stanier and Van Niel (1962) first pointed out no prokaryotes have ever been shown to be capable of taking up another cell or supporting intracellular symbiosis. Martin and colleagues (e.g. Martin and Russell 2003) repeatedly cited the nested symbiosis of a γ-proteobacterium (*Moranella*) within the cytoplasm of a β-proteobacterial ‘endosymbiont’ within the cytoplasm of the citrus mealy bug *Planococcus citri* (von Dohlen et al. 2001) in a failed attempt to refute Stanier’s generalistion and make us believe, contrary to all evidence, that free-living prokaryotes are mechanistically able to take up other cells and sustain cellular endosymbiosis.

Cavalier-Smith (2002b) argued that the mealy bug example does not refute earlier arguments that free living prokaryotes are incapable of taking up other cells and supporting them as intracellular endosymbionts—no example exists in the history of life of a free-living heterotrophic prokaryote ever having ingested another bacterium; the undisputed fact that all eukaryotes with phagocytosis can ingest cells—and do so billions of times a day—makes phagocytosis by far the most likely and the only mechanistically plausible mode of origin of mitochondria (López-Madrigal et al. 2011). Cavalier-Smith (2002b) argued that the inflated cytosol of the β-proteobacterial host was physiologically more analogous to a eukaryotic organelle membrane and must have modified its cell envelope that in free-living eubacteria would prevent foreign cell uptake. This later proved correct. The 139 kb genome size of the β-proteobacterial ‘symbiont’ *Candidatus* Tremblaya princeps (only 110 functional protein-coding genes, 43 RPs: López-Madrigal et al. 2011) is only slightly larger than the largest jakobid mitochondrial genome (Burger et al. 2013), far smaller than any free living bacterium, and several times smaller even than parasites like intracellular mycoplasmas that can be cultivated independently of their eukaryotic hosts that typically have 500 kb or more. Tremblaya absolutely lacks genes for cell envelope biogenesis, energy transport, synthesis of nucleotides, cofactors, aminoacyl-tRNA synthetases, energy production, and transport and cannot be considered a living organism or bacterium. It is a vertically and maternally transmitted *insect organelle* that evolved by enslaving a β-proteobacterium and is retained for the sole function of providing genes for most of the intraorganellar synthesis of essential amino acids for the mealy bug. This amorphous non-rod-shaped organelle, here called the ‘aminosome’, is less like a bacterium even than mitochondria in morphology and division mode; unlike aminosomes, more primitive mitochondria retain FtsZ. Aminosomes must never be given formal Linnean names in the way once wrongly done for the chloroplast of *Cyanophora*, which is more bacteria-like in having retained the murein wall. Like mitochondria, aminosomes lost murein but kept two membranes. Aminosome ability to harbour endosymbionts is not an ancestral bacterial property, but a *novelty* that arose *after* it became an organelle—this ability is no more surprising than the ability of mitochondria to do so. It therefore does not contradict Stanier’s generalisation that bacteria never harbour cellular endosymbionts. Some mealybugs (e.g. *Phenacoccus azaleae* (Koga et al. 2013)) have aminosomes without endosymbiotic *Moranella* γ-proteobacteria. Moreover, those harbouring a *Moranella* can replace it by a different one (Husnik and McCutcheon 2016). *Moranella* is thus an independent organism from the host mealybug, and aminosomes are no longer bacterial symbionts but obligately vertically inherited *eukaryotic organelles*. All are more closely related to each other than to genuine β-proteobacteria and their genomes evolve much faster. Booth and Doolittle (2015) similarly misinterpreted ‘Tremblaya’ as a bacterium rather than an organelle; partly for this reason, but more through underplaying uniqueness of the endomembrane system, nucleus, mitosis, sygnamy, meiosis, and cilia, they did not come to grips with the most difficult eukaryogenic innovations, which are not the origin of mitochondria, but of these and related non-symbiogenetic characters. Margulis’s extreme overemphasis on symbiogenesis lives on.

Lane and Martin (2010) also cite Wujek (1979) as having seen intracellular bacteria within a cyanobacterium. Only a drowning man clutching at straws would cite that as refuting Stanier’s generalisation that prokaryotes never harbour cellular endosymbionts. Only a small fraction of the cells were infected by bacteria and fixation was not good enough to see membranes clearly. Therefore, the infecting bacteria might actually all be in the periplasm, not the cytoplasm of the cyanobacterium, like *Bdellovibrio* a proteobacterial parasite that can cross the OM of negibacteria and live in the periplasm. These bacteria are clearly Gram-negative and their curved shape is consistent with their being *Bdellovibrio*. We suggest they probably are periplasmic *Bdellovibrio*, and irrelevant to the origin of mitochondria, which are truly within the plasma membrane and had to cross it (whose only well-established mechanism is phagocytosis). Until she gave up her idea of mitochondria first to which Martin still clings, Margulis often cited *Bdellovibrio* as possible ancestors of mitochondria in the incorrect belief that they were truly intracellular. Martin has probably made the same mistake. One needs simply to refine Stanier’s dictum by saying that prokaryotes never harbour cellular symbionts within their cytoplasmic membrane. One other example of periplasmic cells exists: *Nanoarchaeum* in *Ignicoccus*. Neither *Bdellovibrio* nor *Nanoarchaeum* is a good model for the origin of mitochondria, which almost certainly followed the origin of phagocytosis, which must have been before LECA.

However, we agree with McInerney et al. (2011) that the idea that eukaryotes arose by a planctobacterium engulfing a thaumarchaeote prior to the proteobacterial/mitochondrial enslavement (Forterre 2011) is unrealistically complicated. It is entirely unnecessary to postulate two cell lineage mergers to make eukaryotes to explain the dual affinity of neomura with both archaebacteria and planctobacteria—provided one does not make the Woesean mistake still clung to by McInery et al. and Forterre of placing LUCA in the neomuran stem, which is incompatible with and decisively refuted by the integrated palaeontological and sequence tree evidence discussed above for crown eukaryotes being about four times younger than eubacteria. As explained when refuting an earlier ‘fusion theory’ involving δ-proteobacteria and archaebacteria (Cavalier-Smith 2010d), all speculations of fusion of prokaryotic lineages to generate eukaryotes are cell biologically and mechanistically implausible as no mechanism is known or proposed for such fusion; these mechanistically defective ideas include the hydrogen hypothesis (Martin and Müller 1998), which misrepresented the classical interpretation of a phagotrophic origin of mitochondria by claiming that the energy benefits of compartmented aerobic respiration over anaerobic metabolism were illusory. But as soon as phagocytosis evolved, it enabled foreign cells to be taken up easily and harboured as symbionts. Contrary to what McInerney et al. (2011) imply, a phagotrophic origin of eukaryotes and a post-phagotrophy origin of mitochondria does not require the existence today of primitively amitochondrial eukaryotes, nor even that mitochondria evolved later than eukaryotes. Both could have evolved at essentially the same time, as we have argued ever since accepting that all extant anaerobic eukaryotes had aerobic eukaryote ancestors (Cavalier-Smith 2002c, 2006a, b, c, 2007a). Origin of endocytosis and endomembrane system were prerequisites for both the origin of the nucleus and of mitochondria. It is therefore not surprising if both happened in a facultatively aerobic prekaryote, in which case there were never any fully developed primitively amitochondrial eukaryotes with mitosis, nuclei, and cilia. We have consistently argued that such a prekaryote was most likely a facultative aerobe/anaerobe and that the same was true of the proteobacterium that it enslaved; that is physiologically more plausible than assuming an obligately anaerobic host for an aerobic symbiont as did the hydrogen hypothesis (Martin and Müller 1998). Even Forterre’s cell fusion hypothesis (he himself thought it wrong) does not ‘predict the existence of primitively amitochochondrial eukaryotes’ as McInerney et al. (2011) misleadingly claimed.

A major merit of the phagotrophy explanation of eukaryogenesis is that it not only simply and naturally explains how mitochondrial ancestors entered the prekaryotic cell, but also explains (as noted above) *why* eukaryotes evolved the endomembrane system (see Cavalier-Smith (2009) for a very detailed explanation of its phagotrophic origin), mitosis and nucleus (both previously explained in great detail: Cavalier-Smith 2010d), and provided the *mechanism* for endomembrane and DNA internalisation, which no fusion theory has ever done. Cavalier-Smith (2009) emphasised that digestion of prey bound by glycoproteins to the cell surface likely preceded full internalisation and intracellular digestion and argued that these complex processes could have been improved in gradual steps each able to be selected for improved efficiency. Very likely, this included an intermediate stage in which branched actin made a cup that only partially enclosed the prey, full internalisation probably requiring actomyosin contraction and novel membrane fusion and scission. *Braarudosphaera bigelowi*, a tiny 1.3-μm haptophyte keeps its only marginally smaller cyanobacterial prey half in and half out of its cell for a long time by a similar form of phagocytosis (‘pomacytosis’: Kamennaya et al. 2018), showing that eukaryotes can probably gain nutrients from prey only partially ingested. That shows that intermediate stages in prey internalisation are of selective advantage and that bacteria only slightly larger than average could have readily evolved phagocytosis once murein and the OM were lost. Even today, most oceanic phagocytosis is by 1–3 μm algae that eat bacteria. A planctomycete can engulf prey cells (Shiratori et al. 2019)

Lane and Martin (2010) made the remarkable totally misleading claim that mitochondria are essentially the only distinctive feature of eukaryotes. In falsely claiming that “virtually every ‘eukaryotic’ trait is also found in prokaryotes”, they listed such traits misleadingly. For example, they listed recombination, linear chromosomes, dynamic cytoskeleton, predation, parasitism, introns, and intracellular signalling, but no serious student of eukaryogenesis claims that any of these are unique to eukaryotes or was the crucial cause of their origin. With respect to phagocytosis that is truly unique to eukaryotes and universally accepted as an ancestral character for them, they simply asked if it was decisive, ‘why didn’t eukaryotes evolve repeatedly for the same reasons?’. The simple answer is that the transition was so complex, involving so many steps, that once eukaryotes evolved the likelihood that it was repeated was zero because inital ‘attempts’ to do so by other prokaryotes would have failed in competition with preexisting eukaryotes. That is the explanation Darwin gave to why didn’t life evolve more than once and evolutionary biologists give to the similar question why birds or vertebrates or echinoderms or vascular plants, or arthropods or any other group with uniquely evolved body plans arose only once. The authors either did not know that commonplace answer to their rhetorical question or deliberately concealed it in an attempt to unfairly dismiss phagotrophy as the real key to eukaryogenesis.

As uniquely eukaryotic properties, Lane and Martin (2010) did not mention endomembrane system, mitosis, nuclear pore complex, meiosis, cilia, DNA replication preinitiation machinery that allows thousands of replicon origins, and cyclin/protein kinase cell cycle controls, all far harder to evolve than mitochondria, and more relevant to the origin of eukaryotic complexity, and none explained at all by the origin of mitochondria. Cavalier-Smith (2009) listed 60 unique eukaryotic properties, almost all still valid (see below).

Their assertions that ‘mitochondrial genes enabled a roughly 200,000 rise in eukaryotic genome size’ and that compartmentalisation of respiration was not a factor in the success of mitochondria are both mechanistically implausible and logically indefensible. What might have done that or how? One flaw in Lane and Martin (2010) is their assumption that power per Mb DNA is equivalent to power per gene; this overlooks the fact that in eukaryotes unlike prokaryotes DNA amounts do not scale with gene numbers because so much nuclear DNA and a hugely variable proportion of the genome is non-coding. Therefore, their calculations grossly inflated the supposed energetic cost per gene in eukaryotes. But even if they had done their calculations correctly, they would have been evolutionarily meaningless.

Lane and Martin (2010) asserted that ‘The massive difference in mean genome size between prokaryotes and eukaryotes is most revealingly quantified in terms of energy available per gene. By ‘energy per gene’, we mean the cost of expressing the gene.’ Why they should believe that biologically meaningless abstract ratio to be evolutionarily revealing mystifies us and others (Lynch and Marinov 2016) as selection works through inherited differences in cell and organismal reproductive rates. The classical replicon length argument (see next section) much more powerfully limits bacterial genome size than their imaginary energetic ‘constraint’. As an evolutionarily better informed and more thorough analysis shows (Lynch and Marinov 2015), gene expression costs per gene scale sublinearly with genome size in a continuous fashion between bacteria and eukaryotes; therefore, adding extra genes would have a less than proportional effect on energetic costs. In marked contrast, increases in genome size for prokaryotes will proportionately increase replication time and thus more severely limit reproduction rates. In replying to Lynch and Marinov’s (2015) decisive refutation of their argument, Lane and Martin (2016) falsely claimed that their paper was ‘not about the bioenergetic costs of a gene at all’ and that Lynch’s conclusions refuting their muddled energetic arguments are untrue. They claimed their paper was about energy supply, not demand, criticising Lynch and Marinov for not discussing supply. But nor did Lane and Martin—the word ‘supply’ occurs once only in a figure legend irrelevant to their argument.

By contrast, a central part of the phagotrophy argument is that phagotrophy immediately radically increased the supply of energy and nutrients to the first eukaryotic cells. The cost of the phagotrophic machinery would be manyfold smaller than the gain. Phagotrophy immediately shifted the balance of selective advantages over cell size. Lane and Martin (2010) rightly emphasised the century-known principle that in osmotrophs like bacteria size scaling of diffusion and transmembrane transport gives a strong advantage for small cell size and high surface to volume ratios. That is not true for phagotrophs where greater cell size than prey is often advantageous. It is perverse not to recognise that phagotrophy was the biggest discontinuity in nutritional mode in the history of life that completely changed the scaling laws for both nutrition and genome size evolution with cell volume. In addition, compartmentation by the endomembrane system and mitochondrial enslavement had a major effect in altering scaling.

A branching endoskeleton may have slightly preceded and facilitated and necessarily coevolved with phagocytosis as explained below. Initially, the endomembrane system, endoskeleton, and associated motors would have been more important and cytologically transformative than mitochondria. If to begin with the host was able to respire, enslaving mitochondria would have done much less to increase energy supply proportionally than would the origin of phagocytosis that dramatically increased energy and nutrient supply by providing large chunks of highly digestible food. The scale of the energetic benefit of phagotrophy was vastly greater than anything that could have been supplied by increasing osmotrophic efficiency. Predators with a glut of prey (bears during the salmon run) have no need to use all their food efficiently—it is more advantageous to choose the richest parts and throw away the rest. As lipids are so energy rich, improving β-oxidation of fatty acids by compartmentalising their enzymes in endomembrane-derived peroxisomes may have been more important than compartmentalising respiration in enslaved mitochondria. If they believed energy was the key it was illogical for Lane and Martin to oppose the roles of phagotrophy and compartmentalisation in eukaryogenesis.

Phagotrophy theory argues that the entirely novel selective advantage of being the first organisms ever able to carry out internal digestion (which no predatory bacteria can do: but see final section) so dramatically increased energy supply as to be a far more powerful and innovative selective force than syntrophy that had been around for billenia. But the reason why it transformed bacteria into eukaryotes was not merely huge selective advantage and novelty (so no prokaryote competitors could compete in that adaptive zone), but that it provided novel mechanisms of membrane breakage and fusion as the actual physical mechanisms of formation and differentiation of the endomembrane system. Mere syntrophy does not do that so is conceptually a grossly inferior explanation. Moreover, as Stanier (1970) adumbrated and one of us explained in detail (Cavalier-Smith 1975, 1978, 1981, 1982, 1987c, 1991a, b, 2006a, b, 2009, 2010d, 2014), the radically new phagotrophic adaptive zone was a powerful evolutionary force for evolution of morphological complexity, especially of mt skeleton and cilia. As Cavalier-Smith repeatedly stressed, intracellular coevolutionary interactions between endomembranes and cytoskeleton and between both and genome and chromosomal organisation reshaped fundamental cell biology, genetics, and cell cycle to make eukaryotes (Cavalier-Smith 1975, 1981, 1987c, 1993, 2002c, 2006b, 2010d, 2014). These are not independent problems. Syntrophy and metabolic/bioenergetic aspects of evolution stressed by Martin and colleagues though important have nothing useful to say about such radical innovations far beyond their narrow metabolic/energetic paradigm. Evolutionary cell biology is immensely broader than biochemistry. Evolution embraces changes in cell lineages of all things affecting their relative reproductive success.

## Intracellular coevolution and eukaryote genome size

The claim that origin of mitochondria was the key stimulus for eukaryotic genomes becoming more complex (Lane and Martin 2010) by providing more energy was fallacious and much discussion therein tendentious. They arbitrarily selected mitochondria as the key defining character of eukaryotes and entirely ignored the earlier logical explanation given for the much larger genome sizes of eukaryotes than of bacteria, which was threefold. First, and most fundamentally was the novel cell cycle controls of eukaryotes via initiation of DNA replication at the beginning of S-phase that allows an indefinite number of replicons to initiate simultaneously—impossible in prokaryotes (Cavalier-Smith 1981, 1985a, b, 1987a, 2010d). That essential permissive innovation directly removed the previous contraint on the time taken for DNA replication that in prokaryotes is set by the total length of DNA in the single chromosome (Cavalier-Smith 1985b). With a single replicon origin, bacterial replication time increases linearly with genome size so the cell cycle takes proportionately longer. Thus, a thousandfold increase in genome size would convert a one hour cell cycle into a 6-week cell cycle, a huge selective disadvantage. Prokaryotes cannot increase genome size and gene numbers without slowing down cell reproduction rates greatly. That is not true of eukaryotes which can increase genome size without any replication time limits just by multiplying initiation points (Cavalier-Smith 1985b). Ability of a few archaebacteria to have two or three origins makes no essential difference to this compelling argument. The second innovation was mitosis, which allows simultaneous rapid DNA segregation without limit to the number of chromosomes, which can exceed a thousand. This also made total DNA length not mechanically limiting during segregation, but that was a subsidiary and less crucial limit to genome size. Thirdly, origin of the nuclear envelope gave eukaryotic DNA a new skeletal function in directly influencing nuclear size and the cytonuclear ratio, which are crucial for balanced growth in eukaryotes (Cavalier-Smith 2005); that is why in eukaryotes (in marked contrast to prokaryotes), genome size has to increase in direct proportion with cell volume, thereby explaining why non-coding DNA increases more than proportionally with cell volume in eukaryotes and is kept to a minimum in prokaryotes (Cavalier-Smith 1985a). That provided a positive selective advantage for more DNA (not more genes) in larger cells.

## Planctobacterial origin of eukaryote microtubules

We have adopted the view of a planctobacterial origin of neomura (Reynaud and Devos 2011) partly because the formerly postulated posibacterial origin (Cavalier-Smith 1987c, 2014) now appears to be excluded by RP trees. More important are cumulative discoveries of properties of the microtubules (mts) of the verrucomicrobial *Prosthecobacter* that make it highly probable that planctobacterial mini mts (Pmts) were ancestral to eukaryote mts (Deng et al. 2017; Pilhofer et al. 2011), not derived from them by LGT as previously supposed (Pilhofer et al. 2007b; Schlieper et al. 2005).

Eukaryote mts are typically composed of 13 protofilaments, but in animals, rare examples with 11, 12, 14, or 15 occur naturally; in vitro tubulin self assembly of αβ-tubulin heterodimers produces a broad range of diameters with 9–16 protofilament mts, 14 being most abundant (Chaaban and Brouhard 2017). As 13 protofilament (pf) mts are universal in eukaryotes and have never been found in prokaryotes, they likely evolved in stem eukaryotes during eukaryogenesis. The 13-pf pattern is probably imposed by nucleation on the γ-tubulin ring complex composed of 13 γ-tubulins (Chaaban and Brouhard 2017), which has not been identified in prokaryotes. Pmts by contrast have only 4 pfs when self assembled in vitro from heterodimers (bacterial tubulin A/B) accompanied by GTP hydrolysis (Deng et al. 2017) and possibly 5 in vivo. Nonetheless, individual pf architecture is strikingly similar to that of mts, being a structurally polarised linear array of identically head-to-tail oriented heterodimers (Deng et al. 2017; Martin-Galiano et al. 2011). As one would expect of ancestral tubulins, they can self assemble without any γ-tubulin. Also as expected for ancestral tubulins, A/B heterodimers fold correctly without any requirement for eukaryotic chaperonin CCT or other proteins essential for eukaryotic heterodimer formation; the even more primitive prokaryotic GTPases, FtsZ, evolved pre-LUCA long before their tubulin homologues. Unlike αβ-tubulin and BtubA/B, FtsZ and likely derived TubZ, both form single or double pfs only of a single protein, never make mts and do not bind standard antitubulin drugs. FtsZ lacks the typical loops that make contacts between tubulin heterodimers or with CCT; absence of these loops is clearly the ancestral condition.

Folding of α- and β-tubulins individually and their subsequent heterodimerisation is complex, requiring first binding to the generalised chaperonin CCT, a neomuran innovation, helped by prefoldin, then to a succession of five tubulin-specific folding factors (TBCA-E), several essential for life (Szolajska and Chroboczek 2011) which apparently evolved during eukaryogenesis after divergence from archaebacteria. CCT is hetero-octomeric, its eight related proteins having arisen by repeated gene duplication in stem eukaryotes after they diverged from archaebcteria whose CCTs comprise only 1–3 proteins (Archibald et al. 2000). Thus, CCT was homomeric in the ancestor of archaebacteria and neomura and is unknown in eubacteria (Archibald et al. 2001). The very first ancestral mts cannot have had such complex requirement for 14 different proteins for their assembly or they would never have been able to evolve from a simpler ancestor like FtsZ/TubZ. As more has been learned about their folding complexity, the less reasonable has it been to suppose that mts with such requirements evolved suddenly just after murein loss led to the origin of phagotrophy as originally proposed (Cavalier-Smith 1975). Later it was argued that after murein loss mts first evolved in still rod-shaped premitotic prokaryotic cells as a longitudinal supporting skeleton that would constrain division (putatively then by a primitive actin ring) between daughter DNA attachment sites to the CM (Cavalier-Smith 1987c). This premitotic mechanism was assumed to have evolved before evolution of the nucleus and phagotrophy at the cell surface with mts having initially a structural and cleavage-furrow-constraining role prior to evolution of mt-associated motors later coopted for true mitosis. Discovery of Pmts supports that idea that mts evolved whilst cells were still prokaryotic with DNA attached to the CM, not ER, and makes it likely that mts evolved for a purely structural cell cortical role even before loss of murein and OM. Their much simpler folding and assembly requirements, needing only KCl not other proteins (Deng et al. 2017; Pilhofer et al. 2011) lessens the mechanistic abruptness of the origin of mitotic machinery if PMts were the previously missing link between FtsZ/TubZ single filaments and mts.

Previously, TubZ that partitions DNA in plasmids and phages (Oliva et al. 2012), especially in Endobacteria, and undergoes treadmilling, was proposed as a more likely tubulin ancestor than FtsZ (Cavalier-Smith 2010d). We now argue that BtubA/B are even more likely ancestors as they have already undergone gene duplication and divergence to make mini mts bound to the CM inner face (predominantly in the *Prosthecobacter* stalk region). Less innovation would be necessary for recruitment for eukaryotic functions, greatly simplifying the difficult origin of mitosis after murein loss and chromosome internalisation by phagotrophy made DNA segregation changes unavoidable. As noted above, archaebacteria kept eubacterial DNA segregation by ParA, so phagocytic chromosomal internalisation, not murein loss per se, was likely the key trigger for evolving mt-based mitosis. Pmts like mts exhibit treadmilling, dynamic instability, and polarised growth (faster at one end: Diaz-Celis et al. 2017) and are functionally true mts despite their notable ability to self assemble without other proteins helping.

The intermediate nature of BtubA/B is shown not only by their simpler folding/assembly mechanisms but in their 3D structure and separate functions of A and B being weakly differentiated compared with α- and β-tubulins (Martin-Galiano et al. 2011), e.g. both BtubA and B can hydrolyse GTP, and the 8-amino-acid insertion specific for α-tubulins (derived compared with FtsZ) is absent in BtubA and B—our own alignment including B-tubs suggests that insertion is really a seven amino acid insertion plus a single amino acid deletion in β-tubulin six amino acids downstream and that γ- and ε-tubulins like Btubs lack the 7-amino-acid insertion and the single amino acid deletion. Both Btubs are rather divergent from αβ-tubulins in their C-terminal region that binds eukaryote-only molecular motors and the regions that bind CCT. Unlike most eukaryote tubulins, which are not linked and separately transcribed, Btubs are adjacent within a typically bacterial operon, which includes a gene for a third cytoskeletal protein (‘kinesin-like’ or BtubC) (Pilhofer et al. 2007a, b). All three genes are cotranscribed and have their own typically eubacterial Shine-Dalgarno sequences. BtubC binds to pfs every 8 nm, predominantly via BtubB; it inhibits Pmt catastrophe and probably links Pmts to the CM (Deng et al. 2017). BtubC, like kinesin light chains, is a tetratricopeptide repeat (TPR) protein but its 3D structure is no closer to kinesin light chain TPRs than to proteobacterial TPR protein MamA that binds to membrane protein Mms6 of magnetosomes (Nguyen et al. 2016). TPR proteins are rare in prokaryotes but abundant in eukaryotes, e.g kinesin light chain and several proteins of the anaphase promoting complex (APC).

There is no reason to think that BtubC might be of eukaryotic origin; the fundamentally prokaryotic nature of the Btub operon makes it unlikely but not impossible that it came by LGT from eukaryotes. If Btubs did originate from eukaryotes they must come from one of the rare protists, e.g. *Trypanosoma* where α- and β-tubulin genes are clustered and cotranscribed (Imboden et al. 1987); most eukaryotes would not be plausible ancestors. The alternative idea of LGT from a stem eukaryote lineage after the divergence of α- and β-tubulins but before evolution of dependence on CCT/TBCA-E means that they would not have come from a eukaryote but from a transitory prekaryote. As that must have existed over 850 My ago and would have been just a single short-lived lineage, any chance of such a donor even making such a transfer would have been very low, quite apart from its likely inability to survive. It is much simpler to suppose that verrucobacterial Tubs were ancestral to α-tubulin and β-tubulin rather than derived from a transient prekaryote by LGT, for which there is no direct evidence.

BtubA/B are in all four *Prosthecobacter* species and on parsimony trees BtubA and B are distant sisters to α-tubulin and β-tubulin respectively, γ-tubulin and the centriole-specific tubulins δ, ε and ζ are more distant (Jenkins et al. 2002). By ML, BtubA remained sister to α-tubulin but BtubB formed a trifurcation with α-tubulin and β-tubulin, all being strongly mutually closer than to FtsZ/TubZ or centriole-associated tubulins (Findeisen et al. 2014). Another study by distance methods with or without local ML rearrangements put BtubA and B weakly sisters, their joint clade being sister to α-tubulin/β-tubulin; two technically better ML trees grouped BtubA and B individually with α-tubulin and β-tubulin (Yutin and Koonin 2012) consistently with other studies. Thus, all three analyses imply that BtubA and α-tubulin are related and diverged from BtubB and β-tubulin (also mutually related) in a single ancestral gene duplication and divergence that yielded the mt-forming heterodimer. Pilhofer et al. (2011) showed an ML tree where BtubA and α-tubulin grouped together but BtubB grouped together with both γ- and β-tubulin, but their exact positions within the clade were unstable amongst analyses; this instability was an inadequate reason for their unparsimoniously suggesting that BtubA/B and α-tubulin/β-tubulin arose from a homomeric ancestor by two independent gene duplications. A single ancestral duplication is more likely and consistent with the higher aminoacid identity of A with α and B with β than of A with B. Poor resolution on a tree should never be used as a reason for suggesting a more complex evolutionary pathway than other evidence requires—yet it too often is, notorious examples being longstanding resistance to accepting single origins of mitochondria, chloroplasts, and chromist membrane topology (Cavalier-Smith 2018). All four studies failed to give any evidence for LGT of Btubs from eukaryotes: i.e. they never grouped either B-tub within either α- or β-tubulin eukaryotic sequences, in marked contrast to β-tubulin of the proteobacterium *Beggiatoa* that groups shallowly within eukaryotic β-tubulin (Yutin and Koonin 2012), giving positive evidence of its LGT from eukaryotes. Because of that nested position of *Beggiatoa* β-tubulin and the fact that its α-tubulin and β-tubulin genes are not clustered together and are not known to make mts or even be expressed *Beggiatoa* probably acquired these genes (or pseudogenes?) by LGT.

Even though Jenkins et al. (2002) said their tree topology argued against eukaryote to *Prosthecobacter* LGT, Schlieper et al. (2005) ‘preferred’ the idea that *Prosthecobacter* got Btubs by LGT from eukaryotes merely because they are unique to *Prosthecobacter* and much more similar to α-tubulin and β-tubulin in structure than to FtsZ, especially in the C-terminal region that lies on the surface of mts and in eukaryotes interacts with motor proteins. However, all those facts are perfectly compatible with verrucomicrobial mini mts having been ancestral to eukaryote mts and are not phylogenetic evidence for LGT, contrary to their paper’s title. Pilhofer et al. (2007b) ‘assumed’ tubulin LGT from eukaryotes to *Prosthecobacter* merely because *Verrucomicrobium spinosum* lacks tubulin but *Prosthecobacter* and other Verrucomicrobia have FtsZ. Coexistence of both means that FtsZ cannot have been simply converted into tubulin, but does not exclude the possibility that a duplicate of it or more likely a planctobacterial TubZ was directly ancestral to α- and β-tubulins, and mts were lost by *Verrucomicrobium*, which we argue is most likely. They also thought that the Btub operons being in differing genetic environments in different species favoured LGT over vertical descent, which is fallacious. All it tells us is that their arrangement on the chromosome altered since their (quite recent) common ancestor and nothing about whether that common ancestor got it by LGT or vertically. After discovering Pmts, Pilhofer et al. (2011) rightly considered them ancient tubulins and that they could have been present in ancestral Verrucomicrobia (being lost by lineages without them) and ancestral to eukaryotic mts; curiously, though no longer arguing for LGT from eukaryotes, they raised the possibility of LGT from a yet unidentified bacterial lineage—a pointless complexifying speculation unlikely to be refutable or confirmable; neither a useful explanation which should explain things by known facts or principles, nor evidence for LGT. Despite lack of clear evidence for LGT from eukaryotes, McInerney et al. (2011) in trying to argue that all similarities between planctobacteria and eukaryotes are convergent cited only Jenkins et al. (2002) and Pilhofer et al. (2007b) in support of their claim for a eukaryotic LGT origin of BtubA/B.

Yutin and Koonin (2012) believed that their trees supported tubulin LGT to *Prosthecobacter* because Btub groups more closely with α- and β-tubulins than with FtsZ. But that is exactly what is expected if BtubA was inherited vertically from a common ancestor with α-tubulins and BtubB from a common ancestor with β-tubulin and Btubs are ancestral to eukaryote tubulins. Yutin et al. believed that their trees implied an archaebacterial origin of eukaryotic tubulin. We disagree, as we explain after considering why eukaryotes evolved wider mts and archaebacteria lost them. Both FtsZ and tubulins have an inherent ability to form either filaments or rings (Erickson and Stoffler 1996), but in eukaryotes, tubulin rings have opposite curvature to FtsZ (Housman et al. 2016)—we speculate that this key difference probably arose when Btubs duplicated and became heteromeric in planctobacteria, but their curvature sense and that of archaebacterial tubulins is unknown.

The smaller diameter and independence of their nucleation from γ-tubulin also support their intermediate character between FtsZ/TubZ and eukaryotic tubulin. We suggest that γ-tubulin evolved from BtubB, with which it shares several sequence motifs (and groups weakly on both CAT-GTR and ML trees when we align it with Btubs and α- and β-tubulin (with or without ε-tubulin; new trees using 444 amino acids rooted between BtubA and B, not shown)—by gene duplication in a stem eukaryote after its divergence from stem archaebacteria—and that it was modified so as to bind other proteins instead of BtubA and form γ-tubulin homooligomers able to nucleate 13-pf mts. The most obvious selective advantage of larger diameter mts would be greater rigidity and less bendyness, as would be newly advantageous when they acquired the novel mechanical function of pushing cell poles apart, i.e. mediating anaphase B (Scholey et al. 2016), previously argued to have originated at the cell surface before differentiation of the endomembrane system or origin of the nucleus and anaphase A (Cavalier-Smith 2010d). In effect, mt-based anaphase B replaced prokaryotic non-mt pole separation pushing mechanisms based on ParM or TubZ in plasmids or (non-cytoskeletal diffusion-ratchet) ParAB for the main chromosome (which archaebacteria and planctobacteria both retain) (Gerdes et al. 2010).

## Centrosomes, γ-tubulin, and the origin of premitosis

By premitosis, we mean chromosomal segregation by 13-pf microtubules pushing apart centrosomes at the surface of a pre-eukaryotic lineage that still had its DNA attached to the surface CM, not to endomembranes (see Fig. 3c of Cavalier-Smith 2010d), which theoretically should give the smoothest transition from prokaryote to eukaryote DNA segregation methods (Cavalier-Smith 1987a). γ-tubulin complexes were probably the key innovation that made centrosomes (Cavalier-Smith 2010d). Eukaryote mt nucleation depends on γ-tubulin complexes with gamma complex proteins (GCP2-6), a family of structurally related proteins (having at opposite ends grip1 and grip2 domains) that are bundles of helices that bind respectively to each other and to γ-tubulin (Farache et al. 2018; Sulimenko et al. 2017). GCP2 and 3 form heterotetrameric small γ-tubulin complexes (γ-TuSCs), whereas GCP4-6 aggregate these into ring complexes (γ-TuRCs). As GCP4-6 have been secondarily lost by budding yeasts and the *Trypanosoma* complex contains only CCP2-4, only 2/3 being essential for procyclic viability and flagella formation, we suggest that the simpler γ-TuSCs may have been sufficient for the transition from Pmts to eukaryotic mts and the evolution of mitosis and cilia, but with GCP4 at least being added preLECA. GCP2/3 have similarly simple domain structure so are likely to be ancestral: GCP 5 and 6 have different insertional elongations and GCP4 is N-terminally truncated, all arguably derived. We suggest that γ-TuSCs originated during the earliest phases of eukaryogenesis at the same time as CCT was differentiating its different subunit functions more than in their archaebacteria sisters. Eukaryotic CCT is a substrate-specific chaperonin ATPase essential for assembling α-tubulin/β-tubulin heterodimers, F-actin filaments and an array of protein complexes characterised by 7-bladed WD40 repeats that are either eukaryote-specific (e.g. APC, G-protein complexes, ER/Golgi coatomers) or became more complex in eukaryotes than in archaebacteria (e.g. nucleolar U3 snoRNPs). We suggest that the separate function of its subunits differentiated following gene duplications in prekaryotes as these novel functions arose during eukaryogenesis, and their coevolution with CCT was central to the associated proteome expansion.

We suggest that γ-tubulin evolved by gene duplication of BTubB not A because on some ML trees it groups with BTubB more closely than does β-tubulin (Pilhofer et al. 2011) not as sister to the whole α-tubulin/β-tubulin/BtubA/B clade as in most others. It has four arguably derived 1–3 amino acid insertions not present in FtsZ or αβ-tubulins. Possibly, these inhibited coassembly with αβ-tubulins in the mt but allowed homo-oligomerisation and nucleation of mts, possibly enhanced by novel binding to GCPs. We note that (in opisthokonts at least) a very different tubulin-binding protein family normally involved in stimulating growth at both ends of mts (Stu2 and relatives) can sometimes nucleate mts independently of γ-TuSCs or augment their nucleating function (Gunzelmann et al. 2018). Though BLAST shows distantly related proteins across all eukaryotes, it is unclear whether this γ-Tu-independent mode of nucleation exists in all eukaryotes; convincing Stu2 relatives were not found in prokaryotes so at present we do not give them a role in initial eukaryogenesis, but premitosis must have involved more proteins than we discuss.

**Fig. 13:**
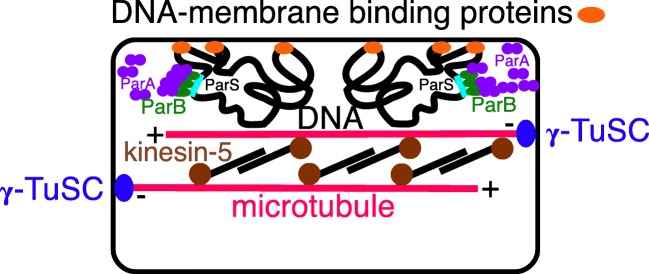
Origin of premitosis is crucial for eukaryogenesis. It is argued that prokaryotic ParABS-based DNA segregation and MinD-based cell polarity must have been retained for cell viability during eukaryogenesis when origin of centromeric nucleation of 13-pf mts by γ-TuSC originated a novel cell polarity mechanism, and origin of bipolar kinesin-5 enabled centrosome-nucleated mts to form an intercentrosomal mitotic spindle able to mediate anaphase B. Only then could anaphase A (not shown) evolve by conversion and/or replacement of ParB to/by inner kinetochore proteins and their slideable linkage by outer kinetochore Ndc80 rods to mt + ends depolymerisable by novel kinesin-8, as the text summarises. This sequence of events leaves no period during eukaryogenesis without a workable DNA segregation machinery and explains simply how both prokaryotic segregation and cell polarity mechanisms based on protein diffusion ratchets could be harmlessly replaced by radically different eukaryotic mt/kinesin motor mechanisms. Once centromeric mts took over the general polarity function of bacterial MinD they provided the structural framework on which other kinesin mt motors, then dyneins, could act and also stable polarity on a larger spatial scale than can prokaryotes by Min, MreB, and FtsZ, and thus help organise the now branched actin network

As the ancestral neomuran must have retained both mts (universal in eukaryotes) and FtsZ and MreB (both present in many archaebacteria and some Planctobacteria) and ParAB for DNA segregation (general in archaebacteria), the neomuran stem lineage likely retained a basic prokaryote-like DNA segregation and cell division machinery during earliest stages of premitosis evolution. In particular, we suggest that at first γ-TuSC assembly was geometrically positioned at both poles of a rod-shaped neomuran by the same polar positional information machinery (MinCD) that eubacteria use to move ParB and other proteins to poles. That would automatically have resulted in a bipolar premitotic spindle of antiparallel mts, nucleated at each pole at their minus ends by a γ-TuSC assembly functioning as primitive centrosomes (Fig. 13). Without any further new proteins, + end growth of these bipolar spindle mts would gradually push apart the centrosomes. If initially daughter DNA remained attached to prokaryotic ParB proteins and continued to be pushed to poles by ParA gradients throughout interphase, premitotic spindle dynamics could evolve in temporal overlap with the retention of prokaryotic segregation, so there would be no awkward hiatus in which mitosis would have to originate suddenly, as was the case before we realised that mts arose in planctobacteria before murein was lost (Cavalier-Smith 1981, 1987c, 1992a, 2002c, 2010d, 2014). Thus, planctobacterial origin of mts makes the prokaryote to eukaryote transition in cell growth and division mechanisms radically more gradualistic and so comprehensible than it once was—or would be if one supposes that eukaryotes evolved from archaebacteria, which lack mts, as many still do (Yutin and Koonin 2012; Yutin et al. 2008).

The premitotic spindle would have been more stable as an endoskeleton during interphase growth if the antiparallel mts were cross-linked by reversible cross bridges similar to kinesin-5 which does this in modern spindles (Sharp et al. 1999). A bipolar homotetrameric kinesin specific for antiparallel mts (kinesin-5: Acar et al. 2013) could have evolved more easily than most kinesins which are heteromeric and have tails that have to combine with light chains to help bind them to their cargo. A bipolar kinesin need only bind its head reversibly to mts and its tail to homologous tails so its origin would have been more self-contained. We therefore suggest that kinesin 5 was the ancestral kinesin and all others evolved from it by gene duplication, divergence, and evolving tails and light chains with varied cargo-specific binding properties—11 major kinesins evolved in the eukaryote stem lineage. Kinesin ATPases are thought to have evolved from a P-loop GTPase of the TRAFAC class, which also includes myosin ATPases, both of which must have switched their binding nucleotide from GTP to ATP (Leipe et al. 2002), rather than from the SIMBI class that includes other key cell cycle proteins such as the ParA/Soj subfamily and MinD that is involved in pole topogenesis in rod-shaped eubacteria (MacCready et al. 2017) as well as the FtsY and SRP54/Ffh subfamiles. Kinesins and myosins belong to the superfamily that also includes eukaryotic dynamins but is distinct from the translation factor and Ras-like and septin-like superfamilies (Leipe et al. 2002). As well as the dynamin (eukaryotes only)/YjdA eubacterial family, the kinesin/myosin superfamily includes the GB1 family, and the YlqF circularly permuted GTPase family. Prokaryote members of the YjdA family are more widespread in eubacteria than archaebacteria and suitable ancestors would have been present, possibly already with a motor function in Planctobacteria. We suggest that the shared deletion that removed the protein region including the ancestral GTP binding motif in kinesin and myosin (Leipe et al. 2002) occurred once only in the ancestor of kinesin; myosin evolved by duplicating it followed by insertions to lengthen it and make bipolar myosin II ATPase able to bind antiparallel actin filaments after formin evolved in the preeukaryote to nucleate unbranched actin bundles (see below)—possibly somewhat later than 13-pf mts. Myosin II plus the specific location of formins at the cell equator would be logically sufficient to evolve a contractile actomyosin ring for cytokinesis, which also needed septin GTPases to curve the septal membrane. Thus, origin of bipolar kinesins and myosins would establish both a spindle capable of anaphase B-like elongation and actomyosin cytokinesis machinery.

On that interpretation, bipolar kinesins and myosins had a common origin in prekaryotes as motors for premitotic spindle elongation and cytokinesis respectively. Prokaryotes lack both. As Bmts occur in planctobacteria and actin was ancestrally present in filarchaeote archaebacteria (Guy and Ettema 2011), whereas euryarchaeotes have FtsZ like eubacteria, origins of both mts and actin predated the origin of eukaryotes, so contrary to early ideas (Cavalier-Smith 1975) were not the key molecular innovations that made eukaryotes. Instead, the five key molecular novelties were the origins of novel mt and F-actin nucleation machinery (γ-TuSCs, formins (Chalkia et al. 2008; Skau and Waterman 2015)), bipolar molecular motor ATPases kinesin 5 and myosin II that mediated their sliding in antiparallel bundles, and coiled-coil septins (Cao et al. 2009). All evolved after murein loss, soon after prekaryotes diverged from archaebacteria. Dynamins stabilise the actin cytokinetic ring in podiate eukaryotes (Masud Rana et al. 2013) and dynamin-related proteins are required for green plant cytokinesis (Arakaki et al. 2017). We argue that dynamins evolved first for cytokinesis in prekaryotes from planctobacterial precursors before they later were coopted for membrane division by mitochondria, peroxisomes (Fujimoto et al. 2009), and other organelles, including endocytic clathrin-coated vesicle formation. Dynamin-related proteins are widespread in eubacteria but extremely rare in archaebacteria, the three cases being attributable to LGT from eubacteria, yet another reason why a negibacterial ancestry is simpler than an archaebacterial one. Claims that dynamin originated from mitochondria and its obvious eubacterial ancestry supports their idea that mitochondria preceded phagotrophy (Martin et al. 2015) are both fallacious, and stem from the arguably false assumption (Ku et al. 2015; Van Valen and Maiorana 1980) that archaebacteria are directly ancestral to eukaryotes. As explained above, the Margulis-derived mitochondria-first fallacy falls foul of the inability of prokaryotes to take up foreign cells; the assertion that ‘Clear examples for such symbioses do indeed abound’ are profoundly misleading and essentially false (Gould et al. 2016). None exists.

The three centriole-associated tubulins (δ, ε, ζ) must have evolved after mts and γ-tubulin even though paralogue trees rooted on FtsZ typically show them as branching below α,β,γ-tubulins. The crown branches of δ, ε, and ζ are much longer than for α,β,γ-tubulins, showing they have evolved faster, thus under fewer constraints. As their stems are also long, their lower position on these trees is almost certainly an artefact of LBA. They are found only in eukaryotes with centrioles and must all have originated before LECA, which was a biciliate with complex mt roots (Cavalier-Smith 2017), but have been lost in all lineages without centrioles/cilia (Turk et al. 2015). They are also all absent in *Drosophila* whose centrioles have doublets only in most cells but triplets in spermatocytes, so cannot be intrinsically necessary for making triplet centrioles. δ and ζ are sisters and can be lost independently in different lineages, e.g. placental mammals and the clubmoss *Selaginella* have only δ and marsupials only ζ, so they probably have equivalent functions, but in other eukaryotes with centrioles both are essential. ζ and ε are located at the distal end of the ciliary foot or centriolar appendage. Mutations interfere with positioning of centrioles and γ-tubulins in relation to cortical actin and mt skeletons, so these tubulins are probably involved in structural positioning of centrioles not their basic architecture even though triplet structure can also be upset in mutants. What macromolecular complexes they form is not known but we can infer that they are not so tightly specified as mts and nucleation complexes. We suggest that δ/ε-tubulin common ancestor and ζ-tubulin evolved in stem eukaryotes (not in prekaryotes) from different gene duplicates of BtubB or γ-tubulin after cilia evolved in the ancestral uniciliate so as to improve their anchoring to and positioning within the cortical skeleton. The too-deep position of centriole-associated tubulins on sequence trees illustrate the point that paralogue trees are often greatly distorted by rapid evolution and can be seriously misleading about the temporal order of events and need more critical interpretation than many realise.

## Planctobacterial origin of archaebacterial tubulins?

If as RP trees and our arguments on mt origins suggest neomura evolved from planctobacteria with mini mts, absence of mts in archaebacteria raises the question of the evolutionary origin of archaebacterial tubulins. Unlike eukaryotes and *Prosthecobacter*, archaebacteria have only a single tubulin: CetZ in many euryarchaeotes (Duggin et al. 2015), and artubulins in filarchaeotes (a few thaumarchaeotes (Yutin and Koonin 2012) and odinarchaeotes (Zaremba-Niedzwiedzka et al. 2017)); both evolve immensely faster than mt tubulins or FtsZ (faster in thaumarchaeotes than odinarchaeotes) and have such long branches on trees that they are even more likely to be misplaced though LBA than δ, ε, and ζ; both branch more deeply. Though it has been said they ‘appear to be intermediate in sequence and structure between FtsZ and tubulin’ (Amos and Löwe 2017), it does not follow that they are phylogenetically intermediate as Yutin and Koonin (2012) assumed for artubulins before CetZ were known. If the tree of Zaremba-Niedzwiedzka et al. (2017; supplementary Fig. 6a) were correctly rooted on *Prosthecobacter* tubulins, odinarchaeote (Asgard) tubulin would be part of the artubulin clade seemingly grouping with ε/δ tubulin, which we interpret as a long-branch artefact; if so, their statement that they are more closely related to eukaryote tubulins than artubulins is incorrect; on our interpretation, all archaebacterial tubulins should be sister to eukaryotic tubulins in the absence of such artefacts. CetZ homomeric protofilaments are responsible for maintaining the rod-shape of Halobacteriales (Duggin et al. 2015), and likely also for rod-like euryarchaeotes in general.

If as we argue Pmts are ancestral to eukaryotic mts, archaebacterial tubulins cannot be ancestral to mts. We suggest instead that the putatively hyperthermophilic stem archaebacterium lost membrane attached Pmts when their membranes were radically changed by replacing acyl ester lipids by prenyl tetraether lipids. We argue that they lost membrane-attachment protein BtubC and either BtubB or BtubA but retained the other formerly mini mt protein and adapted it as a homopolymeric filament for membrane support. This putative secondary shift from heteropolymer to homopolymer (we suggest by reducing the number of specific binding interactions of archaebacterial tubulin) could have released coevolutionary constraints on archaebacterial tubulins allowing them to evolve immensely faster. That artubulins appear as deep sister of eukaryotic tubulins, whereas CetZ branches one node deeper, implies that divergence from the ancestral state was substantially greater for stem euryarchaeotes than for stem filarchaeotes. It is well known that in eukaryotes after centrioles and δ-, ε- and ζ-tubulins were secondarily lost the evolutionary rate of α-, β-tubulins increased greatly in many lineages, sometimes causing misplacement on trees (Cavalier-Smith 2015). Losing BtubA would even more dramatically accelerate rates.

The alternative assumption that archaebacterial tubulins evolved from FtsZ, implicit in Yutin and Koonin (2012), does not explain why they evolved so much faster and is incompatible with the strong arguments for planctobacterial mts being primitive rather than secondarily derived after LGT. For similar reasons Jékely (2014) also questioned the assumption that artubulins are ancestral to eukaryotic tubulins, but postulated their LGT from a eukaryote. That would require even greater change in sequence than if their common ancestor with CetZ diverged as sister to eukaryotes. Our LGT-free interpretation is simpler.

## Planctobacterial origin of neomuran actin

In contrast to tubulin, archaebacterial actin filaments are so much more similar to eukaryote F-actin than to any eubacterial actin-like proteins that there is little doubt that they share a common ancestor that like both had a double helix of parallel pfs whose assembly is inhibited by binding a small protein, e.g. profilin in eukaryotes, arcadin 2 in crenactin (Izoré et al. 2016). As for archaeal tubulins, actins from the two phyla are so divergent that they do not consistently group together on trees. The presence of profilin in asgards (Zaremba-Niedzwiedzka et al. 2017) suggests that profilin inhibition of actin polymerisation evolved in stem neomura but was lost by euryarchaeotes. Most (not all) asgard profilins, which are sisters not ancestral to eukaryotic ones by sequence phylogeny, weakly inhibit polymerisation of rabbit actin and bind to it as a broadly similar complex, but unlike eukaryotic profilins do not bind polyproline motifs that are absent in archaebacterial actins (Akιl and Robinson 2018). We suggest that a primitive profilin-regulated actin skeleton evolved in stem neomura to stabilise cells when the planctobacterial precursor first lost murein and OM, but profilin was lost by ancestral euryarchaeotes when they re-evolved a rigid prokaryote wall but was retained by many filarchaeotes. The assumption that profilin arose first in asgards (Zaremba-Niedzwiedzka et al. 2017; Akιl and Robinson 2018) and that eukaryotes are their sisters ignores the possibility of gene loss which has been rampant in archaebacteria.

In an ML tree, *Thermoplasma* actins were weakly within the ParM clade (Yutin et al. 2009) but crenactins grouped with eukaryote actins plus actin related proteins (Arp2/3), whereas a more strongly supported and perhaps more reliable Bayesian tree placed the better sampled Thermoplasmatales outside ParM with maximal support but sister to it (Ettema et al. 2011). Wagstaff and Löwe (2018) asserted that *Thermoplasma* actin ‘is probably the result of horizontal transfer of a bacterial Alp (actin-like protein) into an archaeum’ citing only Ettema et al. (2011) and Hara et al. (2007), neither of whom mentioned LGT. On the contrary, Hara et al. (2007) interpreted their results as implying that the ancestral neomuran already had actin, also our interpretation of both papers. The tree of Ettema et al. (2011) contradicts the idea of LGT from Alps as it strongly excludes Thermoplasmatales from the ParM/Alp clade. Moreover, unlike ParM/Alp, which is a very long branch (in keeping with the fact that these genes are plasmid or phage encoded and such genes evolve faster than host cell genes (Derman et al. 2009)), Thermoplasmatales actin branch is much shorter, no longer than the MamK clade, so cannot have come from within the long ParM clade as Roeben et al. (2006) postulated (not because of phylogenetic evidence but just because of their great divergence from archaebacterial MreB and as *Thermoplasma* have plasmids they *might* theoretically have got it from a eubacterial plasmid!). These branch length disparities similarly show that the also relatively shorter eukaryote actin clade cannot have evolved from the faster evolving ParM.

We therefore argue that MamK must have been the eubacterial ancestor of all neomuran actins. MamK is known only from two eubacterial phyla, both gracilicutes: Proteobacteria and Planctobacteria. Some members of planctobacterial ‘subphylum’ Omnitrophica have magnetosomes and their MamK appears robustly sister to those of Proteobacteria (Ettema et al. 2011). As there is no evidence from RP trees for Proteobacteria being ancestral to neomura, but there is for Planctobacteria, actin probably evolved from planctobacterial MamK. Planctomycete actin is even closer (Shiratori et al. 2019).

The alignment of ParM with *Thermoplasma* and other neomuran actins and Arps and MreB (Yutin et al. 2009) shows that in indel structure ParM has two derived insertions not present in MreB that preclude it from being ancestral to eukaryote actin or Arps. Moreover, indels in *Thermoplasma* actin are generally more like those of crenactin than ParM or eukaryotes but have several unique features (Yutin et al. 2009) that imply great divergence from all other actins. We suggest that this great divergence caused it to group falsely with ParM by LBA rather than with crenactins which we argue were its historic sisters.

LBA is often a superior acronymic explanation of tree inconsistencies than LGT. One of us also previously made the mistake of tentatively attributing archaebacterial actins to ParM LGT (Cavalier-Smith 2009); actin and α- and β-tubulins (and ubiquitin; see below) must now be removed from that paper’s Table 1 list of 60 eukaryote-specific characters, but the total should be increased to 61 as cohesins and octoheteromeric CCTs were omitted and all tubulins were lumped, as were actin and Arps 1–3. Arp 2 and 3 each have unique insertions that mark them out as derived. Neither could be ancestral to actin, but both could have evolved from it in prekaryotes as argued previously (Cavalier-Smith 2006a, 2009, 2010d).

The presence of MamK homologues in Planctobacteria is another reason additional to those previously discussed (Reynaud and Devos 2011) why this negibacterial phylum is now more plausible than any posibacterium as the eubacterial ancestor of neomura. Given the key importance of the branched eukaryote actin cytoskeleton for the origin of phagocytosis and eukaryogenesis the evidence adduced here that actin as well as tubulin evolved from planctobacterial proteins strengthens our planctobacterial origin.

## Secondary origins of unipolar single head motors

Bipolar kinesin and myosins could generate single-headed descendants by mutating their tails, whether directly or by domain fusion, so as to introduce novel binding sites enabling their binding a variety of different cargos whilst simultaneously preventing dimerisation. Of special importance for initiating eukaryogenesis would have been the origin of single-headed myosin I, the key motor for phagocytosis (Dürrwang et al. 2006), which together with myosin II is one of five inferred to have originated before LECA, the others being IV, V-like, and VI (Sebé-Pedrós et al. 2014). Myosin VI is exceptional in moving towards the minus end of actin and possibly evolved from myosin II by losing much of its tail including the IQ domain; predominantly found in podiates with pseudopodia (not in fungi), it is possibly not an ancestral paralogue; its presence in haptophytes only amongst corticates might result from LGT (Sebé-Pedrós et al. 2014). Myosins IV and V-like appear phylogenetically slightly closer to II than to I consistent with the I II divergence being either particularly early or involving more substantial change. The presence of IQ domains in all these except possibly derived VI implies it was an ancestral myosin domain. We suggest that myosin I evolved in the prekaryote as soon as Arp2/3 static, branched cortical skeleton evolved and that it may have played a similar role to *Dictyostelium* myosin IB in recruiting Arp2/3 to the plasma membrane. If so, myosin I and Arp2/3 would both have been present before the origin of phagocytosis, making its evolution relatively simple. This scenario has the same logic but more detail than the earlier suggestion that actomyosin evolved for cytokinesis, then was recruited for phagocytosis and much later for amoeboid movement (Cavalier-Smith 2002c). In both, the prekaryote cell cortex was likely preadapted for the origin of phagocytosis by already having its key machinery, making the crucial step the signalling between prey binding and spatiotemporal coordination of the constituent processes.

On our hypothesis, bipolar myosins were ancestral, and cytokinetic, unipolar myosins evolved from them by modifying their tails to provide binding sites for a wide range of cargos, predominantly transport vesicles. The different tail domains (Richards and Cavalier-Smith 2005; Sebé-Pedrós et al. 2014) imply different cargos and preclude ancestral dimer formation as is needed for contracting unbranched filaments.

## Cytokinesis diversification in eukaryotes

As noted above, when the murein wall was lost, triggering the neomuran revolution though disrupting the eubacterial divisome in which actin homologue FtsA tethers FtsZ to the division site, FtsZ, MreB, and FtsA were retained by some archaebacterial lineages only, but lost by eukaryotes, and new ESCRT III proteins evolved in the neomuran ancestor. Not only did archaebacterial lineages diverge mutually in cytokinetic machinery as their walls diversified, but so did eukaryotes, e.g. myosin II here argued to be the ancestral myosin is found only in podiate eukaryotes (opisthokonts, Amoebozoa, Sulcozoa), in Percolozoa (Sebé-Pedrós et al. 2014) and one hemimastigophoran flagellate (Lax et al. 2018). Phylum Hemimastigophora, like scotokaryotes and Eozoa, lacks cortical alveoli, but is probably sister of Corticata (Lax et al. 2018), making it possible that myosin II was lost in the ancestor of Corticata when cortical alveoli evolved, whose novel attachment inside the plasma membrane probably necessitated a modification of cytokinesis. Myosin II is the key motor for cleavage furrowing in podiates (Nguyen et al. 2018) upon which our model for ancestral cytokinesis is based on the assumption that Percolozoa use the same mechanism (currently unknown). But eukaryotes without myosin II must use different motors.

In Euglenozoa, sister of Percolozoa, cytokinesis is more mt-dependent, the cleavage furrow being positioned by the mt-associated flagellar attachment zone (FAZ) filament (Farr and Gull 2012). The cleavage furrow protein CIF-1 coordinates many cleavage furrow proteins in trypanosomes, including KLIF that is required for furrowing and has a kinesin plus-end-directed motor domain and a tropomyosin-like domain that would enable it to interact both with mts and another myosin (Zhou et al. 2018). The FAZ cytokinetic machinery likely evolved in the common ancestor of Euglenozoa and Percolozoa as a discicristate synapomorphy as ciliary attachment zone architecture is present throughout Euglenozoa and in Percolozoa flagellate phases also (Cavalier-Smith 2017). We speculate that percolozoan flagellates with a similar mt-rich pellicle to Euglenozoa (Cavalier-Smith 2017) may use the same non-myosin-II cytokinetic machinery as trypanosomes, whereas the amoeboid phase might use the postulated ancestral form that we suggest evolved before cilia. If ancestral discricristates had both phases, Euglenozoa could have evolved by loss of myosin II and its cytokinetic machinery when ancestral Euglenozoa lost the amoeboid phase. The amoeboid phase of the percolozoan *Naegleria* though lacking centrioles has centrosomes containing γ-tubulin, pericentrin, and myosin II which duplicate every cell cycle and also nucleate centrioles during transformation into flagellates.

A few groups may have lost all myosins: Metamonada, some red algae (Sebé-Pedrós et al. 2014). The metamonad *Giardia* uses a combination of vesicle targeting and ciliary motility for abscission—actin appears to be necessary indirectly for cytokinesis, not for the cleavage furrow. *Giardia* has the most modified of all eukaryote actins and has lost several actin-binding proteins (e.g. formins that remain in another diplomonad) and greatly modified others (more remain than was once realised), likely a secondary result of extreme actin divergence. Apparent absence of myosin II in Corticata (Plantae, Chromista), Euglenozoa, Eolouka, and Neolouka (Metamonada, Malawimonada) could be explained by just five losses and replacement by independently evolved bipolar myosins. However, an alternative explanation requiring no loss or radical change in cytokinesis, is that myosin II became modified independently in each of these lineages so substantially that it cannot be recognised as being a divergent myosin II by either sequence trees or domain characteristics. Candidate bipolar myosins in these groups should be studied to see whether they mediate cytokinesis or whether any have truly myosin-free cytokinesis.

Model corticates like *Tetrahymena*, *Paramecium*, and *Chlamydomonas* need investigation of candidate bipolar myosin functions including cytokinesis to see whether they power their contractile actin rings. Myosin VIII specific for Viridiplantae (Sebé-Pedrós et al. 2014) has a similar long tail to myosin II likely compatible with dimerisation and possibly evolved from it by deleting the MyoN-term domain; it branches only one node away from myosin II so may represent a diverged derived cytokinetic bipolar myosin. Myosin Myo13 of *Tetrahymena* is similar and postulated to be bipolar unlike other ciliate myosins and thus a candidate for a cytokinetic bipolar myosin (Sugita et al. 2011); though more distant on sequence trees, basal resolution amongst classes is so weak that they cannot reliably reconstruct the phylogeny of the deepest branches and therefore almost certainly show as polyphyletic classes that historically were a clade (Richards and Cavalier-Smith 2005); unfortunately, Sebé-Pedrós et al. (2014) did not show their PhyloBayes tree or basal support values.

We do not know whether chromists and plantae share a single method ancestrally as it is poorly understood in all corticates except angiosperms whose cell plate machinery is so highly derived that it tells us little about ancestral corticates. *Chlamydomonas* cleavage furrows have both actin and tubulin and dynamin-like vesicle secretion as well as intraflagellar transport (IFT) proteins suggesting they all are involved in cytokinesis (Cross and Umen 2015). A role for actin was hard to establish as volvocalean algae have two actins (Kato-Minoura et al. 2015): conventional actin and a rapidly evolving actin paralogue that may function primarily in ciliary regeneration (Kato-Minoura 2005), but can substitute for standard actin in null mutants for several functions including cytokinesis (Hirono et al. 2003; Kato-Minoura et al. 1997; Onishi et al. 2016).

## Planctobacterial origin of eukaryotes: centrality of β-propeller/α-solenoid proteins

A planctobacterial origin of eukaryotes as shown by our eubacteria/eukaryote RP trees makes the already explained origins of endomembranes (Cavalier-Smith 2009) and consequential origins of mitosis and nucleus (Cavalier-Smith 2010d), and of cilia (Cavalier-Smith 2014) more gradual and less sudden than if they had evolved from a posibacterium as previously supposed primarily because they already had a single membrane. All three innovations depend on proteins with β-propeller and α-solenoid domains: many secretory or endocytic vesicle coat proteins, many nuclear pore complex proteins, and many ciliary IFT proteins essential for ciliary growth and origin have both β-propeller and α-solenoid domains in the same protein and many others have one or other such domains (Devos et al. 2004; Jékely and Arendt 2006). Eukaryogenesis therefore mandatorily required such proteins: Planctobacteria are the only prokaryotic phylum known to have both β-propeller and α-solenoid domains in the same protein (Santarella-Mellwig et al. 2010). Their presence in all three major planctobacterial clades (Planctomycetia, Verrucomicrobia, Lentisphaeria) apart from the secondarily simplified Chlamydiia that likely lost them (after their eukaryote hosts evolved) is strong evidence for the views that neomura evolved from Planctobacteria rather than posibacteria (Devos and Reynaud 2010; Reynaud and Devos 2011) *and* that archaebacteria are sisters not ancestors of eukaryotes (Cavalier-Smith 1987c). No other prokaryotes are anywhere near as good candidates for eukaryote ancestors.

McInerney et al. (2011) wrongly criticised that important conclusion on the grounds that repeats related to solenoid domains are also present in Bacteroidetes (Sphingobacteria). That is not suprising and merely shows that α-solenoid domains were in the common ancestor of Planctobacteria and Sphingobacteria (robust sisters on all our eubacterial trees) and therefore evolved before they were joined to β-propeller domains in Planctobacteria alone. The assertion that ‘there is no molecular phylogenetic evidence that would link any lineage of planctomycetes with eukaryotes’ (Martin et al. 2015) is now invalidated by our RP trees that place eukaryotes within Planctobacteria as sister to Planctomycetes (Figs. 8, S11, and 11). If planctobacteria are indeed ancestral to neomura, as all available evidence collectively strongly indicates, then this domain fusion occurred once only in the history of life in the common ancestor of Planctobacteria and neomura, not independently in eukaryotes and planctobacteria as gratuitously assumed by Gould et al. (2016); such proteins must have been lost in the ancestor of archaebacteria when they evolved hyperthermophily based on novel membrane lipids.

One β-propeller/α-solenoid membrane coat-like protein was shown to be located within the inflated periplasm, over a third of label being spatially associated with the CM (Santarella-Mellwig et al. 2010). We suggest that the planctobacterial ancestor had one such protein on its inner face that was concentrated in invaginations and supported that morphology much as does clathrin in eukaryote coated pits. This precursor was therefore preadapted for evolution of clathrin-coated vesicles. The GTPase dynamin that further constricts their neck had evolved later for prekaryote cytokinesis (see above). As explained above, ESCRT-III for final membrane scission to render the coated vesicle topologically distinct from the CM arose for neomuran cytokinesis and so was coopted for endocytotic scission.

Some planctomycetes were also trophically preadapted for evolving endocytosis: *Gemmata* uses energy to actively import environmental proteins across its OM for digestion in the periplasm, wrongly called ‘endocytosis-like’ (Lonhienne et al. 2010). To be endocytosis-like, it would have had to involve membrane budding and scission, and take place at the CM, not the OM, both untrue. It is equally misleading to call planctobacterial CM invaginations endomembranes; unlike eukaryote endomembranes, they are not topologically discontinuous from the CM, and thus cannot be regarded as endomembranes any more than can the respiratory CM invaginations in some proteobacteria or the chromatophore CM invaginations of Rhodobacteria (precursors of mitochondrial cristae: Cavalier-Smith 1983b, 2006b; Muñoz-Gómez et al. 2015, 2017), the magnetosome membrane invaginations of ‘Omnitrophica’ Planctobacteria and Proteobacteria or planctomycete anammoxosomes. Anammoxosomes oxidise ammonium to nitrogen gas, using a cytochrome-based respiratory chain to make up to 50% of atmospheric N_2_ (Ferousi et al. 2013; Grant et al. 2018; Neumann et al. 2014), but it is profoundly misleading to call them “the bacterial ‘mitochondrium’” (Jogler 2014). The anammoxosome membrane containing ladderene lipids and a membrane ATPase is topologically a CM (Neumann et al. 2014), evolutionarily equivalent to rhodobacterial chromatophores and mitochondrial cristae, not to the whole proteobacterial cell as are mitochondria. Its major metabolic enzymes are in the inflated periplasm, not the cytosol. Planctobacteria are not in any sense evolutionary intermediates between prokaryotes and eukaryotes and do not have eukaryotic characters as has so often wrongly been said with respect to their membrane topology and other characters. One lineage is phagotrophic (Shiratori et al. 2019)

A major interpretative error of electron micrographs not previously criticised is the claim that nuclear-pore-like structures occur in internal membranes of *Gemmata obscuriglobus* (Sagulenko et al. 2017). The structures sectioned in their Fig. 1A and B are not endomembranes but just the usual negibacterial CM invaginations; they have internal densities linking the two adhering membrane faces, but the pale oval area marked by the arrowhead is simply part of the periplasmic space between two adjacent membranes (topologically equivalent to the intracristal space of mitochondria) and nothing like the lumen of a Npc. Its dimensions are 6 by 12 nm, comparable to a ribosome not an Npc, whose diameter is ~ 130 nm. These structures are an order of magnitude too small to be related to Npcs. Moreover, there is not a scrap of evidence that they form pores connecting the cytosol on the outer side of the membrane complex with that on the nucleoid side; the membrane is continuous on both sides. Calling them pore-like is a serious error as is calling these membranes ‘nuclear envelope’. As Devos’s group has decisively shown (refs above), and one of us, TCS, explained to Fuerst when we met at Brisbane in 2005, these invaginations are NOT ‘nuclear envelope membranes’ but sheet-like CM invaginations more comparable to mitochondrial cristae; it was grossly misleading to call them nuclear envelope membranes. Contrary to the authors’ assertion, the diameter and pattern of densities is NOT ‘consistent with’ the larger annular structures with central density in their Fig. 1E. Nor is there any reason to consider the negatively stained membrane structures from lysed cells in Fig. 1 D/E to represent CM invaginations. Almost certainly they are OM ghosts, and thus a completely different membrane, as also are Figs. 3 and 4. Collectively, they reveal that there are two different kinds of cylindrical protein complex in the *Gemmata* OM. Both might act as pores or active transport complexes. Very likely one of them has the machinery for importing food proteins for digestion in the periplasm. The total diameter of the larger OM pore complex in Fig. 4C is ~ 23 nm and size of its ‘central pore’ only ~ 3 nm. Their Fig. 4A, B sections make it abundantly clear that the larger ‘pore complex’ is associated with a single membrane, almost certainly the OM, and does NOT traverse two membranes as do Npcs. Neither the authors nor the referees realised that these beautifully negatively stained structures are in the OM not in the CM invaginations and that they have nothing topologically, structurally, or evolutionarily in common with Npcs. Our interpretation can be tested by high-quality cell fractionation yielding pure OM and CM fractions and their concerted biochemical and EM analysis. These OM structures must all have been lost when the neomuran ancestor lost murein. They cannot be Npc precursors or homologues. The freeze-fractured cells of Fig. 2A and B both show that the major sheet-like CM invagination only partially invests the nucleoid area and that it is misleading to call it a ‘nuclear body organelle’. As it lacks Npc equivalents, it would kill the cell if it completely surrounds the nucleoid, as TCS told Fuerst in 2005. The ribosome-sized ovals in their Fig. 2A insets are hard to interpret, but smaller than the negatively stained OM complexes and much less regular, confirming they are not the same; those in Fig. 2C are nearly spherical and smaller (~ 9 nm) and thus probably enzyme complexes not ribosomes. Several different structures, none like Npcs, were conflated.

Setting aside such erroneous claims, Planctobacteria remain evolutionarily highly significant as likely precursors of eukaryotes having several key characteristics that made eukaryogenesis a much smoother, more gradual transition than would have been possible from any other prokaryotes. Contrary to myths that planctobacteria lack an OM, their OM has normal negibacterial β-barrel pores and they have LPS synthesis genes (van Teeseling et al. 2018).

## Planctobacterial origin of the endomembrane system

As repeatedly emphasised (Cavalier-Smith 2002c, 2006a, 2009, 2014), breaking membrane topology by the origin of phagocytosis and of coated vesicle machinery (that probably originated with receptor-based clathrin-like endocytosis) was the decisive step in eukaryogenesis. The necessary precursor was a eubacterium that already fed on extracellular proteins and already had the necessary precursors for the eukaryote cytoskeleton, endomembranes, and nuclei. It is now clear that planctobacteria, and only planctobacteria, fit those long recognised requirements far more closely than could have been predicted when the topological logic of the endocytotic interpretation of eukaryogenesis causality was adumbrated. The presence of so many types of CM invagination in planctobacteria—magnetosomes supported by actin-precursor MamK, respiratory anammoxosomes, and *Gemmata*-like invaginations involved in periplasmic digestion of extracellular proteins associated with β-propeller/α-solenoid membrane-coat like proteins (CPs), coupled with self assembling mts makes Planctobacteria (especially Verrucomicrobia) beyond any comparison the most likely ancestor of eukaryotes and makes the idea that they could have evolved instead from archaebacteria by engulfing a premitochondrion almost risible. If we properly invoke Occam’s razor and consider this evidence we can slice away all the other ideas listed by Martin et al. (2015) as scientifically grossly inferior.

A planctobacterium with these properties that lost murein and OM through mutating the bridges between them, thereby preventing their synthesis, would immediately become unimembranous; but could still have fed on extracellular soluble proteins during the transition to phagocytosis in the prekaryote lineage or to methanogenesis in the prearchaeal lineage. OM loss immediately caused a nutritional problem as most amino acids after digestion would be lost to the environment, loss being proportionally greater in regions where the CM was not invaginated. That would impose an immediate novel selective advantage for better spatial targeting of preexisting digestive proteases towards preexisting CP-associated invaginations and to recruiting preexisting dynamin and ESCRT-III to them to effect topological separation from the CM and thereby invent intracellular endomembrane precursors for the first time.

Previously, coated vesicle budding was assumed to have evolved immediately after the onset of phagocytosis (Cavalier-Smith 1987c, 2009); we now suggest it would have been easier for receptor-mediated endocytosis using preexisting CP-ancestors to have evolved immediately before phagocytosis. The key logical role of the origin of coated vesicles as the decisive factor in making ribosomal SRP receptor sites and DNA/membrane attachment sites permanent ER, immediately after they were internalised to yield a topologically separate endomembrane system (Cavalier-Smith 1987c, 2006a, 2009), is retained in the present scenario. But evolving coated vesicles slightly before (or no later than), phagocytosis would allow coated-vesicle-budding-based return of SRP- and DNA-free membrane to the plasma membrane from the very onset of phagocytosis.

As proposed previously (Cavalier-Smith 2009), preexisting proteasomes could have further eased this transition by coupling protein uptake to digestion in the cytosol. We suggest both improvements went on in tandem, one leading to differentiation of endomembrane types, the other differentiating eukaryotic proteasome subunits. Earlier, it was assumed that actinobacteria were the only eubacteria with proteasomes (Cavalier-Smith 2006d), but proteasome components can now be identified in GenBank in all eubacterial phyla from Chloroflexi, through Cyanobacteria, Endobacteria, Proteobacteria, and (most importantly) all subgroups of Planctobacteria: their seemingly significant secondary absence in *Bacillus subtilis* and *E. coli* were misleading consequences of highly incomplete sampling that led to Actinobacteria being wrongly singled out as the most suitable ancestors of neomura. Proteasomes therefore originated in LUCA and were present in Planctobacteria before OM loss; it will be good to see if any Planctobacteria already use them for digesting environmental proteins.

On the present scenario, actin nucleation by formins evolved before the Arp2/3 system used by phagocytosis, not the reverse as once suggested (Cavalier-Smith 2006a). But as soon as murein/OM were lost, the newly evolved neomuran N-linked glycoproteins could have been recruited as a CM receptor for binding other bacteria to the now wall-free prekaryote cell surface—the primary step in phagocytosis. Immediately, existing proteases could start digesting some of their surface proteins and the prekaryote could start to become a predator on other cells. Such predation could be made more effective by actin gene duplication yielding Arp2/3, making a branched cortical actin skeleton that is the mechanical basis for making lamellipodia able (initially partially, later completely) to envelop prokaryote prey. Additional enzymes and membrane-piercing proteins could be added to the repertoire to render more and more prey molecules available to the predator. Enwrapment and total engulfment would make a higher proportion of digested molecules available. Possibly Arp2/3-generating duplications to make a cortical branched actin gel arose in the prekaryote simply to stabilise its cell surface before phagocytosis even started to evolve. If so, the key initiating steps were simply use of glycoprotein receptors for binding cells and spatial control of Arp2/3 actin nucleation to localise lamellipodial growth around bound prey, which probably involved transmembrane signalling that is now so much more highly developed in eukaryotes than in prokaryotes. After this critical step, endomembrane differentiation could have occurred much as previously explained in enough detail to need no repetition (Cavalier-Smith 2009). The main difference here is that substitution of planctobacteria for posibacteria as ancestors makes earliest stages much smoother and evolutionarily comprehensible as mts evolved before neomura and actin evolved from MamK not MreB, so both persisted through the neomuran revolution. As mts apparently evolved before, not after, phagocytosis they provided additional *cortical stabilising*, and premitosis did not have to evolve in a sudden rescue after phagocytosis.

## Specious objections to phagocytosis-mediated eukaryogenesis

Martin et al. (2017) supposed that if the prokaryote ancestor of eukaryotes used chemiosmotic coupling to make ATP at its CM the origin of phagotrophy would make it digest its own ATP synthesis machinery! If that were true, they argue it would be so harmful that phagocytosis could not have evolved before mitochondria provided ATP. But it is not true; that absurd suggestion overlooks the fundamental fact that phagocytic eukaryotes do *not* digest the food vacuole membrane, but recycle it after it fuses with lysosomal or other endomembranes. The pioneering paper on the origin of phagocytosis explicitly pointed out that the phagosome membrane would, after *digestion of its contents only* by enzymes secreted across its membrane, have been recycled to the cell surface (initially by direct refusion, later by smooth vesicle budding and fusion) and so the first phagotroph would never have digested its own phagosomal membrane (Cavalier-Smith 1987c); though citing this paper, they either forgot its key contents or deliberately distorted phagotrophic theory. Furthermore, phagotrophic planctobacteria avoid harmful self-digestion of chemisomotic ATPases: see final section. Also Cavalier-Smith (1987c) argued that the prekaryote that evolved phagocytosis must already have had cortical mts and ingestion must have been *localised *between them, so most chemiosmotic machinery would not have entered phagosomes, and localised cytostomes arose early. Cavalier-Smith (1981, 1987c) always argued that the first eukaryote cannot have been a formless amoeba, randomly ingesting over its whole surface as Haeckel (1866) and so many diagrams since (including fig. 1 of Martin et al. 2017) imagined, because continued cell viability during the transition from prokaryote to eukaryotic DNA segregation requires mts before phagocytosis. *Prosthecobacter* mts now confirm the idea that cortical mts existed before phagocytosis and that ingestion must have been localised.

The anaerobic syntrophy hypothesis (Martin et al. 2017) lacks a mechanism for mitochondrial uptake and fails to explain what removed ribosomes from the ancestral CM, as phagotrophy theory logically explains by phagotrophic internalisation plus coated vesicles being a selective filter during the recycling process (Cavalier-Smith 2002c). These authors seem never to have understood the basic logic of those papers and did not cite another that explained endomembrane differentiation in far more detail and more logically than any of theirs, suggesting that SNAREs may have evolved in the first direct recycling stage and discussed membrane recycling evolution in detail (Cavalier-Smith 2009). If as our trees suggest, the eukaryote root is within or beside Eozoa, then it follows that LECA was a biciliate eukaryote with localised ingestion, either via a cytostome or a feeding groove. This plus *Prosthecobacter* implies that all stem eukaryotes and prekaryotes had cortical mts and localised ingestion. It is odd that Martin et al. (2017) suggest that phagocytosis evolved in association with an excavate feeding groove—thus strictly localised—yet make the spurious and contradictory suggestion that it would digest all chemiosmotic proteins! Cavalier-Smith (2002c) explicitly argued that the ancestral neomuran was most likely a facultative aerobe with oxidative phosphorylation at the cell surface. That capacity would not have been destroyed during the transitional stage, especially if mts prevented phagotrophy in places and there were numerous invaginations as is characteristic of Planctobacteria. Claiming that phagotrophy first requires an ATP source other than chemiosmotic coupling at the cell surface to allow acidification of the food vacuole (Martin et al. 2017) was wrong.

Claiming that ‘phagocytosis demands the full complexity of a eukaryote cell’ (Martin et al. 2017) is tendentious nonsense, aiming to bolster Martin's mechanistically untenable ideas that mitochondria originated first and phagocytosis could not have done. LECA had mitochondria and cilia, extremely complex structures, neither necessary for phagocytosis. Numerous non-ciliate amoebae can phagocytose, as can numerous anaerobic protists without mitochondrial energy generation, including the oxymonad, metamonad protozoan *Monocercomonoides* that has lost every trace of mitochondria (Karnkowska et al. 2016). That also puts the lie to the profoundly mistaken claim that mitochondrial energy production is essential for and caused eukaryotic genomic or cellular complexity (Lane and Martin 2010). No major innovation had at its start the full panoply of molecules and controls that it acquired when fully evolved. No evolution would be possible if complex processes could not start simply with immensely fewer components. Essentials of phagocytosis are binding prey, lamellipodial growth, and fusion around it to generate a phagosome, intraphagosomal digestion, and export of products into cytosol. These do not intrinsically require other complex eukaryotic features like mitochondria, cilia, nucleus, meiosis, and syngamy.

Martin et al. (2017) and Gould et al. (2016) attempt to imply that the derived nature of *Monocercomonoides* makes it irrelevant to their thesis. That is untrue; it is highly relevant and is a decisive counterargument disproving the central claim of Lane and Martin (2010) for the reasons for large eukaryote genomes and complex cells. Of course it does not support the defunct idea of extant ancestrally amitochondrial eukaryotes (Cavalier-Smith 1983a, b), which Cavalier-Smith abandoned 20 years ago (Cavalier-Smith 1998a, b) and was never logically necessary for the much earlier ideas of De Duve, Stanier, and Cavalier-Smith that origin of phagocytosis was the key transformative event in eukaryogenesis—still the only unrefuted logical explanation for the origin of the endomembrane system and nucleus.

The Martin et al. (2017) paper is riddled with oversimplified dichotomies and false and/or tendentious historical statements. One is that ‘the concept of phagocytosing archaea is so deeply engrained in thoughts about endosymbiotic theory’. Martin ought to know that Cavalier-Smith who has written most extensively on phagocytosis, endosymbiosis, and eukaryogenesis and eukaryotic organelles for 50 years *never* invoked archaebacterial phagocytosis, nor ever thought archaebacteria were ancestral to eukaryotes by any mechanism (as Martin and many others still erroneously do). Another in the section on Cavalier-Smith’s “Archezoa concept” (a misleading term he never used) is ‘Ideas designed to derive a phagocytic host were not based on data or observations in nature but rather from expectations generated from endosymbiotic theory, which suddenly needed such a cell for the sole purpose of acquiring mitochondria’, which is false and rather insulting. The organismal features of Archezoa came directly from data and observations.

Martin et al. (2017) pretend that ‘Phagocytosis-first theories predicted that eukaryotes lacking mitochondria should be primitively amitochondriate’. Untrue; that was not said by Stanier, De Duve or Cavalier-Smith. In proposing for the first time the idea that the mitochondrial OM was of purple bacterial OM not host origin (now universally accepted) and arguing against Margulis’ ‘mitochondria first’ ideas, Cavalier-Smith (1983b) hypothesised that eukaryotes evolved long before mitochondria, and referred for the first time to hypothetically primitively amitochondrial eukaryotes as Archezoa. The accompanying taxonomic paper formally established Archezoa as a protozoan subkingdom and ranked archaebacteria as subkingdom and considered them sister to eukaryotes (probably correct) and both younger than Eubacteria (correct) (Cavalier-Smith 1983a). It stated ‘we must take seriously the possibility that some of, or even all, the Archezoa are primitively mitochondrionless, and ask whether there is any firm evidence for the loss of mitochondria’ and called it a ‘taxonomic hypothesis, which can in principle be refuted or strengthened’. Neither paper predicted that all amitochondrial eukaryotes were primitively so or implied that the validity of ‘phagotrophy first’ depended on that being true. Moreover, the hypothesis of a long delay between these events was based (as explicitly explained: Cavalier-Smith 1983b) not on endosymbiotic theory but on the fossil record which palaeontologists had misinterpreted as showing eukaryotes as early as 1500 My ago long before the post 750 Ma major protist diversification. Ever since he realised that palaeontologists had seriously misinterpreted the sparse 1500 My data (Cavalier-Smith 2002a), that reason for invoking a long delay disappeared, it becoming likely that origins of nuclei and mitochondria were near-contemporary consequences of the phagotrophic origin of the endomembrane system and intracellular digestion (Cavalier-Smith 2002c), which was also favoured by amitochondrial eukaryotes having turned out to be secondarily anaerobic (nearly all having hydrogenosomes or mitosomes of mitochondrial origin). Even earlier, he argued from their double envelopes that trichomonad hydrogenosomes were of mitochondrial origin (Cavalier-Smith 1987d) (first to argue they were not an independent symbiosis) over a decade before sequences proved that correct. Implying that ‘phagotrophy first’ predicted that all amitochondrial eukaryotes must be primitively so, and would be disproved if none were, seriously misrepresented the theory. These authors earlier falsely equated ‘phagotrophy first’ with ‘Archezoa theory’, asserting it ‘was rejected over a decade ago because its predictions failed’ (Gould et al. 2016); that also was seriously misleading—rejection of the primitiveness of extant amitochondrial eukaryotes in no way contradicts ‘phagotrophy first’. Martin et al. (2017) claimed that the hydrogen hypothesis (Martin and Müller 1998), which itself seriously multiply misrepresented phagotrophy theory (see Cavalier-Smith 2002c), ‘explicitly predicted that any eukaryotes lacking mitochondria should be the result of secondary mitochondrion loss’. In fact, that was already known from phylogeny so was not a prediction; moreover, they wrote ‘many, and probably all’ not ‘any’; their last paragraph did make three explicit predictions, none yet confirmed. Our RP trees argue fairly strongly against the first: that comparative genomics should show ‘a strictly H_2_-dependent’ ‘probably a methanogenic ancestry’ for eukaryotes (Martin and Müller 1998).

Gould et al. (2016) asserted that studies of enzyme phylogeny ‘indicate that mitochondria predated peroxisomes in evolution, which is consistent with our model’—tendentiously misleading. Such studies claimed to show a few peroxisomal proteins were of α-proteobacterial origin (some supposedly from other eubacteria) (Bolte et al. 2015; Gabaldon et al. 2006). Even were that true it would not mean that mitochondria came first. They might both have evolved simultaneously, both after phagotrophy, as part of compartmentational origin of these two respiratory organelles to improve efficiency of energy extraction from engulfed food, as argued by Cavalier-Smith (2002c, 2014). These studies provide no evidence that mitochondria evolved before phagotrophy so offer no support to that improbable thesis. They fell short of demonstrating an α-proteobacterial origin of these enzymes because of poor taxon sampling and multiple paralogue, poorly resolved trees, making their interpretation hard. In particular Gabaldon et al. (2006) studied only opisthokont peroxisomal enzymes (none from deep-branching eukaryotes) and did not list eubacteria used to search for homologues, and showed a tree for only one of the enzymes claimed of α-proteobacterial origin (NADH diphosphatase); that tree included only two eubacteria other than α-proteobacteria, so does not exclude the possibilities that this enzyme might be of planctobacterial origin or was transferred to opisthokont peroxisomes from mitochondria after LECA. Bolte et al. (2015) listed the eubacteria included in their search, including 13 α-proteobacteria but only 8 representatives of only 4 other of the 14 eubacterial phyla recognised here; no Planctobacteria except genomically reduced *Chlamydia* were included, so it cannot be excluded that some studied proteins occur also in Planctobacteria and they are as closely related to peroxisomal ones as are α-proteobacteria. What appears to be the case is that lipid β-oxidation enzymes (likely the primary selective advantage of peroxisomes: Cavalier-Smith 2002c) probably had a eubacterial not an archaebacterial origin. Furthermore, some of the peroxins that mediate import into peroxisomes are related to the ubiquitin-dependent ERAD Cdc48 motor that pulls proteins out of the ER for proteasomal digestion, which has homologues in archaebacteria (not identified in eubacteria), suggesting a common origin. That fits the view that ubiquitin-labelled proteasomal digestion, ER, and peroxisomes all evolved together as an early consequence of the origin of phagotrophy and improved protein digestion machinery (Cavalier-Smith 2009).

Gould et al. (2016) mistakenly claimed it was ‘seldom if ever asked’ where energy for prelysosomal acidification came from on the phagotrophy theory. In fact, Cavalier-Smith (2002c) explicitly discussed the phagocytic relocation of the V-ATPase homologue to endomembranes and argued that the prekaryote ancestor already had aerobic respiration before the origin of mitochondria, whose main benefit was compartmentation (Cavalier-Smith 2006c, 2007a). Contrary to Gould et al. (2016), the earliest known host to harbour mitochondria was a phagotrophic biciliate protozoan (most likely an eozoan; see Fig. 8 of Cavalier-Smith 2017) not a ‘hypothetical’ phagotroph.

## Fatal flaws in two recent endomembrane origin speculations

After it was discovered that negibacteria can produce OM vesicles (OMVs) **(**Schwechheimer and Kuehn 2015), Gould et al. (2016) speculated that such vesicles generated by an α-proteobacterium hypothetically endosymbiotic with an archaean might have generated the rough endoplasmic reticulum (ER) by acquiring SRP receptors from the host CM. This ‘model’ fails as an explanation of ER origin by not accounting for its key properties already well explained by classical phagotrophy theory (Cavalier-Smith, 2002c). It fails mechanistically; no selective advantage is given for any key steps nor any physical mechanisms. No mechanism is given for the initial proteobacterial uptake (likely impossible, see above). None is given for how OMVs acquired an ability to grow by insertion of acyl ester lipids whose genes were located in the proteobacterium and enzymes in the proteobacterial CM; they don’t even mention this fundamental requirement let alone give a mechanism. OMV vesicles therefore could not grow and multiply in the host cytosol, so it is wrong to imply that they are equivalent to ER. No mechanism is given of how OMVs acquired SecY/Sec61 from the host CM, nor for why that non-existent mechanism did not similarly place them in the mitochondrial OM converting it to rough ER, nor how they were lost by the host CM. No mechanism is given for how OMVs acquire eukaryotic machinery for exocytosis, needed if they were to fuse with the host CM and to replace archaeal by eubacterial lipids.

Their model inverts classical logic by putting coated vesicle origin at the end of the process after evolution of the nucleus, making the whole idea illogical and devoid of selective advantage, just as was done by an earlier failed ‘explanation’ for the nucleus (Martin and Koonin 2006), whose fundamental defects were shown in detail (Cavalier-Smith 2010d). How could the plasma membrane become different from the ER in the absence of coated vesicle budding? They assert that the host CM proton-pumping translocase was retargeted to an OMV-derived ER but give no mechanism, and fail to acknowledge that no such targeting was necessary on the classical theory as the lysosome evolved from the food vacuole in a simple step (Cavalier-Smith 1987c). Given that neither selective advantages nor mechanisms are given, and that all these problems were more simply solved by a cell biologically plausible phagotrophy-first explanation forty years ago, it seems perverse to propose such a causally vacuous ‘model’.

Baum and Baum (2014) floated a topologically ingenious but mechanistically dubious inside-out model for eukaryogenesis that posits nuclear pore complex (Npc) evolution before any endomembranes or vesicle budding as a consequence of the postulated origin of protoplasmic protrusions and lateral growth in a prokaryote precursor. Topologically and geometrically, this resembles the mode of segregation between the ectoplasm and endoplasm of ectoretan Rhizaria (foraminifera, some Radiozoa) where a double membrane central capsule with large cytoplasmic pores separates an outer organelle-free and inner organelle-rich zone (Cavalier-Smith et al. 2018). However, that developmental mode depends on eukaryotic reticulopodial formation and preexisting endomembrane, which the supposed precursor archaebacterium with S-layer lacked. They assume the ancestor was an archaebacterium that fused β-propeller domains (preexisting in archaebacteria) to α-solenoid domains independently of Planctobacteria, but the initial stages of their model could instead be applied to a mutant planctobacterium once it lost murein/OM, which as explained above would be phylogenetically greatly preferable. This heterodox model is less obviously impossible than most one-off attempts to explain eukaryogenesis and merits some consideration. But four major flaws make it explanatorily inadequate and evolutionarily incredible.

First, through assuming membrane continuity from CM to protonuclear envelope throughout all the early steps and deferring the origin of membrane budding until much later and the origin of actin and myosin till the very end of eukaryogenesis they attempt to develop a cell of extreme topological simplicity (just one membrane) but immense geometric complexity without any cytoskeleton except Npcs and perinuclear cisternal LINC complexes. This may seem plausible in a sectional diagram, but we doubt that such a cell could exist mechanically without a skeleton and maintain such complexity. Secondly, how could it segregate its DNA and divide without either an actin or mt skeleton? The paper does not even mention when they think mts evolved, how or why, or say anything about evolution of actin and tubulin homologues during eukaryogensis, as we have in detail (proving that both actin and tubulin evolved before eukaryogenesis, refuting their scenario). Thirdly, though claiming to give selective explanations of all successive steps, they do not. The only selective force plausibly invoked is a presumed advantage of the first step of starting to invest the mitochondrial ancestor by blebs to improve syntrophy efficiency. No selective advantage or credible mechanism is given for the topological separation of CM and ER which is attributed to dynamin-mediated membrane scissions—with such a large number of channels linking the CM to the nucleus, it seems highly unlikely that they all could have been simultaneously cleaved by what they inappropriately call a ‘phagocytosis-like’ mechanism even had it any logical selective reason. They avoid explaining how phagotrophy evolved or recognising its great trophic advantages. Whenever they propose any innovation, the phrase ‘it is easy to see how ... could’ is repeated without a physical mechanism or selective advantage being made explicit. Likewise ‘selective pressure’ assertions avoid specifying how such an innovation would increase reproductive success, which is how selective processes actually work (Cavalier-Smith 2010c). Fourthly, reasons given for preferring this idea over other ideas are so vague or involve such false dichotomies as to be non-discriminating. What they lump as outside-in theories are so varied themselves that most have very different weaknesses and need individual not global criticism. In general they uncritically accept the syntrophy idea that an archaebacterium engulfed a proteobacterium by purely formal elaborations of its cell surface to make that appear plausible. Their assertion that ‘a significant fraction of eukaryotic genes assigned a function in lipid metabolism and transport have their closest prokaryotic relatives in α-proteobacteria (Thiergart et al. 2012) ... strongly suggests that eukaryotes acquired their bacterium-like lipids from mitochondria’ is mistaken. In fact, a much larger number of such genes have closest relatives in β+γ-proteobacteria (Thiergart et al. 2012).

## Planctobacterial ancestry of eukaryote phosphoinositides?

Phosphatidylinositol acyl ester lipids are universal in eukaryotes and their phosphoinositide metabolism much more complex than in prokaryotes, podiate eukaryotes having additionally evolved inositoltriphosphate ‘second messenger’ plasma membrane signalling (Michell 2008). Phosphatidylinositol prenyl ether lipids with the same headgroups (architydyl) are present instead in both archaebacterial phyla. Phosphatidylinositol acyl ester lipids are absent in *E. coli* and *B. subtilis* and have been well studied only in actinobacteria. It was formerly thought that they were absent in other phyla making actinobacteria the best candidates for ancestors of neomura (Cavalier-Smith 1987d, 2002c), but scattered presence of inositol metabolism in other eubacterial phyla shows it to be more widespread (Michell 2008). Phylogenetic analysis of homologues made it highly likely that the hyperthermophilic eubacterium *Thermotog*a got its *myo*-inositol 1P synthase gene (*ino1*) by LGT from a euryarchaeote hyperthemophile (Nesbø et al. 2001). However, the claim that all eubacteria got this gene by LGT from archaebacteria was unjustified, as their tree was wrongly rooted between eukaryotes and prokaryotes and much harder to interpret than they imagined, prokaryotes likely having three extremely divergent paralogues each with such long stems and so divergent from the also ultralong-stem for eukaryotes as to make it subject to LBA paralogue rooting artefacts. They found one paralogue (prokaryote group 2 subtree) in both crenarchaea and euryarchaea and three eubacterial phyla (Chloroflexi, 3 groups of Actinobacteria, Aquificia). This is likely to be the ancestral paralogue and shows no evidence of LGT and should be rooted on Chloroflexi and is congruent with our RP prokaryote trees; it has a single bipartition between eubacteria and archaebacteria, consistent with our evidence that archaebacteria evolved vertically from within neonegibacteria. Prokaryote group 3 comprises only the euryarchaeote *Archaeoglobus* and a different (actinobacterial) *Streptomyces coelicolor* paralogue than the ancestral one; it gives no evidence for LGT—we interpret it as a separate early diverging paralogue. Even though if the tree were rooted as it should be within paralogue 2, paralogue 3 would appear to be sister to eukaryotes, that position is likely to be a systematic LBA artefact as the stems are so drastically stretched. Paralogue 1 has only *Thermotoga* and euryarchaeotes *Pyrococcus* and *Aeropyrum*; we suggest it is an especially hyperthermophilic version that evolved by duplication of paralogue 1 after euryarchaeotes diverged from crenarchaeotes, this adaptation being linked to abnormally rapid amino acid substitution. Our BLAST-P analysis using phosphatidylinositol synthase of the actinobacterium *Rhodococcus rhodochrous* (presumably paralogue 3) as query identified convincing homologues in all major eubacterial phyla including all subgroups of Actinobacteria. Inositol monophosphatase that makes free inositol in eukaryotes is also annotated in GenBank for all eubacterial and archaebacterial phyla. Therefore inositol and phosphatidylinositol synthesis dates back to LUCA and was most likely inherited directly by neomura from a planctobacterial ancestor. Phosphatidylinositol is no longer a reason to favour posibacteria as ancestors; it is a common phospholipid in the aquithermote *Thermodesulfatator* (Moussard et al. 2004). It remains a reason to reject archaebacteria as direct ancestors as they lack the acyl ester version which is also so rare in α-proteobacteria that it is unlikely (as Martin’s ‘mitochondria first’ scenario claimed) to have entered eukaryotes via mitochondria, which themselves essentially lack such lipids (Michell 2013). Michell (2008, 2013) assumed that phosphatidylinositol originated in archaebacteria and before LUCA (phylogenetically contradictory assumptions), based on the misinterpretaton of Nesbø et al. (2001) and erroneous assumption of archaebacterial ancestry for eukaryotes. Eukaryote phosphoinositide functions depend on many β-propeller proteins binding to them (Michell 2013).

## Planctobacterial origin of mitosis and dyneins: dynein RP coevolution

Very likely premitotic spindle mts were more stable through the cell cycle (like in *Prosthecobacter*) than are mitotic mts. If during the transition chromosomes maintained attachment to them and origin segregation via ParA, premitosis also could have been gradual through the cell cycle, not sudden as in eukaryotes. Thus, for a period prokaryote attachments of the chromosome to the cortical mt and actin skeleton as well as to the CM would have made chromosomes more resistant to the DNA-attachment regions of the CM being rapidly internalised by onset of phagocytosis in a way traumatic to proper chromosome segregation. Thus, endomembrane differentiation likely evolved in two distinct phases. First, whilst DNA was still attached to the CM on part of the cell surface, phagocytosis being restricted to another with less robust cortical skeleton—much as many flagellate protozoa (including all eozoan flagellates) today display such regional differentiation that allows the conflicting requirements of cortical stability and local ingestion, unlike aciliate amoebae that are a bad model for the first eukaryote (Cavalier-Smith 1981). If so then there was time for endomembranes and intracellular digestion and the ubiquitin system controlling targeting to proteasomes to become quite sophisticated before accidental internalisation of DNA-attachment sites initiated the second phase of mitosis origin (kinetochore mts and anaphase A) by making concerted evolution of nuclei and true mitosis imperative.

Tromer et al. (2019) inferred that the kinetochore itself had 52 proteins in LECA, likely an overestimate if the eukaryote root is within or beside Eozoa contrary to their assumption, but a good estimate for the cenancestral neokaryote. As many kinetochore proteins (KPs) are related by gene duplications, stem eukaryotes must have had successively fewer as one gets closer to their common ancestor with archaebacteria. A few stem eukaryote ancestral KPs appear to be of stem neomuran origin, notably histone-dervived CenpA and CenPS/T/X/W, proteins related to E2 ubiquitinating enzymes (with plausible precursors in planctobacteria as noted above; ancestral to eight non-catalytic KPs), relatives of TATA-box-DNA-binding proteins, and HORMA-domain nucleotide sensing proteins. HORMA domains are present throughout eubacteria, but not archaebacteria (other than halobacteria that presumably got them by LGT and are unrelated to eukaryotes). HORMA proteins (often modified by closely linked Trip13 AAA+ ATPase) evolved at least as long ago as the common ancestor of Cyanobacteria and Actinobacteria, so the two HORMA KPs (and relatives for autophagy, DNA repair, and meiosis) are yet more eukaryotic proteins that cannot have been inherited from archaebacteria but could easily have come vertically from Planctobacteria. Paralogue 2, more closely related to eukaryotes than apparently older paralogue 1, is in planctomycetes, sphingobacteria, proteobacteria, and actinobacteria, but eukaryote HORMAs do not group specifically with any of these as their common stem is so long, another example of long-branches appearing more distant from their ancestors than they really are; these trees cannot tell us whether HORMAs arose from mitochondrial ancestors or from the planctobacterial ancestor of the nucleocytoplasm, as we consider more likely as chromosome segregation must have been more important than mitochondria for prekaryotes.

Most KPs were recruited from novel eukaryote-specific proteins whose relatives are involved in functions as diverse as centrosomes, intraciliary transport, nuclear transport, vesicle transport, chromatin structure, and DNA repair and replication (Tromer et al. 2019), further supporting our longstanding view that these evolved essentially simultaneously and drew on a common pool of novel eukaryotic proteins. There are also seven protein domains found only in kinetochores, i.e. novel domains, presumably arising by more drastic modification of unknown stem eukaryote proteins. This study further emphasises that archaebacteria are fundamentally prokaryotic in chromosome segregation and that radical evolution of extremely complex kinetochores was essential for eukaryogenesis. Logically, the most fundamental KPs are those binding to centromeric DNA (five Cenps) and those binding mts; both must have been present at the beginning. Most others were likely interpolated between them as mitosis rapidly improved to allow more reliable regulation and decrease the frequency of missegregation, probably the major selective force behind the great increase in complexity between the first and last eukaryotic common ancestor. To reconstruct intermediate stages, we must more firmly position the eukaryotic root and thereby determine the true number of LECA KPs and functions of all on both sides of the root; function is mainly known only in opisthokonts, and that only partially.

Origin of the nucleus by recruiting β-propeller/α-solenoid CPs to make the initial scaffold of the nuclear pore complex would have happened as explained in detail earlier (see Cavalier-Smith 2010d, not repeated here) almost immediately following internalisation of DNA-attachment sites. At that time, perhaps the most critical innovation to prevent missegregation would have been chromosome attachment by a kinetochore directly to the + end of a centrosome-nucleated mt followed by evolution of anaphase A (kinetochore mt shortening by controlled depolymerisation). This attachment is mediated by a rod-like Ndc80 protein complex attached at one end to the mt and at the other to inner kinetochore proteins in such a way that + ends can lengthen or shorten without breaking the slideable attachment of Ndc80. Origin of Ndc80-like proteins in LECA (D’Archivio and Wickstead 2017) was the crucial step in evolving mitosis anaphase A, together presumably with inner kinetochore-like proteins whose early evolution is less clear in eozoa, perhaps because they may have been secondarily modified in trypanosomes if they secondarily lost CenpA as is likely (D’Archivio and Wickstead 2017). One might suppose that inner KPs evolved from early neomuran ParB proteins (in *Sulfolobus* some have a CenpA-like domain as noted above) and their interactors in such a way that binding ability for ParA was lost but binding to ParS DNA modulated by CenpA retained—but no trace of a ParB-relationship is seen in modern kinetochores (Tromer et al. 2019). For a brief period, prokaryotic Min/ParA-based and eukaryotic kinetochore-based segregation might have functioned in parallel if ParS sequences duplicated, competed for ancestral ParB and derived inner-kinetochore binding, and eventually diverged, and the superseded ParA/MinD system was discarded. Crucial for perfecting kinetochore function was the origin before LECA of mt-depolymerising kinesins-8 and kinesins-13 (Walczak et al. 2013). Though both evolved before LECA, only one (?8) would have been necessary at first as yeasts secondarily lost 13; the other made quantitative improvements, both needed in many eukaryotes for establishing metaphase plate and anaphase A. We cannot be sure that ParA and MinD were lost during eukaryogenesis rather than repurposed; for example BLAST-P with an archaebacteria protein annotated as MinD as query against eukaryotes strongly hits cytosolic Fe-S cluster assembly factor NTPase NUBP1, and using eukaryotic NUBP1 as query against prokaryotes hit numerous ATPase/ParA annotations (all phyla; most top hits to Planctobacteria), implying that such P-loop ATPases are remarkably conserved despite likely different functions and that one cannot understand their evolution without more structural studies and experimental study of molecular function in many diverse taxa. The NUBP1/2 heterodimer of related P-loop NTPases is a negative regulator of ciliogenesis, is involved in centriole duplication, and interacts with the CCT/TRiC chaperone complex (Kypri et al. 2014) as well as mt-severing factors (Ververis et al. 2016), so it is multifunctional for centrosomal/mt functions as well as transferring Fe/S clusters. We suggest its core function may be not in Fe/S protein assembly but positioning other molecules to centrosomes or nuclei and that NUBP1 and 2 could have evolved by gene duplication of originally planctobacterial MinD (or ParA?) during eukaryogenesis.

Even though cytoplasmic dynein 1 has multiple functions in animal mitosis, it cannot be absolutely essential for mitosis or any other non-ciliary functions as it has been lost in Viridiplantae and in all species without any dynein (a few yeasts, piroplasms, unicellular red algae, and microsporidia (Kollmar 2016)—all have rather small cells and spindles—unlike Viridiplantae). Nonetheless, dynein 1 had evolved in or before LECA as did ciliary dyneins (IFT dynein 2 and eight axonemal dyneins) and their major intermediate and light chains that associate with the heavy chain tail and likely mediate cargo binding and motility regulation (Kollmar 2016). Dynein is especially suitable for mediating sliding of parallel mts and likely evolved for that function at or before the origin of anaphase A. The dynein heavy chain is an AAA^+^ ATPase belonging to a eukaryote-specific family that includes nucleolus-located midasin (Garbarino and Gibbons 2002) and its fungal homologue Rea1, a large motor ATPase vital for converting large subunit preribosomal RNA to an early preribosomal particle (Romes et al. 2016) and then for facilitating massive structural rearrangement of the pre-60S ribosome precursor enabling it to bind nuclear export factors (Barrio-Garcia et al. 2016), whose origin must have played a central role in nuclear origins. This phylogenetic connection between dyneins, the ciliary axonemal motors whose cytoplasmic relatives are involved in anaphase B, and two nucleolar motors required for ribosome biogenesis strongly supports two earlier ideas: (1) that nuclei, mitotic anaphase A, and cilia all evolved at essentially the same time and that their origin entailed coevolution between all three processes in the prekaryote stem lineage (Cavalier-Smith 1987c); and (2) evolutionary hyperacceleration in the eukaryote stem of ribosomal trees is most logically explained as coadaptive with major novelties in nucleolar assembly and trans-Npc export of eukaryotic ribosomal subunits, which is absent in prokaryotes (Cavalier-Smith 2002a). Involvement of dynein relatives never found in bacteria in two stages of nuclear large ribosomal subunit maturation (Shchepachev and Tollervey 2016) provides clear evidence of the molecular mechanisms underlying this example of intracellular molecular coevolution previously deduced from evolutionary logic (Cavalier-Smith 2002a, 2014).

In such concerted evolution, it is hard to order events that must have been partly in parallel, but we suggest dynein heavy chains originated first as a simple two-headed homodimer able to reversibly cross link parallel mts in premitotic half spindles. This would both have stabilised spindles and allowed kinetochore mts to slide during their shortening relative to interpole mts. If that was their only function intermediate and light chains of dynein might not have been necessary, making the origin of dynein/midasin motors easier to understand. Closest relatives of the dynein/midasin family are the MoxR family: chaperones in assembly of specific enzyme complexes ranging from methanol dehydrogenases through nitric oxide reductases, RuBisCo of eubacteria and archaebacteria, and even eubacterial gas vacuoles that mediate flotation (Iyer et al. 2004). The dynein/midasin common ancestor changed too much during eukaryogenesis to allow us to identify a specific MoxR subfamily from which it evolved, so does not help identify the eubacterial ancestor of eukaryotes. A chaperone function is retained in midasin/Rea1. Midasin mediates addition of the ribosome assembly factor Ytm1/WDR12 to early large subunit precursors, subsequently removing the early assembly factor Rsa4/Nle, and activating Nug2 GTPase to make an export-committed large ribosomal subunit. Ytm1 and Rsa4 are related propeller proteins with a ubiquitin-like (UBL) domain next to their WD40 repeats, emphasising the major contribution of propeller and ubiquitin domains to eukaryotic innovations. Midasin binds to UBL domains. By contrast dynein acquired mt-binding domains instead. Then as a generalised dynein heavy chain became diversified in function as ciliary axonemes started to evolve, it also became able to bind intermediate and light chains for regulating different functions. Intermediate light chains are related to Ras-like GTPases and more distantly to kinesins, emphasisng the expanded role of GTPases in eukaryotic regulation (Leipe et al. 2002). So both a P-loop GTPase-relative and an AAA+ ATPase were coopted to make dynein—this greater complexity in its origin is consistent with our argument that dynein likely evolved after kinesin and myosin.

## Planctobacterial origin of eukaryote cell cycle control

The central logic of the eukaryote cell cycle (Nasmyth 1995) is that cyclin proteins increase coordinately with growth and control timing first of DNA replication initiation and then anaphase though cyclin-dependent kinase (CDK) phosphorylation of numerous proteins. Anaphase is initiated by CDK-dependent activation of anaphase promoting complex (APC) triggering proteasome-mediated digestion of numerous ubiquitin-tagged proteins which resets the cell cycle for the next cell generation. This array of processes ensures balanced growth with conserved cell volume and coordination between growth, replication, and division by a fundamentally different machinery than prokaryotes use, which must have evolved in the eukaryote stem before LECA coordinately with the structural innovations outlined above, as Cavalier-Smith (2014) explained.

CDKs are serine/threonine (S/T) kinases, which are also involved in other eukaryote-specific control machinery (e.g. mitotic aurora kinases (Brown et al. 2004) or polo-like kinases that can amongst other things control mt nucleation by γ-tubulin (Gouveia et al. 2018)), but are relatively rare in prokaryotes. Even CDKs are not solely cycle controllers but affect other processes, e.g. COPII vesicle transport (Hu et al. 2008), transcription and RNA processing and have several paralogues in most eukaryotes (Tulin and Cross 2015). S/T kinases evolved in LUCA and are sparsely present throughout prokaryotes, not restricted to eukaryotes as once supposed (Krupa and Srinivasan 2005). However, they are really abundant only in Posibacteria (especially Actinobacteria, one reason they were once favoured as eukaryote ancestors: Cavalier-Smith 2002c), Planctobacteria, and the δ-proteobacterial genus *Myxococcus* (Arcas et al. 2013). Their prokaryotic functions are well studied only in posibacteria and include regulating murein synthesis and associated two-component control systems (Dworkin 2015; Libby et al. 2015; Rajagopalan et al. 2018). If murein regulation is also a function in Planctobacteria, loss of murein by a planctobacterial ancestor of neomura would have made them available for new functions including cell cycle regulation which had to change after histones evolved in stem neomura and after the origin of mitosis and nuclear division. On an ML tree, eukaryote and archaebacterial S/T kinases nest separately within the hugely diverse planctobacterial clade not within posibacteria or *Myxococcus* (Arcas et al. 2013), making Planctobacteria a more likely ancestor of both than are posibacteria, and inconsistent with archaebacteria being much older than or ancestral to eukaryotes.

S/T phosphatases are equally important in these regulatory processes and of similar phylogenetic distribution and likely also came from Planctobacteria by vertical inheritance. CDK cell cycle and transcriptional control occur across eukaryotes including in opisthokonts, which may have a distinct CDC paralogue subgroup (Krylov et al. 2003), Plantae, and Euglenozoa (Badjatia et al. 2016), so (however one roots the eukaryote tree) must have evolved in LECA. Cyclins share a domain with neomuran-specific transcription factor TFIIB so may have evolved from it after eukaryotes diverged from archaebacteria (Cavalier-Smith 2014).

## Planctobacterial origin of neomuran ubiquitin system

It was also once thought that ubiquitin (Ub) was restricted to eukaryotes, but Ub and ubiquitylation involving a cascade of E1, E2, and E3 enzymes is now well established in the thaumarchaeote *Candidatus* Caldiarchaeum subterraneum and related enzymes are known in Asgards (Fuchs et al. 2018), so ubiquitylation was likely present in the last common ancestor of Filarchaeota. E2 homologues have now been discovered in Planctomycetes and in Cyanobacteria, both posibacteria phyla, and δ-proteobacterial genus *Myxococcus.* Arcas et al. (2013) speculated that all prokaryote E2 came by LGTs from eukaryotes, but their Bayesian tree did not support that, showing a single > 90% supported bipartition between prokaryotes and eukaryotes. At present, there is no evidence for eubacterial Ub.

Prokaryotes generally use a ubiquitin-like protein (Ubl) with similar β-grasp domains (Iyer et al. 2006) and an E1-like Ubl-activating enzyme for sulphur transfer to various molecules (Maupin-Furlow 2013b); Ubl likely diverged from an RNA-binding protein before LUCA (Iyer et al. 2006). JAB peptidase components of the proteasomal lid that remove Ub before degradation have eubacterial homologues (Iyer et al. 2006), but the enzymes involved in tagging proteins by the Ubl Pup (Delley et al. 2017) of actinobacteria (pupylation: Pearce et al. 2008) are not E1 or E2 homologues (Becker and Darwin 2017). Planctobacteria, the more ancient Armatimonadetes, some Proteobacteria, and one archaebacterium, have a Ubl protein differing from Pup, so it will be important to see if it mediates a more eukaryote-like tagging mechanism or is yet another prokaryotic variant of tagging machinery likely to extend back to LUCA. Archaebacteria additionally use Ubl tagging (sampylation) both for S-transfer and for identifying metabolic proteins for proteolysis by proteasomes, best studied in euryarchaeotes (Fu et al. 2016, 2018; Hepowit et al. 2016; Maupin-Furlow 2013a). Involvement of Cdc48 type AAA+ ATPases in halobacterial sampylation for proteasomal targeting (Fu et al. 2016) as in eukaryotic ERAD proteolytic digestion show that link had already evolved at least as early as the neomuran stem, consistent with ERAD-like proteolysis perhaps having preceded phagotrophy as a way of feeding on external proteins (Cavalier-Smith 2009). To clarify this, planctobacterial mechnisms need intensive study.

We suggest that ancestral neomura inherited E1 and E2 enzymes vertically from planctobacterial ancestors; a linkage between Ub/Ubl and proteasomal proteolyis was either already present in planctobacteria or evolved in ancestral neomura at which time E3 probably arose. Ub and Ubl systems still coexist in eukaryotes and some TACK/Asgard Filarchaeota but Ub, E2, and E3 appear to have been lost in euryarchaeotes and likely Sulfolobia (crenarchaeotes s. s.). In our view, Ub and E3 are further examples to be added to core histones, more complex SRPs, drastically modified replication enzymes, Mcms, and actin that were innovations in the neomuran stem rather than in the eukaryote stem only as originally thought (Cavalier-Smith 1987c). Further study of asgards may reveal other characters of stem neomuran rather than prekaryote origin, but if such exist they should not be misinterpreted (as these other neomuran characters have been) as favouring a direct origin of eukaryotes from archaebacteria, as the frequency of differential character loss amongst archaebacterial lineages was so great (this cannot be denied for ancestrally eubacterial characters like FtsZ or MreB). Presence versus absence can be a hazardous character for phylogenetic inference, as the frequency of loss is generally hugely underappreciated.

## RP parsimony rooting the universal tree

In a more critical discussion than most of the universal tree, Forterre (2015) argued that early parsimony rooting suggesting a root in the neomuran stem is completely unreliable because of LBA artefacts and loss of information in the long stems of many proteins, though he was unaware of the existence of paralogue trees with less bias that root the tree within eubacteria as stressed by Cavalier-Smith (2002a, 2006a, b). Instead, Forterre argued that the tree can be better rooted by parsimony arguments about gain and loss of the 102 RP families, only 34 being found in all three domains (Lecompte et al. 2002) of which two are absent in some eubacteria. As neomura share 33 proteins uniquely with each other but neither eukaryotes nor archaebacteria do uniquely with eubacteria, he sensibly argued that eubacteria having many fewer RPs most likely represent the ancestral state, making neomura a derived state and a clade; we agree that the 33 novel proteins are shared derived characters that arose in the neomuran revolution by replacement (or radical transformation beyond recognition) of the 23 RPs unique to eubacteria. Oddly unaware that that clade has been called neomura for 32 years (Cavalier-Smith 1987c), Forterre (2015) adopted a pointless new name! More seriously unaware of the strong arguments for the root being within the eubacterial crown, Forterre placed it within the neomuran stem, mistakenly calling it the ‘bacterial branch’. Rooting in that stem is a fallacy because his parsimony argument cannot distinguish between a root in the neomuran stem or eubacterial crown. It shows that neomura are indeed a clade, thus firmly excludes the root from the neomuran crown and (in combination with fossil evidence for eukaryotes being immensely younger than eubacteria) confirms that eubacteria are ancestral to them as Van Valen and Maiorana (1980) and Cavalier-Smith (1981) first argued. But RP parsimony alone cannot localise LUCA to a specific part of the eubacterial tree.

Because almost all eubacterial phyla have nearly the same number of RPs (65–67), we could put the root anywhere within crown eubacteria without significantly altering the number of losses and gains on the overall tree compared with rooting in the neomuran stem, so RP gain/loss parsimony does not put it specifically in the neomuran stem. By contrast, if the root were anywhere within neomura, e.g. at the base of or within archaebacteria, one would have to invoke 33 losses of the uniquely neomuran RPs, 10 more than with a eubacterial root. In addition, an imaginary transition from neomura to eubacteria would (a) require all retained RPs to undergo numerous deletions to shorten and simplify them and (b) complicate the origin of RPs in LUCA with 67, not just 57 new RPs as with a eubacterial root. Thus, RP parsimony decisively excludes the root from neomura but not from within eubacteria, contrary to Forterre’s assumption that might have been unconsciously driven by illogical ‘folkloric’ carry over from unreliable 1989 neomuran stem paralogue rooting that he rightly rejects. Fashion not evidence or logic drives most assumptions of this root position. It was simpler for life to begin with fewer and shorter RPs than with many more longer ones. Eukaryotes evolved 11 cytosolic RPs absent from prokaryotes.

Irrespective of where we place the root, we have to accept rare RP losses within eubacteria, more substantial losses within archaebacteria (nearly all lineages have lost 1-11 RPs), and very rare losses in eukaryotes (largely restricted to 4 RPs in microsporidia). Most eubacterial phyla have some members that have lost one or two RPs (especially S1, S21, L25, L30) (Lecompte et al. 2002). Only Planctobacteria appear to have lost L30 altogether. Though RP parsimony is a strong argument against putting the root within clade neomura and for neomura being derived, RPs alone do not decisively exclude the root from the neomuran stem, though one can argue that if it were in that stem the dramatic change from a eubacterial to neomuran state (the most complex change to ribosomes since life began) would have had to have happened *almost immediately* after the origin of life and the first 57 RPs: in effect, one would be imagining that the two most difficult ribosomal innovations in the history of life (origin of ribosomes and of neomura) took place almost simultaneously. In our view, that and the numerous other changes needed to explain the eubacterial/neomuran transition would make the early origin of life much harder to understand if they had to happen immediately after life began. We can exclude the root from the neomuran stem indirectly by using the fossil record to show that eukaryotes are billions of years younger than eubacteria, so neomura must be derived from them far later than assigning LUCA to the neomuran stem implies (Cavalier-Smith 1987, 2002a, 2006a, b). Thus, the origins of life and of neomura cannot have been near simultaneous as so many, e.g. Martin and Russell (2003) who still cling to the disproven progenote idea, implicitly assume. Much discussion above makes a late eubacterial to neomuran transition cell biologically more comprehensible, as does our mapping sequence trees critically onto the fossil record. Nobody has ever justified neomuran stem rooting in comparable detail or explained neomuran origin as thoroughly.

Forterre, like most other molecular biologists, has persistently ignored that key fossil evidence as well as arguments from over 20 independent character transition analyses that clearly established the eubacterial ancestry of neomura (Cavalier-Smith 2002a). The only palaeontological data Forterre (2015) cited, 2.1 Ga large pyrite concretions claimed to represent multicellular ‘fossils’ (El Albani et al. 2010), he calls ‘possible multicellular eukaryotes’ in an effort to persuade readers that eukaryotes are almost three times as old as more reliable evidence discussed above indicates. But these structures do not resemble multicellular eukaryotes in any way! Though some palaeontologists consider them bacterial (Donoghue and Antcliffe 2010), we agree with distinguished palaeontologist Seilacher (Nature doi:10.1038/news.2010.323) that they are probably pseudo-fossils—‘aggregations of the mineral pyrite that grew in different shapes depending on the changing state of the surrounding sediment’ saying nothing about biology. Were they eukaryotes they would be totally discordant with all other evidence, yet still ~ 1.3 Ga years too young to give any credence to the fallacious rooting of LUCA within the neomuran stem.

Forterre’s review is superior to most in realising that deep branching in rDNA and paralogue trees is mostly artefactual and that comparative biochemistry can be at least as informative as sequence trees about evolutionary pathways. To supplement his RP rooting, he considers the biosynthesis pathway of N^6^-threonylcarbamoyl (TC) adenosine found in all tRNAs decoding codons that start with adenosine, which helps codon-anticodon pairing and stops frameshifting. To make its precursor TC-AMP, all life uses the same enzyme family (TsaC/Sua5) that evolved before LUCA, but to transfer TC to tRNA eubacteria use TsaBDE whereas neomura use a ‘KEOPS’ complex of four proteins (five in fungi). The simplest interpretation is that TsaBDE was the ancestral mode of transfer in LUCA, but stem neomura replaced it by the more complex KEOPS, whose Kae1 protein is related to TsaD. Mitochondria have a simpler system where a TsaD orthologue Qri7p can do the transfer on its own (Thiaville et al. 2014). We therefore suggest that changeover from eubacterial TsaBDE could have been achieved via an intermediate stem neomuran that lost ancestral TsaBE proteins analogously to mitochondria, replacing their function by the other three KEOPS proteins. That is mechanistically and phylogenetically plausible. It does not require that LUCA was in the neomuran stem as Forterre (2015) wrongly assumed. He rightly argued that putting LUCA within neomura is less parsimonious as it implies that the more complex KEOPS came first and was replaced by simpler TsaBDE in eubacteria. But he did not even consider putting the root within eubacteria, which overall gives the simplest of all possible interpretations as (unlike his) it fits evidence for eubacteria being immensely older than neomura. He assumed that LUCA had only TsaC/D and that eubacteria added TsaB/E and neomura the other three KEOPS proteins independently.

Considered entirely on its own, his idea is marginally simpler than our interpretation in that it avoids the loss of TsaBE we infer in the neomuran stem. But postulating this loss is entirely reasonable given that mitochondria prove that precisely such loss is mechanistically possible and actually happened in stem eukaryotes. Avoiding postulating two instead of one TsaBE losses is a very weak argument for putting LUCA in the neomuran stem when so much far stronger evidence argues against it. This emphasises that rooting arguments by comparative biochemistry must not use just one isolated snippet of information but the totality of evidence and must be reconciled with palaeontology that directly gives evidence of relative and absolute timing of historical events. As in Forterre’s scenario, we suggest that when threonylcarbamoyl (TC) adenosine first evolved in stem eubacteria (before Chloroflexi and other eubacteria diverged), they probably needed only a single transfer protein, but that would have been a TsaD version not a hypothetical intermediate between TsaD and Kae1 that only arose billions of years later.

## Neomuran revolution increase in SRP and ribosome complexity

Applying parsimony to the SRP, effectively the third ribosomal subunit necessary for cotranslational protein insertion into membranes, shows a clearcut increase in complexity during the neomuran revolution, previously proposed as a coevolutionary trigger for associated ribosomal changes (Cavalier-Smith 2002a): eubacterial SRP RNA is usually shorter (4.5S) than neomuran 7S SRP RNA, lacking translation arrest helix 6 that binds novel neomuran protein SRP19. Eubacterial SRP RNA has only one protein (Ffh modified to neomuran SRP54); ancestral neomura added SRP19 and eukaryotes evolved four more. Prokaryote SRP receptor (SR) is single peripheral membrane protein, GTPase FtsY, which eukaryotes anchored more firmly to the ER membrane by evolving switch GTPase SRβ, an unrelated novel integral membrane protein. We propose that SRβ was the ancestor of related switch GTPases, Arf and Sar1, recruited to control CopI and CopII coated vesicles respectively during early eukaryogenesis (Jékely 2004).

Extra neomuran SRP complexity is associated with loss of eubacterial SecA ATPase and SecB chaperone used for posttranslational unfolded protein insertion (neomura kept TAT machinery for folded protein translocation). As SecA and ribosomal binding sites overlap on the shared SecY protein-conducting channel across the CM (Knyazev et al. 2018), SecA loss would likely have had coevolutionary side effects on ribosomes as their structure would no longer be constrained by competitive binding of SecA to SecY. Purely cotranslational unfolded protein translocation and CM insertion could thereafter be optimised without ancestral constraints posed by having partially to share SecY binding sites between SecA and ribosomes. This gives the first specific molecular justification for the thesis that the stem neomuran switch from partially posttranslational insertion into SecY to cotranslational insertion only was likely the major coevolutionary explanation of the neomuran revolution in ribosome structure that explains the long neomuran stems on rRNA and RP trees (Cavalier-Smith 2002a). We argue that SecA/B loss was made easier by losing the planctobacterial OM, as OM proteins cross the CM posttranslationally whereas proteins in the retained CM are mostly inserted cotranslationally by SRPs. Adding a translation arrest domain would slow translation till after SRP docking on SR and avoid wasteful production of unfolded, envelope proteins in the cytosol that SecA/B could no longer insert. Thus modifying ribosome binding both to SRP and to the SecY closable channel substantially changed stem neomuran ribosomes.

The neomuran revolution of RP composition is more comprehensible if it was an immediate coevolutionary response to destabilisation of nascent protein secretion by simultaneous loss of both murein and OM by a planctobacterium, as we now argue, than when we thought neomura evolved from posibacteria that had lost the OM long before. In endobacteria, when Clostridia/Bacilliia lost the OM, they independently evolved a similar translation arrest domain but without SRP19 (Rosenblad et al. 2009), we argue for similar reasons. So also did Thermotogales, possibly because their looser radically modified OM had repercussions on protein secretion rates. As Actinobacteria lost the OM without lengthening SRP RNA (Rosenblad et al. 2009), this is not the only possible response to OM loss, but its happening in two independent groups means that SRP expansion by a new arrest domain was almost certainly an adaptive response to OM loss in both Clostridia/Bacilliia and stem neomura. But ribosomal changes in non-neomuran cases of OM loss were less radical because SecA was kept, so competitive binding to SecY continued to constrain ribosomal structure to the eubacterial pattern.

From a parsimony perspective, it is unambiguously simpler to suppose that the eubacterial version of cotranslation secretion by SRP was the ancestral form in LUCA, and that it later directly gave rise to the more complex neomuran one and then the very complex eukaryote one that is most derived. But we agree with Forterre (2013) that neither the neomuran nor the eukaryote ancestor was an archaeon, and that archaebacteria underwent thermal streamlining when their ancestor evolved hyperthermophily via novel lipids and sublineages underwent extensive differential gene loss, unlike their eukaryote sisters. From a likelihood perspective, it would have been easier for LUCA to have evolved the simpler eubacterial system than the more complex neomuran one. Ffh and FtsY share two major domains and evolved by gene duplication preLUCA from an ancestral GTPase with both and addition of a third domain at opposite ends to mediate SRP-RNA and membrane attachment respectively. If the planctobacterium that generated neomura had polar flagella, they were lost together with FlhF GTPase that posttranslationally directs flagellar assembly to cell poles (as far back as Chloroflexi) and shares their two core domains with Ffh and FtsY (Bange et al. 2007). All three arose preLUCA as the only members of the SRP GTPase family. The unique N-terminal domain of FlhF presumably was added to bind the flagellar export machinery.

## Mechanisms of stem neomuran RP replacement after planctobacterial OM loss

This is not nearly as difficult as many assume since many eubacterial RP genes can be experimentally deleted individually without obviously harming cell reproduction (Dabbs 1986). Therefore, when OM loss destabilised ribosome/membrane interactions, non-universal RPs could have been lost one at a time and replaced by similar unrelated ones that could fit into the same cracks in rRNA structure. Losing all 23 eubacteria-specific RPs simultaneously would probably have been too harmful to allow survival but losing one or two at the same time must have been tolerable. When enough were lost to slightly slow growth, selection would favour their replacement by a neomuran equivalent to restabilise the ribosome.

Figure 17 of Klein et al. (2004) shows six examples comparing eubacterial and archaebacterial ribosomal regions in 3D where replacement RPs fill the same cracks between RNA helices of the large subunit. Replacements had to be small basic proteins of more or less the same overall size and shape as the originals but need not have had (and mostly did not have) the same secondary structure; it is much easier for a molecule that is essentially an adhesive filler to be replaced by a structurally non-homologous one than it is for catalysts. At least one replacement (L44e) appears to have evolved from a smaller eubacterial ancestor (L33) by circular permutation of its globular ‘filler’ domain and insertion of a long non-globular intermediate domain that cannot fit into the original slot on the ribosome but appears to do no harm. This restructuring to make a non-orthologue could have occurred at the DNA level by an inversion and an insertion, so this protein is not entirely new to the ribosome. Possibly some other neomuran ‘replacement RPs’ might be similarly drastic reconstructions of eubacterial proteins, but most are probably separately recruited proteins that happened to fill the gap, and thus truly convergent. That loss and later replacement was the main mechanism is also suggested by the fact that L36 was lost and not replaced, so a gap remains in archaebacterial ribosomes even though its potential binding sites are conserved. Conversely at least one novel neomuran protein L18e fills in and stabilised a gap that was never filled in the ancestral ribosome, and another (L19e) fills a different part of the same large cleft from the original H59. These non-equivalences mean that replacement probably did not perfect each region independently. What would have mattered was overall stability of each ribosomal subunit and their continued ability to bind each other, SRPs, mRNA, tRNAs, and protein cofactors, not the precise structure of each part.

Given an initially strongly destabilising force, stabilisation would come about by a succession of partially random selections of replacements ranging from very similar to radically different, but any that happened to stabilise overall performance would be selected. RPs with a key early role in assembling modern ribosomes were not replaced but kept by all three domains. Replacements largely affected less crucial stabilising gap fillers and must have been completed relatively quickly in stem neomura before archaebacteria and eukaryotes diverged. Replacements and adjustment to rRNAs must have secondarily accelerated changes (both amino acid substitutions and insertions) to the universally conserved RPs, accounting for the immensely long neomuran stems on both RP and rRNA trees, misinterpreted as ancient by Woese and others, many of whom mistakenly assumed they represented long-drawn-out slow change rather than rapid short-lived results of sudden secondary destabilisation. Once the neomuran replacements and rapidly following stem eukaryotic RP innovations had settled down and could undergo no further major improvements, stabilising selection prevented such major change in most neomura, except when massive genome reduction, notably in microsporidia and DPANNs, caused further destabilisation—though less radical than those caused by OM loss, origin of nuclei and initial complexification of ER SRPs.

Such a piecemeal neomuran RP changeover is mechanistically much more plausible than assuming that LUCA had only the 34 universal RPs and eubacteria evolved 23 and neomura 33 unique RPs simultaneously as each diverged from an imaginary ancestor positioned on the neomuran stem as Klein et al. (2004) and Forterre (2015) speculated. More likely, the first ribosomes had much smaller rRNAs with stabilising cations but no RPs, the large subunit being a small self-folding peptidyl transferase core and as soon as translation and the genetic code evolved crudely, extra RNA helices and associated RPs were added simultaneously to produce the complete cenancestral eubacterial ribosome (Lanier et al. 2017; Petrov et al. 2015). That accretionary expansion to a full eubacterial ribosome with 57 RPs involved coevolution of rRNA helices and RPs and must have been complete before LUCA. Broad principles of the accretionary model are probably correct, but its phylogenetic perspective is oversimplified in ignoring secondary reductions in ribosome size as in microsporidia and many archaebacterial lineages and also in misrooting the overall tree beside rather than within eubacteria (Petrov et al. 2014). We doubt that a 34-RP ribosome with full length rRNAs implied by Klein et al. (2004) and Forterre (2015) would be stable and it seems unlikely that two diverging sisters would suddenly have added a total of 56 new RPs. That idea is based solely on misrooting the universal tree in the neomuran stem coupled with devout adherence to the strong but unjustifiable prejudice of Woese (2000) that ‘modern cells are sufficiently complex, integrated and “individualized” that further major change in their designs does not appear possible’ which Forterre quoted with excessive approval. Contrary to that prejudice, destabilising losses, e.g. of OM, murein, and SecA, albeit rarely, can allow radical changes.

Woese and Forterre were probably right in supposing that eukaryotes did not evolve from archaebacteria but wrong in rejecting the possibility that their common neomuran ancestor evolved directly from a highly developed eubacterium. Neither Woese nor his followers like Forterre, Martin or Koonin, still trapped in the mistaken prejudice that direct transition from eubacterium to archaebacterium is impossible, ever credibly explained why anyone should believe that unreasonable and intellectually restrictive dogma or engaged with the strong phylogenetic cum palaeontological and cell biological evidence that arguably refuted it decades ago. Our piecemeal model shows how a relatively late neomuran revolution was possible for RPs. Foregoing sections explain how billions of years after LUCA all other major innovatory cell characters of stem neomura could have arisen in a concerted way and also how simultaneous conversion rapidly thereafter of an early neomuran prokaryote into a eukaryote and an archaebacterium could have occurred phylogenetically, selectively, and mechanistically.

We hope readers will be sufficiently stimulated to question Woesean unwillingness even to think how transitions between domains are possible. If our proposals for a planctobacterial origin of neomura and sisterhood of archaebacteria and eukaryotes seem deficient in some respects, please draw attention to the biggest problems and try to overcome them constructively. Alternative proposals should be in comparable detail to allow adequate assessment and criticism.

## Impossibility of a eukaryote to prokaryote transition

Mariscal and Doolittle (2015) misleadingly call ‘eukaryote-first’ any ideas about LUCA that imagine that it had a few supposedly ‘eukaryotelike’ characters. However, of the 60 major eukaryote characters listed by Cavalier-Smith (2009), it appears that nobody has seriously suggested that even one was present in LUCA, though Bisset (1963) in a cursory line suggested that bacteria might have lost the nucleus (giving no reason or mechanism). Mariscal and Doolittle (2015) considered only one of the 60 characters (spliceosomes) and argued that converting them to prokaryotic group II introns would be ‘nearly impossible’. We agree, but a high proportion of the rest would also be ‘nearly impossible’ to reverse. No genuinely eukaryote-first theory has ever been proposed or could ever be compatible with already known facts. It would have been impossible for eukaryotes to have evolved first and all eubacteria to have evolved from their mitochondria that escaped to became free living to make the first α-proteobacterium and from it all other eubacteria (or all prokaryotes). Eukaryotes-first would make the origin of life immensely harder to understand; nobody has ever suggested any way of making a nucleus with pore complexes, Golgi and mitosis, and cilia directly from precellular life without a prokaryote intermediate.

Mariscal and Doolittle (2015) are right that it is not irrational or illogical to question the idea that LUCA might have been more similar to eukaryotes in some unspecified way than are prokaryotes. But without specifying details, that idea is empty and unhelpful. They also fail to appreciate that all evidence decisively rejects the possibility that a real eukaryote with these 60 characters (most never figure in their thinking and were unknown when Bisset wrote) could ever have been converted into a prokaryote. For a major fraction of such characters, conversion would also be ‘nearly impossible’. The only way to make prokaryotic membrane topology and DNA- and SRP-receptor attachment to the CM (major features of prokaryoteness (Cavalier-Smith 2007b) that their naively Pacean perspective overlooks) from a eukaryote ancestor would be by nuclear envelope fusion with the plasma membrane and complete elimination of nuclear pore complexes, coated vesicle budding and targeting. Even were that mechanistically possible (which we strongly doubt), it would almost certainly kill the cell; any soluble proteins in the endomembrane lumen would immediately be lost to the cell and ribosomes would immediately start translating unspliced premessengers to make harmful non-functional proteins.

Not only would all 60 characters have to be lost or reversed entailing hundreds of dedifferentiations of related genes (that actually arose by gene duplication) back to single versions (and many hundreds of protein domain losses) but there are many more positive prokaryote characters than Mariscal and Doolittle (2015) recognise in their essay, that would in practice make convergence between eubacteria and archaebacteria, which they unwisely seriously entertain, quite impossible. Their discussion (using their own words) is ‘too coarse grained’ to be useful. How ever could mitosis in all its complexity evolve into DNA segregation by the ultrasimple prokaryote-wide ParAB diffusion-reaction mechanism? ParAB needs only two homodimeric DNA-binding proteins and no cytoskeleton for its core functions (though most lineages have ancillary polar-scaffolding proteins: Lin et al. 2017); in contrast, mitosis needs hundreds of proteins, many having undergone repeated gene duplications (represented only once in prokaryotes) and cannot have arisen in the first cell. There would be scores of ‘near impossibilities’ in any truly eukaryote-first scenario. Though one can imagine such things verbally, they are so highly improbable that nobody deeply familiar with either cell biology or palaeontology could regard them as anything but distracting absurdities. Logically, with complete knowledge, we could enumerate a series of mutations that could have converted an elephant into *Escherichia coli* (*E. coli*) or a giant sequoia tree or a boa constrictor into *Entamoeba coli* (another *E. coli*), but just because we can imagine a possibility, it does not become a scientifically acceptable hypothesis. Such conversions are so unlikely that even in theory it would be ridiculous to suggest that any happened. For them, however, we have palaeontological evidence that decisively tells us that tetrapods and trees are so much younger than either protozoa or bacteria that such physically possible (but exceedingly improbable) conversions were temporally impossible; even with no DNA sequence information, palaeontology coupled with comparative anatomy can tell us that many logically imaginable phylogenies are temporally impossible—one such is eukaryotes first.

No physical law prevents destruction of a city by bombing from being spontaneously reversible in principle but it is so highly improbable that entropy means it never happens. That must also be true of making a prokaryote from a eukaryote—one cannot undo large numbers of gene duplications and functional divergence or deletions even though point mutations are easily reversible. Moreover, critically interpreted, palaeontology proves that neither animals nor plants nor any other real eukaryotes could have preceded prokaryotes as they are billions of years younger—a bigger time difference by far than between humans and amoebae. Transition analysis (Cavalier-Smith 2006d) can polarise change because as Dollo (1893) pointed out evolution of any complex character is practically irreversible and one can work out which evolutionary direction is more likely (converting an ungulate into a whale or a dinosaur into a bird or lizard into a snake is inherently easier than the reverse; see also the argument why eubacterial flagella must have evolved in a negibacterium, not a monoderm: Cavalier-Smith 2006c), which sequence trees based on easily reversed substitutions only cannot tell us. Any truly eukaryote-first scenario is physically impossible. We have shown how the reverse transition could have happened.

## A large planctobacterium with actin-like skeleton engulfs and digests prey cells

After proof correction, an extremely important paper appeared describing a novel predatory ancient relative of anammox Planctobacteria (*Candidatus* Uab amorphum) (Shiratori et al. 2019), which greatly strengthens our conclusion that neomura arose from Planctobacteria. Uab's cellular properties are unique for prokaryotes and crucial to many aspects of the planctobacterial origin of eukaryotes and archaebacteria advocated here. Cell engulfment by Uab's large (>4-5 μm) soft, quasi-amoeboid, highly mobile cells involves invagination and budding of both CM and OM and is thus not strictly homologous to eukaryotic phagocytosis. However it provides the first incontrovertible example of a prokaryote able to engulf and digest prey cells and proof that this ability must have evolved in a planctobacterium in the absence of a mitochondrion (contrary to Lane and Martin 2010; Uab uses CM-linked surface F-ATPase to power engulfment, so decisively disproves the fallacious speculation that evolution of phagocytosis by such a prokaryote would necessarily digest its ATP-sythesis machinery and prevent phagocytosis evolving before mitochondria: Martin et al. 2017). Ultrastructural presence of a complex fibrous endoskeleton is unique for prokaryotes, exemplifying the thesis (Stanier 1970; Cavalier-Smith 1975, 1987c) that evolution of phagotrophy would lead to a more complex cytoskeleton and genome; Uab's actin-like protein similar to those of lokiarchaeotes is consistent with our thesis that actin evolved in a planctobacterium from a MamK-like ancestor before archaebacteria and eukaryotes arose and played a key role in the origin of phagocytosis. Uab makes it possible that a proto-phagocytic mechanism evolved in a similarly flexible peptidoglycan-poor planctobacterium prior to loss of the OM and complete loss of all peptidoglycan discussed here; it corroborates our thesis that rigidity of most other bacterial walls is what prevents phagocytosis and symbiogenesis.

In principle the double membrane 'phagosome' of the Uab lineage could have been converted into a true eukaryotic phagosome by OM loss and need not be convergent with phagocytosis. Though some digestive vacuoles may be topologically separate, they often remain attached to the cell surface by a narrow duct, so Uab's digestive system resembles pomacytosis more than standard phagocytosis, confirming our thesis that such topologically continuous intermediates are plausible eukaryogenesis precursors, and suggesting that Uab may not need membrane-fusion-based membrane recycling machinery akin to exocytosis. Uab apparently has ribosomes on its CM invaginations like rough ER and its large genome of 5660 genes mostly of unknown function makes it a much more suitable model for a eukaryote ancestor than are genically impoverished and genomically reduced archaebacteria. Intensive study of the large Uab clade, which the 171-protein tree implies diverged early enough from other planctobacteria to be ancestral to neomura, is vital - both to study the molecular basis of its unique shape-changes, locomotion, and prey uptake and to see whether Uab-like engulfing ability is old enough to have been ancestral to eukaryotic phagocytosis and discover if other characters previously considered unique to neomura have already evolved in these unique planctomycetes, thereby making planctobacterial origins of eukaryotes and archaebacteria even simpler than we argued. Is Uab really a descendant of a previously missing link between bacteria and eukaryotes or a remarkable but illuminating recent convergence?

## 26 major conclusions

1. Site-heterogeneous 51-RP trees for 143 eukaryotes show essentially the same clades as more gene-rich 187-protein trees but statistical support for the deepest nodes is substantially lower. Even with only 26 RPs, the main clades are essentially the same, but some have lower support and there are more minor inconsistencies (weakly supported) than with 187-protein trees.
2. Site-heterogeneous 26-RP trees for 143 eubacteria including all formally named phyla show 14 robust clades that merit recognition as phyla (under half the number previously recognised), several comprising more than one solely-rDNA-based ‘phylum’, whose constituent clades are better treated as subphyla or classes to simplify eubacterial classification. Relative branching order of the 14 robust phyla is much more strongly supported than in site-homogeneous analyses, and significantly doubtful only for the relative order of Hadobacteria and Fusobacteria, which might really be a single clade rather than successive branches.
3. Eubacteria-only trees strongly support the broad interpretation of Proteobacteria, including subphyla Rhodobacteria, Acidobacteria, and Geobacteria (Cavalier-Smith 2002a), which is a robust clade, whereas Proteobacteria sensu Woese that excludes various minor rDNA-defined phyla is paraphyletic (as on other published multiprotein trees).
4. Proteobacteria are sister to a robust clade comprising Planctobacteria, Sphingobacteria (sister phyla, both revised here by including insufficiently-distinct splinter ‘rDNA phyla’; jointly called Planctochlora), and Spirochaetae. This 4-phylum clade (infrakingdom Gracilicutes) is consistently supported by CAT and ML trees.
5. Thermophilic and hyperthermophilic eubacteria group in two distinct well-supported phyla: Synthermota (Thermotogia, Synergistia and relatives) and Aquithermota (Aquificia, Thermodesulfobacteriia). Aquithermota are sister to Gracilicutes.
6. Eubacteria-only CAT trees place Fusobacteria, Hadobacteria, and Synthermota successively more deeply than Aquithermota/Gracilicutes, these five groups collectively forming major clade Neonegibacteria.
7. Robust phylum Endobacteria (‘Firmicutes’ + ‘Mollicutes’), ancestrally negibacteria with endospores, includes lineages that independently lost the OM (at least twice, most likely four or five times), so endobacterial posibacteria are polyphyletic. Mollicutes are also polyphyletic, having lost murein walls at least twice.
8. Actinobacteria (ancestrally unimembranous posibacteria) are not sisters of Endobacteria, but probably sisters of Endobacteria plus Neonegibacteria, this joint grouping being sister to the robust Cyanobacteria/Melainabacteria clade (new superphylum Oxybacteria), all the foregoing being sister to Armatimonadetes, the most deeply divergent negibacterial phylum with OM lipopolysaccharide (LPS), to which we provisionally assign ‘Eremiobacteria’ a novel type of anoxygenic photosynthesiser.
9. Most Chloroflexi have an OM with no LPS and are not unimembranous posibacteria, though a few with thicker murein appear to have lost the OM convergently with posibacteria. The root of the eubacterial tree probably lies between Chloroflexi and all other organisms or (possibly) within Chloroflexi.
10. Ancestral eubacteria were probably negibacteria with an OM, which was lost about 6-8 times in evolution not just once as previously argued. OM loss and its regeneration from CM in every spore generation of negibacterial Endobacteria uniquely enabled multiple OM losses in Endobacteria, explaining why only Endobacteria had multiple losses. OM loss by murein hypertrophy occurred once in the history of life to create Actinobacteria before edospores evolved. A third mechanism—mutational loss of OM/CM cross bridges to simultaneously eliminate murein and OM in a planctobacterium—generated neomura.
11. Site-heterogeneous 51-RP trees for 60 archaebacteria place DPANN lineages within Euryarchaeota as two distinct lineages: ‘Nanohaloarchaea’, strongly sister to Halobacteriales making a halophilic clade, and ‘Microarchaea’ as sister to all euryarchea except Thermococcales. We argue that DPANN is not a natural group but a long-branch artefact resulting from two independent cell and genome miniaturisations by massive gene loss and accelerated sequence evolution. Therefore, the root of archaebacteria is probably between Euryarchaeota and Filarchaeota, the only subgroups to merit phylum rank.
12. RP evolutionary rates are more uniform within eubacteria than within archaebacteria or eukaryotes. Huge episodic accelerations in the stems of the neomuran and eukaryote subtrees are attributable respectively to coevolutionary consequences of changes in SRP and to the origin of transnuclear ribosome transport in eukaryotes which probably both occurred ~ 2.5 Gy after the origin of eubacteria. We give the first molecular explanation of how SecA loss in stem neomura probably caused coevolutionary changes in cotranslational protein insertion/secretion, resulting in transiently accelerated ribosomal evolution in the neomuran stem. These radical RP changes removed so many ancestral characters that most sites underwent numerous amino acid substitutions leaving hardly any extant information in RPs useful for inferring the positions of the roots of eukaryotes, archaebacteria, or neomura. That explains why rooting is so difficult and why using ribosomal sequences to establish from which eubacterial phylum neomura evolved is controversial.
13. The difficulty of rooting archaebacteria and eukaryotes and weak support for their basal branches is exacerbated by near simultaneous radiation of basal lineages in both domains and the sheer number of deep-branching eukaryote lineages, which reflect explosive radiations immediately after the origins of neomura, archaebacteria, and eukaryotes. Deep branches in the more uniformly evolving eubacteria are better spread out, making more characters available to allow robuster basal topology.
14. Our neomuran and three-domain trees all place the eukaryote root beside or within Eozoa, mostly between Percolozoa and all other eukaryotes. That is consistent with our earlier arguments that Eozoa are the paraphyletic ancestors of neokaryotes and with an old suggestion that Percolozoa are the most divergent mitochondriate eukaryotes, but loss of information in the eukaryote stem by hyperaccelerated substitutional overprinting is so great that the position of the eukaryote root remains an open question needing other evidence to settle it.
15. RP trees for eubacteria plus eukaryotes place eukaryotes within planctobacteria, whereas prokaryote trees contradictorily place archaebacteria as sisters of Planctobacteria/Sphingobacteria. Other evidence strongly supports the idea that neomura evolved directly from planctobacteria by the loss of murein and OM and the origin of core histones, more complex SRP, N-linked glycoproteins, ESCRT III, and actin, and that 13-pf eukaryote mts evolved from the more slender 4/5-pf planctobacterial mts.
16. Planctobacteria are atypical negibacteria with inflated periplasm and numerous characters preadapting them for simultaneous loss of OM/murein leading to archaebacterial origin and eukaryogenesis via the origin of mitosis and phagotrophy. Uniquely in prokaryotes, they have β-propeller/α-solenoid proteins essential for evolution of coated vesicle budding (and therefore the endomembrane system), nuclei and cilia.
17. Despite past serious misinterpretations of planctobacterial cell organisation, many key characters (e.g. mts, β-propeller/α-solenoid proteins, actin-like MamK, sterols) make planctobacteria superior to posibacteria or archaebacteria as direct ancestors of eukaryotes, for which our RP trees provide the first direct sequence support.
18. We explain in much more detail than hitherto how the eubacterial cytoskeleton was changed during the neomuran revolution and eukaryogenesis and how the cell cycle was simultaneously modified.
19. Because of the long eukaryote stem and very close basal radiation of archaebacteria RP trees cannot reliably place eukaryotes relative to archaebacteria. All neomuran trees place eukaryotes near the base of archaebacteria, disproving the idea that archaebacteria are several times older than eukaryotes. Though all place eukaryotes within and near the base of Filarchaeota, their exact position is contradictory; only some group them with asgards, others putting them in different places within TACK. Given these contradictions and great RP character loss in the eukaryote stem, all these positions could be wrong. In our view, RP trees cannot safely distingush between the ideas that eukaryotes are sisters of all archaebacteria, which for many other cell evolutionary reasons is most likely, or that they evolved from early filarchaeotes, which would be possible only if such filarchaeotes had retained almost all eubacterial characters that have been lost by all known archaebacteria. It is simpler to accept that the trifurcation Euryarchaeota/Filarchaeota/Eukaryota was too sudden following murein/OM loss for this star phylogeny to be accurately resolved. Critical transition analysis and intellectual reconstruction of likely evolutionary paths as adumbrated here more simply explains eukaryogenesis than would an origin from an early filarchaeote.
20. Archaebacteria were probably ancestrally facultatively aerobic respirers and likely evolved from facultatively aerobic Planctobacteria with enzymes and coenzymes for methylotrophy that were recruited for methanogenesis. Stem archaebacteria probably inherited prenyl diether lipids from planctobacteria, but ancestrally evolved novel tetraether monolayer membranes enabling them to become stabler hyperthermophiles, but lost planctobacterial sterols and acyl esters as their main membrane lipids, both retained by their eukaryote sisters. Their walls became more rigid, so they lost mts and failed to evolve phagotrophy and therefore did not change their cell cycles and structure as radically as eukaryotes, remaining osmotrophs like all prokaryotes.
21. Photosynthetic reaction centres and molybdenum-dependent nitrogenase almost certainly both evolved before LUCA. Comparison of their trees with our more robust RP trees shows that their genes were largely vertically inherited throughout prokaryotes and that multiple LGTs need not be invoked to explain their evolution. Vertical inheritance and multiple losses of photosynthesis and nitrogenase are sufficient explanation, except for photosynthesis LGT from Proteobacteria to *Gemmatimonas*. Critical reavaluation of the complex history of nitrogenase paralogues gives further evidence for eubacterial ancestry of archaebacteria.
22. Misrooting the universal tree in the neomuran stem through incorrect assumptions about stretched stems on ribosomal and protein paralogue trees grossly confused evolutionary cell biology leading to many incorrect conclusions, some of the more important ones being corrected here.
23. Prokaryote taxonomy based solely on rDNA divergence inflated the number of prokaryote ‘phyla’ by assuming that failure to group robustly with other clades is sufficient reason to make new phyla despite a low degree of divergence in important phenotypic characters. Most or even all ‘candidate phyla’ do not deserve such high rank and can be found sensible homes within established phyla. Robust site-heterogeneous RP multiprotein trees provide a sounder basis for a biologically superior and simpler taxonomy than less resolving 16S rDNA ML trees. We give many examples of such improvements at phylum, subphylum, and class rank and provide a complete 14-phylum higher classification for eubacteria. Such changes make it easier to see the wood for the trees.
24. ML trees are generally less strongly resolving than CAT trees and more easily perturbed when the extremely different sequences spanning two or three domains are added. Correct interpretation of two- and three-domain trees is much harder than often supposed; critical correlation with fossil evidence is essential to avoid being stuck in self-confirming arbitrary fashions without objective evidence as to root positions.
25. A eukaryote to prokaryote transition is physically imposssible, but the reverse is now comprehensible in considerable detail.
26. Crucial for spatial control of eukaryogenesis was replacement of prokaryotic mechanisms of DNA segregation and cell polarity based on ParAB and MinCD protein-diffusion ratchets by the origin of centrosome-polarised mts and stable bipolar spindle by evolving γ-TuSC mt-nucleation and bipolar kinesin-5. Together with cross-linked actin skeleton and coated-vesicle-based endomembrane system, this radically novel polarity mechanism effective over hundreds of micrometres enabled eukaryotic cells to grow much larger by overcoming the diffusion limits of prokaryotes.

### Electronic supplementary material

ESM 1 (PDF 1403 kb)

ESM 2 (PDF 132 kb)

ESM 3 (PDF 175 kb)

ESM 4 (PDF 161 kb)

ESM 5 (PDF 148 kb)

ESM 6 (PDF 114 kb)

ESM 7 (PDF 120 kb)

ESM 8 (PDF 141 kb)

ESM 9 (PDF 137 kb)

ESM 10 (PDF 119 kb)

ESM 11 (PDF 118 kb)

ESM 12 (PDF 128 kb)

ESM 13 (PDF 80.5 kb)

ESM 14 (PDF 124 kb)

ESM 15 (PDF 83.1 kb)

ESM 16 (PDF 114 kb)

ESM 17 (PDF 64.7 kb)

ESM 18 (PDF 75.1 kb)

ESM 19 (PDF 85.1 kb)

ESM 20 (PDF 75.7 kb)

ESM 21 (PDF 70.5 kb)

ESM 22 (PDF 116 kb)

ESM 23 (PDF 134 kb)

ESM 24 (PDF 107 kb)

ESM 25 (PDF 122 kb)

ESM 26 (PDF 91.5 kb)

ESM 27 (PDF 106 kb)

ESM 28 (PDF 84.2 kb)

ESM 29 (PDF 98.1 kb)

ESM 30 (PDF 111 kb)

ESM 31 (PDF kb)

ESM 32 (PDF 83.4 kb)

ESM 33 (PDF 103 kb)

ESM 34 (PDF 74.8 kb)

ESM 35 (PDF 57.6 kb)

ESM 36 (PDF 91.4 kb)

ESM 37 (PDF 64.3 kb)

ESM 38 (PDF 41.8 kb)

ESM 39 (PDF 56.5 kb)

ESM 40 (PDF 72.7 kb)

ESM 41 (PDF 212 kb)

ESM 42 (PDF 171 kb)

ESM 43 (PDF 109 kb)

ESM 44 (PDF 163 kb)

ESM 45 (PDF 148 kb)

ESM 46 (PDF 112 kb)

ESM 47 (PDF 149 kb)

ESM 48 (PDF 134 kb)

ESM 49 (PDF 134 kb)

ESM 50 (PDF 146 kb)

ESM 51 (PDF 150 kb)

ESM 52 (PDF 139 kb)

ESM 53 (PDF 1.16 mb)

## Taxonomic Appendix by T. Cavalier-Smith

I establish three new subkingdoms, an infrakingdom, three new phyla, revise circumscription of some others and make 21 new classes and 5 orders as well as a few new intermediate categories:

### New eubacterial taxa

**New subkingdom Chlorobacteria** Cavalier-Smith**. Description:** negibacteria with OM with no lipopolysaccharide or monoderm**;** green non-sulphur bacteria, e.g. *Chloroflexus* and their non-photosynthetic relatives. Murein layer and periplasmic space evenly thin in diderms, thicker in monoderms. Endospores absent. Photosynthesis anoxygenic by type II reaction centre and chlorosome antennae. Flagella rare, penetrate OM. Comprises all Chloroflexi. Originally a phylum restricted to the phototrophs (Cavalier-Smith 1992b). Type order Chloroflexales Gupta et al. 2013. **Etymology:***Chloro-* Gk yellowish green + bacteria.

**New subkingdom Eoglycobacteria** Cavalier-Smith**. Description:** photosynthetic or heterotrophic (respiratory or fermentative) negibacteria with OM with lipopolysaccharide and murein sacculi. Periplasmic space not inflated. Photosynthesis oxygenic by type I and II reaction centres and phycobilisome or chlorophyll *b* antennae. The clade comprising the last common ancestor of *Armatimonas* and *Anabaena* and all its non-chloroplast descendants. Largely mesophilic; thermophiles rare. Some have flagella, always penetrate OM. Endospores absent. Three phyla: photosynthetic Cyanobacteria, aerobic Armatimonadetes and fermentative Melainabacteria. Type order Armatimonadales Tamaki et al. 2011. **Etymology:***Eo-* Gk dawn; *Glucus* Gk sweet, based on Glycobacteria Cavalier-Smith 1998, 2002 to signify that they are the most ancient clade of bacteria with lipopolysaccharide (LPS).

**Subkingdom Posibacteria** Cavalier-Smith 1987**. Emended Description:** comprises Actinobacteria, ancestrally with no OM, and Endobacteria, ancestrally having an OM with LPS and endospores, but mostly consisting of monoderm lineages that lost OM. Murein if present (secondarily lost in some lineages of Bacillia) typically very thick, usually with teichoic acids and lipoproteins targeted by sortase(s). Largely non-photosynthetic heterotrophs; some chemoautotrophs or photoheterotrophs (type I reaction centre; do not fix carbon). Flagella lack the flange connecting to negibacterial OMs. A probably (not certainly) paraphyletic group ancestral (or, if Fig. 5 of Raymann et al. 2015 were correct, sister) to Neonegibacteria. Type order Bacillales Prévot 1953 (Approved Lists 1980). **Comment:** originally (1987) included only monoderms, but following Cavalier-Smith (2002a) now includes diderm endobacteria (Selenomonadia, Halanaerobiia) necessary to make the subkingdom monophyletic; but adding Thermotogales (Cavalier-Smith 2002a) or Chloroflexi (Cavalier-Smith 2014; Ruggiero et al. 2015) made it polyphyletic so both are here excluded. **Etymology:** Posi- root of positive to signify that most stain Gram-positively.

**New subkingdom Neonegibacteria** Cavalier-Smith**. Description:** photosynthetic or heterotrophic or chemoautotrophic negibacteria; OM always present, typically with LPS (sometimes lost); murein sacculus usually thin, sometimes locally thickened or in some planctobacteria secondarily lost except at septum. Periplasmic space can be inflated (Synthermota, Planctobacteria) or include flagella (Spirochaete). Comprises the last common ancestor of *Thermotoga* and *Escherichia* and all its eubacterial descendants. Photosynthesis anoxygenic by type I or II reaction centres, never both, with or without chlorosomes. Mesophilic, thermophilic, or hyperthermophilic. A paraphyletic group ancestral to Neomura; a clade on eubacteria-only trees. Type order Enterobacterales Adeolu et al. 2016. **Etymology:***Neo-* Gk new plus Negibacteria (Cavalier-Smith 1987) emphasises they are a later negibacterial group than Eoglycobacteria.

**New infrakingdom Thermobacteria** Cavalier-Smith**. Description:** non-photosynthetic heterotrophs or autotrophs; often thermophilic, sometimes hyperthermophilic; frequently have reverse DNA gyrase. LPS sometimes lost. External polar flagella common**.** Comprise 4 phyla: thermophilic or hyperthermophic Aquithermota; largely thermophilic Synthermota; partially thermophilic Hadobacteria; mesophilic or psychrophilic Fusobacteria, a minor phylum of anaerobic fermenters with only one order and 10 genera in two families. Paraphyletic ancestors of infrakingdom Gracilicutes. Type order Thermales Rainey and Da Costa 2002. **Etymology:** based on *Therm-* stem (Gk hot) of type genus of type order plus suffix bacteria to denote high rank.

**New phylum Aquithermota** Cavalier-Smith**. Description:** Gram-negative non-photosynthetic eubacteria with reverse DNA gyrase unlike most Gracilicutes; thermophilic or hyperthermophilic, anaerobic or microaerophilic; typically reduce sulphate or thiosulphate to sulphide; cladistically more closely related to Aquificales than to Thermotogales; chemolithoautotrophs, less often fermentative; OM with LPS; with or without a single polar external flagellum. Acyl ester lipids may include phosphatidylinositol. Spores not formed; mesosomes are the only known cytoplasmic membrane invaginations. Typically rod-like. Phylogenetically defined as all eubacteria derived from the last common ancestor of *Aquifex* and *Thermodesulfobacterium*. **Type order Aquificales** Reysenbach 2002. **Etymology:** name a composite of *Thermo*- (hot) and *Aqui*- (water) roots of the foregoing phylogenetically defining genera. Comprises new classes Aquificia (orders Aquificales; Desulfurobacteriales Gupta and Lali 2014; Thermosulfidibacterales ord. n.), and Thermodesulfobacteriia, with spelling corrected from now invalid classes Aquificae Reysenbach 2002 and Thermodesulfobacteria (Hatchikian et al. 2001).

**New class Aquificia** Cavalier-Smith. **Description:** anaerobic or microaerophilic hyperthermophiles or thermophiles; the clade comprising the last common ancestor of *Aquifex* and *Thermosulfidibacter* and all its descendants as shown on ribosomal multiprotein trees. Type order Aquificales Reysenbach 2002. Other order Desulfurobacteriales Gupta and Lali 2014.

**New order Thermosulfidibacterales** Cavalier-Smith. **Description:** anaerobic thermophiles more closely related to *Thermosulfidibacter* than to *Thermovibrio* on multiprotein trees. Type genus *Thermosulfidibacter* Nunoura *et al.* 2008.

**New family Thermosulfidibacteraceae** Cavalier-Smith. **Description:** anaerobic thermophiles more closely related to *Thermosulfidibacter* than to *Thermovibrio* on multiprotein trees. Type genus *Thermosulfidibacter* Nunoura *et al.* 2008.

**New class Thermodesulfobacteriia** Cavalier-Smith. **Description:** anaerobic sulphate-reducing thermophiles more closely related cladistically to *Thermodesulfobacterium* than to *Aquifex*. Acyl ester or acyl ether lipids. Lack the four conserved sequence indels defining ‘Aquificae’ (Table 2 Gupta and Lali 2013; now Aquificia). Type order Thermodesulfobacteriales Hatchikian *et al.* 2002.

**New phylum Synthermota** Cavalier-Smith. **Description:** Gram-negative non-photosynthetic eubacteria; thermophilic, hyperthermophilic, or mesophilic organotrophs; outer membrane often partially separated from murein layer by inflated periplasmic space, some lineages lack LPS. Spores not formed; rod-like. Organotrophs; typically catabolise amino acids. Phylogenetically defined as all eubacteria derived from the last common ancestor of *Synergistes* and *Thermotoga*. **Etymology:** from parts of the names of the two defining genera. Type order Thermotogales Reysenbach 2002. Comprise new subphyla Synergistetes and Thermocalda:

**New subphylum Synergistetes** Cavalier-Smith. **Description:** Anaerobic, mesophilic, amino acid degrading negibacteria with LPS. Type order Synergistales Jumas-Bilak et al. 2009. Sole class Synergistia Jumas-Bilak *et al.* 2009. Circumscription and etymology same as phylum Synergistetes Jumas-Bilak *et al.* 2009.

**New subphylum Thermocalda** Cavalier-Smith. Description: Anaerobic, usually thermophilic or hyperthermophilic, rarely cold-tolerant, mostly amino acid degrading negibacteria with or without LPS. Phylogenetically defined as the clade comprising the last common ancestor of *Thermotoga*, *Caldisericum* and *Dictyoglomus* and all its descendants as shown on multiprotein trees. Type order Thermotogales Reysenbach 2002. **Etymology:** name a composite of *Thermo*- and *Cald*- roots (both meaning hot) of *Thermotoga* and *Caldisericum*. Comprises classes Thermotogia cl. n.; Dictyoglomia Patel 2012; and Caldisericia Mori *et al.* 2009 emended here:

**New class Thermotogia** Cavalier-Smith. **Description:** OM partially separated as a toga with modified composition (often without LPS) from murein by an inflated periplasmic space; usually hyperthermophilic. Defined as all bacteria phylogenetically closer to *Thermotoga* than to *Dictyoglomus*. Type order Thermotogales Reysenbach 2002. Replacement name to correct spelling of now invalid class ‘Thermotogae**’** Reysenbach 2002.

**Class Caldisericia** Mori *et al.* 2009. **Emended description:** thermophilic amino acid degrading heterotrophs or rarely (*Thermodesulfobium*) anaerobic respiring chemoautotrophs with normal negibacterial envelopes. Phylogenetically redefined as all bacteria closer to *Caldisericum* than to *Dictyoglomus*. Immotile or with single polar flagellum; rods or multicellular filaments. Type order Caldisericales Mori *et al.* 2009. Two other orders now included: Coprothermobacterales Pavan *et al.* 2018 (earlier wrongly in ‘Firmicutes’; recently given its own unnecessary monogeneric phylum and class: Pavan et al. 2018). **Thermodesulfobiales** Cavalier-Smith ord. n. **Description:** Anaerobic chemoautotrophic acidothermophilic sulphate reducers. Includes all bacteria cladistically closer to *Thermodesulfobium* than to *Coprothermobacter*. Type family Thermodesulfobiaceae Mori *et al.* 2004 (previously wrongly in Clostridia).

**Phylum Endobacteria** Cavalier-Smith 1998 (originally ranked as subphylum)**. Emended Description:** Often flagellate Eubacteria with endospores or which have secondarily lost them; ancestrally negibacteria with OM having LPS; OM and more rarely murein sometimes lost. Type order Bacillales Prévot 1953 (Approved Lists 1980). **Etymology:***Endo-* Gk inside refers to endospores + bacteria. Comprises four new classes:

**New class Halanaerobiia** Cavalier-Smith. **Description:** clade comprising anaerobes with OM with LPS, phylogenetically closer to *Halanaerobium* than to *Selenomonas*. Type order Halanaerobiales corrig. Rainey and Zhilina 1995

**New class Selenomonadia** Cavalier-Smith. **Description:** clade comprising anaerobes with an OM with LPS that are phylogenetically closer to *Selenomonas* than to *Halanaerobium*. Replaces invalid class ‘Negativicutes’ Marchandin *et al.* 2010. Type order Selenomonadales Marchandin et al. 2010. Establishing new familes Selenomonadaceae and Sporomusaceae using indels and unique proteins was useful (Campbell et al. 2015), but placing the other two families in separate orders was unjustified taxonomic inflation; family rank is sufficient for Acidaminococcaceae and Veillonellaceae. Ordinal rank should be reserved for taxa showing greater phenotypic differences and/or phyletic depth than they do.

**New class Clostridiia** Cavalier-Smith. **Description:** uniformly with only a single membrane, no OM or LPS; murein wall thin (ancestrally) or thick. Mostly anaerobes but some aerobes. Type order Clostridiales Prévot 1953 (Approved Lists 1980) (here restricted to genera with thick murein walls and no OM), Thermoanaerobacterales Wiegel 2010, Heliobacteriales ord. n., and Sulfobacillales ord. n. **Comment:** a probably polyphyletic class that should be divided into holophyletic groups when its phylogeny is better understood.

**New order Heliobacteriales** Cavalier-Smith. **Description:** photosynthetic green bacteria related to *Heliobacterium* plus non-photosynthetic endobacteria (e.g. *Carboxydothermus*, *Syntrophothermus*) more closely related on multiprotein trees to *Heliobacterium* than to *Clostridium* or *Sulfobacillus* or *Thermoanaerobacter*. Murein wall thin. Type family Heliobacteriaceae Madigan and Asao 2010.

**New order Sulfobacillales** Cavalier-Smith. **Description:** non-photosynthetic thermophilic endobacteria with thin murein layer and no OM; autotrophic or heterotrophic aerobes or syntrophic anaerobes. Phylogenetically defined as the clade comprising the last common ancestor of *Sulfobacillus* and *Symbiobacterium* and all its descendants. Type family **Sulfobacillaceae fam. n.** Cavalier-Smith. **Description:** aerobic sulphur and hydrogen oxidising autotrophic acidophiles *Sulfobacillus* or heterotrophs (*Thermaerobacter*). Phylogenetically, the clade comprising the last common ancestor of *Sulfobacillus* and *Thermaerobacter* Takai et al. 1999 on multiprotein trees. Type genus *Sulfobacillus* Golovacheva and Karavaiko 1991. Other family Symbiobacteriaceae Shiratori-Takano *et al.* 2014.

**New class Bacillia** Cavalier-Smith. **Description:** uniformly with only one membrane; no OM or LPS; murein wall thick or secondarily absent. The deepest clade comprising the last common ancestor of *Alicyclobacillus*, *Lactobacillus*, and *Mycoplasma*, but which excludes *Clostridium* and *Anaerostipes*. Type order Bacillales Prévot 1953. Comprises two new subclasses:

**New subclass Bacillidae** Cavalier-Smith. **Description:** mostly heterotrophic aerobes; murein wall thick. Type order ***Bacillales*** Prévot 1953 (Approved Lists 1980). Paraphyletic ancestors of Erysipelotrichiidae.

**New subclass Erysipelotrichiidae** Cavalier-Smith. **Description:** heterotrophs; murein walls thick or absent. The clade comprising the last common ancestor of *Turicibacter* and *Erysipelothrix*. Type order Erysipelotrichales Ludwig et al. 2010 (paraphyletic). The oldest secondarily wall-less mollicute orders Mycoplasmatales and Acholeplasmatales, are also included; but orders Ureaplasmatales, Entomoplasmatales, and Haloplasmatales are abandoned—I formally transfer Haloplasmataceae Rainey et al. in Antunes et al., 2008 to Acholeplasmatales and all genera from Ureaplasmatales and Entomoplasmatales to Mycoplasmatales.

**New superphylum Planctochlora** Cavalier-Smith. **Description:** superphylum comprising phyla Planctobacteria and Sphingobacteria, which are sisters on eubacterial multiprotein trees. Type order Planctomycetales Schlesner and Stackebrandt 1987. **Etymol:** combines elements from constituent phylum Planctobacteria and subphylum Chlorobia of Sphingobacteria.

**Phylum Planctobacteria** Cavalier-Smith 1987, 2002. **Revised Description:** non-photosynthetic negibacteria with a tendency for inflated periplasm (because of loose or partial attachment of the cytoplasmic membrane to murein), cytoplasmic membrane invaginations and partial reduction or loss of the murein sacculus. Phylogenetically defined as the last common ancestor of *Planctomyces* and *Elusimicrobium* and all its eubacterial descendants. Type order Planctomycetales Schlesner and Stackebrandt 1987. Comprises two new subphyla:

**New subphylum Elusimicrobia** Cavalier-Smith **Description:** pleomorphic glucose-fermenting rods to coccoids; normal negibacterial sacculus; only slight to moderate inflation of periplasmic space; comprise all bacteria more closely related to *Elusimicrobium* and *Endomicrobium* than to *Planctomyces*. Type order Elusimicrobiales Geissinger et al. 2010. Sole **class Elusimicrobiia cl. n.** Cavalier-Smith. **Description:** Replaces now invalid class Elusimicrobia (Geissinger et al. 2009); includes all eubacteria descended from the last common ancestor of *Elusimicrobium* and *Endomicrobium*. Type and sole order Elusimicrobiales. **Comment**: I have not validated now invalid class Endomicrobia (Zheng et al. 2018) as neither it nor the valid order Endomicrobiales is needed. *Endomicrobium* and *Elusimicrobium* are so similar phenotypically that they need only separate families (eventually more than two will be needed) within single order Elusimicrobiales; therefore I transfer *Endomicrobium* and Endomicrobiales to Elusimicrobiales.

**New subphylum Euplancta** Cavalier-Smith **Description:** Stronger tendency for periplasmic inflation than in *Elusimicrobium*; sacculus often highly modified or lost except on septum. Phylogenetically defined as last common ancestor of *Planctomyces* and *Chlamydia* and all its eubacterial descendants. Type order Planctomycetales Schlesner and Stackebrandt 1987. **Etymol:***Eu-* Gk well, true; Planct- abbreviation of Planctobacteria to emphasise that it corresponds to the original Planctobacteria clade and phylum before Elusimicrobia was added. Three new infraphyla:

**New infraphylum and class Planctomycetia** Cavalier-Smith. **Description:** mostly free living flagellated aerobes with sacculus but strong tendency for inflated periplasm and cytoplasmic membrane invagination. Phylogenetically defined as all planctobacteria cladistically more closely related to *Planctomyces* than to *Chlamydia*. Type order Planctomycetales Schlesner and Stackebrandt 1987. I also transfer into Planctomycetia order Phycisphaerale*s* Fukunaga et al. 2010 that is insufficiently distinct to merit class rank, so I do not replace invalid class Phycisphaerae (Fukunaga et al. 2009). A third planctomycete order will be needed for the deep-branching clade including anammox species like *Candidatus* Kuenenia, and a fourth for the *Candidatus* Aub clade when named.

**New infraphylum Chlamydiia** Cavalier-Smith. **Description:** highly simplified obligate intracellular energy parasites of eukaryote cells; ancestrally with entire sacculus often with murein reduced to division septum. Type order Chlamydiales Storz and Page 1971 (Approved Lists 1980). Sole class Chlamydiia Horn 2016.

**New infraphylum Opitutae** Cavalier-Smith. **Description:** mostly free living or extracellular symbionts of animal guts or protists; sacculus often reduced to septal region; microtubules sometimes present. Phylogenetically defined as all planctobacteria cladistically more closely related to *Prosthecobacter* than to *Chlamydia*. Type order Opitutales Choo et al. 2007. Comprises three classes: Verrucomicrobiia cl. n., Lentisphaeria Cho et al. 2012 and Opitutia (replaces invalid class Opitutae Choo et al. 2007). I do not accept Oligosphaeria as a separate class as its members differ only trivially in phenotype from Lentisphaerales, so deserve ranking no higher than order at most (even family might be sufficient): Oligosphaerales (Qiu et al. 2013) are therefore here formally transferred into Lentisphaeria to join Lentisphaerales. Ordinal rank for Victivallales (Cho et al. 2004) is also excessive (like ‘phylum Lentisphaerae’, it was based solely on rDNA divergence not significant phenotypic differences), so I transfer its sole family Victivallaceae Derrien et al. 2012 into Lentisphaerales Cho et al. 2004 em. Cavalier-Smith.

**New class Verrucomicrobiia** Cavalier-Smith. **Description:** Cladistically defined as planctobacteria more closely related to *Verrucomicrobium* than to *Lentisphaera* or *Opitutus*. Type order Verrucomicrobiales Ward-Rainey et al. 1996. Replaces invalid class Verrucomicrobiae Hedlund et al. 1998.

**New class Opitutia** Cavalier-Smith. **Description:** Cladistically defined as planctobacteria more closely related to *Opitutus* than to *Lentisphaera* or *Verrucomicrobium*. Type order Opitutales Choo et al. 2007. Replaces invalid class Opitutae Choo et al. 2007).

After they have been cultured and properly named at least one new subphylum will be needed for ‘Poribacteria’, *Candidatus* Hydrogenedentes, and *Candidatus* Aerophobetes.

**Phylum Sphingobacteria** Cavalier-Smith 2002a. **Revised description:** Phylogenetically defined as all descendants of the last common ancestor of *Chlorobium*, *Bacteroides* and *Gemmatimonas* as shown on the best multiprotein trees. Gracilicute negibacteria, immotile, with gliding motility or rarely swimming by external non-periplasmic flagella. Usually, murein layer is closely attached to both OM and CM, but occasionally separates locally from CM. Non-spore forming. Sphingolipids often present. Photosynthesis if present anoxygenic. Type class Sphingobacteriia Kämpfer 2012. Four subphyla:

**New subphylum Gemmatimonadetes** Cavalier-Smith. **Description**: immotile rod-shaped mesophilic negibacterial heterotrophs or phototrophs with type II reaction centres but no chlorosomes. Type class **Gemmatimonadia cl. n.** Cavalier-Smith. **Description**: motile or immotile negibacterial heterotrophs and phototrophs without chlorosomes. Replaces invalid class Gemmatimonadetes Zhang et al. 2003. Type order Gemmatimonadales Zhang et al. 2003. **Comment**: I have not validated now invalid class Longimicrobia (Pascual et al. 2016) but instead transfer Longimicrobiales Pascual et al. 2016 into Gemmatimonadia as a separate class was undesirable rank inflation.

**New subphylum Calditrichae** Cavalier-Smith. **Description**: moderately thermophilic motile negibacterial heterotrophs with single polar non-periplasmic flagellum (or immotile); anaerobes cladistically closer to *Caldithrix* and *Calorithrix* than to *Gemmatimonas* or *Chlorobium*. **Type class Calditrichia** Cavalier-Smith. **Description:** as for Calditrichae. Replaces class Calditrichae (Kublanov et al. 2017; invalid as not in a validation list and suffix incorrect) with same etymology. Genera *Caldithrix* Miroschenko et al. 2003; *Calorithrix* Kompantseva et al. 2017; both are here placed in new family Calditrichaceae Cavalier-Smith (Description as for Calditrichae; type genus *Caldithrix*) and new order Calditrichales Cavalier-Smith (Description as for Calditrichae) as these taxa of Kublanov et al. (2017) were not validated.

**New subphylum Chlorobia** Cavalier-Smith. **Description**: The clade comprising the last common ancestor of *Chlorobium* and *Cytophaga* and all its descendants as defined by the best multiprotein sequence trees. Negibacteria without inflated periplasmic space; heterotrophs or phototrophs with chlorosomes containing bacteriochlorophyll *c* and often *d* and/or *e* and type I reaction centres; sometimes flagellate, often glide. **Etymology:** plural of included genus *Chlorobium*. **Type class Chlorobiia** cl. n. Cavalier-Smith.

**New infraphylum Chlorobi** Cavalier-Smith. **Description:** Clade comprising the last common ancestor of *Chlorobium* and *Ignavibacterium* and all its descendants as defined by the best multiprotein sequence trees. Heterotrophs or phototrophs with chlorosomes. Immotile, flagellate or gliders. Unlike too-highly ranked ‘phylum Chlorobi’ (Iino et al. 2010) which was not unambiguously defined explicitly includes clade OPB within Ignavibacteria (see Hiras et al. 2016 Fig. 3). Type order Chlorobiales Gibbons and Murray 1978 (Approved Lists 1980). Two classes:

**New class Chlorobiia** Cavalier-Smith. **Description:** immotile or gliding phototrophs with type I reaction centres and chlorosomes containing bacteriochlorophyll *c* and often *d* and/or *e*. Replaces class Chlorobea Cavalier-Smith 2002a rejected by Tindall (2014). Type order Chlorobiales Gibbons and Murray 1978.

**New class Ignavibacteriia** Cavalier-Smith. **Description:** Non-photosynthetic, with flagellar genes. Replaces now invalid class Ignavibacteria Iino et al. 2010 so bears the same type.

**New infraphylum Bacteroidetes** Cavalier-Smith. **Description:** Heterotrophic; clade comprising the last common ancestor of *Bacteroides*, *Sphingobacterium*, and *Flavobacterium* and all its descendants as defined by the best multiprotein sequence trees. Type order Bacteroidales Krieg 2012. Two new superclasses: Bacteroidia, Rhodothermaeota.

**New superclass Bacteroidia** Cavalier-Smith. **Description:** non-flagellates, sometimes predatory. Phylogenetically defined as the last common ancestor of *Bacteroides*, *Sphingobacterium*, *Flavobacterium* and *Cytophaga*. Type order Bacteroidales Krieg 2012. Comprises six classes: Cytophagia Nakagawa 2012 plus Bacteroidetia cl. n. Cavalier-Smith (spelling corrected for Bacteroidia Krieg 2012 with same description and type); Flavobacteriia Bernardet 2012; Sphingobacteriia Kämpfer 2012; Chitinophagia Munoz et al. 2016 which might have been better ranked as four subclasses.

**New superclass Rhodothermae** Cavalier-Smith. **Description:** aerobes or facultative anaerobes requiring NaCl for growth; often extremophilic for salt, temperature or pH. Sometimes flagellate. Phylogenetically defined as eubacteria more closely related to *Rhodothermus* and/or *Balneola* than to *Cytophaga* or *Bacteroides*. Type order Rhodothermales (Munoz et al. 2016). Was too highly ranked as phylum Rhodothermaeota. Two classes: Balneolia Munoz et al. 2016; Rhodothermia Munoz et al. 2016.

**New subphylum Fibrobacteres** Cavalier-Smith. **Description**: immotile negibacteria without inflated periplasmic space; heterotrophs, mostly cellulose-digesters. Type class Fibrobacteria (Spain et al. 2010), with spelling here corrected to Fibrobacteriia. Compositionally identical to too-highly ranked phylum Fibrobacteres.

This classification retains the long-used names Fibrobacteres, Bacteroidetes and Chlorobi at lower and more reasonable ranks.

**New subphylum Cloacimonetes** Cavalier-Smith. **Description**: heterotrophic negibacteria more closely related on multiprotein trees to *Candidatus* Cloacimonas acidaminovorans (Pelletier et al. 2008) the type species than to *Fibrobacter* or to *Bacteroides*.

**Phylum Proteobacteria** Cavalier-Smith 2002a. **Revised description:** negibacteria with closely attached OM, murein and cytoplasmic membrane (CM); CM sometimes with invaginations that unlike in Planctobacteria never contain murein. Frequently with external non-periplasmic flagella, polar or multiple. Anoxygenic photosynthetic, heterotrophic or chemosynthetic; aerobes or anaerobes. Phylogenetically defined as last common ancestor of *Rhodospirillum*, *Chloracidobacterium*, *Geovibrio* and all its descendants. Type order Enterobacterales Adeolu et al. 2016. Three subphyla:

**Subphylum Rhodobacteria** Cavalier-Smith 2002a. **Revised description:** purple photosynthetic bacteria with invaginated CM chromatophores and type II reaction centres plus their non-photosynthetic relatives; phylogenetically defined as the last common ancestor of *Rhodospirillum*, *Myxococcus*, and *Nitrospina*. Type order Rhodospirillales Pfennig and Trüper 1971 (Approved Lists 1980). Includes α-, β-, δ-, γ-, and ζ-proteobacteria and *Nitrospina*. Comprises five new classes: Caulobacteria, Chromatiia, Mariprofundia, Myxococcia, Nitrospinia:

**New class Caulobacteria** Cavalier-Smith. **Description:** ancestrally photosynthetic; purple non-sulphur bacteria and non-photosynthetic relatives. The clan comprising the last common ancestor of *Rhodospirillum* and *Pelagibacter* and all its non-mitochondrial descendants, i.e. all α-proteobacteria. Type order Caulobacterales Henrici and Johnson 19 35 (Approved Lists 1980). Replaces rejected class Alphabacteria Cavalier-Smith (2002a) and invalid class Alphaproteobacteria Garrity et al. 2006.

**New class Chromatiia** Cavalier-Smith. **Description:** ancestrally photosynthetic; purple sulphur bacteria and non-photosynthetic relatives. The clade comprising the last common ancestor of *Chromatium* and *Acidithiobacillus* and all its descendants. Flagella polar or peritrichous with 11 protofilaments. Type order Chromatiales Imhoff 2005. Replaces rejected class Chromatibacteria Cavalier-Smith 2002a. Three subclasses:

**New subclass Acidithiobacillidae** Cavalier-Smith. **Description:** non-photosynthetic; proteobacteria cladistically more closely related to *Acidithiobacillus* than to *Chromatium*. Type order Acidithiobacillales Garrity et al. 2005. Class Acidithiobacillia Williams and Kelly 2013 was too highly ranked.

**New subclass Neisseriidae** Cavalier-Smith. **Description:** ancestrally photosynthetic proteobacteria cladistically more closely related to *Neisseria* than to *Chromatium* and *Pseudomonas*; all β-proteobacteria. Type order Neisseriales Tønjum 2006. Now invalid class Betaproteobacteria Garrity *et al.* 2006 was too highly ranked.

**New subclass Pseudomonadidae** Cavalier-Smith. **Description:** ancestrally photosynthetic proteobacteria cladistically more closely related to *Chromatium*, *Xylella*, *Escherichia*, and *Pseudomonas* than to *Neisseria*: all γ-proteobacteria. Type order Pseudomonadales Orla-Jensen 1921 (Approved Lists 1980). Now invalid Gammaproteobacteria Garrity *et al.* 2005 was much too highly ranked.

**New class Mariprofundia** Cavalier-Smith. **Description:** non-photosynthetic iron-oxidising chemolithotrophs with thin iron oxide filaments many times cell length. Type genus *Mariprofundus* Emerson et al. 2010. Order Mariprofundales Makita et al. 2010 is not yet validly published. Replaces invalid class Zetaproteobacteria (Makita et al. 2017).

**New class Myxococcia** Cavalier-Smith. **Description:** non-photosynthetic eubacteria cladistically closer to *Myxocccus* than to *Chromatium* or *Rhodospirillum*; all δ-proteobacteria. Type order Myxococcales Tchan et al. 1948 (Approved Lists 1980). Replaces rejected Deltabacteria (Cavalier-Smith 2002a) and invalid Deltaproteobacteria Kuever et al. 2006. To encompass their diversity I divide Myxococcia into three new subclasses (all strongly supported clades), but reduce the orders from nine to five:

**Myxococcidae subcl. n.** Cavalier-Smith. **Description:** non-photosynthetic eubacteria cladistically closer to *Myxocccus* than to *Geobacter* or *Bdellovibrio.* Type and sole order Myxococcales Tchan et al. 1948 (Approved Lists 1980).

**Geobacteridae subcl. n.** Cavalier-Smith. **Description:** non-photosynthetic eubacteria cladistically closer to *Geobacter* than to *Myxocccus* or *Bdellovibrio.* Type order Desulfuromonadales corrig. Kuever et al. 2006; only other order Desulfobacterales Kuever et al. 2006 em. Cavalier-Smith (to which I transfer all genera included in orders Desulfovibrionales corrig. Kuever et al. 2006, Desulfoarcuales corrig. Kuever et al. 2006, and Syntrophobacterales Kuever et al. 2006, none different enough for separate ordinal rank).

**Oligoflexidae subcl. n.** Cavalier-Smith. **Description:** non-photosynthetic eubacteria cladistically closer to *Oligoflexus* than to *Geobacter* or *Bdellovibrio.* Type order Oligoflexales Nakai et al. 2014 em. Cavalier-Smith (to which I transfer family Silvanigrellaceae Hahn et al. 2017 as separate order Silvanigrellales Hahn et al. 2017 is unnecessary). Only other order: Bdellovibrionales Garrity et al. 2006 em. (to which I transfer Bacteriovoracaceae Davidov and Jurkevitch 2004 and Halobacteriovoraceae Koval et al. 2015, as separate order Bacteriavoracales Hahn et al. 2017 is unjustified, and from which I exclude *Vampirovibrio*, which is a melainabacterium (Soo et al. 2015b). I do not accept class Oligoflexia (Nakai et al. 2014) as it nests within other δ-proteobacteria on a 73-protein 93-taxon 83-proteobacteria ML tree Hahn et al. 2017; as that tree better samples Acidobacteria and Bdellovibrionales and better matches site-heterogeneous Fig. 5 and has 81% support for Rhodobacteria it is probably more reliable than their contradictory 85-taxon one in which Acidobacteria weakly go into Rhodobacteria.

**New class Nitrospinia** Cavalier-Smith. **Description:** negibacteria more clsely related to *Nitrospina* than to *Geobacter* or *Chromatium.*Type family Nitrospinaceae Garrity et al. 2006.

**New subphylum Acidobacteria** Cavalier-Smith (2002a as class). **Description:** mostly non-photosynthetic; some photosynthetic with chlorosomes and type I reaction centres. Type order Holophagales Fukunaga *et al.* 2008. Two classes: Blastocatellia Pascual et al. 2016 emended here; Nitrospiria cl. n. Cavalier-Smith:

**Class Blastocatellia** Pascual et al. 2016. **Emended description:** redefined as the common ancestor of *Blastocatella*, *Acidobacterium*, *Holophaga*, and all acidobacteria descended from it; heterotrophs or photosynthetic. Type order Blastocatellales Pascual et al. 2016. Two other included orders: Holophagales Fukunaga *et al.* 2008; **Terriglobales** Cavalier-Smith ord. n. **Description:** heterotrophic soil bacteria; defined as all bacteria phylogenetically closer to *Terriglobus* and *Acidobacterium* than to *Blastocatella* or *Holophaga*. Type genus *Terriglobus* Eichorst et al. 2007. Comprises Acidobacteriaceae Thrash and Coates 2012 and **new family Terriglobaceae** Cavalier-Smith. **Description:** Phylogenetically defined as acidobacteria cladistically closer to *Terriglobus* than to *Acidobacterium*. Type genus *Terriglobus* Eichorst et al. 2007. **Comment:** This revision of Blastocatellia by adding two orders makes it identical in concept to the original class Acidobacteria Cavalier-Smith (2002a) since rejected and also solves the problem of order Acidobacteriales also being rejected (making it impossible to validate Acidobacteriia as a class) and class Holophagae Fukunaga *et al.* 2008 being invalid (wrong suffix). There is no justification for 3 classes for these very similar soil bacteria; orders suffice.

**New class Nitrospiria** Cavalier-Smith. **Description:** non-photosynthetic heterotrophs or autotrophs; often curved rods; last common ancestor of *Nitrospira* and *Leptospirillum* and all its descendants. Flagella polar. Type order **Nitrospirales** Cavalier-Smith ord. n. **Description:** curved rods with strong tendency to spiral; Type genus *Nitrospira* Winogradsky and Winogradsky 1933 (Approved Lists 1980). Two families: **Nitrospiraceae** Cavalier-Smith **fam. n. Description:** oxidise nitrite (often also ammonium) to nitrate; thick periplasmic space with dense contents; more closely related to *Nitrospira* than to *Leptospirillum*. Type genus *Nitrospira* Winogradsky and Winogradsky 1933; **Leptospirillaceae** Cavalier-Smith **fam. n. Description:** oxidise iron; more closely related to *Leptospirillum* than to *Nitrospira*. Type genus *Leptospirillum* (ex Markosyan 1972) Hippe 2000.

**New order Thermodesulfovibrionales** Cavalier-Smith. **Description:** non-spiral rods, sometimes with two bipolar flagella or magnetotactic. Defined as Nitrospiria cladistically closer to *Thermodesulfovibrio* than to *Nitrospira*. Type genus *Thermodesulfovibrio* Henry et al.1994. **New family Thermodesulfovibrionaceae** Cavalier-Smith. **Description:** bacteria more closely related to *Thermodesulfovibrio* than to *Candidatus* Magnetobacterium. Type genus *Thermodesulfovibrio* Henry et al. 1994. Includes *Candidatus* Magnetobacterium.

**Subphylum Geobacteria** Cavalier-Smith 2002a. **Revised description:** Non-photosynthetic, mostly anaerobic respirers common in soil, and fermentative relatives. Phylogenetically defined as last common ancestor of *Nautilia* and *Deferribacter* and all its descendants. Type order Deferribacterales Huber and Stetter 2002 (the original type order Geovibriales Cavalier-Smith 2002 was unreasonably rejected and cannot be revived: Tindall 2014). Two classes:

**New class Deferribacteria** Cavalier-Smith. **Description:** anaerobic respirers cladistically more related to *Deferribacter* than to *Nautilia*. Often reduce metals, e.g. iron, manganese, arsenate. Type order Deferribacterales Huber and Stetter 2002. Two families: Deferribacteraceae Huber and Stetter 2002; **Geovibriaceae** Cavalier-Smith **fam. n. Description:** bacteria cladistically closer to *Geovibrio* than to *Deferribacter*. Type genus *Geovibrio* Caccavo *et al.* 2000.

Other order Chrysiogenales Garrity and Holt 2002. Replaces invalid classes Deferribacteres Huber and Stetter 2002 and Chrysiogenetes Garrity and Holt 2002. Class and phylum rank were excessive as Chrysiogenales (only one family, three genera) are phenotypically very similar to Deferribacterales and always sisters on RP trees, so best included in the same class.

**New class Nautiliia** Cavalier-Smith. **Description:** last common ancestor of *Nautilia* and *Campylobacter* and all its descendants, i.e. all ε-proteobacteria. Flagella single, polar, adapted for viscous media with wider C-ring than in Chromatiia and numerous other differences including at least in *Campylobacter* 7 not 11 protofilaments (Beeby 2015). Type order Nautiliales Miroshnichenko et al. 2004. Other orders Campylobacterales Garrity et al. 2006, Desulfurellales Kuever et al. 2006.

### New archaebacterial taxa

**New phylum Filarchaeota** Cavalier-Smith. **Description:** Predominantly non-methanogenic archaebacteria; mesophilic (often ammonia-oxidisers) or thermophilic. Ancestrally with Cdv (ESCRT-III related) membrane scission proteins at division septum, and FtsZ GTPase. Defined as the last common ancestor of *Thermoproteus* and Asgard archaebacteria (Zaremba-Niedzwiedzka et al. 2017) and all its archaebacterial descendants. Type order Thermoproteales Zillig and Stetter 1982. **Comment:** I refrained from adopting the earliest phylum name Sulfobacteria (Cavalier-Smith 1986) for this assemblage as it is descriptively apposite only for Sulfolobia, the other classes now included then being unknown. Comprises subphylum Crenarchaeota Cavalier-Smith 2002a and clade 'Asgardia' that should be made a subphylum when at least one genus is validly published; superphylum is too high rank—until Asgard subclades are cultivated and phenotypes established it is inappropriate to rank them, but orders or classes rather than phyla will almost certainly be high enough. Crenarchaeota should embrace all groups originally included in subphylum Filarchaeota (Cavalier-Smith 2014). Currently, the sole valid crenarchaeote class is Nitrososphaeria Stieglmeier et al. 2014, ammonia-oxidising mesophiles with FtsZ, since validly published class Crenarchaeota Cavalier-Smith 2002 was unwisely and irresponsibly rejected for no valid reason (Tindall 2014), a high-handed nomenclaturally destablising act; as its later synonym Thermoprotei Reysenbach 2002 is now also invalid under the new rules, I establish a replacement:

**New class Sulfolobia** Cavalier-Smith. **Description:** Typically hyperthermophile sulphur-oxidisers without FtsZ. The clade comprising the last common ancestor of *Sulfolobus* and *Thermoproteus* and all its descendants. Type order Sulfolobales Stetter 1989. Other orders Thermoproteales, Desulfurococcales. Fig. 4 shows that *Acidilobus* is maximally supported as sister to *Aeropyrum* within Desulfurococcales, contrary to the unresolved 16S tree of Prokofeva et al. (2009) but in agreement with a 59-RP tree (Brochier-Armanet et al. 2011). Therefore, establishing a fourth order Acidilobales (Prokofeva et al. 2009) was mistaken, as removing Acidilobaceae and Caldisphaeraceae made Desulfurococcales paraphyletic. This illustrates the danger of relying solely on an ill-resolved rDNA tree for higher taxonomy; phenotypically Acidilobaceae and Caldisphaeraceae are too similar to other Desulfurococcales to merit a separate order, so I do not accept Acidilobales but formally return both families to Desulfurococcales.

Within Nitrososphaeria, Nitrososphaerales is best regarded as replacing now invalid (see Tindall 2014) order ‘Cenarchaeales’ Cavalier-Smith 2002, so it should be expanded to include the whole thaumarchaeote clade on Fig. 5 of Stieglmeier et al. (2014), which appears to be ancestrally ammonia-oxidising. Neither hyperthermophilic ‘Aigarchaeota’ (Hua et al. 2018; Nunoura et al. 2011) nor methanogenic ‘Bathyarchaeota’ deserve phylum rank; following Cavalier-Smith's (2014) ranking, both should eventually be made orders within Nitrososphaeria, for which I retain thaumarchaeote as an informal name; ‘Korarchaeota’ should be a class not phylum.

### **Phylum Euryarchaeota** Garrity and Holt 2002

**New subphylum Thermococcia** Cavalier-Smith. **Description:** anaerobic sulphur-reducing coccoid heterotrophs without methanogenesis; flagella multiple. Comprises all archaebacteria cladistically closer to *Thermococcus* than to *Methanopyrus*. Type order Thermococcales Zillig 1988.

**New class Thermococcia** Cavalier-Smith. Description and type as for subphylum Thermococcia.

**New subphylum Halomebacteria** Cavalier-Smith. **Description:** ancestrally methanogens; methanogenesis multiply lost, generating non-methanogenic phenotypes, e.g. halophily, sulphur/nitrate reducers, or hyperacidophiles. Defined as last common ancestor of *Methanopyrus* and *Halobacterium* and all its descendants. Type order Halobacteriales. Etymology: *halo-* Gk salt; *Me* abbreviation for methane plus *-bacteria* meaning rods. Name proposed at phylum rank (Cavalier-Smith 1986), now reduced; though rejected as a class name (Tindall 2014) at subphylum rank it remains legitimate. Comprises class Methanonatronarchaeia Sorokin et al. 2018 em. Cavalier-Smith, and four new classes:

**New class Methanobacteriia** Cavalier-Smith. **Description:** methanogens more closely related to *Methanococcus* than to *Thermoplasma* or *Methanomicrobium*; walls usually of pseudomurein, sometimes with S-layer also or instead; a clade on 200-protein trees, but often paraphyletic with sparser protein sampling. Type order Methanococcales Balch and Wolfe 1981. Replaces rejected class Methanothermea (Cavalier-Smith 2002a). Methanogen class I of Brochier-Armanet et al. (2011) and invalid Methanomada of Petitjean et al. (2015).

**New class Methanocellia** Cavalier-Smith. **Description:** methanogens without pseudomurein and non-methanogenic halophiles; more closely related to *Methanocella* than to *Methanococcus* or *Thermoplasma*. Type order Methanocellales Sakai et al. 2008. Methanogen class II of Brochier-Armanet et al. (2011) plus Halobacteriales.

**New class Thermoplasmia** Cavalier-Smith. **Description:** non-methanogens or methanogens with tetraether lipids cladistically closer to *Thermoplasma* than to *Methanocella*. Type order Thermoplasmatales Reysenbach 2002. Replaces rejected class Picrophilea Cavalier-Smith 2002a and now invalid class Thermoplasmata Reysenbach 2002; unlike them it also includes Methanomassiliicoccales and *Candidatus* Aciduliprofundum boonei. The name Diaforarchaea for this clade (Petitjean et al. 2015) is invalid.

**New class Archaeoglobia** Cavalier-Smith. **Description:** Non-methanogenic sulphate- or nitrate-reducing hyperthermophiles with glycoprotein walls; tetraether lipids; with DNA gyrase and reverse gyrase. Type order Archaeoglobales Huber and Stetter 2002. Replaces rejected class Archaeoglobea Cavalier-Smith 2002a and now invalid class Archaeoglobi Garrity and Holt 2002.

**Class Methanonatronarchaeia** Sorokin et al. 2018. Extreme halophiles with methanogenesis. The clade comprising *Methanonatronarchaeum* and all halophiles cladistically closer to it than to *Methanosarcina*. Type order Methanonatronarchaeales (Sorokin et al. 2018).

**Comment.** A vast number of novel archaebacterial lineages have recently been discovered, most too recently for inclusion in our trees, and most without cultured representatives so they cannot be included yet in formal taxonomy. As for eubacteria, too many have been called new ‘phyla’ when more thorough study and wiser ranking will likely put them within existing phyla, probably all within the only two recognised here. Two clades will probably deserve recognition as new classes within phylum Filarchaeota, i.e. the clade comprising 'Marsarchaeota' (Jay et al. 2018) plus ‘Geoarchaeota’ (Kozubal et al. 2013), each of which would be more sensibly ranked as families or orders, not phyla, and ‘Verstraetarchaeota’ (Vanwonterghem et al. 2016). Within phylum Euryarchaeota, if trees continue to show its distinctness from Thermococcia, clade Stygia might reasonably be made a class rather than superclass as Adam et al. (2017) suggested.
