# Supplementary material for: Multidomain ribosomal protein trees and the planctobacterial origin of neomura (eukaryotes, archaebacteria)
Source: Protoplasma. 2020 Jan 3;257(3):621–753. doi: 10.1007/s00709-019-01442-7 (PMC7203096; doi:10.1007/s00709-019-01442-7)
Supplement: Supplementary file 1 — (PDF 1403 kb) [file 709_2019_1442_MOESM1_ESM.pdf]

Supplementary Material for:

Multidomain ribosomal protein trees and the planctobacterial origin of neomura (eukaryotes, archaebacteria)

Thomas Cavalier-Smith and Ema E-Yung Chao

Contents: Supplementary Figures S1-S17. pp. 1-23

Protein Alignments p. 24

Treefiles p. 24

Figure S1. Site-heterogeneous PhyloBayes GTR-CAT (4 gamma rates) tree for 26 ribosomal proteins from 143 eukaryotes representing all the most divergent lineages.
Alignment of 4156 amino acids; the number included for each taxon is shown after the @. Support values are posterior probabilities. 105,136 trees were summed after removing the first 20% as burnin: maxdiff 0.29708. The tree is rooted between Eozoa and neokaryotes

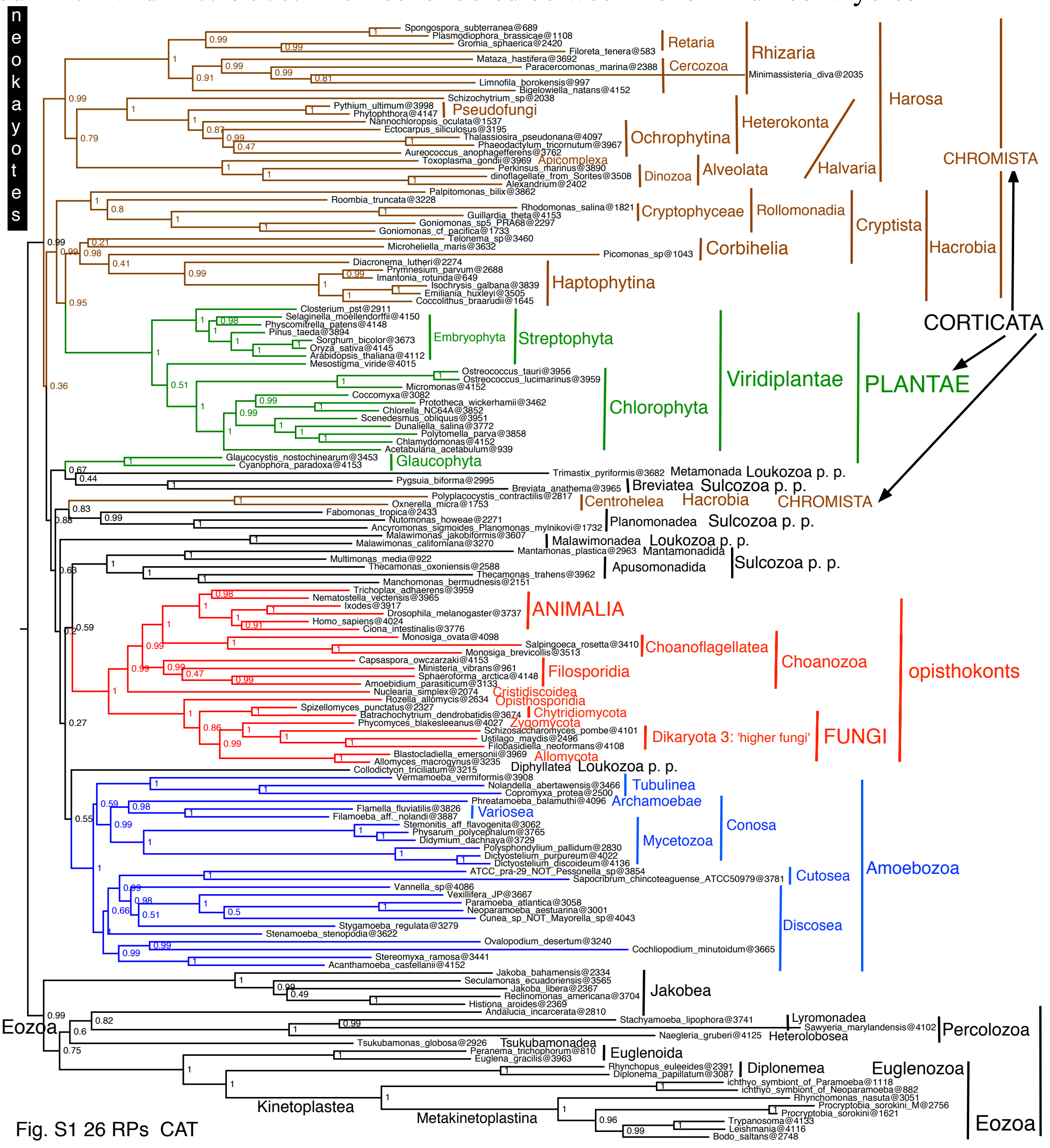

Fig. S2. Site-homogeneous RAxML PROTGAMMALGF (4 gamma rates) tree for 26 ribosomal proteins from 151 eukaryotes representing all the most divergent lineages.
Alignment of 4156 amino acids; the number included for each taxon is shown after the @. The tree is rooted between Eozoa and neokaryotes. Support values are percentages for 100 pseudoreplicates.

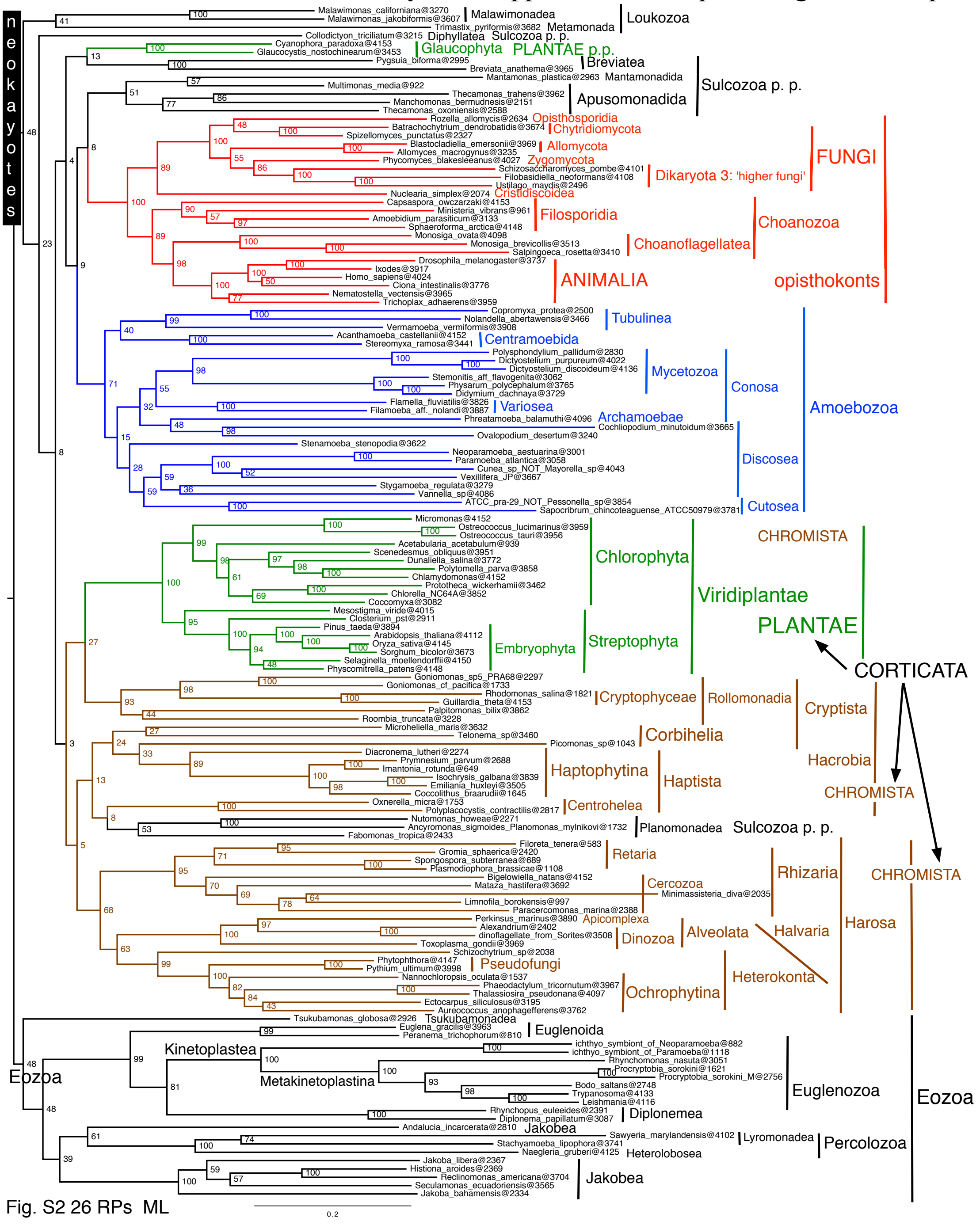

Figure S3. Site-heterogeneous PhyloBayes GTR-CAT (4 gamma rates) tree for 51 ribosomal proteins from 60 archaebacteria representing all the most divergent lineages.
This is chain 2 which placed 'Nanohaloarchaea' in a different position from chain 1 (see Fig. 4). (49, 886 trees summed after removing 40% as burnin).
Support values are posterior probabilities.

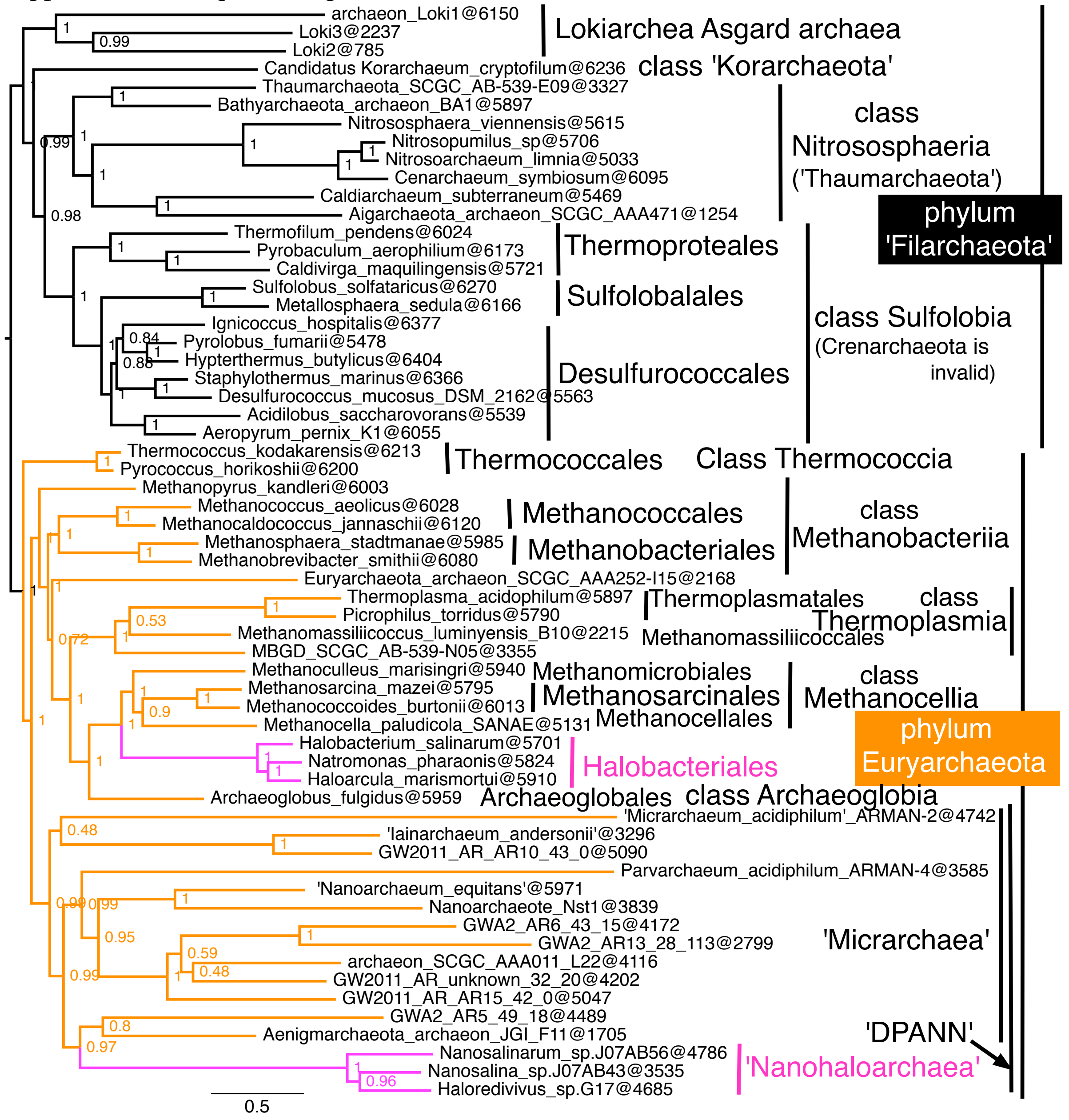

Figure S4. Site-heterogeneous PhyloBayes GTR-CAT (4 gamma rates) tree for 26 ribosomal proteins from 60 archaebacteria representing all the most divergent lineages. Support values are posterior probabilities. after removing 40% of the trees as burnin the remaining 55,580 trees from two chains were summed: maxdiff 0.0669948.

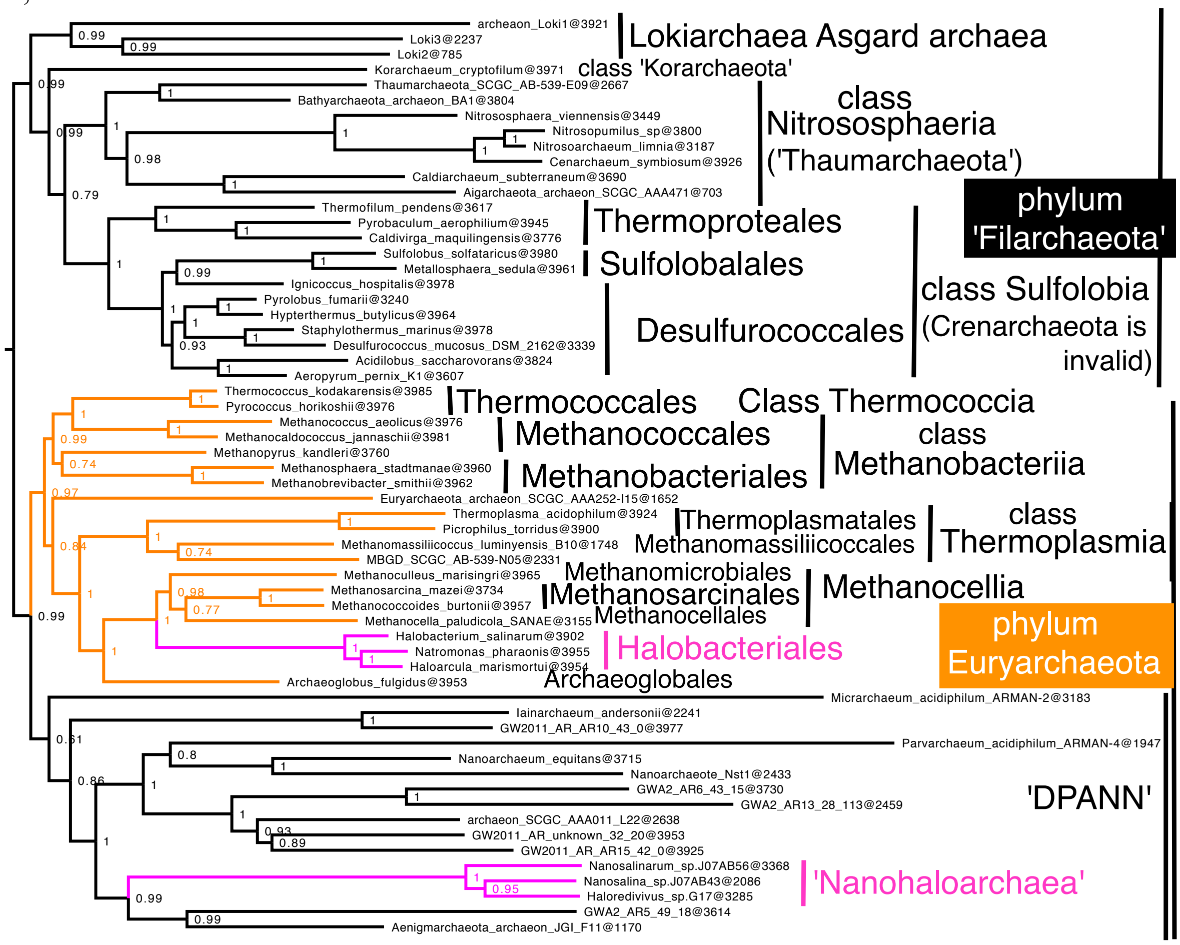

Fig. S4 26 RPs CAT

Fig. S5. Site-homogeneous RAxML PROTGAMMALGF (4 gamma rates) tree for 26 ribosomal proteins from 60 archaebacteria representing all the most divergent lineages.

Support values are percentages for 100 pseudoreplicates.

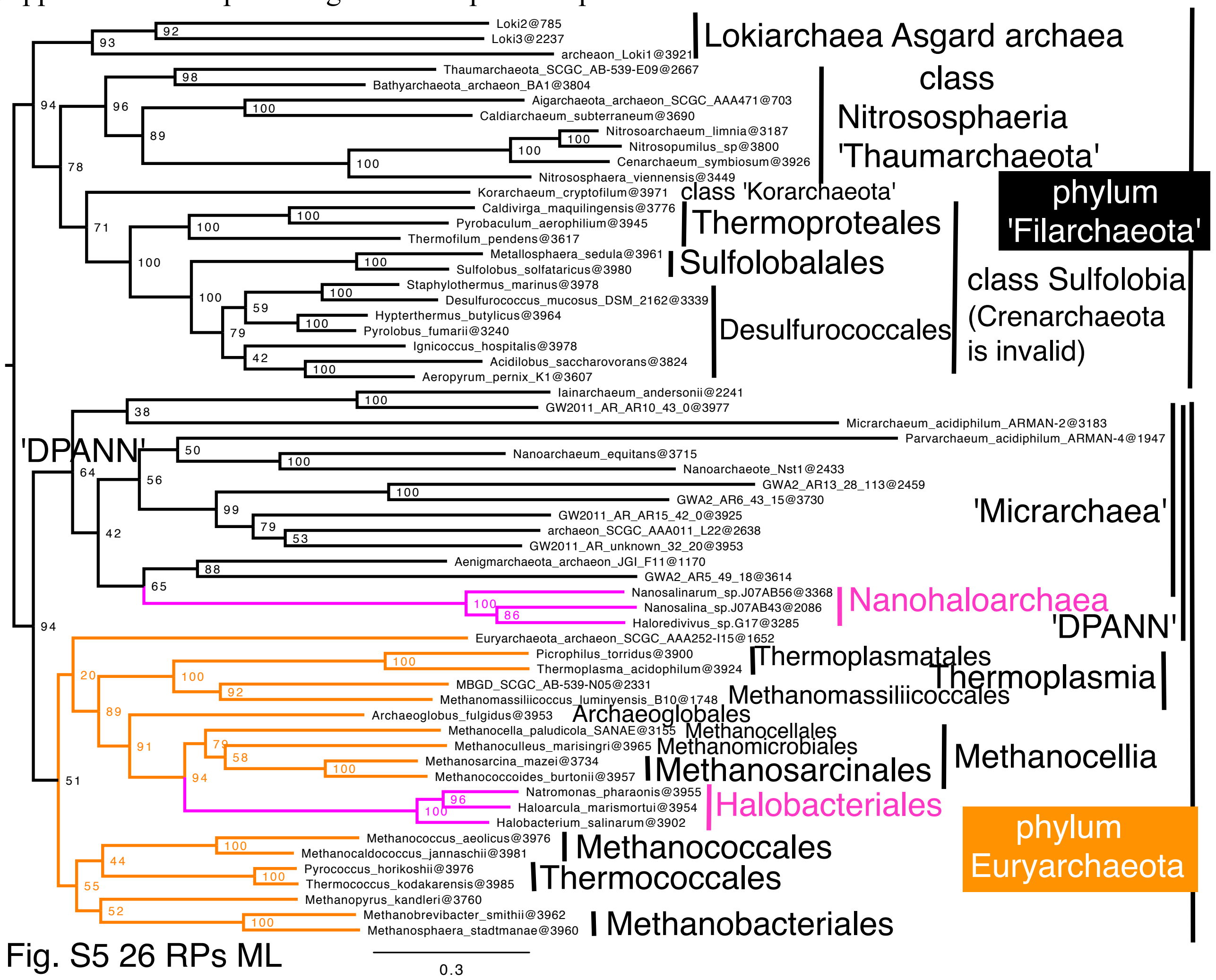

Figure S6. Site-homogeneous RAxML PROTGAMMALGF (4 gamma rates) tree for 26 ribosomal proteins from 151 eubacteria representing all the most divergent lineages. Support values are percentages for 100 pseudoreplicates.

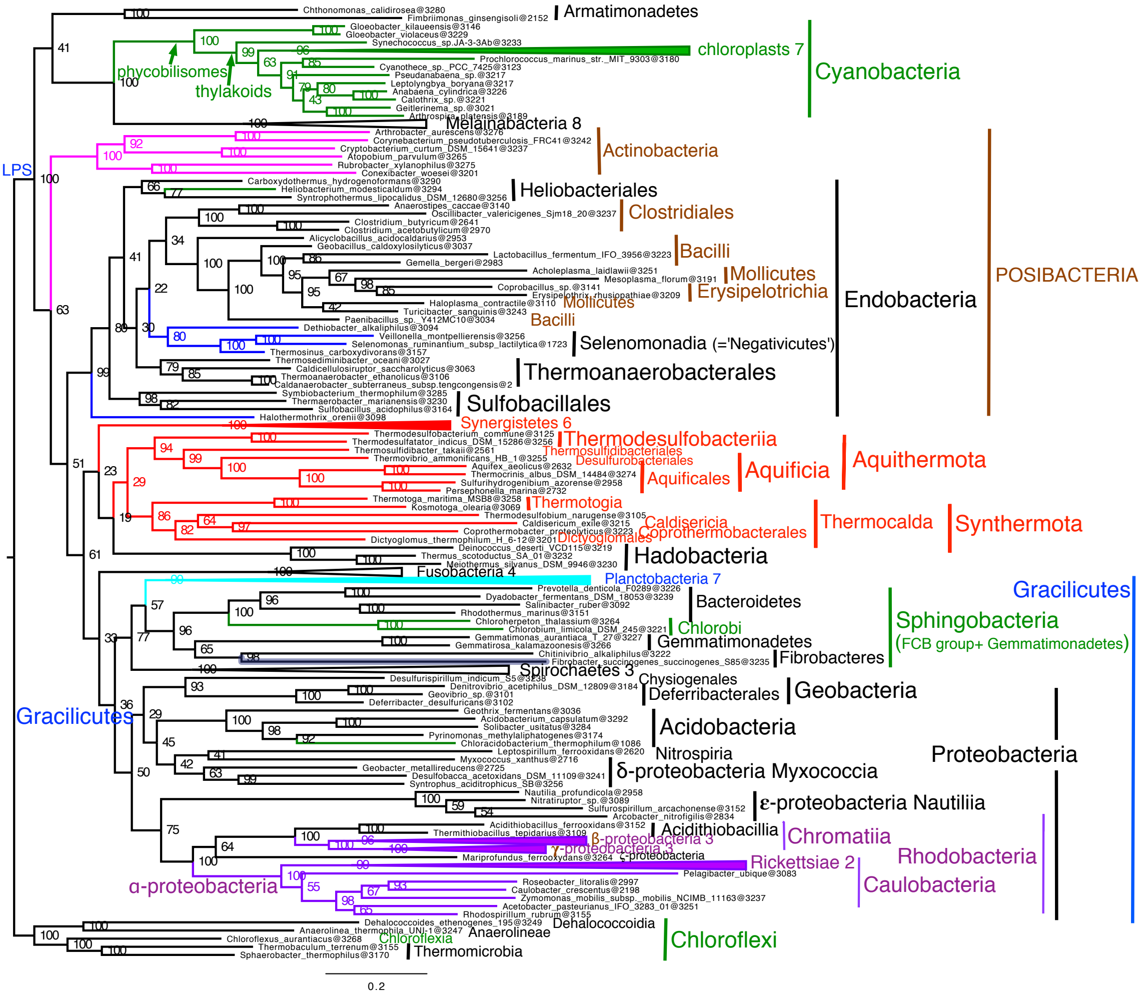

Fig. S7. Site-heterogeneous PhyloBayes GTR-CAT (4 gamma rates) tree for 26 ribosomal proteins from 203 neomura representing all the most divergent lineages. Support values are posterior probabilities.

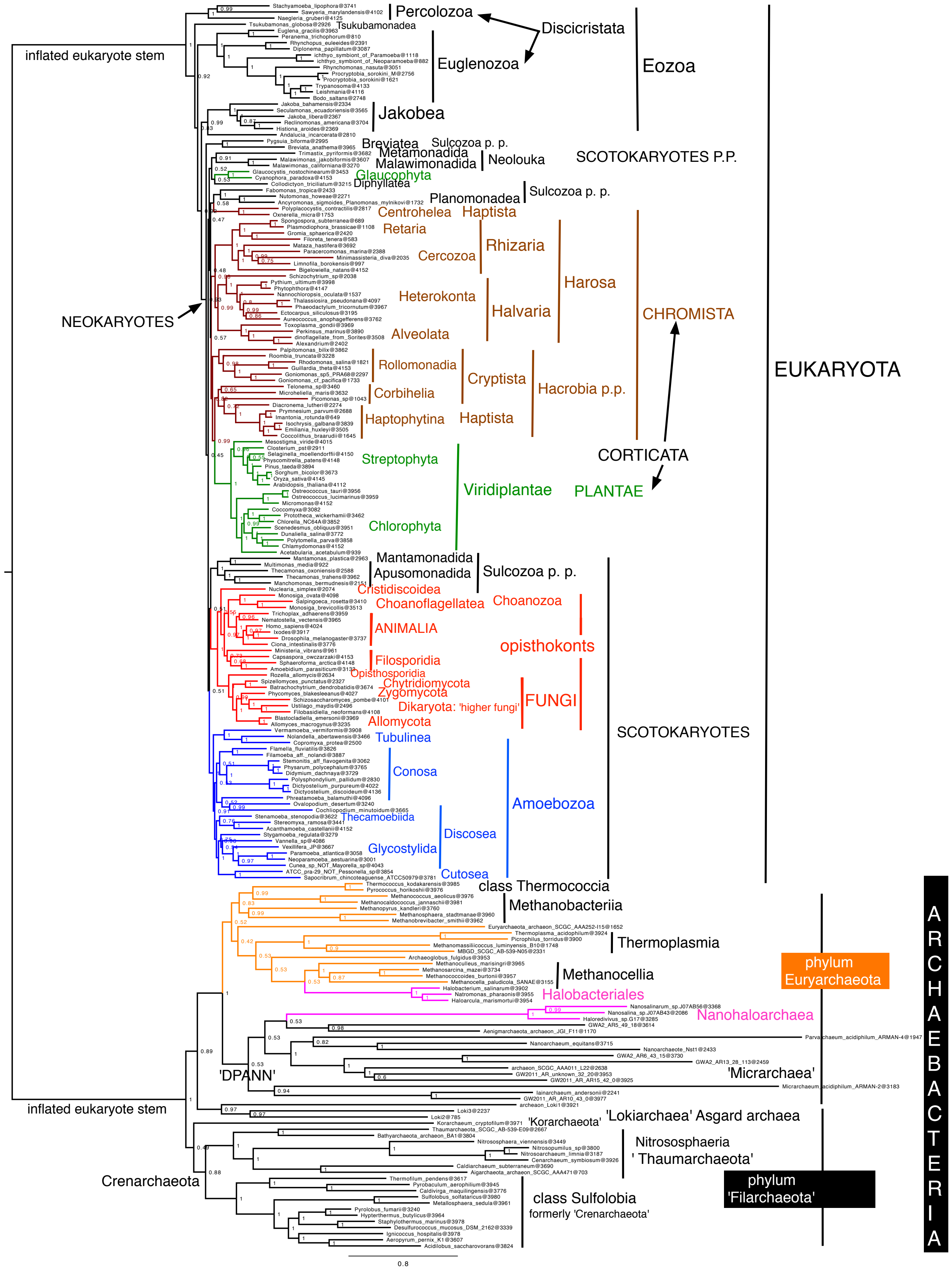

Figure S8. Site-homogeneous RAxML PROTGAMMALGF (4 gamma rates) tree for 26 ribosomal proteins from 203 neomura representing all the most divergent lineages. Support values are percentages for 100 pseudoreplicates.

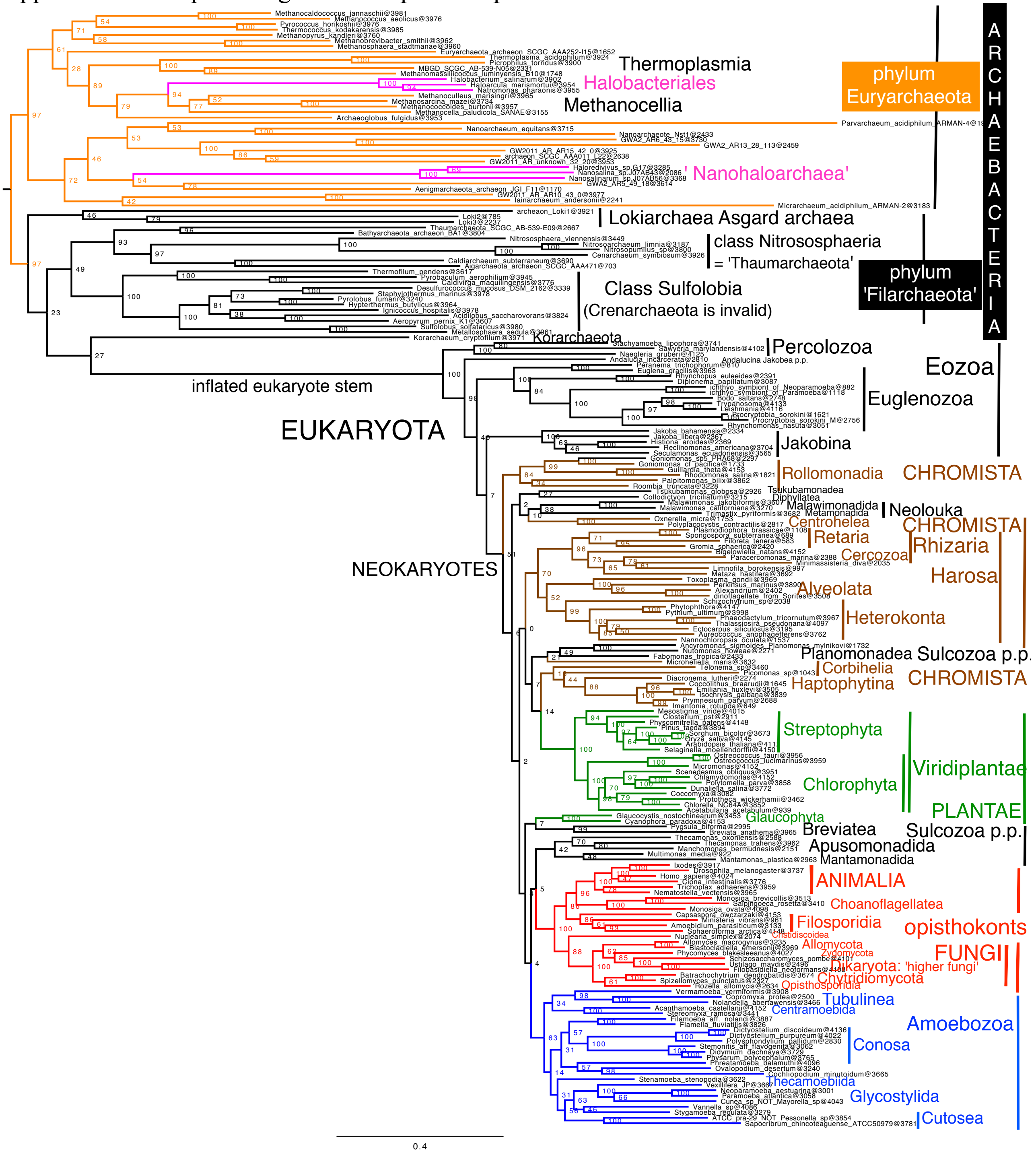

Fig. S9. Site-heterogeneous prokaryote PhyloBayes CAT-GTR tree for 51 ribosomal proteins from 60 archaebacteria and 26 ribosomal proteins from 151 eubacteria representing all the most divergent lineages, including chloroplasts. Support values are posterior probabilities. See next page:

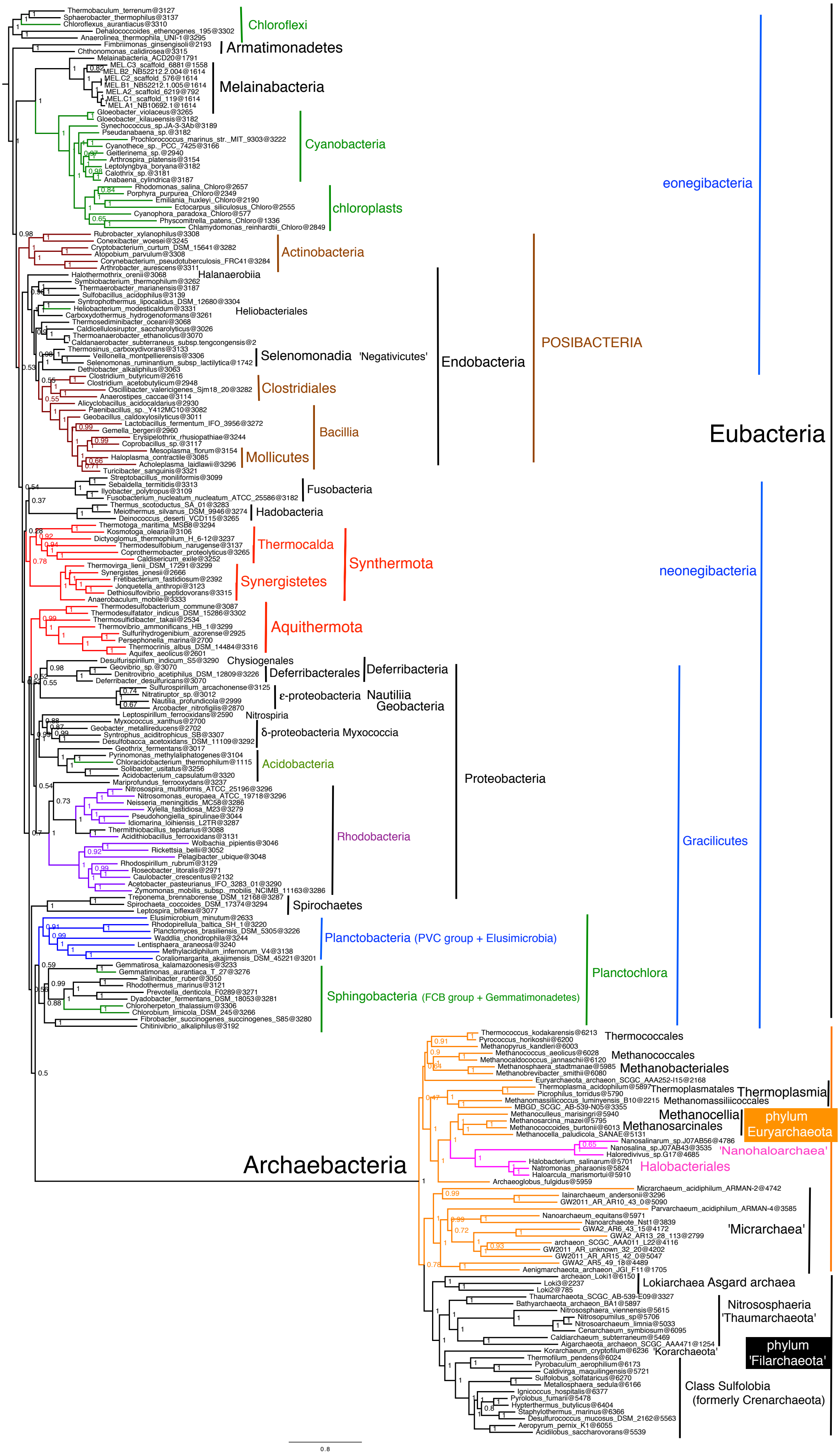
Chloroflexi
Armatimonadetes
Melainabacteria
Cyanobacteria
chloroplasts
eonegibacteria
Actinobacteria
Halanaerobiia
Heliobacteriales
Selenomonadia 'Negativicutes'
Endobacteria
POSIBACTERIA
Clostridiales
Bacillia
Mollicutes
Eubacteria
Fusobacteria
Hadobacteria
Thermocalda
Synthermota
Synergistetes
neonegibacteria
Aquithermota
Chrysiogenales
Deferribacterales
Deferribacteria
ε-proteobacteria
Nautiliia
Geobacteria
Nitrospiria
δ-proteobacteria Myxococcia
Acidobacteria
Proteobacteria
Rhodobacteria
Gracilicutes
Spirochaetes
Planctobacteria (PVC group + Elusimicrobia)
Planctochlora
Sphingobacteria (FCB group + Gemmatimonadetes)
Thermococcales
Methanococcales
Methanobacteriales
Thermoplasmatales
Thermoplasmia
Methanomassiliicoccales
Methanocellia
Methanosarcinales
phylum Euryarchaeota
'Nanohaloarchaea'
Halobacteriales
Archaebacteria
'Micrarchaea'
Lokiarchaea Asgard archaea
Nitrososphaeria 'Thaumarchaeota'
'Korarchaeota'
phylum 'Filarchaeota'
Class Sulfolobia (formerly Crenarchaeota)
0.8

Fig. S9

Figure S10. Site-homogeneous prokaryote RAxML PROTGAMMALGF (4 gamma rates) tree for 26 ribosomal proteins from 60 archaebacteria and 151 eubacteria representing all the most divergent lineages.

Support values are percentages for 100 pseudoreplicates. See next page:

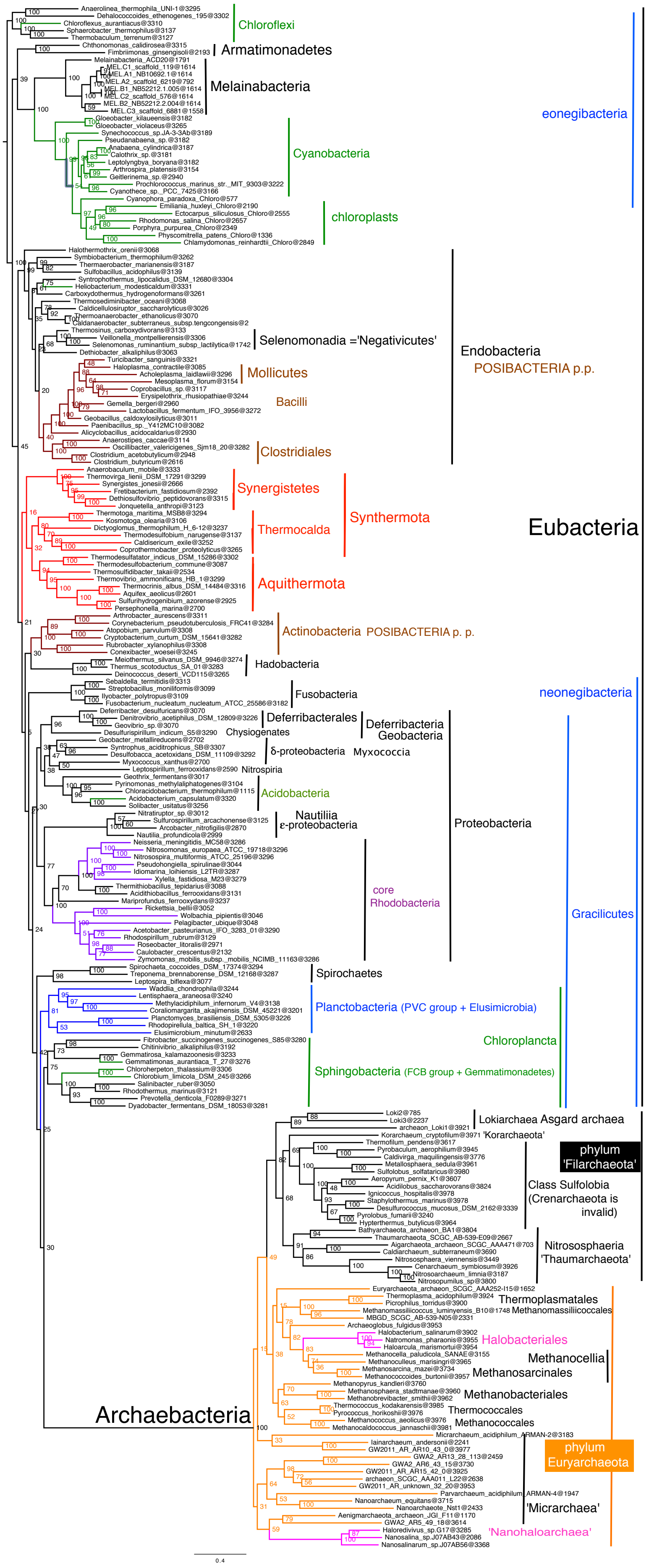
Chloroflexi
Armatimonadetes
Melainabacteria
Cyanobacteria
chloroplasts
eonegibacteria
Selenomonadia ='Negativicutes'
Endobacteria
POSIBACTERIA p.p.
Mollicutes
Bacilli
Clostridiales
Synergistetes
Synthermota
Thermocalda
Aquithermota
Eubacteria
Actinobacteria POSIBACTERIA p. p.
Hadobacteria
Fusobacteria
neonegibacteria
Deferribacterales
Deferribacteria
Geobacteria
Chysiogenates
δ-proteobacteria
Myxococcia
Nitrospiria
Acidobacteria
Nautiliia
ε-proteobacteria
Proteobacteria
core
Rhodobacteria
Gracilicutes
Spirochaetes
Planctobacteria (PVC group + Elusimicrobia)
Chloroplancta
Sphingobacteria (FCB group + Gemmatimonadetes)
Lokiarchaea Asgard archaea
'Korarchaeota'
phylum
'Filarchaeota'
Class Sulfolobia
(Crenarchaeota is
invalid)
Nitrososphaeria
'Thaumarchaeota'
Thermoplasmatales
Methanomassiliicoccales
Halobacteriales
Methanocellia
Methanosarcinales
Methanobacteriales
Thermococcales
Methanococcales
Archaebacteria
phylum
Euryarchaeota
'Micrarchaea'
'Nanohaloarchaea'
0.4

Fig. S10

Fig. S11. Site-heterogeneous 2-domain PhyloBayes CAT-GTR tree for 26 ribosomal proteins from 143 eukaryotes and 151 eubacteria representing all the most divergent lineages.
Support values are posterior probabilities. Consensus tree for two chains: 38,969 trees were summed after removing 40% as burnin. The chains converged on the same topology except for a few nodes with .5 or less support: Max diff 1. See next page:

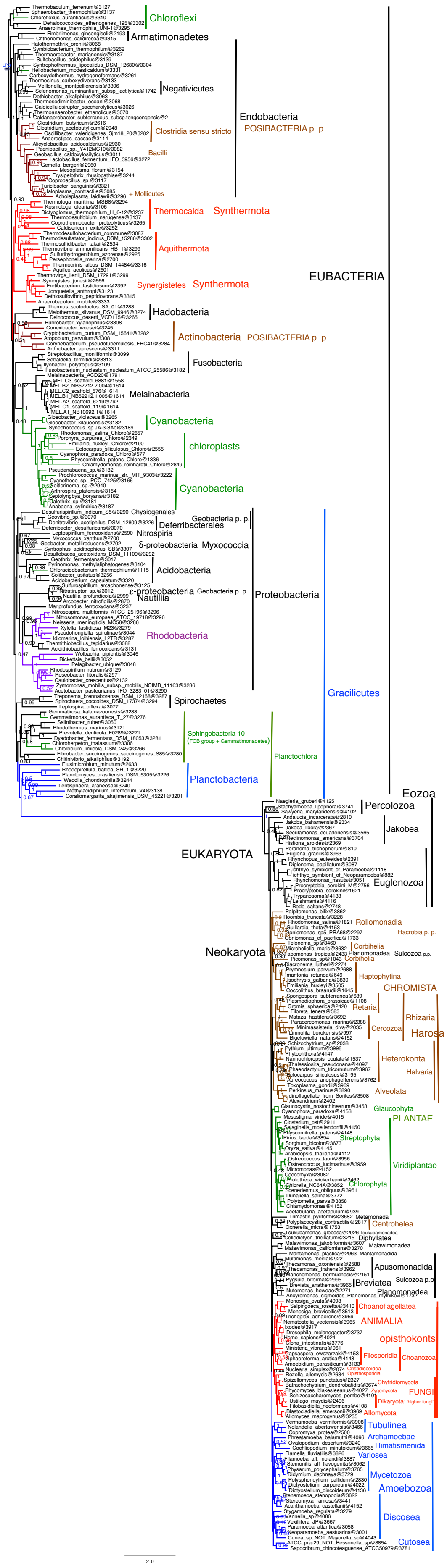
Chloroflexi
Armatimonadetes
Negativicutes
Endobacteria
POSIBACTERIA p. p.
Clostridia sensu stricto
Bacilli
+ Mollicutes
Thermocalda
Synthermota
Aquithermota
EUBACTERIA
Synergistetes
Synthermota
Hadobacteria
Actinobacteria
POSIBACTERIA p. p.
Fusobacteria
Melainabacteria
Cyanobacteria
chloroplasts
Cyanobacteria
Chrysiogenales
Geobacteria p. p.
Deferribacterales
Nitrospira
δ-proteobacteria
Myxococcia
Acidobacteria
ε-proteobacteria
Geobacteria p. p.
Nautilia
Proteobacteria
Rhodobacteria
Gracilicutes
Spirochaetes
Sphingobacteria 10
(FCB group + Gemmatimonadetes)
Planctochlora
Planctobacteria
Eozoa
Percolozoa
Jakobea
EUKARYOTA
Euglenozoa
Neokaryota
Rollomonadia
Hacrobia p. p.
Corbihelia
Planomonadea
Sulcozoa p.p.
Corbihelia
Haptophytina
CHROMISTA
Retaria
Rhizaria
Cercozoa
Harosa
Heterokonta
Halvaria
Alveolata
Glaucophyta
PLANTAE
Streptophyta
Viridiplantae
Chlorophyta
Metamonada
Centrohelea
Diphyllatea
Malawimonadea
Mantamonadida
Apusomonadida
Breviatea
Sulcozoa p.p
Planomonadea
Choanoflagellatea
ANIMALIA
opisthokonts
Filosporidia
Choanozoa
Chytridiomycota
FUNGI
Zygomycota
Dikaryota: 'higher fungi'
Allomycota
Tubulinea
Archamoebae
Himatismenida
Variosea
Mycetozoa
Amoebozoa
Discosea
Cutosea
2.0

Figure S12. Site-homogeneous 2-domain RAxML PROTGAMMALGF (4 gamma rates) tree for 26 ribosomal proteins from 143 eukaryotes and 151 eubacteria eubacteria representing all the most divergent lineages.

Support values are percentages for 100 pseudoreplicates.

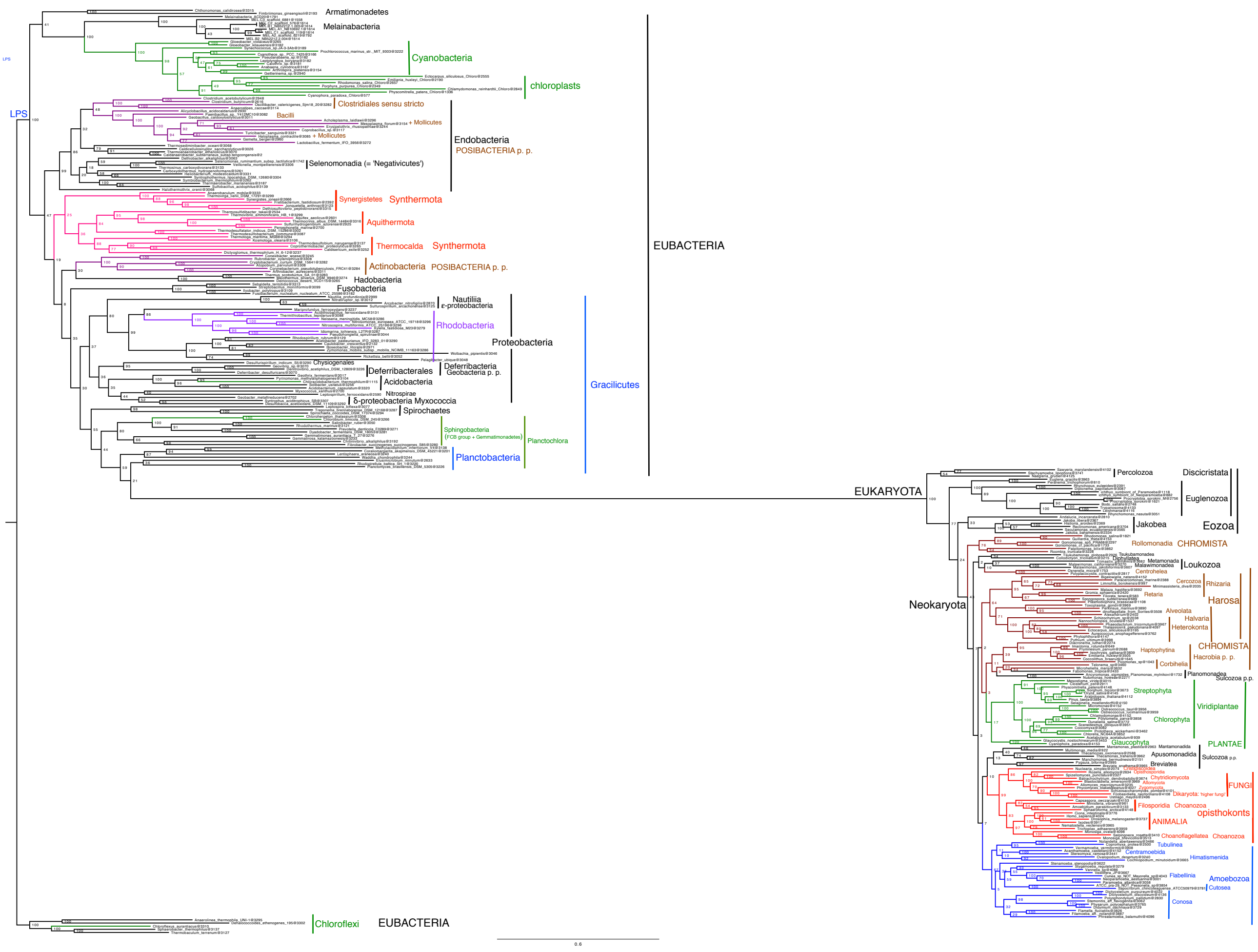

Fig. S13. Site-heterogeneous universal 3-domain PhyloBayes CAT-GTR tree for 26 ribosomal proteins from 143 eukaryotes, 60 archaebacteria, and 151 eubacteria representing all the most divergent lineages.

Support values are posterior probabilities. Two chains were summed (28,777 trees) after removing the first 40% pre-log-likelihood plateau trees as burnin; despite clear plateauing a few deep-branching topological differences (PP 0.5 or less only) remained between the two chains, making maxdiff 1. This consensus tree and chain 1 are the only trees that wrongly placed Thermodesulfobacteriaceae inside Proteobacteria, implying that in chain 1 PhyloBayes got stuck in the wrong topology and did not cope well with the large number of taxa. Chain 2 had maximum support for their being sisters of Aquificia, their usual position; the corresponding ML tree (Fig. S14) confirms this with 89% support. See next page:

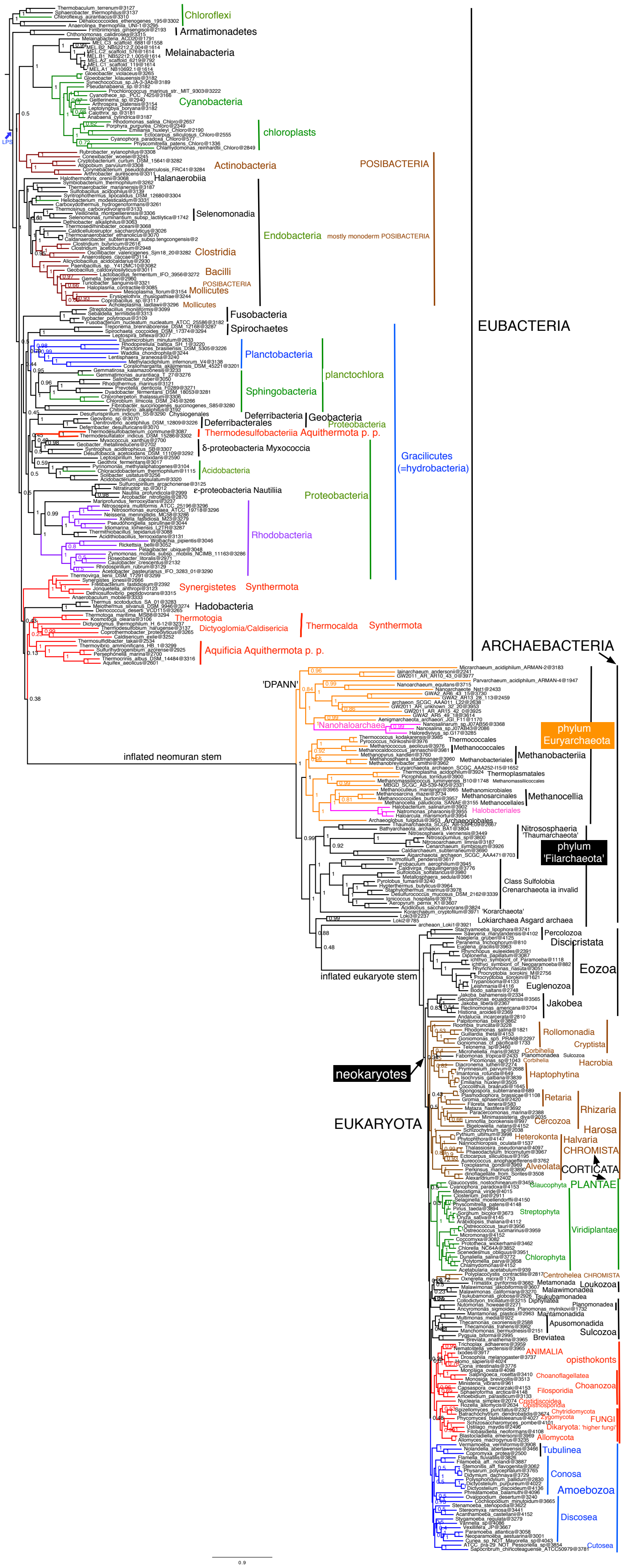
Chloroflexi
Armatimonadetes
Melainabacteria
Cyanobacteria
chloroplasts
LPS
Actinobacteria
POSIBACTERIA
Halanaerobiia
Selenomonadia
Endobacteria
mostly monoderm POSIBACTERIA
Clostridia
Bacilli
POSIBACTERIA
Mollicutes
Fusobacteria
Spirochaetes
EUBACTERIA
Planctobacteria
planctochlora
Sphingobacteria
Deferribacteria
Geobacteria
Deferribacterales
Proteobacteria
Thermodesulfobacteriia Aquithermota p. p.
δ-proteobacteria Myxococcia
Gracilicutes
(=hydrobacteria)
Acidobacteria
ε-proteobacteria Nautiliia
Rhodobacteria
Synergistetes
Synthermota
Hadobacteria
Thermotogia
Thermocalda
Dictyoglomia/Caldisericia
Aquificia Aquithermota p. p.
ARCHAEBACTERIA
'DPANN'
'Nanohaloarchaea'
phylum
Euryarchaeota
inflated neomuran stem
Thermococcales
Methanococcales
Methanobacteriales
Methanobacteriia
Thermoplasmatales
Methanomicrobiales
Methanosarcinales
Methanocellia
Methanocellales
Halobacteriales
Archaeoglobales
Nitrososphaeria
'Thaumarchaeota'
phylum
'Filarchaeota'
Class Sulfolobia
Crenarchaeota ia invalid
'Korarchaeota'
Lokiarchaea Asgard archaea
Percolozoa
Discicristata
Eozoa
inflated eukaryote stem
Euglenozoa
Jakobea
Rollomonadia
Cryptista
Corbihelia
Hacrobia
neokaryotes
Haptophytina
Retaria
Rhizaria
EUKARYOTA
Cercozoa
Harosa
Heterokonta
Halvaria
CHROMISTA
Alveolata
CORTICATA
Glaucophyta
PLANTAE
Streptophyta
Viridiplantae
Chlorophyta
Centrohelea
CHROMISTA
Metamonada
Loukozoa
Malawimonadea
Diphylatea
Planomonadea
Mantamonadida
Apusomonadida
Sulcozoa
Breviatea
ANIMALIA
opisthokonts
Choanoflagellatea
Choanozoa
Filosporidia
FUNGI
Dikaryota: 'higher fungi'
Allomycota
Tubulinea
Conosa
Amoebozoa
Discosea
Cutosea
0.9

Figure S14. Site-homogeneous universal 3-domain RAxML PROTGAMMALGF (4 gamma rates) tree for 26 ribosomal proteins from 143 eukaryotes, 60 archaebacteria, and 151 eubacteria representing all the most divergent lineages.

Support values are percentages for 100 pseudoreplicates. See next page:

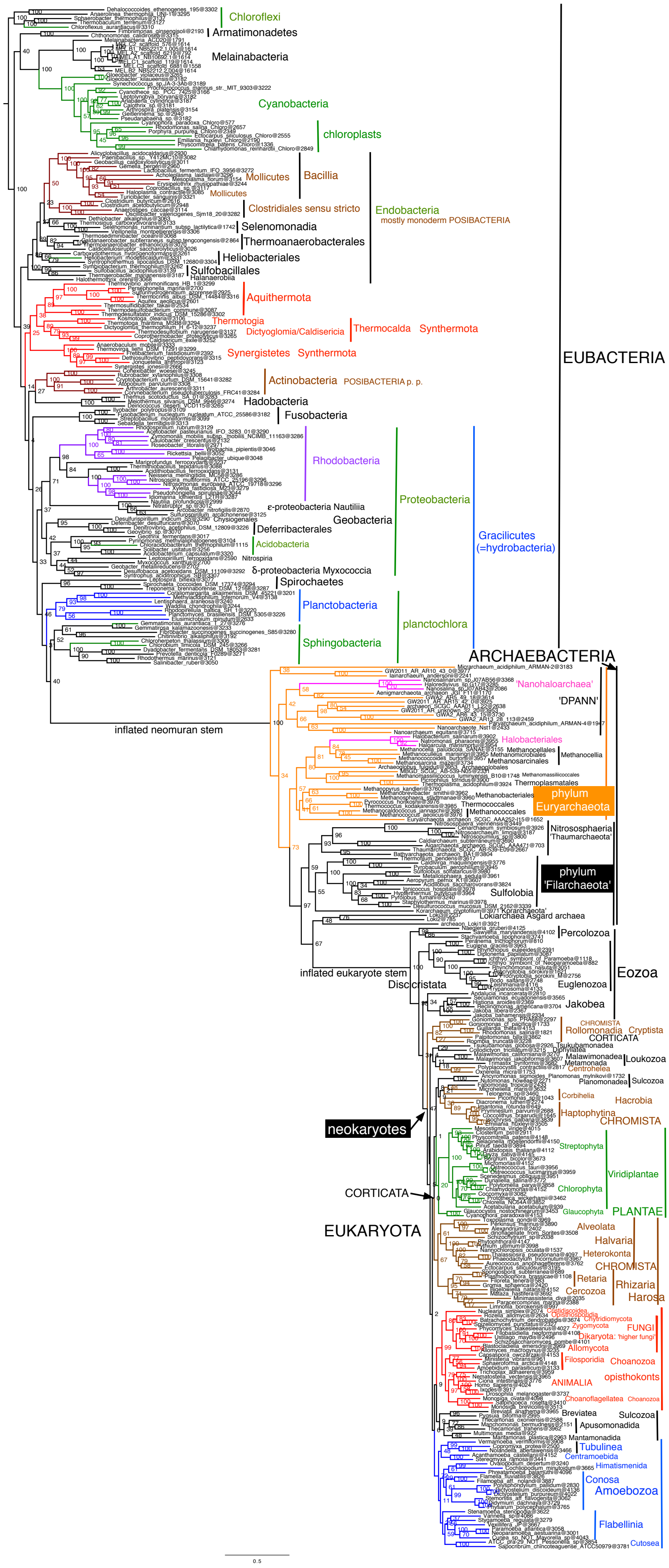
Chloroflexi
Armatimonadetes
Melainabacteria
Cyanobacteria
chloroplasts
Mollicutes
Bacillia
Clostridiales sensu stricto
Endobacteria
mostly monoderm POSIBACTERIA
Selenomonadia
Thermoanaerobacterales
Heliobacteriales
Sulfobacillales
Halanaerobiia
Aquithermota
Thermotogia
Dictyoglomia/Caldisericia
Thermocalda
Synthermota
Synergistetes
Synthermota
Actinobacteria
POSIBACTERIA p. p.
Hadobacteria
Fusobacteria
EUBACTERIA
Rhodobacteria
ε-proteobacteria Nautiliia
Geobacteria
Deferribacterales
Acidobacteria
Nitrospiria
δ-proteobacteria Myxococcia
Spirochaetes
Proteobacteria
Gracilicutes
(=hydrobacteria)
Planctobacteria
planctochlora
Sphingobacteria
inflated neomuran stem
ARCHAEBACTERIA
'Nanohaloarchaeae'
'DPANN'
Halobacteriales
Methanocellales
Methanocellia
Methanosarcinales
Thermoplasmatales
Methanobacteriales
Thermococcales
Methanococcales
phylum
Euryarchaeota
Nitrososphaeria
'Thaumarchaeota'
phylum
'Filarchaeota'
Sulfolobia
'Korarchaeota'
Lokiarchaeota Asgard archaea
inflated eukaryote stem
Discicristata
Percolozoa
Euglenozoa
Jakobea
Eozoa
CHROMISTA
Rollomonadia
Cryptista
CORTICATA
Malawimonadea
Loukozoa
Metamonada
Centrohelea
Planomonadea
Sulcozoa
Corbihelia
Hacrobia
Haptophytina
CHROMISTA
neokaryotes
Streptophyta
Viridiplantae
Chlorophyta
CORTICATA
Glaucophyta
PLANTAE
EUKARYOTA
Alveolata
Halvaria
Heterokonta
CHROMISTA
Retaria
Rhizaria
Cercozoa
Harosa
Opisthosporidia
Chytridiomycota
Zygomycota
FUNGI
Dikarya: 'higher fungi'
Allomycota
Filosporidia
Choanozoa
opisthokonts
ANIMALIA
Choanoflagellatea
Choanozoa
Breviatea
Sulcozoa
Apusomonadida
Mantamonadida
Tubulinea
Centramoebida
Himatismenida
Conosa
Amoebozoa
Flabellinia
Cutosea
0.5

Fig. S15. Site-heterogeneous PhyloBayes CAT-GTR tree for 26 ribosomal proteins from 156 eubacteria representing all the most divergent lineages with cultivated repesentatives plus Melainabacteria, chloroplasts and 5 shorter-branch mitochondria.

Support values are posterior probabilities. Two chains were summed (40,782 trees) after removing 5725 pre-log-likelihood plateau trees as burnin; despite clear plateauing a few deep-branching topological differences (PP 0.5 or less only) remained between the two chains, making maxdiff 1. The corresponding ML tree placed mitochondria one node lower as sister to all α-proteobacteria. None grouped them with the long-branch rickettsias.

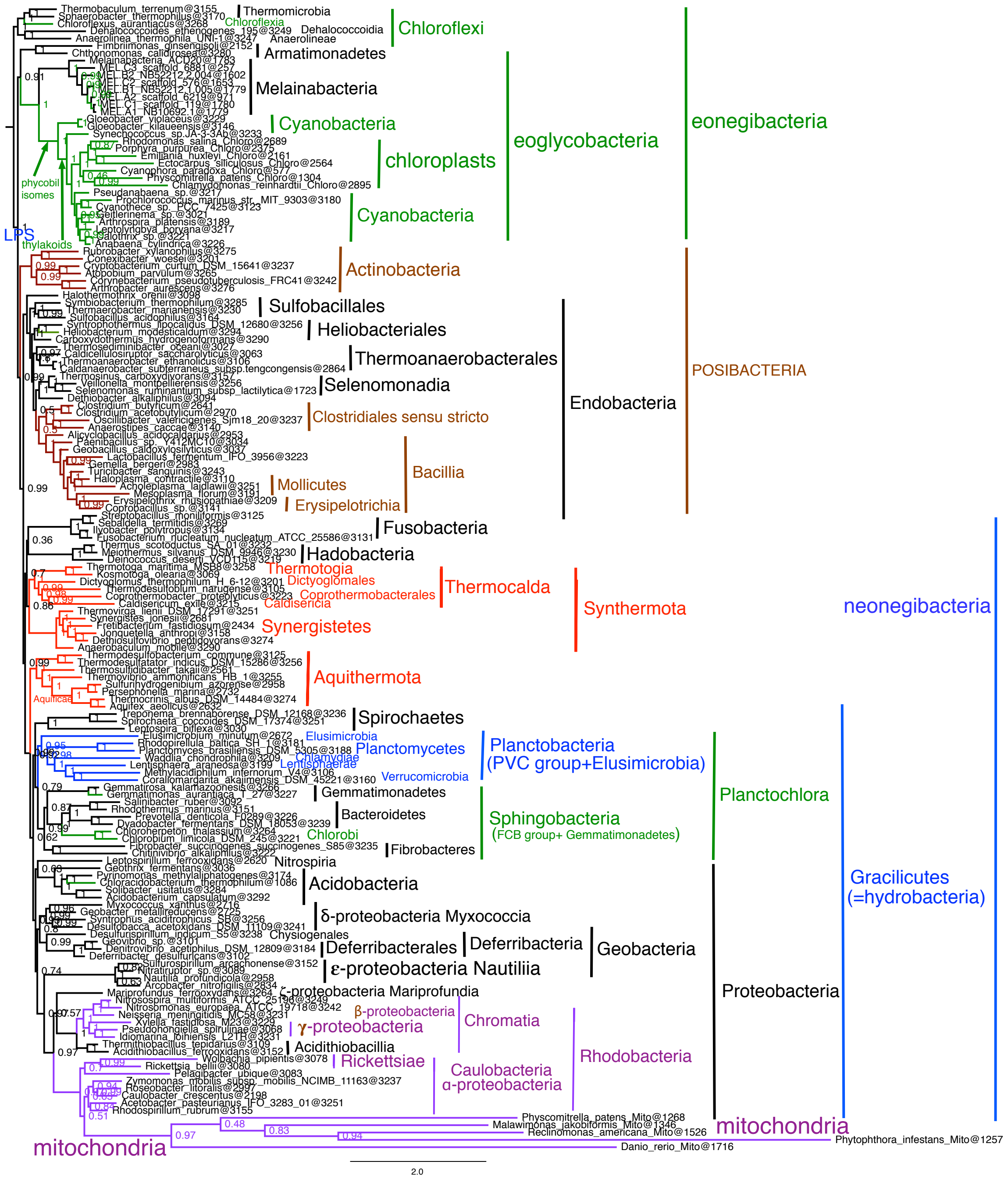

Figure S16. **Site-homogeneous RAxML-PROTGAMMALGF (4 gamma rates) tree of 305 prokaryotic structural maintenace of chromosome (SMC) proteins using 448 amino acid positions. See next page:**

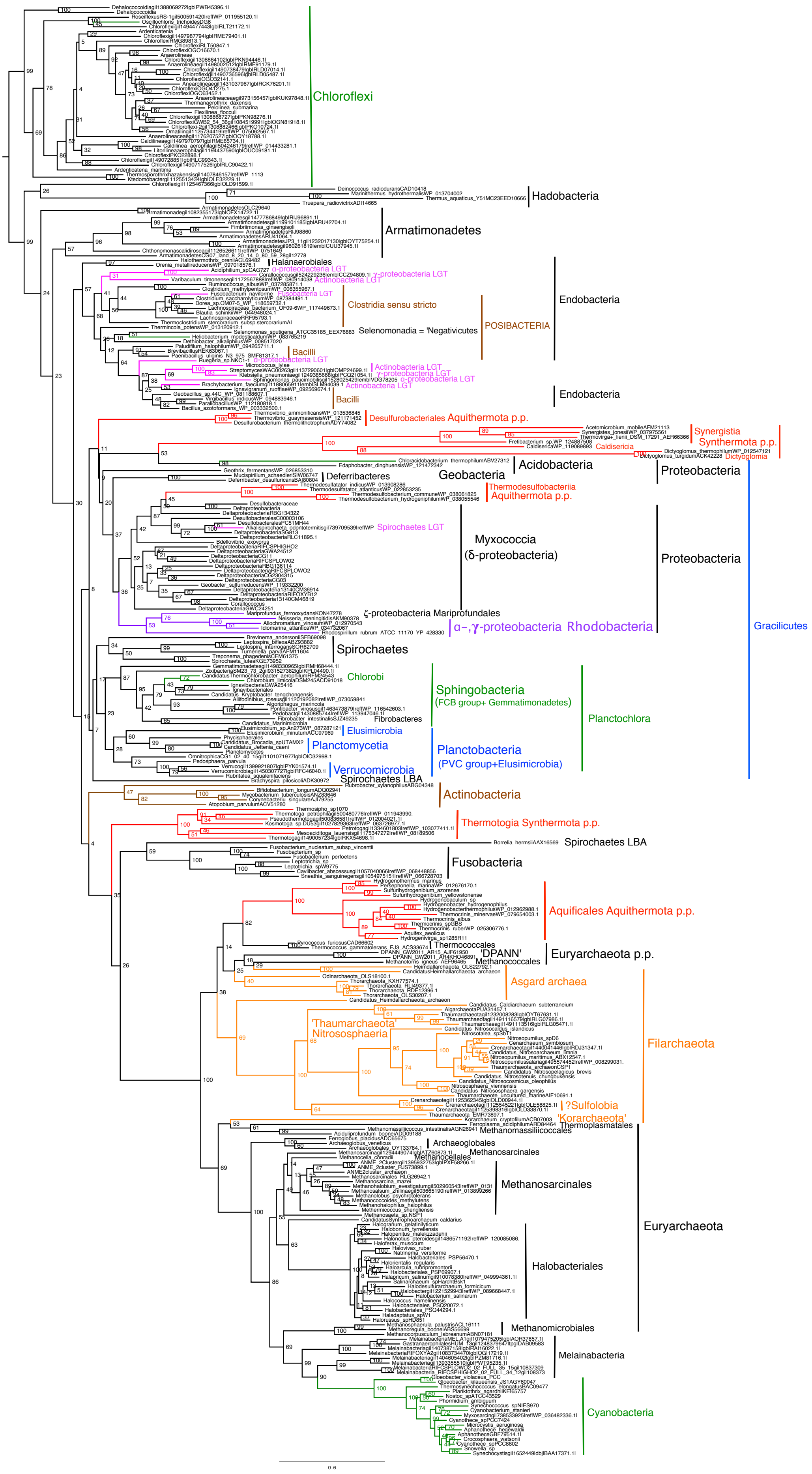
Chloroflexi
Hadobacteria
Armatimonadetes
Halanaerobiales
α-proteobacteria LGT
γ-proteobacteria LGT
Actinobacteria LGT
Fusobacteria LGT
Clostridia sensu stricto
Selenomonadia = 'Negativicutes'
POSIBACTERIA
Endobacteria
Bacilli
Desulfurobacteriales Aquithermota p.p.
Synergistia
Synthermota p.p.
Caldisericia
Dictyoglomia
Acidobacteria
Geobacteria
Deferribacteres
Proteobacteria
Thermodesulfobacteriia
Aquithermota p.p.
Spirochaetes LGT
Myxococcia
(δ-proteobacteria)
ζ-proteobacteria Mariprofundales
α-,γ-proteobacteria Rhodobacteria
Gracilicutes
Spirochaetes
Chlorobi
Sphingobacteria
(FCB group+ Gemmatimonadetes)
Planctochlora
Fibrobacteres
Elusimicrobia
Planctomycetia
Planctobacteria
(PVC group+Elusimicrobia)
Verrucomicrobia
Spirochaetes LBA
Actinobacteria
Thermotogia Synthermota p.p.
Spirochaetes LBA
Fusobacteria
Aquificales Aquithermota p.p.
Thermococcales
'DPANN'
Methanococcales
Euryarchaeota p.p.
Asgard archaea
'Thaumarchaeota'
Nitrososphaeria
Filarchaeota
?Sulfolobia
'Korarchaeota'
Thermoplasmatales
Methanomassiliicoccales
Archaeoglobales
Methanosarcinales
Methanocellales
Methanosarcinales
Euryarchaeota
Halobacteriales
Methanomicrobiales
Melainabacteria
Cyanobacteria
0.6

Figure S16

Figure S17. Site-heterogeneous PhyloBayes CAT-GTR tree of 321 largely prokaryotic structural maintenace of chromosome (SMC) proteins using 448 amino acid positions. maxdiff 0.1246. Four of the five eukaryote sequences group weakly with a relatively short-branch arsgard seuce whereas the human sequence groups with a longer-branch lokiarchaeote sequence. Some other Asgard seunces have even longer branches and group weakly with *Korarchaeum* and strongly with an almiost ceratinly artefactual grouping of Sulfolobia. *Methanopyrus* and the Synthermote eubacterium *Coprothermobacter*, the longest branch on the whole tree.

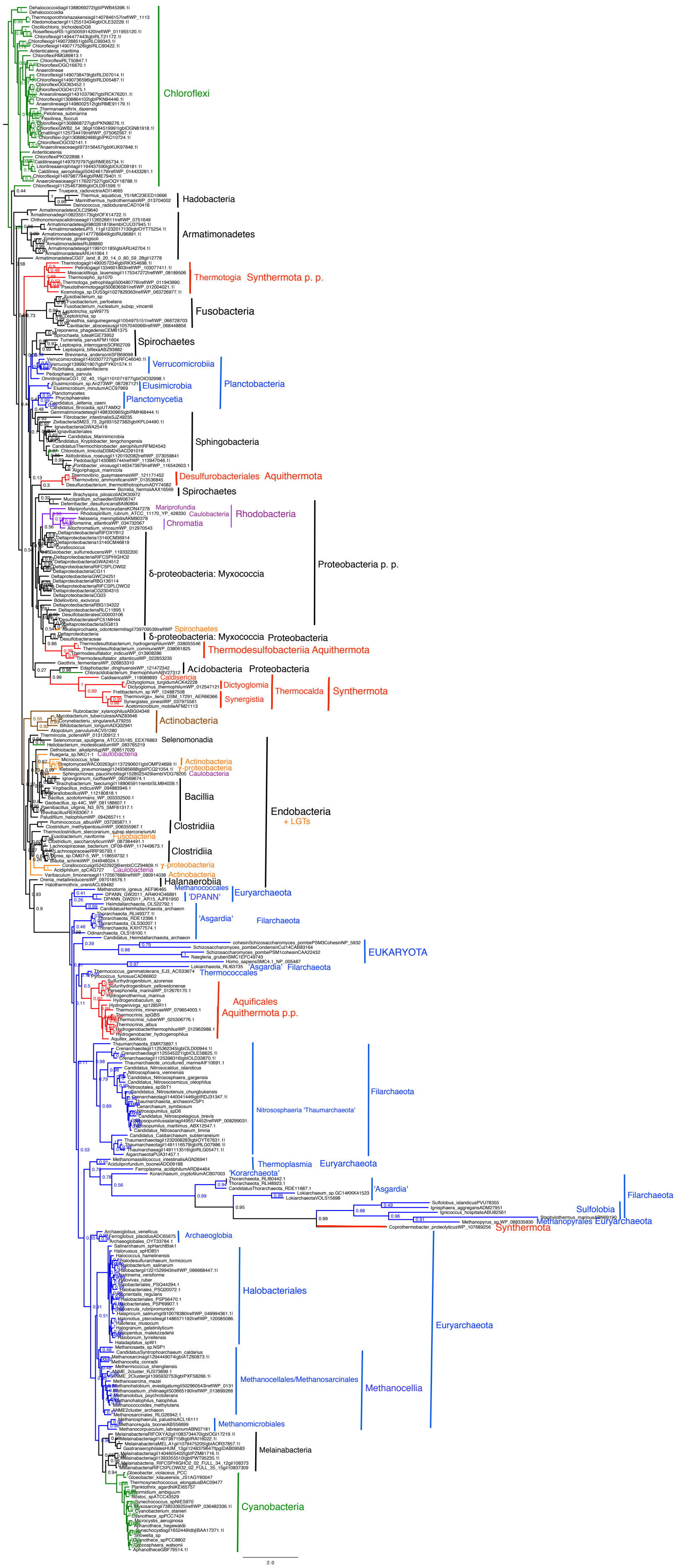
Chloroflexi
Hadobacteria
Armatimonadetes
Thermotogia Synthermota p. p.
Fusobacteria
Spirochaetes
Verrucomicrobiia
Planctobacteria
Elusimicrobia
Planctomycetia
Sphingobacteria
Desulfurobacteriales Aquithermota
Spirochaetes
Mariprofundia
Caulobacteria
Rhodobacteria
Chromatia
Proteobacteria p. p.
δ-proteobacteria: Myxococcia
Spirochaetes
δ-proteobacteria: Myxococcia Proteobacteria
Thermodesulfobacteriia Aquithermota
Acidobacteria Proteobacteria
Caldisericia
Dictyoglomia
Thermocalda
Synthermota
Synergistia
Actinobacteria
Selenomonada
Caulobacteria
Actinobacteria
γ-proteobacteria
Caulobacteria
Bacilli
Endobacteria
+ LGTs
Clostridiia
Fusobacteria
Clostridiia
γ-proteobacteria
Caulobacteria
Actinobacteria
Halanaerobiia
Methanococcales
Euryarchaeota
'DPANN'
'Asgardia'
Filarchaeota
EUKARYOTA
'Asgardia' Filarchaeota
Thermococcales
Aquificales
Aquithermota p.p.
Filarchaeota
Nitrososphaeria 'Thaumarchaeota'
Thermoplasmia
Euryarchaeota
'Korarchaeota'
'Asgardia'
Filarchaeota
Sulfolobia
Methanopyrales Euryarchaeota
Synthermota
Archaeoglobia
Halobacteriales
Euryarchaeota
Methanocellales/Methanosarcinales
Methanocellia
Methanomicrobiales
Melainabacteria
Cyanobacteria
2.0

Figure S17

Ribosomal protein (RP) alignments in fasta format as a zip files
1. These 51 **.fasta files** comprise the regions for 51 RPs after trimming as used for our trees, including all trees shown directly on Figs 3-10 plus those by other methods whose support values are included on them and include over 414 taxa, some excluded from our final analyses.

To enable readers to identify those taxa for which our alignment database uses abbreviated names or interim names, we also include the SCAFOS taxonomy (OTU) file as a key to translate from them to the 354 final names on these trees:
OTU354Arch+Eub+Euk_NoAtrichosa_28July2016.txt

2. **SMC proteins.** 305 complete SMC sequences are in GenBank format (.gb) plus a mask (at the beginning of file) in which 1s mark the 448 included positions and 0s mark those excluded from our analyses.

Tree files in Newick format in a zip file with separate subfolders for each of the nine figures.
These comprise all 22 trees used for Figs 3-10 and 12. Their titles indicate the method used (PoiPB means PhyloBayes Poisson; PB means PhyloBayes CAT-GTRGamma)
